# Yigong San Extract Modulates Metabolism, Antioxidant Status, and Immune Function to Improve Health in Diarrheic Calves

**DOI:** 10.3390/metabo15090618

**Published:** 2025-09-18

**Authors:** Sijuan Huang, Chao Han, Jianyu Lv, Xiaosong Zhang, Xuan Ni, Xin Wang, Jianfei Wang, Yunfei Ma, Zhihui Hao

**Affiliations:** 1 State Key Laboratory of Veterinary Public Health and Safety, College of Veterinary Medicine, China Agricultural University, Beijing 100193, China; b20223050398@cau.edu.cn (S.H.); s20233051040@cau.edu.cn (C.H.); s20223050978@cau.edu.cn (J.L.); zhangxsgs@cau.edu.cn (X.Z.); b20243050542@cau.edu.cn (X.N.); 2 Innovation Centre of Chinese Veterinary Medicine, College of Veterinary Medicine, China Agricultural University, Beijing 100193, China; 3 Key Biology Laboratory of Chinese Veterinary Medicine, Ministry of Agriculture and Rural Affairs, Beijing 100193, China; 4 Dairy Cattle Center, Beijing Shounong Animal Husbandry Development Co., Ltd. (Capital Agribusiness Group), Beijing 100029, China; b20223050447@cau.edu.cn (X.W.); s20243051163@cau.edu.cn (J.W.)

**Keywords:** traditional Chinese medicine, calf diarrhea, therapeutic effect, metabolomics

## Abstract

Background/Objectives: Calf diarrhea is a serious health problem in dairy farming, which seriously affects their production performance in adulthood. Diarrhea in calves is usually treated with antibiotics, which may lead to issues of antibiotic residue and resistance. Therefore, finding antibiotic alternatives is of critical importance. Yigong San (YGS) is a traditional Chinese medicine formula traditionally indicated for inflammatory gastrointestinal disorders. This study aimed to investigate whether YGS can be used as an alternative to antibiotics for the effective treatment of calf diarrhea and the underlying mechanisms. Methods: Ten healthy Holstein calves served as the control (Ctrl) group, while twenty diarrheic calves were randomly assigned to either a natural diarrhea (ND) group or a YGS treatment (YGS) group, which received YGS for seven days. Serum samples were collected post-treatment and analyzed for biochemical parameters, oxidative stress indicators, cytokine levels, and metabolomic profiles. Results: The results showed that YGS effectively alleviated diarrhea and improved abnormal biochemical indicators. YGS significantly increased serum levels of CAT, GSH-PX, and SOD, while reducing MDA levels. In addition, YGS also suppressed the expression of multiple proinflammatory cytokines, including IL-1α, TNF-α, IL-1β, IL-17A, IL-18, IL-21, IFN-γ, as well as chemokines CXCL9 and CXCL10. Metabolomic analysis revealed that YGS treatment significantly improved metabolic disorders and enriched the Arginine and Proline Metabolism pathways. The metabolites 1-methylhydantoin and ornithine were significantly and negatively correlated with pro-inflammatory cytokine levels. Conclusions: YGS effectively treats calf diarrhea by enhancing antioxidant capacity, reducing inflammatory factor levels, regulating immune function, and modulating serum metabolites. It provided valuable insights for the development of safe and effective antibiotic alternatives for preventing and treating calf diarrhea.

## 1. Introduction

Calf diarrhea represents a critical global challenge to livestock health and productivity, imposing substantial economic burdens due to its high morbidity and mortality rates. Data from the United States highlights the severity of this issue: the 2018 National Animal Health Monitoring System (NAHMS) report indicates that diarrhea accounts for 39% of calf deaths within the first three weeks of life [1]. Beyond acute mortality, surviving calves face long-term repercussions, including impaired growth (manifested as reduced pre-weaning average daily gain), delayed reproductive maturity (extending the interval to first calving by 3−6 months), and a 12−15% reduction in milk yield during their first lactation, accompanied by inferior milk components [2]. These persistent effects arise from compromised intestinal barrier function, which disrupts nutrient partitioning, induces epigenetic metabolic reprogramming, and triggers chronic immune dysregulation. Cumulatively, each diarrheic calf incurs an average lifetime economic loss of $1800, encompassing treatment expenses, diminished productivity, and premature culling [3]. Addressing this disease is therefore pivotal for enhancing the sustainability of dairy operations and improving animal welfare.

Factors contributing to calf diarrhea include infectious factors, such as intestinal pathogen infections, and non-infectious factors such as poor feeding management and low immunity [4]. Although antibiotics remain a conventional clinical strategy for the prevention and treatment of calf diarrhea, growing evidence highlights the significant risks they pose: exacerbating the spread of antimicrobial resistance in zoonotic pathogens and contributing to drug residues within the food chain [3]. Consequently, identifying effective antibiotic alternatives capable of improving gut health and controlling diarrhea is imperative for promoting sustainable and healthy livestock production.

Traditional Chinese Medicine (TCM) has emerged as a promising candidate for such an alternative, exhibiting significant anti-inflammatory, antibacterial, and immunomodulatory properties that contribute to effective disease management [5]. TCM extracts have a positive impact on livestock production when used as drugs or feed additives, effectively treating diarrhea or reducing its incidence [6,7]. Oral administration of plant extracts or natural compounds from them can also be effective in treating calf diarrhea [8,9]. Calves with diarrhea may experience inflammation and oxidative stress [10], and metabolic disorders have also been reported to be associated with calf diarrhea. [11].Metabolites from the host and gut are crucial in regulating host energy metabolism and immune responses [11]. According to research reports, calf diarrhea alters the gut metabolite profile, causing growth impairment [12].

Therefore, therapeutic agents that can ameliorate metabolic disturbances while enhancing antioxidant defenses and immune function represent a viable strategy for mitigating diarrhea and improving calf survival. Yigong San (YGS), a renowned traditional Chinese herbal formula composed of *Panax ginseng* C.A.Mey., *Citrus reticulata* Blanco, *Glycyrrhiza uralensis* Fisch. ex DC., *Poria cocos* (Schw.) Wolf and *Atractylodes macrocephala* Koidz., exhibits significant antioxidant and anti-inflammatory properties [13,14]. Key components of YGS have also been reported to confer beneficial effects in livestock, including improved weight gain, enhanced antioxidant capacity, boosted immunity, and reduced disease incidence [15,16]. However, the mechanism of action of YGS remains inadequately elucidated, particularly concerning its impact on the metabolic profile of calves. Hence, this study aimed to evaluate the therapeutic efficacy of YGS in diarrheic calves. We specifically assessed its effects on oxidative stress indicators, inflammatory cytokines, chemokines, and serum metabolite profiles. Our objective was to elucidate the underlying mechanisms by which YGS alleviates calf diarrhea. The findings are anticipated to contribute to the development of effective and safe antibiotic alternatives for the prevention and management of this economically significant disease.

## 2. Materials and Methods

### 2.1. Preparation and Chemical Composition Analysis of YGS

All herbs were commercially sourced from Beijing Meikang Hall Pharmaceutical Technology Co., Ltd. (Beijing, China), accompanied by inspection certificates of conformity (Supplemental Information S3). The herbal formulation YGS, whose complete composition is detailed in Table 1, was used for subsequent experiments. The dried herbs underwent two 2.5-h boiling extractions, each using a 12-fold volume of water (v/w). Concentrate the mixed filtrate under reduced pressure to 1 g/mL and store at 4 °C for subsequent use. Qualitative analysis of the active ingredients in YGS was conducted based on previous research [17], with the detailed mass spectrometry conditions provided in the Supplementary Information S1.

### 2.2. Animals

Twenty diarrheic Holstein calves and ten healthy calves (5−7 days old, 45−55 kg) were selected from Shounong Farm (Beijing, China). Diarrheic calves met the inclusion criteria: (1) fecal score > 2 and (2) age 1−30 days. The twenty diarrheic calves finally selected were all from the 5−7 days of age segment, matching the age of the healthy calves. All diarrheal calves were screened using antigen test cards for common infectious pathogens, including Bovine Coronavirus, Rotavirus, Cryptosporidium, Giardia, *E. coli* K99, and Bovine Viral Diarrhea Virus. Only calves that tested negative for all these infectious agents were included in the study. Calves were raised individually in calf pens. Overall, feeding management followed standard operating procedures for commercial farms. All experimental protocols were reviewed and approved by the Animal Ethics Committee of China Agricultural University (approval number: AW31605202-2-01).

### 2.3. Drug Administration

Twenty diarrheic calves were randomly allocated into two groups: a natural diarrhea (ND) group and a YGS-treated (YGS) group, with ten calves each. Details regarding random grouping procedures were provided in Supplementary Information S1. The clinical routine dosage of YGS for humans is 75 g/d [14], and the administration dose for calves, converted based on body surface area, is 0.915 g/kg. Detailed calculation steps were provided in Supplementary Information S1. Ten healthy calves serving as the control (Ctrl) group and the ND group received daily oral gavage with equivalent volumes of water. The calf did not receive any other medication during the YGS treatment. Disease remission was defined as a fecal score ≤ 1 persisting for two consecutive days. Detailed supportive care protocols and resuscitation criteria are provided in Supplementary Information S1. In short, calves received fluid and electrolyte support, nutritional support, and environmental and nursing care. After the experiment, collect blood samples from each group. During sampling, calves are kept in a standing position to minimize stress caused by the procedure. Blood was collected into plain tubes (without anticoagulant). Following centrifugation at 3200 rpm for 6 min, the resulting serum was aliquoted and stored at −20 °C for subsequent analysis.

### 2.4. Therapeutic Efficacy Observation

During the experimental period (Day 0−Day 7), as shown in Table S1, the calves’ mental status, dehydration status, and fecal consistency were observed and recorded in accordance with a previous study [18]. Therapeutic efficacy was subsequently calculated.

### 2.5. Analysis of Serum Biochemical Indices

Serum levels of aspartate aminotransferase (AST), globulin (GLB), alkaline phosphatase (ALP), lactate dehydrogenase (LDH), total protein (TP), alanine aminotransferase (ALT), creatine kinase (CK), total bile acids (TBA), total cholesterol (CHOL), uric acid (UA), amylase (AMY), phosphorus (P), iron (Fe), chloride (Cl), potassium (K), calcium (Ca), and magnesium (Mg) were measured using a Roche Cobas 6000 c501 automated clinical chemistry analyzer.

### 2.6. Oxidative Stress Indicator Detection

According to the manufacturer’s instructions, we analyzed serum levels of catalase (CAT), malondialdehyde (MDA), glutathione peroxidase (GSH-PX), and superoxide dismutase (SOD) using commercial kits (Nanjing Jiancheng Bioengineering Institute, Nanjing, China).

### 2.7. Cytokine Detection

The serum levels of IFN-α, IFN-γ, IL-13, IL-1α, IL-1F5, IL-21, CXCL10, CXCL9, CCL4, and TNF-α in calf serum were quantified using enzyme-linked immunosorbent assays (ELISA) (Catalog #QAB-CYT-1-2, RayBiotech, Norcross, GA, USA). Levels of ANG-1, Decorin, IL-1β, IL-17A, LIF, CD40 Ligand, IFN-β, IL-10, IL-18, and CCL5 were quantified using ELISA (Catalog #QAB-CYT-3-2, RayBiotech, Norcross, GA, USA). All steps were performed strictly according to the manufacturer’s protocols.

### 2.8. Preparation of Serum Samples for Metabolomic Analysis

To evaluate system stability, a quality control (QC) sample was prepared by combining equal volumes of serum from each of the three groups. For metabolite extraction, 200 µL of serum was combined with 800 µL of ice-cold methanol-acetonitrile (1:1, *v*/*v*) and vortex-mixed. The mixture was then sonicated for 1 h in an ice-water bath, incubated at −20 °C for 1.5 h, and finally centrifuged at 13,000× *g* for 25 min at 4 °C.

### 2.9. Metabolomics Analysis of Serum Samples

Serum metabolomic profiling was conducted using ultra-performance liquid chromatography coupled with quadrupole-Orbitrap mass spectrometry (UPLC-Q-Orbitrap MS). The system integrated a Nexera X2 LC-30AD UHPLC (Shimadzu, Kyoto, Japan) with a Q-Exactive Plus mass spectrometer (Thermo Scientific, San Jose, CA, USA). Chromatographic separation was performed on a HSS T3 column (2.1 × 100 mm, 1.8 μm; Waters, Milford, MA, USA) with a gradient elution program. Mass spectrometric detection was carried out in both positive and negative ionization modes with a heated electrospray ionization (HESI) source. Full MS and data-dependent MS^2^ scans were acquired at resolutions of 70,000 and 17,500, respectively.

### 2.10. Data Processing and Statistical Analysis

Raw data were processed using MS-DIAL for peak detection, alignment, and metabolite identification based on accurate mass and MS/MS spectra matching against HMDB, MassBank, and an in-house database. Features detected in more than 50% of samples in at least one group were retained. Multivariate analyses were performed in R. Metabolites with VIP > 1.0 and *p* < 0.05 were considered significant. Pathway enrichment analysis was performed using KEGG. Detailed experimental procedures and data processing workflows are provided in the Supplementary Information S1.

Data distribution normality was evaluated using GraphPad Prism 10 and SPSS (version 26.0). Data are presented as mean ± standard deviation (SD). One-way analysis of variance (ANOVA) was employed if the data were normally distributed with homogeneous variance, and a non-parametric test was used if not. Correlations were analysed using Spearman’s method via chiplot.online. Significance was set at *p* < 0.05.

## 3. Results

### 3.1. Identification of the Main Chemical Components in YGS by UPLC-MS/MS

**Figure 1:**
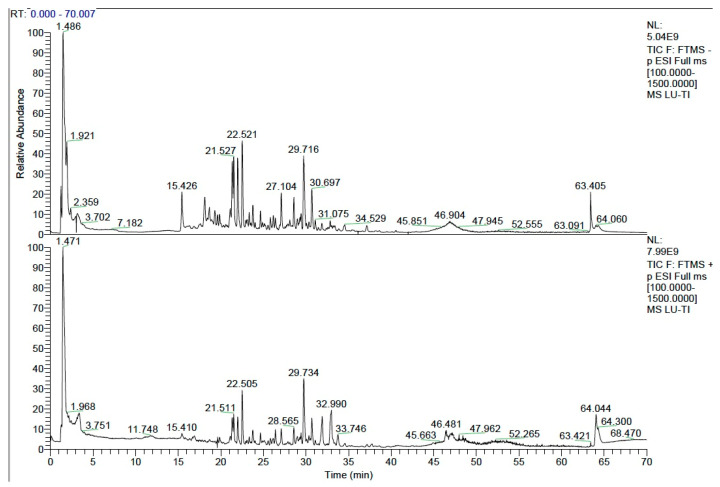
Total ion chromatograms (TIC) of active compounds in YGS extract detected by UPLC-MS/MS. The upper panel shows the TIC in negative ion mode, and the lower panel shows the TIC in positive ion mode.

The main active components in YGS were identified using the UPLC-MS/MS analytical method (Figure 1). A total of 147 primary compounds were identified (Table S2), among which the most abundant categories include flavonoids and their glycosides, phenolic acids and their derivatives, amino acids and their derivatives, terpenoids, organic acids, and alkaloids.

### 3.2. The Therapeutic Effects of YGS on Diarrheal Calves

As shown in Table 2, diarrheic calves exhibited significantly higher fecal status, mental status and dehydration scores compared to the Ctrl group (*p* < 0.05). Following YGS treatment, fecal status, mental status, and dehydration scores showed a decreasing trend on Day 7, ultimately showing no significant difference compared to the Ctrl group (*p* > 0.05). The clinical efficacy rate of YGS treatment, defined as the ratio of calves cured within 7 days to the total number of diarrheic calves treated, was 100%. We present the daily trajectories of fecal status, mental status, and dehydration scores for all 30 calves in the Table S3. We characterized the cumulative remission rate over the observation period. Results showed the YGS group achieved a 100% cumulative remission rate by D7 (Figure S1). The median time to remission was 6.5 days, with a 95% confidence interval (95% CI) of 6.13–6.88 days, confirming consistent and timely therapeutic effects. These results indicate that YGS was associated with improved clinical scores compared with ND under the conditions tested.

### 3.3. The Effects of YGS on the Biochemical Blood Indexes

We further investigated the effect of YGS administration on blood biochemical indices of diarrhea calves (Table 3). Compared to the Ctrl group, diarrheic calves exhibited significantly lower (*p* < 0.05) serum levels of ALT, ALP, and CHOL, Ca and Mg, while levels of TP, GLB, CK, AMY and UREA were significantly higher (*p* < 0.05). YGS treatment reversed previously altered indices to baseline levels (*p* < 0.05). Furthermore, no significant alterations were found in the concentrations of AST, LDH, TBA, UA, P, Cl, K, or Fe, despite the occurrence of sporadic variations (*p* > 0.05).

### 3.4. The Effects of YGS on the Oxidative Stress Indicators

**Figure 2:**
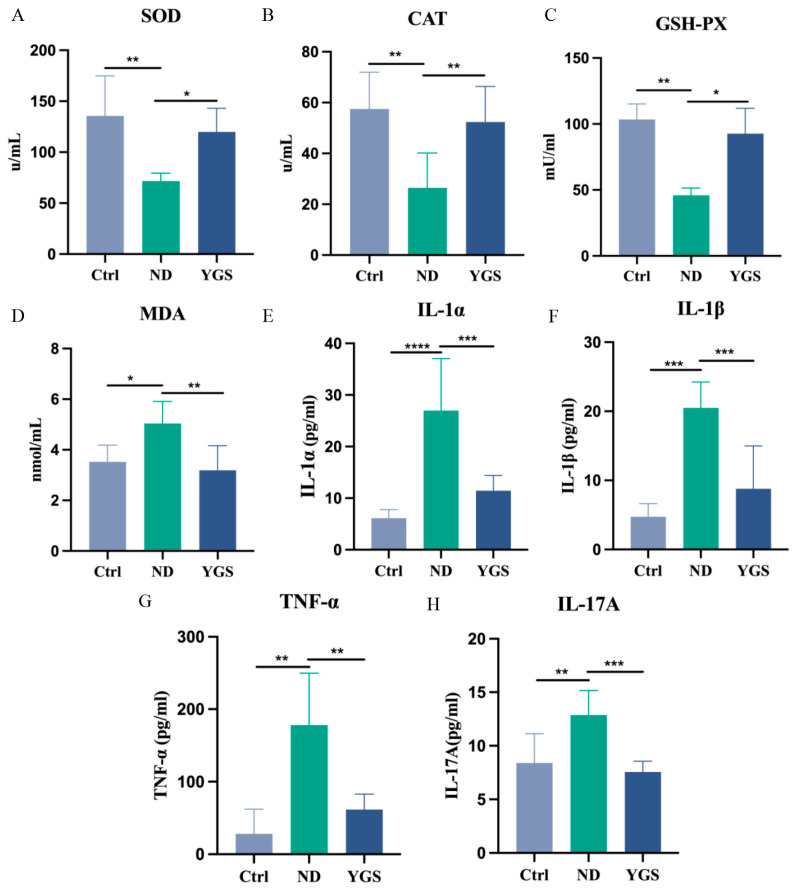
Effects of YGS treatment on serum oxidative stress indicators and pro-inflammatory cytokines in diarrheic calves. (**A**) Superoxide dismutase (SOD), (**B**) Catalase (CAT), (**C**) Glutathione Peroxidase (GSH-PX), (**D**) Malondialdehyde (MDA), (**E**) Interleukin-1α (IL-1α), (**F**) Interleukin-1β (IL-1β), (**G**) Tumor necrosis factor-α (TNF-α), (**H**) Interleukin-17A (IL-17A). Ctrl: healthy control group; ND: natural diarrhea group; YGS: YGS treatment group. The data is presented as mean ± SD (n = 7). * *p* < 0.05, ** *p* < 0.01, *** *p* < 0.001, **** *p* < 0.0001.

Serum levels of oxidative stress indicators in calves are closely associated with intestinal health [19]. Subsequently, we evaluated the effect of YGS treatment on serum oxidative stress indicators in diarrheic calves. The results revealed that serum levels of CAT, SOD and GSH-PX were significantly lower in diarrheic calves compared to healthy controls (*p* < 0.05), YGS treatment significantly restored the serum levels of these antioxidant enzymes (*p* < 0.05) (Figure 2A−C). Moreover, the administration of YGS significantly reduced the increase in serum MDA levels induced by calf diarrhea. (*p* < 0.05) (Figure 2D). This indicates that YGS administration significantly improves oxidative stress levels in calves with diarrhea. 

### 3.5. The Effects of YGS on the Cytokine Levels

**Figure 3:**
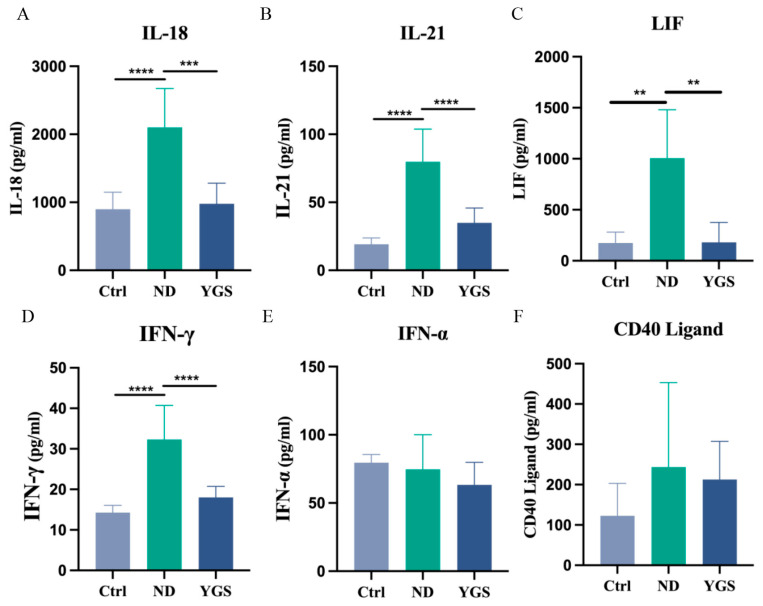
Effects of YGS treatment on serum pro-inflammatory cytokines in diarrheic calves. (**A**) Interleukin-18 (IL-18), (**B**) Interleukin-21 (IL-21), (**C**) Leukemia Inhibitory Factor (LIF), (**D**) Interferon-γ (IFN-γ), (**E**) Interferon-α (IFN-α), (**F**) CD40 Ligand. Ctrl: healthy control group; ND: natural diarrhea group; YGS: YGS treatment group. The data is presented as mean ± SD (n = 7). ** *p* < 0.01, *** *p* < 0.001, **** *p* < 0.0001.

**Figure 4:**
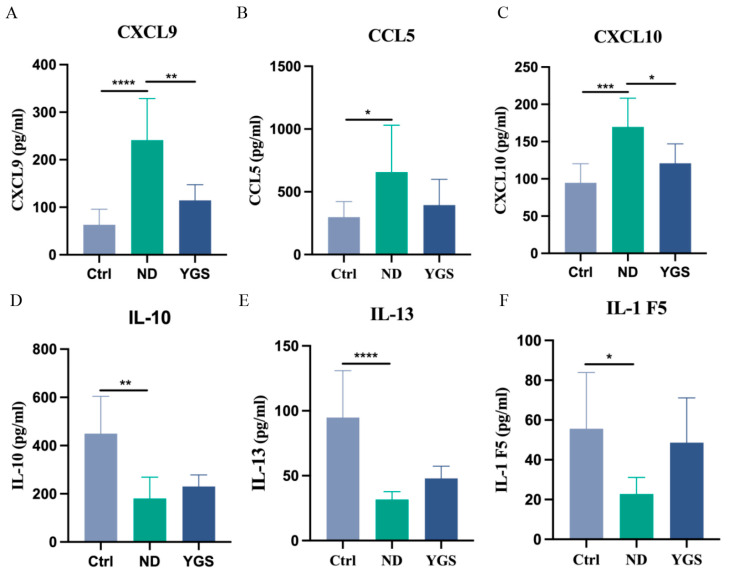
Effects of YGS treatment on serum chemokines and anti-inflammatory cytokines in diarrheic calves. (**A**) C-X-C Motif Chemokine Ligand 9 (CXCL9), (**B**) C-C Motif Chemokine Ligand 5 (CCL5), (**C**) C-X-C Motif Chemokine Ligand 10 (CXCL10), (**D**) Interleukin-10 (IL-10), (**E**) Interleukin-13 (IL-13), (**F**) Interleukin-1 Family Member 5 (IL-1F5). ND: natural diarrhea group; YGS: YGS treatment group. The data is presented as mean ± SD (n = 7). * *p* < 0.05, ** *p* < 0.01, *** *p* < 0.001, **** *p* < 0.0001.

To further investigate the effects of YGS on inflammatory responses in diarrheic calves, we comprehensively analysed serum inflammatory mediators. Compared to the Ctrl group, diarrheic calves exhibited significantly elevated levels of the pro-inflammatory cytokines IL-1α, TNF-α, IL-1β, IL-17A, IL-18, IL-21, LIF, and IFN-γ (*p* < 0.05), indicating a severe systemic inflammatory response. YGS treatment significantly reduced the levels of these cytokines (*p* < 0.05) (Figure 2E−H, Figure 3A−D). Levels of CD40 ligand and IFN-α were increased in diarrheic calves and decreased after YGS treatment, although these changes were non-significant (Figure 3E,F). Concurrently, the chemokines CXCL9, CXCL10, and CCL5 were significantly upregulated (*p* < 0.05) in diarrheic calves. Following YGS intervention, CXCL9 and CXCL10 levels decreased significantly (*p* < 0.05) compared to the ND group (Figure 4A−C), while CCL5 levels decreased but the change was non-significant. Notably, levels of the anti-inflammatory cytokines IL-10, IL-13, and IL-1F5 were significantly reduced (*p* < 0.05) in diarrheic calves. YGS administration increased their levels, but the results were non-significant (Figure 4D−F).

### 3.6. The Effects of YGS on Metabolomics

**Figure 5:**
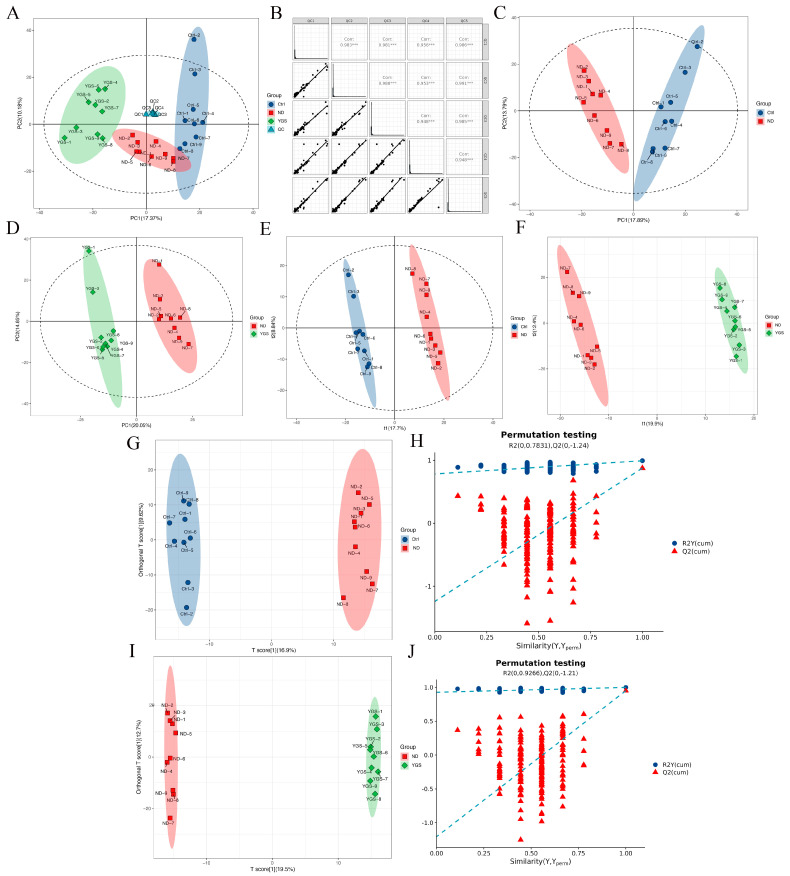
Evaluation of serum metabolomic profiling differences in diarrheic calves before and after YGS treatment. (**A**) Principal Component Analysis (PCA) of experimental samples (Ctrl, ND, YGS groups) and quality control (QC) samples. (**B**) Pearson correlation analysis of QC samples. (**C**) PCA score plot of the ND group vs. Ctrl group; (**D**) PCA score plot of the YGS group vs. ND group. (**E**) Partial Least Squares-Discriminant Analysis (PLS-DA) score plot of the ND group vs. the Ctrl group; (**F**) PLS-DA score plot of the YGS group vs. the ND group. (**G**,**H**) Orthogonal PLS-DA (OPLS-DA) score plot of the ND group vs. the Ctrl group; (**I**,**J**) OPLS-DA score plot of the YGS group vs. the ND group. *** *p* < 0.001.

To further investigate the changes in serum metabolites of diarrheic calves following YGS administration, we conducted serum metabolomic profiling. QC were employed to assess sample reproducibility and analytical system stability throughout the experimental process. Following normalization, principal component analysis (PCA) was performed on peaks extracted from all experimental and QC. QC clustered tightly, indicating excellent experimental reproducibility (Figure 5A). QC were further subjected to Pearson correlation analysis. Correlation coefficients all exceeding 0.9 confirmed good correlation (Figure 5B). Following rigorous quality assessment, the data were analyzed further. 

PCA, partial least squares-discriminant analysis (PLS-DA), and Orthogonal PLS-DA (OPLS-DA) were employed to assess inter-group metabolic differences. Pronounced clustering of diarrheic calves versus controls in score plots (Figure 5C−J) evidenced significant endogenous metabolite reorganization during calf diarrhea. 

Notably, significant metabolic changes were observed following YGS group compared to the ND group. The model parameters obtained through 200 permutation tests confirmed the high reliability and accuracy of the two comparative analyses. The comparison model between the ND group and the Ctrl group performed exceptionally well [Q^2^ = 0.876; R^2^X = 0.265; R^2^Y = 0.993; CV-ANOVA *p* = 0.005], while the comparison model between the YGS group and the ND group demonstrated strong reliability and predictive capability [Q^2^ = 0.95; R^2^X = 0.409; R^2^Y = 0.999; CV-ANOVA *p* = 0.005]. 

**Figure 6:**
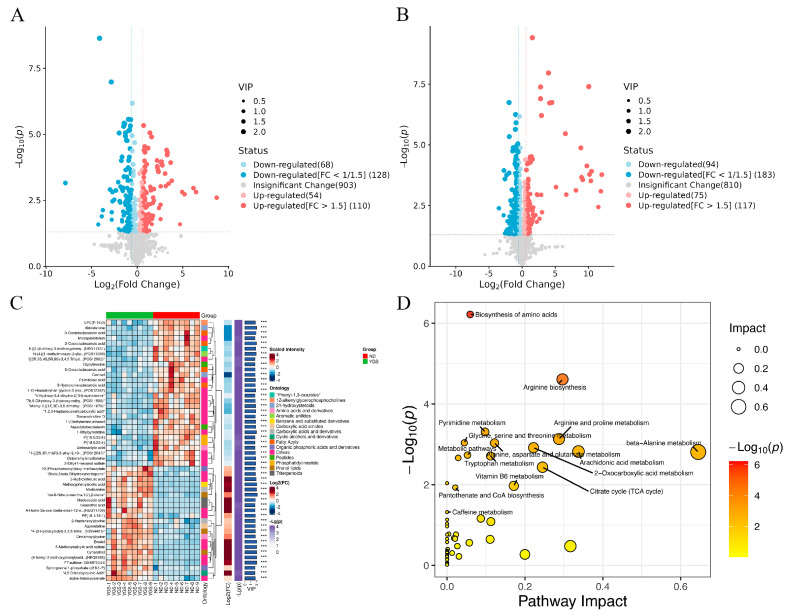
Effects of YGS on serum metabolites and metabolic pathways. (**A**) Volcano plots of serum differential metabolite analysis in the ND and Ctrl groups; (**B**) Volcano plots of serum differential metabolite analysis in YGS and ND groups; (**C**) Heatmap of the relative abundance of metabolites in the top 50 with significantly differences between the YGS and ND groups (VIP > 1.0, and *p* < 0.05 with 95 % confidence intervals); (**D**) The metabolic pathway impact prediction between the YGS and ND groups based on the KEGG. *** *p* < 0.001.

**Figure 7:**
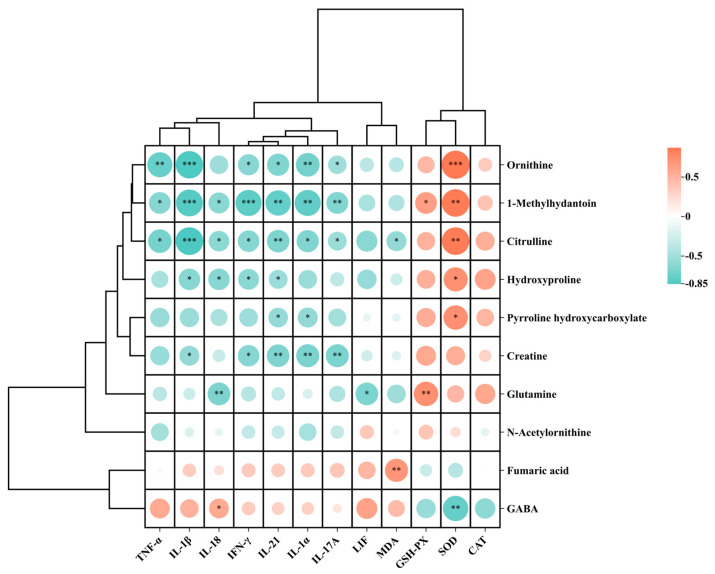
Correlation analysis between differential metabolites in Arginine and proline metabolism and Arginine biosynthesis pathways with serum oxidative stress indicators and cytokines. * *p* < 0.05, ** *p* < 0.01, *** *p* < 0.001.

Compared to the Ctrl group, 164 metabolites were significantly up-regulated and 196 were down-regulated in the ND group. Compared to the ND group, the YGS treatment group exhibited 192 upregulated metabolites and 277 downregulated metabolites (Figure 6A,B). Detailed information on differential metabolites was available in Supplementary Information S2. The top 50 significantly differential metabolites were identified as potential biomarkers distinguishing the ND and YGS groups and are displayed in the heatmap in Figure 6C. Most of the significantly altered metabolites could be categorized into lipids, steroids and derivatives, organic acids and derivatives, alkaloids and derivatives, and phenolic compounds, among others. To further investigate the metabolic pathways altered in diarrheic calves following YGS administration, enrichment analysis was performed on the differentially expressed metabolites. Compared to the ND group, YGS administration induced significant alterations in multiple metabolic pathways (Figure 6D), including Biosynthesis of amino acids, Arginine biosynthesis, Arginine and proline metabolism, Pyrimidine metabolism, and beta−Alanine metabolism. Furthermore, correlation analysis revealed that upregulated metabolites in the arginine biosynthesis and arginine-proline metabolism pathways were negatively correlated with pro-inflammatory cytokines (including IL-1α, TNF-α, IL-1β, IL-17A, IL-18, IL-21 and IFN-γ) and MDA, and positively correlated with antioxidant indicators (CAT, SOD and GSH-PX). Notably, the strongest correlations were observed for the metabolites 1-methylhydantoin, ornithine, and citrulline (Figure 7).

## 4. Discussion

Antimicrobial agents have long been the primary treatment for calf diarrhea. However, the long-term use of antibiotics contributes to antimicrobial resistance and residue concerns, driving the search for alternative therapies. This study investigated the therapeutic effects of YGS on diarrheic calves. Our findings demonstrate that YGS administration significantly ameliorated diarrhea, improved abnormal biochemical parameters, enhanced antioxidant and anti-inflammatory capacity, and alleviated metabolic disturbances in diarrheic calves.

YGS treatment effectively reduced fecal scores, dehydration scores, and mental status scores in diarrheic calves, indicating successful therapeutic intervention. Serum biochemical parameters serve as crucial indicators of animal health status. Furthermore, diarrheic calves exhibited elevated serum GLB levels. Given the close association between GLB and immune function [20], the normalization of GLB levels following YGS treatment suggests its efficacy in modulating the immune response in diarrheic calves.

Antioxidant capacity serves as a critical indicator of animal health status. It has been reported that diarrheic calves exhibit oxidative stress [21]. Consistent with this, the present study observed that the levels of SOD, CAT and GSH-PX were significantly decreased, while the level of MDA was significantly increased in diarrheic calves. Antioxidant capacity can be directly assessed by the activities of SOD, CAT and GSH-PX. SOD acts as the primary scavenger of oxygen free radicals, while CAT catalyzes the decomposition of hydrogen peroxide into water and oxygen, thereby mitigating cellular oxidative damage [22]. The observed decline likely reflects depletion of these antioxidant enzymes in the bloodstream, as the organism attempts to counteract excessive free radical production and restore redox homeostasis during oxidative stress [23]. MDA, a biomarker of oxidative stress, is produced through free radical-induced lipid peroxidation and serves as an indicator of biomembrane peroxidative damage [24]. Administration of YGS significantly elevated SOD, CAT, and GSH-PX levels, consequently enhancing antioxidant activity. This synergistic enhancement of the primary antioxidant enzyme cascade ensures the efficient and complete neutralization of reactive oxygen species (ROS). Consequently, the diminished substrate for lipid peroxidation reactions directly leads to the decreased production of MDA, a reliable end-product biomarker of such damage. This finding aligns with previous research demonstrating that the principal components of YGS possess notable antioxidant properties [25,26,27,28,29].

Beyond biochemical abnormalities and oxidative stress, diarrhea triggers inflammatory responses in calves. Cytokines critically orchestrate inflammatory responses and gut barrier homeostasis [30]. Previous studies have reported that serum levels of pro-inflammatory cytokines were significantly higher in calves with E. coli-induced diarrhea compared with those in healthy controls [31]. Our results also demonstrate that YGS significantly suppressed the expression of pro-inflammatory cytokines (IL-1α, IL-1β, TNF-α, IL-17A, IL-18, IL-21, and IFN-γ), effectively reducing inflammation and alleviating diarrhea. These cytokines are key mediators in immune and inflammatory responses. Consistent with our findings, YGS has been shown to inhibit macrophage inflammatory factors and modulate NF-κB and caspase-1 activation, contributing to its anti-inflammatory effects [13].

Chemokines, a family of >50 structurally conserved 8–10 kDa chemoattractant proteins, orchestrate leukocyte trafficking to direct inflammatory and immune responses through targeted recruitment [32]. The CC-chemokine CCL5 (RANTES) drives leukocyte migration, and its pathological elevation is implicated in chronic inflammation and associated disorders [33]. IFN-γ-inducible chemokines (CXCL9 and CXCL10) are crucial for immune cell maturation and migration, modulating inflammation by inducing, sustaining, and amplifying inflammatory/immune responses [34]. Our study confirmed significantly elevated levels of CXCL9, CXCL10, and CCL5 in diarrheic calves, further validating severe inflammation. YGS treatment effectively reduced CXCL9 and CXCL10 levels, suggesting its mechanism may involve modulation of the IFN signaling pathway. Concurrently, we detected downregulation of IL-10, IL-13, and IL-1F5 in diarrheic calves, indicating an imbalance between TH1 and TH2 cells and suppressed secretion of anti-inflammatory factors by TH2 cells, potentially compromising humoral immunity [35]. While YGS showed a tendency to restore these anti-inflammatory cytokine levels, the effect was not statistically significant, suggesting its primary anti-diarrheal action may be through suppressing pro-inflammatory mediators rather than potently elevating anti-inflammatory ones.

Liquid chromatography-mass spectrometry (LC-MS) enables the identification of specific metabolites and prediction of disease phenotype-associated metabolic pathways, facilitating the elucidation of mechanistic links through integrated analysis of the metabolome and host phenotype [36]. In this study, serum metabolomic profiling using UPLC-Q-Exactive Orbitrap-MS was performed to investigate the mechanism by which YGS alleviates calf diarrhea. The results revealed that YGS significantly reduced serum levels of Aldosterone and Aminoadipic acid, metabolites known to promote inflammation and oxidative stress. Aldosterone enhances the expression of inflammatory mediators and chemokine receptors, inducing inflammation and oxidative stress [37]. Aminoadipic acid increases serum inflammatory cytokines and hepatic malondialdehyde levels while altering SOD activity, promoting inflammation via the ROS/TXNIP/NLRP3 signaling pathway [38]. Importantly, metabolic pathway enrichment analysis of differential metabolites indicated that YGS alleviates diarrhea potentially by modulating pathways related to Biosynthesis of amino acids, Arginine biosynthesis, Arginine and proline metabolism, and beta−Alanine metabolism. Amino acid metabolism underpins not only protein synthesis but also controls immune cell function, regulates T cell fate, supports metabolic reprogramming (e.g., glycolysis, mitochondrial metabolism) [39]. Dysfunctional amino acid metabolism is a key driver of inflammatory bowel disease (IBD) progression. Amino acid repletion attenuates IBD-related inflammation and oxidative stress, whereas deficiency exacerbates colitic pathology [39]. Previous studies confirm that disturbances in Arginine and Proline Metabolism can trigger inflammatory responses [40]. Correlation analysis revealed statistically significant negative correlations between the levels of two metabolites in this pathway—1-Methylhydantoin and Ornithine—and the expression of inflammatory cytokines. Both compounds display significant anti-inflammatory properties with concurrent augmentation of antioxidant defenses [41,42]. Therefore, we propose that the anti-diarrheal effect of YGS may involve modulation of the Arginine and Proline Metabolism pathway, elevating levels of 1-Methylhydantoin and Ornithine, which merits further investigation. However, our study still suffers from the problem of small sample size, and the next step will be to further expand the number of test animals to further determine the efficacy of YGS.

## 5. Conclusions

YGS demonstrates significant therapeutic efficacy in the treatment of diarrhea calves. Administration of YGS effectively alleviated clinical symptoms, normalized serum biochemical parameters, enhanced antioxidant capacity by elevating levels of SOD, CAT, and GSH-PX while reducing MDA, and suppressed the expression of key pro-inflammatory cytokines and chemokines. Metabolomic analysis revealed that YGS treatment notably modulated metabolic disorders, particularly enriching the Arginine and Proline Metabolism pathway. Key metabolites such as 1-methylhydantoin and ornithine were significantly correlated with reduced inflammation and improved antioxidant status. These results suggest that YGS ameliorates calf diarrhea through mechanisms involving antioxidant, anti-inflammatory, and metabolic regulatory actions. This study supports the potential of YGS as a safe and effective antibiotic alternative for managing calf diarrhea, contributing to sustainable dairy farming practices. Further research with larger sample sizes is warranted to validate these findings and explore underlying molecular mechanisms.

## Figures and Tables

**Table 1:** The composition of YGS.

Chinese Name	Latin Name	Batch Number	Ratio
Dang Shen	*Codonopsis pilosula* (Franch.) Nannf.	220925002	1
Bai Zhu	*Atractylodes macrocephala* Koidz.	220911004	1
Fu Ling	*Poria cocos* (Schw.) Wolf	221221002	1
Chen Pi	*Citrus reticulata* Blanco	230615001	1
Gan Cao	*Glycyrrhiza uralensis* Fisch. ex DC.	230627001	1

**Table 2:** Effects of YGS treatment on clinical symptom scores of diarrheic calves (Day 0 to Day 7).

Items	Treatment		Group		*p*-Value
		Ctrl	ND	YGS	
Fecal scores					
	D0	0.10 ± 0.32 ^b^	3.70 ± 0.48 ^a^	3.50 ± 0.53 ^a^	<0.001
	D1	0.00 ± 0.00 ^b^	3.60 ± 0.52 ^a^	3.50 ± 0.53 ^a^	<0.001
	D2	0.10 ± 0.32 ^b^	3.60 ± 0.52 ^a^	3.20 ± 0.42 ^a^	<0.001
	D3	0.00 ± 0.00 ^b^	3.00 ± 0.00 ^a^	2.70 ± 0.48 ^a^	<0.001
	D4	0.20 ± 0.42 ^b^	3.00 ± 0.82 ^a^	2.20 ± 0.63 ^a^	<0.001
	D5	0.00 ± 0.00 ^b^	2.60 ± 0.52 ^a^	1.50 ± 0.53 ^a^	<0.001
	D6	0.10 ± 0.32 ^b^	2.50 ± 0.53 ^a^	0.20 ± 0.42 ^b^	<0.001
	D7	0.10 ± 0.32 ^b^	2.60 ± 0.52 ^a^	0.30 ± 0.48 ^b^	<0.001
Dehydration scores					
	D0	0.00 ± 0.00 ^b^	3.60 ± 0.52 ^a^	3.60 ± 0.52 ^a^	<0.001
	D1	0.00 ± 0.00 ^b^	3.40 ± 0.52 ^a^	3.00 ± 0.47 ^a^	<0.001
	D2	0.00 ± 0.00 ^b^	3.40 ± 0.52 ^a^	2.70 ± 0.48 ^a^	<0.001
	D3	0.00 ± 0.00 ^c^	3.50 ± 0.53 ^a^	2.20 ± 0.63 ^b^	<0.001
	D4	0.00 ± 0.00 ^c^	2.80 ± 0.42 ^a^	1.60 ± 0.52 ^b^	<0.001
	D5	0.00 ± 0.00 ^b^	2.40 ± 0.52 ^a^	0.90 ± 0.57 ^b^	<0.001
	D6	0.00 ± 0.00 ^b^	2.60 ± 0.52 ^a^	0.20 ± 0.42 ^b^	<0.001
	D7	0.00 ± 0.00 ^b^	2.70 ± 0.67 ^a^	0.00 ± 0.00 ^b^	<0.001
Mental scores					
	D0	0.00 ± 0.00 ^b^	3.50 ± 0.53 ^a^	3.60 ± 0.52 ^a^	<0.001
	D1	0.30 ± 0.48 ^b^	3.50 ± 0.53 ^a^	2.90 ± 0.32 ^a^	<0.001
	D2	0.10 ± 0.32 ^b^	3.70 ± 0.48 ^a^	2.60 ± 0.52 ^a^	<0.001
	D3	0.30 ± 0.48 ^b^	3.00 ± 0.00 ^a^	2.00 ± 0.47 ^a^	<0.001
	D4	0.10 ± 0.32 ^b^	3.00 ± 0.00 ^a^	1.60 ± 0.52 ^a^	<0.001
	D5	0.00 ± 0.00 ^b^	2.40 ± 0.52 ^a^	0.70 ± 0.48 ^b^	<0.001
	D6	0.00 ± 0.00 ^b^	2.40 ± 0.52 ^a^	0.20 ± 0.42 ^b^	<0.001
	D7	0.10 ± 0.32 ^b^	2.50 ± 0.53 ^a^	0.00 ± 0.00 ^b^	<0.001

^a–c^ Values in a row with no common letters differ significantly (*p* < 0.05) with mean ± SD. Ctrl: healthy control group; ND: natural diarrhea group; YGS: YGS treatment group.

**Table 3:** Effects of YGS treatment on serum biochemical indicators of diarrheic calves.

Items	Group			*p*-Value
	Ctrl	ND	YGS	
ALT (U/L)	11.16 ± 3.17 ^a^	6.30 ± 0.55 ^b^	8.49 ± 1.58 ^a^	<0.001
AST (U/L)	46.34 ± 6.65 ^a^	50.52 ± 6.46 ^a^	47.18 ± 5.62 ^a^	0.341
TP (g/dL)	5.40 ± 0.56 ^b^	6.65 ± 0.61 ^a^	5.21 ± 0.65 ^b^	<0.001
GLB (g/dL)	2.54 ± 0.50 ^b^	3.97 ± 0.48 ^a^	2.34 ± 0.57 ^b^	<0.001
ALP (U/L)	446.43 ± 187.82 ^a^	230.13 ± 65.38 ^b^	512.96 ± 198.97 ^a^	0.001
LDH (U/L)	779.71 ± 76.95 ^a^	744.27 ± 90.07 ^a^	768.66 ± 64.00 ^a^	0.381
CK (U/L)	75.70 ± 19.62 ^c^	188.13 ± 47.30 ^a^	127.27 ± 36.79 ^b^	<0.001
TBA (μmol/L)	19.43 ± 6.39 ^a^	17.76 ± 9.05 ^a^	18.78 ± 3.66 ^a^	0.869
UREA (mg/dL)	10.44 ± 2.12 ^b^	19.16 ± 5.29 ^a^	13.64 ± 1.95 ^b^	<0.001
UA (mg/dL)	1.06 ± 0.21 ^a^	1.03 ± 0.19 ^a^	0.89 ± 0.10 ^a^	0.129
CHOL (mg/dL)	83.09 ± 4.31 ^b^	42.26 ± 12.02 ^c^	105.29 ± 19.47 ^a^	<0.001
AMY (U/L)	21.20 ± 6.74 ^b^	38.06 ± 3.69 ^a^	24.50 ± 5.01 ^b^	<0.001
P (mmol/L)	10.86 ± 1.30 ^a^	10.78 ± 1.09 ^a^	11.33 ± 1.15 ^a^	0.573
CL (mmol/L)	94.93 ± 2.01 ^a^	97.76 ± 3.81 ^a^	96.90 ± 1.47 ^a^	0.085
K (mmol/L)	6.22 ± 0.80 ^a^	6.20 ± 0.34 ^a^	6.39 ± 0.52 ^a^	0.744
Fe (μg/dL)	140.98 ± 73.35 ^a^	157.00 ± 110.06 ^a^	172.13 ± 100.02 ^a^	0.862
Ca (mg/dL)	11.30 ± 0.77 ^a^	10.01 ± 0.27 ^b^	10.82 ± 0.31 ^a^	0.003
Mg (mmol/L)	1.05 ± 0.10 ^a^	0.84 ± 0.05 ^b^	0.97 ± 0.03 ^b^	0.009

^a–c^ Values in a row with no common letters differ significantly (*p* < 0.05) with mean ± SD. alanine aminotransferase (ALT), aspartate aminotransferase (AST), total protein (TP), globulin (GLB), alkaline phosphatase (ALP), lactate dehydrogenase (LDH), creatine kinase (CK), total bile acids (TBA), UREA, uric acid (UA), total cholesterol (CHOL), amylase (AMY), calcium (Ca), phosphorus (P), iron (Fe), magnesium (Mg), potassium (K) and chloride (Cl). Ctrl: healthy control group; ND: natural diarrhea group; YGS: YGS treatment group.

## Data Availability

The original contributions presented in this study are included in the article/supplementary material. Further inquiries can be directed to the corresponding authors.

## Supplementary Materials

The following supporting information can be downloaded at https://www.mdpi.com/article/10.3390/metabo15090618/s1, Table S1. The scoring criteria of clinical examination [1]; Table S2. Biologically active components in YGS extract identified by UPLC-MS/MS. Table S3. Daily clinical symptom trajectories of individual calves in the Ctrl group, ND group and YGS group. Figure S1: Kaplan–Meier survival curves for clinical remission of diarrhea in calves from the YGS group and ND group. Statistical comparison between the two groups was performed using the log-rank test (χ^2^ = 18.81, *p* < 0.0001), indicating an extremely significant difference in remission dynamics between the YGS group and ND group; Supplementary Information S1: Supplementary experimental methods and results; Supplementary Information S2: Differences in metabolites between the ND group and the Ctrl group; Supplementary Information S3: Inspection certificates for component herbs of YGS.

## Abbreviations

The following abbreviations are used in this manuscript:

ALP	alkaline phosphatase
ALT	alanine aminotransferase
AMY	amylase
AST	aspartate aminotransferase
Ca	calcium
CAT	catalase
CHOL	total cholesterol
CK	creatine kinase
Cl	chloride
ELISA	enzyme-linked immunosorbent assays
Fe	iron
GLB	globulin
GSH-PX	glutathione peroxidase
IBD	inflammatory bowel disease
K	potassium
KEGG	Kyoto Encyclopedia of Genes and Genomes
LDH	lactate dehydrogenase
MDA	malondialdehyde
Mg	magnesium
NAHMS	National Animal Health Monitoring System
OPLS-DA	orthogonal partial least squares-discriminant analysis
P	phosphorus
PCA	principal component analysis
PLS-DA	partial least squares-discriminant analysis
QC	quality control
SD	standard deviation
SOD	superoxide dismutase
TBA	total bile acids
TCM	Traditional Chinese medicines
TP	total protein
UA	uric acid
VIP	Variable Importance in Projection
YGS	Yigong San
