# Supplementary material for: Yigong San Extract Modulates Metabolism, Antioxidant Status, and Immune Function to Improve Health in Diarrheic Calves
Source: Metabolites. 2025 Sep 18;15(9):618. doi: 10.3390/metabo15090618 (PMC12471574; doi:10.3390/metabo15090618)
Supplement: Supplementary file 1 [file metabolites-15-00618-s001.zip › Supplementary Information1.pdf]

1. Methods

1.1 Detection methods in main chemical components analysis for YGS by UPLC-MS/MS

Sample Preparation:

Samples were dissolved in 1 mL of 80% methanol, followed by sonication for 10 min and centrifugation at 14,000 rpm for 10 min. Subsequently, 500 μL of the supernatant was filtered through a 0.22 μm membrane prior to UPLC-MS/MS analysis.

Chromatographic Conditions:

Separation was achieved using an ACQUITY UPLC HSS T3 column (2.1 × 100 mm, 1.8 μm; Waters, USA) at a constant flow rate of 0.3 mL/min. The mobile phase consisted of (A) 0.1% formic acid in water and (B) 0.1% formic acid in acetonitrile. The gradient elution program was as follows:

Time (min)	A（%）	B（%）
0	100	0
10	70	30
25	60	40
30	50	50
40	30	70
45	0	100
60	0	100
60.5	100	0
70	100	0

Mass Spectrometric Analysis

MS and MS/MS data were acquired in Information-Dependent Acquisition (IDA) mode using a Q Exactive Focus mass spectrometer (Thermo Fisher Scientific, USA) controlled by Xcalibur software (vX.X, Thermo Fisher Scientific). Full scan MS (m/z 100–1200) with data-dependent MS^2 (ddMS2) was performed in positive/negative polarity switching mode, with key parameters set as follows: capillary temperature 320°C, auxiliary gas heater temperature 350°C, sheath gas flow 40 L/min, auxiliary gas flow 15 L/min, spray voltage ±3.2 kV, full MS resolution 70,000, MS/MS resolution 17,500, and stepped normalized collision energies (NCE) at 30, 40, and 50 eV.

Data Processing:

Raw data were processed using Compound Discoverer 3.3 software (Thermo Fisher Scientific). Metabolite identification was performed by matching MS/MS spectra against the Tsinghua University Secondary Mass Spectrometry Database and the mzCloud online database (Thermo Fisher Scientific).

1.2 Randomisation Sequence Generation

First, 20 diarrheic calves meeting the inclusion criteria (5–7 days old, 45–55 kg, fecal score > 2) were numbered sequentially from 1 to 20 based on their admission order. Next, 20 random numbers between 0 and 1 were generated using the "random number generator" function in Microsoft Excel, and these numbers were sorted in ascending order. Calves corresponding to the first 10 sorted numbers were assigned to the ND group, and those corresponding to the last 10 sorted numbers were assigned to the YGS group. Prior to formal intervention, the veterinarian confirmed calf groupings on-site to ensure that researchers responsible for feeding could not learn the groupings in advance.

1.2 The scoring criteria of clinical examination

Table S1. The scoring criteria of clinical examination[1]

Items	Scoring Basis	Scores
Fecal status	Feces with normal shape and consistency	0
	Semi-formed or pasty feces	1
	Feces are not formed but can adhere to ground or bedding	2
	Watery feces	3
Mental status	Normal, respond quickly to external stimuli and vigorously	0
	Mild depressive retains sucking reflex without vitality	1

	Moderate depressive, stand after stimulation with a weak or irregular sucking reflex	2
	Severe depressive, unable to stand or without sucking reflex	3
Dehydration status	The hydration state is normal, tent test time of upper eyelid skin < 2 s	0
	Eyeball slightly sunken, tent test time of upper eyelid skin is > 2 s but < 4 s	1
	Sunken eyeball, dry nose, tent test time of upper eyelid skin > 5 s	2
	Severe sunken eyeball, cold ears, limbs and mouth, dry nose	3

1.3 Blinding for Outcome Assessment

Clinical scoring:

The observers responsible for recording fecal status, mental status, and dehydration scores were trained uniformly on the scoring criteria (Table 2) but were not informed of the grouping information (i.e., which calves belonged to Ctrl/ND/YGS). During the 7-day observation period, observers only recorded data using calf serial numbers, and the correspondence between serial numbers and groups was decoded only after the entire scoring process was completed.

Biochemical assays and oxidative stress/cytokine detection:

Serum samples were labeled with unique codes (e.g., S-Ctrl-01, S-ND-01, S-YGS-01) before detection, and the detection personnel operating the Roche Cobas 6000 c501 analyzer (biochemical indices) and test kits were unaware of the code-group correspondence. The grouping information was only matched to the detection data after all assays were finished.

Serum metabolomic analysis:

As mentioned, this part was commissioned by Shanghai Bioprofile Co., Ltd. To ensure blinding, only unique sample codes and basic group labels (Ctrl/ND/YGS) were provided to the service provider, and no additional experimental details were disclosed (e.g., the therapeutic effect of YGS, the pathological state of diarrheic calves, or the research hypothesis).

1.4 **Steps for dose conversion**

Animal Dose (g/day) = Human Dose (g/day) × (Animal BSA / Human BSA)

Assuming a standard human with a body weight of 70 kg and a BSA of approximately 1.73 m^2, and a 50 kg calf with a BSA of approximately 1.055 m^2 (based on standard veterinary zootechnical data), the calf dose was derived as follows:

Calf Dose = 75 g/day × (1.055 m^2 / 1.73 m^2) ≈ 75 × 0.61 ≈ 45.75 g/day

Normalized to the calf's body weight (50 kg), this gives:

45.75 g/day / 50 kg = 0.915 g/kg/day

1.5 Supportive care protocols

Fluid and electrolyte support:

All calves (including ND and YGS groups) had ad libitum access to fresh water and oral electrolyte solutions (containing glucose, sodium chloride, potassium chloride, and sodium bicarbonate, formulated to maintain electrolyte balance in diarrheic calves) throughout the experimental period. No calf was withheld from standard rehydration care.

Nutritional support:

All calves were fed colostrum (within 24 h of birth) and milk replacer (3 times daily, 10% of body weight per feeding) according to age-matched standard feeding protocols, ensuring basic nutritional needs were met.

Environmental and nursing care:

Calves were housed in individual, well-ventilated pens with clean bedding (changed daily) to maintain a dry, warm environment (ambient temperature 20–22 °C), reducing stress and secondary infection risk.

1.6 Rescue protocols

If any calf (in ND or YGS group) showed severe deterioration (e.g., dehydration score ≥3 for 2 consecutive days, inability to stand or suck, rectal temperature <38 °C or >40.5 °C), emergency intervention (including intravenous fluid infusion and broad-spectrum antibiotic therapy, per veterinary clinical standards) would be initiated immediately. In this study, no calf required such rescue measures, as YGS treatment or standard supportive care effectively alleviated symptoms.

1.7 Metabolomics analysis of serum samples

Serum metabolomic profiling was performed using ultra-performance liquid chromatography coupled with electrospray ionization and quadrupole-Orbitrap mass spectrometry (UPLC-ESI-Q-Orbitrap MS). The analytical platform comprised a Shimadzu Nexera X2 LC-30AD UHPLC system (Kyoto, Japan) interfaced with a Q-Exactive Plus mass spectrometer (Thermo Scientific, USA). Chromatographic separation was achieved on an ACQUITY UPLC® HSS T3 column (2.1 × 100 mm, 1.8 μm; Waters, USA) main-tained at 40 °C, using a flow rate of 0.3 mL/min. The mobile phase included (A) water with 0.1% formic acid and (B) acetonitrile. The gradient program was as follows: 0–2 min, 0% B; 2–6 min, 0–48% B; 6–10 min, 48–100% B; 10–12 min, 100% B; 12–12.1 min, 100–0% B; followed by 3 min re-equilibration. Mass spectrometry detection utilized a heated elec-

trospray ionization (HESI) source operating in both positive and negative modes. Key settings included spray voltage: ±3.8 kV (positive) / –3.2 kV (negative); capillary and probe temperatures: 320 °C and 350 °C, respectively; sheath and auxiliary gas flows: 30 and 5 arbitrary units; S-lens RF level: 50. Full MS scans spanned m/z 70–1050 at a res-olution of 70,000. Data-dependent MS^2 acquisition was performed at a resolution of 17,500 using an isolation window of 2 m/z and normalized collision energies stepped at 20, 30, and 40 eV. Maximum injection times were set to 100 ms for full MS and 50 ms for MS^2.

2.8 Data processing and statistical analysis

Raw mass spectrometry data were processed using MS-DIAL to detect chromato-graphic peaks, align multiple samples, calibrate retention times, and integrate peak areas for quantification. Metabolites were tentatively identified by matching both accurate precursor ion masses (mass error < 5 ppm) and corresponding MS/MS fragmentation patterns against public databases (HMDB, MassBank) and a custom in-house library of authentic standards. Initial data filtering retained only those ion features present in over 50% of samples within at least one experimental group. Statistical analyses were performed using R (v4.0.3). Multivariate pattern recognition was carried out through principal component analysis (PCA), partial least squares-discriminant analysis (PLS-DA), and orthogonal PLS-DA (OPLS-DA). Metabolites with Variable Importance in Projection (VIP) scores greater than 1.0 in OPLS-DA models were considered influential for group separation. Statistically significant discriminatory metabolites were defined as those with VIP > 1.0 and univariate p-values < 0.05. Hier-archical clustering of these metabolites was conducted using relevant R packages. Pathway enrichment analysis was performed based on the Kyoto Encyclopedia of Genes and Genomes (KEGG) database.

2. Results

Table S2. Biologically active components in YGS extract identified by UPLC-MS/MS.

Source	Name	Formula	Annot. DeltaMass [ppm]	Calc. MW	m/z	RT [min]	Reference Ion
Codonopsis pilosula (Franch.) Nannf.	DL-Stachydrine	C7 H13 N O2	1.12	143.0948	144.1021	1.573	[M+H]+1
	(+)-Magnoflorine	C20 H23 N O4	0.08	341.1627	342.17	21.039	[M+H]+1
	Lobetyolin	C20 H28 O8	-0.74	396.1781	441.1763	23.079	[M+FA-H]-1
	4-Oxoproline	C5 H7 N O3	-1.24	129.0424	128.0352	3.37	[M-H]-1
	Trigonelline	C7 H7 N O2	1.39	137.0479	138.0552	1.543	[M+H]+1
	Schaftoside	C26 H28 O14	0.34	564.1481	563.1404	20.335	[M-H]-1
	Rutin	C27 H30 O16	-0.28	610.1532	609.1456	21.313	[M-H]-1
	Adenosine 3'5'-cyclic monophosphate	C10 H12 N5 O6 P	-0.88	329.0522	328.045	12.133	[M-H]-1
	Cryptochlorogenic acid	C16 H18 O9	-0.54	354.0949	353.0876	18.914	[M-H]-1
	Calycosin-7-O-β-D-glucoside	C22 H22 O10	0.2	446.1214	447.1287	21.333	[M+H]+1
	Medicarpin	C16 H14 O4	0.38	270.0893	271.0966	25.35	[M+H]+1
	Biochanin A	C16 H12 O5	0.52	284.0686	285.0759	23.377	[M+H]+1
	Apigenin	C15 H10 O5	0.41	270.0529	271.0602	19.995	[M+H]+1
	Soyasaponin I	C48 H78 O18	-0.13	942.5187	941.5107	31.12	[M-H]-1
	Kaempferol	C15 H10 O6	-0.53	286.0476	285.0402	24.98	[M-H]-1
	Indole-3-lactic acid	C11 H11 N O3	1.08	205.0741	206.0814	21.285	[M+H]+1
	4,5-Dicaffeoylquinic acid	C25 H24 O12	-0.93	516.1263	515.119	22.961	[M-H]-1
	Hyperoside	C21 H20 O12	0.26	464.0956	463.0883	21.734	[M-H]-1
	Glycitein	C16 H12 O5	-0.23	284.0684	285.0758	32.749	[M+H]+1

	Apigenin-7-O-β-D-glucoside	C21 H20 O10	-0.08	432.1056	433.1132	22.749	[M+H]+1
	Pantothenic acid	C9 H17 N O5	-0.11	219.1107	218.1032	16.389	[M-H]-1
	DL-Tryptophan	C11 H12 N2 O2	-0.77	204.0897	203.0824	17.529	[M-H]-1
	1-Methyladenosine	C11 H15 N5 O4	1.01	281.1127	282.12	16.348	[M+H]+1
	Calycosin	C16 H12 O5	0.11	284.0685	285.0759	25.344	[M+H]+1
	Glycitin	C22 H22 O10	-0.12	446.1212	447.1288	23.382	[M+H]+1
	L-Phenylalanine	C9 H11 N O2	0.94	165.0791	166.0864	11.804	[M+H]+1
	trans-Aconitic acid	C6 H6 O6	-2.05	174.0161	173.0088	1.644	[M-H]-1
	Daidzin	C21 H20 O9	-0.12	416.1107	417.1181	21.131	[M+H]+1
	2,3,4,9-Tetrahydro-1H-β-carboline-3-carboxylic acid	C12 H12 N2 O2	0.39	216.09	217.0973	19.162	[M+H]+1
	Daidzein	C15 H10 O4	0.06	254.0579	255.0653	24.432	[M+H]+1
	Genistein	C15 H10 O5	-0.51	270.0527	269.0452	27.198	[M-H]-1
	Quercetin	C15 H10 O7	-0.31	302.0426	303.0498	21.317	[M+H]+1
Atractylodes macrocephala Koidz.	Atractylenolide III	C15 H20 O3	0.34	248.1413	249.1486	33.103	[M+H]+1
	Atractylenolide II	C15 H20 O2	0.41	232.1464	233.1537	37.287	[M+H]+1
	Dehydrocostus lactone	C15 H18 O2	0.46	230.1308	231.1381	33.106	[M+H]+1
	Ferulaldehyde	C10 H10 O3	1.01	178.0632	179.0705	31.756	[M+H]+1
	7-Methoxycoumarin	C10 H8 O3	0.47	176.0474	177.0547	18.649	[M+H]+1
	Salicylic acid	C7 H6 O3	-1.24	138.0315	137.0243	18.091	[M-H]-1
	Scopoletin	C10 H8 O4	1.48	192.0425	193.0498	21.752	[M+H]+1
	Hexadecanamide	C16 H33 N O	0.11	255.2562	256.2635	46.815	[M+H]+1

	3-Methoxybenzaldehyde	C8 H8 O2	0.46	136.0525	137.0598	31.756	[M+H]+1
	Scoparone	C11 H10 O4	0.93	206.0581	207.0654	23.9	[M+H]+1
	Dihydrocoumarin	C9 H8 O2	-1.35	148.0522	147.045	18.129	[M-H]-1
Poria cocos (Schw.) Wolf	D-Raffinose	C18 H32 O16	-0.39	504.1688	549.1671	2.396	[M+FA-H]-1
	α,α-Trehalose	C12 H22 O11	-1.11	342.1158	341.1085	1.564	[M-H]-1
	Uridine	C9 H12 N2 O6	-0.96	244.0693	243.062	4.969	[M-H]-1
	Poricoic acid A	C31 H46 O5	0.05	498.3346	497.3273	40.813	[M-H]-1
	Oleamide	C18 H35 N O	-0.1	281.2718	282.2791	46.912	[M+H]+1
	D-Glucosamine	C6 H13 N O5	0.97	179.0796	180.0868	1.452	[M+H]+1
Citrus reticulata Blanco	Hesperidin	C28 H34 O15	-0.61	610.1894	609.182	22.533	[M-H]-1
	Nobiletin	C21 H22 O8	0.58	402.1317	403.139	31.93	[M+H]+1
	Narirutin	C27 H32 O14	-0.71	580.1788	579.1713	22.003	[M-H]-1
	Tangeritin	C20 H20 O7	0.56	372.1211	373.1284	33.762	[M+H]+1
	Hesperetin	C16 H14 O6	-0.77	302.0788	303.0863	22.533	[M+H]+1
	Isosinensetin	C20 H20 O7	0.47	372.1211	373.1284	28.893	[M+H]+1
	6-Demethoxytangeretin	C19 H18 O6	0.62	342.1106	343.1178	30.531	[M+H]+1
	Sinensetin	C20 H20 O7	0.56	372.1211	373.1284	30.348	[M+H]+1
	Naringenin	C15 H12 O5	-0.51	272.0683	273.0757	22.003	[M+H]+1
	Didymin	C28 H34 O14	-0.18	594.1948	639.1929	24.666	[M+FA-H]-1
	Synephrine	C9 H13 N O2	0.87	167.0948	168.1021	3.392	[M+H]+1
	Isosakuranetin	C16 H14 O5	-0.43	286.084	287.0913	24.64	[M+H]+1

	Eriocitrin	C27 H32 O15	-0.25	596.174	595.1666	21.053	[M-H]-1
	Prunin	C21 H22 O10	0.13	434.1214	435.1286	22.003	[M+H]+1
	Limonin	C26 H30 O8	0.54	470.1943	471.2016	21.222	[M+H]+1
	5-O-Demethylnobiletin	C20 H20 O8	0.04	388.1158	389.1231	29.076	[M+H]+1
	Tangeretin	C20 H20 O7	0.5	372.1211	373.1284	31.643	[M+H]+1
	3',5,7-Trihydroxy-4'-methoxyflavanone	C16 H14 O6	-0.04	302.079	303.0863	20.615	[M+H]+1
	Eriodictyol	C15 H12 O6	-0.16	288.0633	289.0706	21.054	[M+H]+1
	Neodiosmin	C28 H32 O15	0.3	608.1743	607.1668	22.422	[M-H]-1
	Methyl cinnamate	C10 H10 O2	0.61	162.0682	163.0755	30.798	[M+H]+1
	Rhoifolin	C27 H30 O14	-0.06	578.1635	623.1615	22.216	[M+FA-H]-1
	Isosakuranin	C22 H24 O10	-0.13	448.1369	449.1442	24.671	[M+H]+1
	trans-Cinnamaldehyde	C9 H8 O	0.44	132.0576	133.0649	18.687	[M+H]+1
Glycyrrhiza uralensis Fisch. ex DC.	Glycyrrhizic acid	C42 H62 O16	-0.59	822.4033	821.3959	29.756	[M-H]-1
	Isoliquiritigenin	C15 H12 O4	-0.11	256.0735	257.0808	21.513	[M+H]+1
	Liguiritigenin-7-O-β-D-apiosyl-4'-O-β-D-glucoside	C26 H30 O13	-0.88	550.1682	549.1609	21.363	[M-H]-1
	Liquiritin	C21 H22 O9	-0.72	418.1261	417.1188	21.534	[M-H]-1
	Diammonium glycyrrhizinate	C42 H62 O16	-0.68	822.4032	821.3959	30.706	[M-H]-1
	Isoliquiritin	C21 H22 O9	-0.38	418.1262	417.1188	23.807	[M-H]-1
	Glabrolide	C30 H44 O4	0.33	468.3241	469.3314	27.118	[M+H]+1
	(15Z)-9,12,13-Trihydroxy-15-octadecenoic acid	C18 H34 O5	-0.86	330.2403	329.2331	28.128	[M-H]-1
	18 β-Glycyrrhetintic Acid	C30 H46 O4	0.47	470.3398	471.3471	29.763	[M+H]+1

Liquiritigenin	C15 H12 O4	-0.43	256.0735	255.0661	24.91	[M-H]-1
18-β-Glycyrrhetinic acid	C30 H46 O4	0.47	470.3398	471.3471	30.706	[M+H]+1
Licuroside	C26 H30 O13	0.86	550.1691	551.1764	23.555	[M+H]+1
Butein	C15 H12 O5	-0.36	272.0684	273.0757	21.418	[M+H]+1
(+/-)9,10-dihydroxy-12Z-octadecenoic acid	C18 H34 O4	-0.01	314.2457	313.2384	35.503	[M-H]-1
Glabrone	C20 H16 O5	-0.4	336.0996	337.107	38.279	[M+H]+1
Licochalcone A	C21 H22 O4	0.35	338.1519	339.1594	34.989	[M+H]+1
Licoflavone A	C20 H18 O4	0.34	322.1206	323.128	32.084	[M+H]+1
Kanzonol C	C25 H28 O4	-0.03	392.1988	393.2061	38.441	[M+H]+1
4,2',4'-Trihydroxy-3-methoxydihydrochalcone	C16 H16 O5	0.34	288.0999	289.1072	31.236	[M+H]+1
Lusitanicoside	C21 H30 O10	-1.21	442.1834	441.1761	21.131	[M-H]-1
3-[2-(β-D-Glucopyranosyloxy)-4-methoxyphenyl]propanoic acid	C16 H22 O9	-0.5	358.1262	357.1189	18.744	[M-H]-1
Retrochalcone	C16 H14 O4	-0.56	270.0891	271.0964	27.105	[M+H]+1
Vanillin	C8 H8 O3	0.84	152.0475	153.0548	20.85	[M+H]+1
4-Acetyl-3-hydroxy-5-methylphenyl β-D-glucopyranoside	C15 H20 O8	-0.81	328.1156	327.1083	19.637	[M-H]-1
Benzoic acid	C7 H6 O2	-0.84	122.0367	123.044	33.222	[M+H]+1
Kaempferol 3-O-rutinoside	C27 H30 O15	0.21	594.1586	595.1661	21.383	[M+H]+1
Isorhamnetin-3-O-rutinoside	C28 H32 O16	0.32	624.1692	625.1765	22.528	[M+H]+1
5-O-Methylgenistein	C16 H12 O5	0.3	284.0686	285.0758	34.335	[M+H]+1

	Icaritin	C21 H20 O6	-0.22	368.1259	369.1333	37.375	[M+H]+1
	Citric acid	C6 H8 O7	-0.77	192.0269	191.0196	1.654	[M-H]-1
	L-(-)-Malic acid	C4 H6 O5	-1.56	134.0213	133.014	1.571	[M-H]-1
	D-(-)-Quinic acid	C7 H12 O6	-1.22	192.0632	191.0559	1.511	[M-H]-1
	Ononin	C22 H22 O9	0.13	430.1264	431.134	23.745	[M+H]+1
	2-Isopropylmalic acid	C7 H12 O5	-1.78	176.0682	175.0609	17.998	[M-H]-1
	Isoleucine	C6 H13 N O2	0.99	131.0948	132.102	4.558	[M+H]+1
	Pipecolic acid	C6 H11 N O2	1.32	129.0792	130.0864	1.578	[M+H]+1
	Pyroglutamic acid	C5 H7 N O3	0.36	129.0426	130.05	1.767	[M+H]+1
	Adenine	C5 H5 N5	1.21	135.0547	136.0619	3.017	[M+H]+1
	Asparagine	C4 H8 N2 O3	-1.03	132.0534	131.046	1.434	[M-H]-1
Total	4-Methoxybenzaldehyde	C8 H8 O2	0.46	136.0525	137.0598	37.755	[M+H]+1
	Nicotinic acid	C6 H5 N O2	1.69	123.0322	124.0395	2.658	[M+H]+1
	2-Furoic acid	C5 H4 O3	-2.12	112.0158	111.0085	1.652	[M-H]-1
	L-Tyrosine	C9 H11 N O3	1.3	181.0741	182.0814	5.365	[M+H]+1
	L-Norleucine	C6 H13 N O2	0.99	131.0948	132.102	4.985	[M+H]+1
	Formononetin	C16 H12 O4	-0.32	268.0735	269.0808	23.759	[M+H]+1
	Nicotinamide	C6 H6 N2 O	0.88	122.0481	123.0554	3.193	[M+H]+1
	Fumaric acid	C4 H4 O4	-2.34	116.0107	115.0034	2.046	[M-H]-1
	Tryptophan	C11 H12 N2 O2	1.04	204.0901	205.0974	17.844	[M+H]+1
	Stachyose	C24 H42 O21	-0.24	666.2217	665.2144	1.574	[M-H]-1

4-Hydroxybenzoic acid	C7 H6 O3	-1.79	138.0315	137.0242	23.116	[M-H]-1
Ferulic acid	C10 H10 O4	0.55	194.058	195.0653	19.327	[M+H]+1
Ferulic acid	C10 H10 O4	0.23	194.058	195.0653	18.63	[M+H]+1
L-Aspartic acid	C4 H7 N O4	-1.63	133.0373	132.03	1.437	[M-H]-1
Cytosine	C4 H5 N3 O	1.09	111.0434	112.0507	3.07	[M+H]+1
Isoferulic acid	C10 H10 O4	0.07	194.0579	195.0653	21.789	[M+H]+1
Argininosuccinic acid	C10 H18 N4 O6	0.53	290.1228	291.1301	3.197	[M+H]+1
Chlorogenic acid	C16 H18 O9	-0.71	354.0948	353.0876	17.502	[M-H]-1
N-Acetyl-L-phenylalanine	C11 H13 N O3	-1.06	207.0893	206.0821	20.817	[M-H]-1
4-Hydroxybenzaldehyde	C7 H6 O2	-1.5	122.0366	121.0293	19.566	[M-H]-1
4-Coumaric acid	C9 H8 O3	-1.48	164.0471	163.0398	21.149	[M-H]-1
Protocatechuic acid	C7 H6 O4	-0.89	154.0265	153.0192	15.776	[M-H]-1
Shikimic acid	C7 H10 O5	-1.49	174.0526	173.0453	2.256	[M-H]-1
3-Phenyllactic acid	C9 H10 O3	-1.7	166.0627	165.0554	20.966	[M-H]-1
Isovanillic acid	C8 H8 O4	-1.14	168.0421	167.0348	16.864	[M-H]-1
Cyclo(leucylprolyl)	C11 H18 N2 O2	0.65	210.137	211.1442	20.898	[M+H]+1
Cinnamic acid	C9 H8 O2	0.63	148.0525	149.0598	19.313	[M+H]+1
Caffeic acid	C9 H8 O4	-0.64	180.0421	179.0349	19.517	[M-H]-1
Quinic acid	C7 H12 O6	-0.98	192.0632	191.0559	18.893	[M-H]-1
p-Coumaric acid	C9 H8 O3	1.06	164.0475	165.0548	17.064	[M+H]+1
2-Hydroxy-4-(4-hydroxyphenyl)butanoic acid	C10 H12 O4	-1.23	196.0733	195.066	19.061	[M-H]-1

Riboflavin	C17 H20 N4 O6	-0.2	376.1382	375.1308	19.708	[M-H]-1
Proline	C5 H9 N O2	0.97	115.0634	116.0707	1.551	[M+H]+1
Isorhamnetin	C16 H12 O7	0.49	316.0585	317.0657	22.214	[M+H]+1
Benzoic acid	C7 H6 O2	-0.84	122.0367	123.044	33.222	[M+H]+1

TableS3. Daily clinical symptom trajectories of individual calves in the Ctrl group, ND group and YGS group

Number	Fecal Score							
	D0	D1	D2	D3	D4	D5	D6	D7
Ctrl-1	0	0	0	0	0	0	1	0
Ctrl-2	0	0	0	0	0	0	0	0
Ctrl-3	0	0	0	0	1	0	0	0
Ctrl-4	1	0	0	0	0	0	0	0
Ctrl-5	0	0	0	0	0	0	0	0
Ctrl-6	0	0	0	0	0	0	0	0
Ctrl-7	0	0	1	0	0	0	0	0
Ctrl-8	0	0	0	0	0	0	0	0
Ctrl-9	0	0	0	0	1	0	0	0
Ctrl-10	0	0	0	0	0	0	0	1
ND-1	4	3	3	3	2	3	2	3
ND-2	4	4	4	3	4	3	3	3
ND-3	3	4	3	3	2	3	3	3
ND-4	4	3	4	3	3	2	3	2
ND-5	3	4	4	3	3	2	2	2
ND-6	4	3	4	3	4	3	3	3
ND-7	4	4	3	3	4	3	3	3
ND-8	3	4	3	3	2	3	2	2
ND-9	4	3	4	3	3	2	2	2
ND-10	4	4	4	3	3	2	2	3
YGS-1	3	3	3	2	2	1	0	0
YGS-2	3	4	3	3	2	1	0	0
YGS-3	4	4	3	2	2	1	0	0
YGS-4	4	3	3	3	2	2	1	0
YGS-5	4	3	3	3	3	1	0	1
YGS-6	3	4	4	3	2	2	0	1
YGS-7	3	3	4	3	3	2	0	0
YGS-8	4	3	3	2	1	1	0	0
YGS-9	4	4	3	3	2	2	1	1

YGS-10	3	4	3	3	3	2	0	0

Number	Dehydration scores							
	D0	D1	D2	D3	D4	D5	D6	D7
Ctrl-1	0	0	0	0	0	0	0	0
Ctrl-2	0	0	0	0	0	0	0	0
Ctrl-3	0	0	0	0	0	0	0	0
Ctrl-4	0	0	0	0	0	0	0	0
Ctrl-5	0	0	0	0	0	0	0	0
Ctrl-6	0	0	0	0	0	0	0	0
Ctrl-7	0	0	0	0	0	0	0	0
Ctrl-8	0	0	0	0	0	0	0	0
Ctrl-9	0	0	0	0	0	0	0	0
Ctrl-10	0	0	0	0	0	0	0	0
ND-1	4	3	3	3	3	2	3	2
ND-2	4	3	4	4	3	2	2	2
ND-3	3	4	3	3	2	3	3	3
ND-4	3	4	3	4	3	2	3	3
ND-5	4	3	4	4	3	2	2	2
ND-6	3	3	4	4	3	2	2	3
ND-7	4	3	3	3	3	3	3	2
ND-8	4	4	3	3	3	3	3	3
ND-9	3	3	4	3	2	3	2	3
ND-10	4	4	3	4	3	2	3	4
YGS-1	3	3	3	2	1	1	0	0
YGS-2	4	3	3	3	2	1	1	0
YGS-3	3	2	2	1	1	0	0	0
YGS-4	3	3	2	2	1	2	0	0
YGS-5	4	3	2	3	2	1	1	0
YGS-6	4	3	3	2	2	1	0	0
YGS-7	3	3	3	2	2	0	0	0
YGS-8	4	3	3	2	2	1	0	0
YGS-9	4	3	3	2	1	1	0	0
YGS-10	4	4	3	3	2	1	0	0

Number	Mental scores							
	D0	D1	D2	D3	D4	D5	D6	D7
Ctrl-1	0	0	1	0	0	0	0	0
Ctrl-2	0	0	0	0	0	0	0	0
Ctrl-3	0	1	0	0	0	0	0	0
Ctrl-4	0	0	0	1	0	0	0	1
Ctrl-5	0	0	0	1	0	0	0	0
Ctrl-6	0	1	0	0	1	0	0	0
Ctrl-7	0	0	0	1	0	0	0	0
Ctrl-8	0	1	0	0	0	0	0	0

Ctrl-9	0	0	0	0	0	0	0	0
Ctrl-10	0	0	0	0	0	0	0	0
ND-1	3	3	3	3	3	3	2	2
ND-2	3	4	4	3	3	2	3	3
ND-3	4	3	4	3	3	2	2	2
ND-4	3	3	4	3	3	2	2	2
ND-5	4	4	3	3	3	3	2	3
ND-6	4	4	4	3	3	3	3	3
ND-7	3	3	4	3	3	3	3	3
ND-8	3	3	3	3	3	2	2	3
ND-9	4	4	4	3	3	2	3	2
ND-10	4	4	4	3	3	2	2	2
YGS-1	3	3	3	2	2	1	1	0
YGS-2	4	3	3	2	2	1	0	0
YGS-3	4	3	3	2	1	1	0	0
YGS-4	4	3	3	2	2	1	0	0
YGS-5	4	3	3	2	2	1	1	0
YGS-6	3	3	2	3	2	1	0	0
YGS-7	4	3	2	2	1	0	0	0
YGS-8	3	2	2	1	1	0	0	0
YGS-9	3	3	2	2	1	0	0	0
YGS-10	4	3	3	2	2	1	0	0

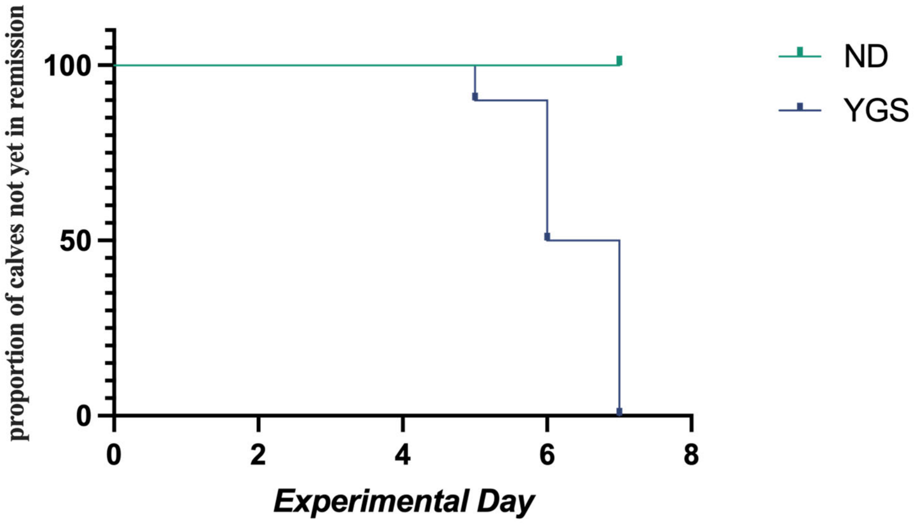

FigS1 Kaplan-Meier survival curves for clinical remission of diarrhea in calves from the Yigong San (YGS) treatment group and natural diarrhea (ND) group. Statistical comparison between the two groups was performed using the log-rank test (χ^2=18.81, P<0.0001), indicating an extremely significant difference in remission dynamics between the YGS group and ND group.

1. Shen, L.; Shen, Y.; You, L.; Zhang, Y.; Su, Z.; Peng, G.; Deng, J. L.; Zhong, Z.; Yu, S.; Zong, X.; Wu, X.; Zhu, Y.; Cao, S., Blood metabolomics reveals the therapeutic effect of Pueraria polysaccharide on calf diarrhea. *BMC Vet Res* **2023,** 19, (1), 98.
