# Supplementary material for: Yigong San Extract Modulates Metabolism, Antioxidant Status, and Immune Function to Improve Health in Diarrheic Calves
Source: Metabolites. 2025 Sep 18;15(9):618. doi: 10.3390/metabo15090618 (PMC12471574; doi:10.3390/metabo15090618)
Supplement: Supplementary file 1 [file metabolites-15-00618-s001.zip › Supplementary Information2.pdf]

Differences in metabolites between the ND group and the Ctrl group

Metabolite name	Rt(min)	Expreiment Mz	Adduct type	Reference m/z	MS/MS spectrum
Vasicinone	6.259	203.08476	[M+H]+	203.08148	55.05516:154704 56.05854:7220 58.99588:255021 60.04546:30948 76.02221:25236 81.04536:16807 82.06591:1254433 83.06922:45962 84.08145:13977 86.09729:32572 86.99054:721996 87.99407:21330 88.04028:22685 100.02253:20050 100.07646:33715 105.0007:1105592 106.00534:28181 113.04229:18226 123.01175:7537 123.09208:55863 128.05273:14794 130.06808:31868 140.05237:44108 144.10254:13235 146.02635:13870 157.08025:245730 158.06343:121123 158.08386:16023 158.11789:40332 159.06677:7265 160.07617:13852 162.05551:6820 168.04736:6274 176.06966:33906 186.05882:2073900 187.06213:148489 203.08376:173630 203.12617:41574 204.06444:15446 204.08656:21728 204.13911:21392
PE(16:0/18:1)	12.319	718.5365	[M+H]+	718.53809	53.00322:3539 55.0552:55583 57.03444:8184 57.07088:167587 58.06624:3932 59.0501:20051 60.08167:18921 67.05526:102955 69.07074:138473 71.04984:9507 71.08627:152944 72.08161:21099 79.05475:18494 81.07032:163958 82.07364:7619 83.08646:152712 84.08949:4431 85.06545:11112 85.10197:92689 86.09717:70265 87.09988:4945 89.06051:8710 93.07077:33599 94.07419:2863 95.08619:230228 96.08944:9975 97.10152:111616 99.08049:4028 99.11698:4387 104.1077:17630 107.08656:27736 109.10185:128978 110.10517:9226 111.08024:3792 111.11695:39919 112.12009:3803 121.10117:68674 123.11664:55623 124.99963:33023 125.13239:11103 133.0862:2984 135.11719:65932 136.12073:3481 137.1328:25468 149.1331:26285 150.13614:3213 151.14793:12836 153.12694:4965 155.00954:3286 163.14885:11875 165.05693:6915 165.16357:8791 170.05698:18577 176.8252:2672 184.07442:832292 185.07835:94966 186.07788:10815 221.22482:3875 222.11377:3048 224.12727:37420

					237.22235:3777 239.23796:41166 247.24211:10706 265.25262:50137 266.25891:4873 282.27975:18883 308.29404:51937 309.29709:8410 313.27151:22674 339.28925:27940 496.34015:3952 533.45624:28187 534.46008:3277 549.48792:41757 550.49127:4451 575.5058:8546 576.50226:11378 577.51489:1150001 578.5163:303823 718.55542:4812
N-Acetylneuraminic acid	0.951	308.09839	[M-H]-	308.09872	59.012:13425 78.98426:3278 87.00719:788865 88.01038:13205 88.03843:30210 89.02287:7339 92.92661:18837 94.92361:115706 96.92025:67563 96.95825:57128 96.96822:50403 98.05962:73827 100.03895:11028 101.02229:15370 102.05399:3122 112.04966:3740 119.0331:27029 126.0542:12023 126.90163:2118 128.03439:16453 130.0601:36696 136.91634:9975 143.04361:2464 144.0648:7747 145.09697:9713 145.99765:31244 146.04446:45909 154.92377:30598 170.00906:10236 170.04434:150852 171.04819:6443 176.89857:12743 180.0334:3613 187.07217:2598 188.01962:80550 188.05481:12717 188.07358:5757 188.10294:6828 190.01863:2872 200.05606:7364 218.0654:3353 239.96182:3377 250.01892:7904 290.08969:6692 308.09741:50356 309.10144:2393
Fasoracetam	6.847	197.12889	[M+H]+	197.12845	55.05513:3181 56.05017:3553 67.05522:7140 68.0504:80947 69.42701:6275 70.06613:36251 80.05054:11176 82.06591:32240 84.04561:4268 84.08139:712070 85.06543:8366 85.08451:25154 86.06055:13759 86.09711:2275 95.08609:4481 99.04493:3166 100.0766:2461 102.09196:2845 114.69359:2291 116.07122:20664 120.08135:22541 124.11244:11690 126.95622:7632 130.08717:418652 131.08928:22317 133.10072:2488 133.95093:2621 134.09653:2688342 135.10077:175295 136.11137:18302 138.05547:2881 141.05577:3602 142.04933:3566 144.96605:10292 151.08623:3515 151.12314:61055 152.07082:15367 152.10757:39420 152.12546:4107 155.97491:4159 156.06549:2367 161.1068:12227 162.09052:16838 169.09766:2757 169.13519:4597 173.98529:3427 174.98445:39614 179.08102:2472 179.11748:15809 180.06696:3429 180.10126:46530 197.09047:35421 197.12968:928444 198.0016:33550 198.07559:9978 198.11263:20923 198.13113:83857

Tenuazonic acid	9.18	196.09669	[M-H]-	196.09792	62.32889:1396 66.03338:120547 67.0364:1354 70.17924:4935 83.02361:5084 94.0274:1664 96.04298:1321 106.03917:1885 108.08055:17925 128.03337:10428 129.05463:3073 150.09094:185418 151.09534:10547 152.07065:20199 152.89195:3016 152.9045:4621 152.95293:7077 153.09117:2530 169.05022:2047 178.08588:6850 182.02002:2381 194.08241:3142 196.05797:13808 196.09564:320574 196.94481:1427 197.08119:4621 197.09956:23990
N-Acetylornithine	0.97	173.09186	[M-H]-	173.09312	58.02826:74093 59.01193:4567 74.02327:10123 84.04385:13218 85.07558:5088 87.05493:11951 96.96822:9271 102.05399:10388 105.01718:9719 111.00742:15322 111.05518:28047 111.07953:46574 112.07552:19023 113.07022:24152 114.05498:27418 116.03387:4155 125.07095:8887 127.04955:9706 127.08596:26634 128.03326:38993 128.07025:5069 128.87688:25234 129.06555:23720 129.09218:4872 129.10176:57207 131.08054:999478 132.0842:28398 137.07146:15176 146.0448:12862 153.06601:5338 155.04454:12822 155.08116:10313 156.07687:8372 172.84232:226308 173.05586:18365 173.09258:916747 173.84274:20155 174.0957:47649
LPC(P-16:0)	12.595	480.34473	[M+H]+	480.34479	55.05515:16695 56.05058:16000 57.03444:47315 57.07082:39786 58.06625:26038 60.08158:304102 62.06105:72813 67.05524:10642 69.07069:53358 70.33955:17645 71.07368:97273 71.08621:37372 81.07098:15465 83.08644:64255 85.10193:30310 86.09709:1726370 87.10065:46137 95.08612:36751 97.10148:50102 98.98515:50272 104.10734:3543513 105.11057:72924 107.08636:11113 109.1018:18935 111.1169:14931 124.99973:973547 151.01585:8973 155.00954:13342 181.02631:735446 184.07426:1524312 185.07567:49791 240.09988:677860 241.10541:29420 265.25226:10768 308.29355:32150 318.55392:9096 339.28775:303645 340.29166:38651 350.3754:9060 480.34528:4430660 481.3454:691261
1,2,3,4-Tetrahydroquin	6.847	134.09677	[M+NH4]+	134.09642	55.05516:5216 67.05536:31087 68.05046:3431 69.69501:12465 79.05464:12696 80.04995:133290 81.05369:5072 91.05516:11699 92.04962:3599 93.05791:6263 93.06984:10206 94.06556:4935 105.07008:11511 106.06593:57524 107.04961:4001

oline					107.06116:10568 107.08617:23510 115.05447:5457 117.07031:50836 118.06536:11222 119.07316:54375 132.08163:22826 133.08914:3617 134.06134:27681 134.07164:36186 134.09668:790302 135.04422:18656 135.05614:33816 135.08144:5292 135.10097:67545
4-Deoxyerythronic acid	0.964	119.03329	[M-H]-	119.03443	55.01723:6753 59.01208:28222 61.98694:15116 63.94749:1588 70.42747:1878 71.01182:7675 72.99165:20238 73.02769:17647 75.0073:13259 78.80746:1589 86.57849:1992 97.57877:1521 99.0067:3525 101.02255:15939 117.0181:2016 118.9248:2389 119.03313:154798 120.03712:6950 120.05583:1517
Tri(butoxyethyl)phosphate	12.57	399.25034	[M+H]+	399.25058	55.05518:256499 57.07084:824706 58.07381:13497 59.05003:75854 70.49087:9382 73.02943:27290 83.08639:297361 84.08952:7429 89.06053:42668 98.98512:139595 101.09658:550036 102.09984:29662 124.9997:67686 142.12329:37668 143.01106:166029 184.07448:35771 195.23276:7201 199.0735:1197917 200.07631:40558 225.08824:29542 243.09909:35155 264.26764:12460 271.15475:7481 299.16281:520129 300.16669:42860 396.58807:8405 399.25305:107742 400.24606:7680
Asparagine propyl ester	1.407	175.10757	[M+H]+	175.10768	60.05647:730474 62.98244:13647 68.05045:8019 69.5567:33442 70.06616:1351358 71.06918:31495 72.08168:63374 84.04491:30671 84.08137:95325 86.99348:71402 87.04441:14115 98.06049:10751 111.05584:30758 112.07607:938166 112.08666:72898 113.07195:69763 113.07993:38357 114.05559:11875 114.10297:24230 115.08694:67431 116.07108:803955 117.07394:23188 129.06609:13994 129.10214:225193 130.0508:44495 130.08728:58448 130.09854:255555 133.92892:7137 134.01845:299722 141.06694:6515 151.94061:8392 157.06137:49942 157.11005:38491 158.0305:50021 158.09276:222405 159.07608:128765 175.07217:82434 175.12038:988588 176.04066:126409 176.06944:8520 176.10712:14404 176.1244:44111
2-Ethyl-1-hexanol sulfate	13.495	209.08458	[M-H]-	209.08476	65.01305:3430 68.35537:4408 68.35912:9908 78.95692:8509 79.95505:11322 82.0394:11609 95.95105:6307 96.9584:265774 118.99118:2425 124.03857:2891 126.01812:2254 156.0546:2436 164.92654:2983 164.98683:9392 180.96478:2974 182.04553:3432 183.03027:3285 183.06503:11514 208.82257:8326 208.9772:6146

					209.08331:1039015 210.04138:2310 210.08881:60614
PFSM-alcohol	8.107	291.99097	[M-H]-	291.98837	69.24303:6771 72.00746:2659 72.99104:8934 74.02299:13428 87.92345:5399 91.05335:7765 99.92422:18965 100.00176:3251 100.90348:10833 100.93105:2254 103.91862:28120 104.92642:9748 106.94178:3302 115.91911:21978 116.9271:12000 117.03253:1934 118.94205:2334 121.02738:2178 123.94438:7092 129.05325:8440 129.93033:10608 129.95781:113160 133.02777:6827 142.94147:2934 146.05989:2544 147.96857:9506 148.07477:15256 155.93814:12146 156.92346:10289 162.83743:9045 164.96283:3550 173.92064:6490 178.97774:10644 186.93153:5220 188.96475:2004 190.05096:7814 190.95117:10534 192.06473:3451 201.99431:6864 203.97484:137697 204.01038:63152 204.97574:8307 205.01471:5292 206.97493:48680 215.97305:16160 217.98911:2674 220.00548:35088 221.00856:2172 221.98404:3172 223.99896:7376 231.97032:1897 233.98381:6568 245.98567:78007 246.98724:8363 247.98798:55136 247.99908:575273 249.0024:52884 249.98148:9171 291.95911:18134 291.99222:59246 292.03955:1915 292.99329:5553
(2S,3S)-3-decyl-2-hydroxy-2-(3-methoxy-3-oxopropyl)butanedioic acid	12.614	361.22278	[M+H]+	361.22208	57.07073:42168 68.99796:15335 69.0707:2670 87.00822:3337 111.00775:16539 129.0188:1059611 130.02113:44100 139.00342:32079 140.00671:2244 147.02896:10929 157.01297:98343 184.07446:7267 185.08054:628907 186.08508:48755 203.0892:7694 211.76248:2150 213.07497:7617 249.06624:3200 259.1524:32465 260.15625:2887 313.45547:2165 333.28137:2173 361.15079:16680 361.233:224291 362.23798:45061 362.32709:16779
Monooctyl phthalate	12.27	277.14401	[M-H]-	277.14453	60.99129:5487 70.00613:3148 106.03938:31807 107.04895:16649 121.02859:126126 122.03117:6196 127.11069:167040 128.11414:10801 129.12695:1814 134.03604:385599 135.03954:18089 147.00719:19890 151.03882:6350 165.01907:8771 173.11632:7428

					190.99805:15533 205.15935:25141 231.13812:1780 233.15407:41188 234.15697:6025 249.14755:6794 277.00671:6979 277.14227:229102 277.1817:18280 278.14716:23818
Propionylcarnitine	5.391	218.13881	[M+H]+	218.13924	57.03445:7305 60.08166:174301 70.11963:6358 73.02942:11741 85.02905:1364282 86.03288:41546 87.04517:6984 101.05992:13822 111.04474:6673 114.05558:3937 121.10114:4503 129.05496:13204 144.10263:40147 147.06447:6232 159.065:261243 160.06851:16153 200.12729:12965 203.1442:55275 218.14008:626420 219.14413:48867 219.17351:418856
ent-1(10)-Halimene-15,19-dioic acid	12.199	335.22308	[M-H]-	335.22223	59.01195:2035 65.01261:1431 69.03312:2615 71.01184:1191 74.6549:1324 77.65511:1385 83.04875:1366 94.28392:1311 114.44529:1357 119.25764:1225 134.07292:2866 137.09584:5199 147.07979:2322 151.11108:2266 151.84666:1305 153.08942:6579 153.12715:8429 155.10686:1619 159.08038:1622 165.12776:1688 177.09158:2288 181.08549:11345 183.1011:22254 192.81165:1218 195.10197:6068 219.17528:1357 249.36412:1363 255.21281:1999 263.20297:1727 273.22031:22328 274.22528:1932 286.83249:1611 289.21545:2753 291.19885:4477 291.23184:14282 292.23846:1799 294.74179:1344 299.20233:8169 317.21054:41350 335.22372:119451 336.22748:11618
L-alpha-Amino-1H-pyrrole-1-hexanoic acid	6.82	195.11272	[M-H]-	195.11336	55.73517:1488 57.54532:1437 59.95698:1360 66.03323:346054 67.03639:8825 69.39298:2637 69.39627:4510 72.00743:2307 80.04865:14089 92.92765:9285 96.83882:1636 107.34144:1388 111.04401:3056 114.05492:1603 114.11964:1402 124.03862:2623 125.03379:1870 128.03313:2520 128.07027:93370 128.99066:1419 141.02875:1506 147.09155:1779 148.96709:2563 151.04936:2367 152.07045:1743 160.83972:44199 178.08585:3184 195.11234:1853522 195.80939:44127 196.05914:2121 196.11681:139374
Methionine sulfone	1.358	182.04868	[M+H]+	182.04819	55.05515:21995 56.05058:2501331 57.05367:64798 62.98291:5830 69.45832:15008 70.0662:7245 72.08165:3638 83.04979:6157 105.03432:5019 105.99648:3471

					107.93108:3391 111.04509:26810 122.0598:3616 123.04412:4536 123.05706:4060 129.05496:4279 136.04247:1152424 136.07556:14129 136.1132:6201 137.04573:36166 137.05942:5379 140.07115:5484 154.08571:6377 164.06981:5712 165.02269:41974 165.10323:7273 174.01584:3632 182.0099:15886 182.04958:624836 182.08011:26062 182.11717:18957 183.05125:23393 183.07661:4630 183.11441:30573 183.14946:6832
Metolachlor	12.247	284.14072	[M+H]+	284.14117	57.07081:8176 65.36414:1960 67.05523:2324 69.07068:2889 71.0862:2571 73.0294:10986 73.06554:128222 74.06909:3270 76.97979:3252 81.07027:2964 81.37923:2065 85.06544:2621 89.06046:41088 90.06371:8284 95.08611:3258 109.10179:3010 119.08539:2715 121.10111:2077 133.0862:18726 134.09769:11763 135.0799:1942 137.13246:1945 146.09644:13399 158.09703:8996 160.11273:12132 174.12906:3031 176.14261:377951 177.14685:43326 184.05336:11866 188.1093:1949 194.0732:13013 211.07727:2022 212.08418:71159 213.0867:3387 233.89644:2033 239.15106:17871 240.14955:1972 243.11339:4031 252.11362:3112214 253.11884:420320 284.14249:188301 284.29242:50361 284.32877:9757 285.07596:6793 285.14526:27826 285.181:5682 285.21408:14255 285.29977:3740
N-Acetyltaurine	0.962	166.01656	[M-H]-	166.01741	58.02819:3522 66.0332:14507 69.28674:4280 69.29057:5077 79.95581:193575 80.96347:61450 88.03864:61149 92.92603:29266 96.84063:3760 100.03873:14930 102.05413:47275 106.02155:3967 106.97961:154015 122.05929:15126 124.00606:81543 127.8684:3526 130.06033:22055 138.92737:17116 164.83626:3372 165.8364:37623 166.01678:2258287 166.83119:147273 167.02026:72041
2-Methylpyridine	6.351	94.06568	[M+NH4]+	94.06567	50.5167:27739 51.02379:9992 53.03951:138699 55.05515:10693 56.96547:9413 59.52196:28218 62.0248:35984 65.03959:7131 67.05524:73492 69.8398:9782 71.02996:976790 71.53044:119320 72.02881:91489 73.53275:8501 77.03931:62194 78.03791:8567 80.03493:335086 80.53563:42423 81.03428:41476 82.53815:216900 83.0383:28269 83.53643:15325 89.04059:7597 91.54323:43641 92.05798:9687 93.05772:33648 94.02921:17815 94.04617:38450 94.06552:2934104 95.05002:977566

					95.0615:80802 95.06941:139046 95.08599:61822 96.05283:22080
Aldosterone	9.659	405.19122	[M-H]-	405.19189	58.60857:1313 90.74675:1365 112.44896:1534 125.05902:1760 137.0593:5303 189.35707:1430 201.82286:1314 219.30763:1344 257.09863:1488 299.16302:3070 301.18027:29132 302.18427:1533 311.16312:9218 327.15964:12625 329.1767:120682 330.18112:13647 341.17514:1494 405.26917:1953
5-Methoxysalicylic acid sulfate	6.972	246.99159	[M-H]-	246.99182	69.90848:3091 79.95592:88320 96.84155:2329 96.95847:175458 103.91842:3816 108.01981:56294 116.92692:2676 118.94203:9519 123.04372:155268 124.04651:6066 132.0437:1665 152.00999:300159 153.01405:14935 162.93205:3258 167.03381:2156506 168.03738:153765 178.94778:2120 179.03181:4024 198.04251:1962 200.87984:2236 202.99997:62467 204.005:1688 213.06824:13682 246.99083:446153 247.99553:29772
Platelet-activating factor	13.224	524.37103	[M+H]+	524.37109	57.07081:149578 58.06666:245299 59.07416:70240 60.0816:1398612 71.0737:80285 71.0862:83902 86.09715:6081708 87.10064:164312 98.98515:148466 104.10737:1262822 124.99978:2653065 184.07446:69774480 185.07591:1797156 258.11081:184884 341.30597:225516 506.3588:619973 507.36456:137591 524.375:2030892 525.37885:416553
2'-Aminoacetophenone	6.347	136.07588	[M+CH3OH+H]+	136.07623	69.09684:24100 81.07047:87798 91.05514:136808 94.06557:194347 95.08619:92573 109.07697:8892 117.05713:25989 118.06531:1332381 119.0349:12272 119.06934:100213 136.02135:369283 136.03944:32880 136.06203:641696 136.07558:554786 136.11171:27133 137.02443:15037 137.04562:137518 137.07626:12101 137.0793:47099 137.10814:43853 137.13251:13169
Kynurenine	6.347	209.09193	[M+H]+	209.09209	65.03952:12953 70.06612:10538 74.02431:1327066 75.02804:28857 77.0387:41675 88.04016:272080 91.0545:34771 94.06548:7463688 95.04998:24539 95.06924:375957 98.02447:20140 99.00841:776955 100.01199:23081 104.04979:228818 116.03436:60727 118.06524:786371 119.06901:48942 120.04498:1658729 121.04796:114550

					122.06087:57277 132.04533:210621 133.04822:11501 136.07547:3605374 137.07918:243742 146.05974:4386470 147.06438:298457 150.05568:1997614 151.05809:151205 163.08742:818039 164.07167:500401 164.09021:60813 165.07504:35623 174.05489:2665387 175.05882:258798 191.08249:46462 192.06598:4886308 193.06981:460345 209.09305:368998 209.13756:50885 210.09457:23980 210.11191:17940
2-Hydroxyphenyl acetic acid	8.299	151.03864	[M-H]-	151.04002	59.01194:6791 63.00175:21670 65.0131:2894 66.03323:6488 67.0286:6182 68.0126:3232 71.01263:9097 78.98427:3021 81.04431:7009 83.02362:7806 89.02287:5840 101.02247:5542 106.9911:102398 107.04884:463730 108.04422:7766 108.05168:20107 109.03944:15588 110.02344:7995 110.03439:6993 111.01852:5006 122.95036:25568 123.04356:10086 124.03865:8646 124.05045:5740 125.03382:7418 134.03462:8397 136.01527:23295 150.98221:16330 151.01421:7077 151.02477:5719 151.03885:56257 151.04941:23097 152.03438:10269 152.04523:14121 152.0705:2630
PFAP-diPAP	8.909	332.93726	[M-H]-	332.93799	69.35957:9358 79.95588:83863 80.96361:29339 107.04887:207402 108.05169:11391 164.99902:72214 187.00667:1478280 188.01006:72937 189.00171:3957 208.98886:70370 209.99248:4437 232.76315:2926 234.7597:3544 242.94377:6514 252.98099:29021 333.01102:10450
OPFC-perfluoroalkyl_sulfate	6.283	353.00784	[M-H]-	353.00992	69.77452:2472 71.01184:8106 72.00747:6495 87.92373:11318 87.997:2291 90.93153:2231 92.92684:1688 94.9912:5108 99.92404:2611 102.94671:2246 103.91876:10829 104.92644:2424 105.93365:1803 114.05381:5262 123.94439:9078 126.01823:2204 127.94255:1840 131.98692:9614 144.04314:10006 144.95786:8375 146.93777:7231 147.98224:1742 148.90855:1773 158.99669:1651 168.98358:2244 171.93233:13777 172.03912:1795 174.96957:2176 175.93944:2120 188.94753:3094 189.99406:15342 192.89803:7178 193.9953:1504 201.99432:2721 202.98895:45266 214.98926:5794 215.95822:2430 217.00648:3063 217.98912:3530 220.00548:8107 221.9872:1478 233.98723:9039 235.99959:1881 238.0834:9992 243.9706:8216

					245.98537:14903 249.04694:10351 260.99786:41618 261.04398:17760 262.00024:1903 262.05252:3390 263.02795:12810 263.99384:13861 289.97729:28121 294.04269:156150 295.0444:3019 296.0419:1769 307.01031:16061 308.02197:6073 316.96408:1547 325.01398:2066 353.1105:213358 354.11374:17942
Methyl 3-[(1E,3E)-3,5-dimethyl-1,3-heptadien-1-yl]-8-hydroxy-6a,8-dimethyl-6-oxo-6a,8,9,9a-tetrahydro-6H-furo[2,3-h]isochromene-9-carboxylate	11.675	437.1925	[M+Na]+	437.19342	52.73198:2168 58.06582:12020 60.0816:2622 65.09783:2350 69.82485:5289 86.09713:3394 89.06059:26607 95.08612:2749 98.98515:2602 99.62807:2399 133.08644:15653 140.14348:3369 155.01137:8940 177.11324:2493 184.07443:4712 195.12151:2887 210.18434:2707 239.15048:20930 303.12286:61841 376.25882:3022 419.17938:2415 422.16617:2395 437.19122:3435821 438.1983:557010
4-Hydroxyquinoline	6.347	146.0602	[M+H]+	146.06007	71.01636:6308 82.0659:13942 84.08146:6451 86.06079:4294 91.05537:31902 95.05009:8609 97.00809:22054 100.04816:5935 100.0767:67511 100.11273:8106 104.04997:19962 117.05833:4819 118.06526:75250 123.96442:28199 128.05:9246 146.05986:2118886 146.0833:7007 146.11847:14210 147.0647:163201 147.09323:6545
Tyrosine	3.809	182.08134	[M+H]+	182.08118	69.24033:155680 69.24416:60618 91.05502:894906 95.04977:291479 103.05459:61074 105.93544:45990 109.06508:55007 113.96438:46057 119.04962:3298358 120.05263:175186 121.06577:186780 123.04408:6387994 124.0483:386206 136.07555:17586776 137.07921:1085939 147.04408:3249139 148.04703:85286

					163.94034:135654 165.0549:8332816 166.05916:534427 182.08208:877703 183.11186:61959
PE(18:1/18:2)	12.313	742.53961	[M+H]+	742.53809	55.05516:131584 57.07082:85955 58.0637:15818 58.06664:76424 59.05003:24081 59.07413:13305 60.08158:301516 67.05518:397437 69.07065:300318 71.07347:81669 71.08621:66222 79.05516:92432 81.07092:514849 82.07359:11881 83.08633:255423 85.06541:23865 85.10173:44410 86.09707:1442700 87.1006:29039 91.05537:46864 93.07059:153869 95.08607:508129 96.08939:33158 96.8424:14462 97.06513:24008 97.10142:165637 98.98447:44432 99.08044:20627 104.10722:148652 105.07101:29924 107.08621:120668 109.1018:247686 111.08018:24404 111.11648:53804 119.08534:26699 121.10096:157696 123.11785:128120 124.9996:637624 127.11235:11338 133.1019:52042 135.11707:165879 137.13206:47997 146.98152:12664 147.11682:28409 149.13277:68570 151.14786:24861 161.13197:32166 163.14877:42101 165.1635:12871 175.14897:23796 181.02635:12824 184.07411:16273722 185.07794:482230 245.2265:10062 247.24199:36189 263.23517:70526 265.25214:97184 306.27939:96960 308.2934:28700 319.26672:10775 337.2739:54377 339.28778:52779 480.34537:36505 502.32874:95024 601.52124:1928488 602.51593:535246 742.5791:479052 743.57831:166984
Methyl syringate	1.221	235.05719	[M+Na]+	235.05772	52.19751:7285 71.01864:11064 79.16743:6743 80.94859:12264 82.17883:6772 96.92207:17517 98.54579:8065 98.91988:857702 106.95094:17403 120.00404:115447 142.9091:7188 175.02382:50864 177.02638:69114 177.06183:78663 193.03427:12324 194.58755:6684 199.99045:11267 231.70013:7912 235.05769:1090297 235.10558:44931 236.05939:65906
Tridec-8-enoylcarnitine	10.172	356.27969	[M+CH3OH+H]+	356.28009	55.05515:11757 57.03444:8299 57.07082:10092 58.06625:2745 60.08163:172896 61.08515:7183 67.05524:7890 69.07067:24130 69.1939:7211 71.08622:8333 73.02942:12648 81.07087:15468 83.08633:37755 85.02901:1522999 86.03274:50372 93.07067:3992 95.08626:18066 97.10143:46178 107.08636:3818 109.10181:3063 111.11688:25130 121.10142:15370 126.05482:3509 131.03381:10213 133.0862:4009

					135.11716:7153 136.06201:4241 144.10242:47254 177.16425:20557 195.17581:9588 283.17288:2971 297.2049:138944 298.20953:23293 309.90161:2590 356.27731:808447 357.28073:160498
(R)-3-Hydroxy-5-phenylpentanoic acid	10.954	193.08562	[M-H2O-H]-	193.08647	52.58442:1281 52.65538:1541 60.78577:1277 66.03318:6273 67.69871:5573 67.70241:14984 68.39505:1422 78.59023:1499 80.04944:1302 80.97302:5512 82.0394:1481 83.02356:1928 90.93172:35513 99.92396:1843 101.27518:1405 102.93176:1850 102.94662:2313 108.04308:6181 112.93563:2903 115.91891:1483 125.92693:7014 146.92244:5827 149.05847:3040 149.09663:91293 150.05417:1933 150.0995:6803 151.0493:1357 165.92342:1906 178.02493:5662 193.08603:572496 194.04384:1472 194.09041:47640
Jatrorrhizine	8.119	337.99701	[M-H]-	338	71.01411:1500 74.02292:29860 80.03931:1457 82.94548:1597 87.92345:1878 102.9467:3422 103.91859:14273 104.92643:2174 114.05381:2722 129.92252:1913 129.95786:43161 146.93803:12521 147.96858:2620 148.07448:2840 162.89264:3238 173.94893:2324 178.9796:2610 190.92619:2276 191.95874:3484 192.06454:15077 203.97482:55949 204.01047:19171 204.95625:1627 206.97484:16626 215.97325:6816 220.00456:10648 223.10815:3384 224.00215:2023 225.99312:1875 233.98383:2334 245.98506:45119 246.99094:2599 248.00276:432883 249.00294:26194 249.98149:2054 291.99203:155862 292.9928:10214 338.01779:2633 338.17111:1699
5-Bromotryptamine	8.908	237.0067	[M-H]-	237.00301	69.35848:3982 69.36177:2869 71.01241:1850 79.956:190860 80.96358:35043 85.02799:2118 87.92345:1874 93.03323:11379 95.01227:3251 96.95859:56526 97.02831:13206 103.91844:2693 107.04902:25188 109.02765:3130 111.04406:6425 121.02769:24800 123.04377:14287 124.01523:32232 134.8644:32897 136.86185:69983 137.0229:9701 138.85883:11751 139.03928:82760 140.04124:2436 157.05051:1751 187.00677:81566 188.01003:7153 203.00027:48389 204.0078:2862 209.01172:31538 210.01558:2072 218.99673:22292 220.00238:2456 236.75809:23783 237.00682:85884 238.01015:5052

o-Cresol sulfate	8.91	187.006	[M-H]-	187.0065	69.51268:538644 79.95586:19874758 80.96342:6308005 107.04887:58627104 108.05177:3127576 125.09617:621233 187.00676:85497792 187.09647:649718 188.01019:5005610
Allantoin	0.928	157.03537	[M-H]-	157.03671	59.02345:108661 69.2802:25112 71.02322:371531 72.00745:25218 78.95759:158499 96.92015:173106 96.95827:226551 96.96823:682908 97.00262:4604796 98.00605:106350 98.95441:17901 98.97215:15232 109.03952:21693 114.02946:4224498 114.09077:15645 115.03309:95078 128.89905:68023 138.91333:119313 140.00815:269922 156.92363:48375 157.03551:668411 157.86189:23407 158.08054:98741
Anisole	8.907	107.0485	[M-H]-	107.04969	64.00526:17707 65.0127:117282 65.99661:43709 67.92173:66908 67.92545:58812 96.83706:11818 96.84248:25763 106.04137:69687 107.03416:104808 107.04886:2041471 108.05161:107831
1-(Diethylamino)ethanol	13.184	118.12286	[M+H-H2O]+	118.12264	53.03948:1691 55.05524:66182 56.05856:2745 58.0659:43039 59.06141:11671 59.07378:51293 71.02942:1709 72.08168:181108 73.06556:54364 73.0851:7260 74.06911:1488 77.03493:2009 79.05531:1500 87.04442:4862 87.05597:1987 91.05507:13411 95.05009:7723 96.04482:6022 100.11276:226869 101.11558:13225 117.07034:1672 118.06522:12917 118.08604:204491 118.12258:73997 119.06093:18173 119.08572:24333 119.12611:2512
beta-Ureidoisobutyric acid	1.389	129.0661	[M+H-H2O]+	129.06586	55.05515:36836 56.05024:67089 57.03445:15786 57.07082:7995 58.06583:8039 67.05524:29388 69.59633:17213 69.60018:15984 70.06564:9761 71.04986:28580 72.0451:7276 83.06126:2021380 84.04492:1306561 84.05953:7731 84.06454:75157 84.08144:402521 85.02901:8997 85.06552:103147 86.06079:10050 95.04921:7058 106.74352:8116 112.07624:113927 119.02998:8203 129.01881:40578 129.06606:606508 130.05067:208172 130.0704:13871 130.08592:484629
4-Methylumbellif	8.907	254.99425	[M-H]-	254.99692	69.34534:12478 69.34917:4599 79.95589:127426 80.96348:49833 81.95144:4286 82.95909:12048 87.15441:3106 96.83976:4371 107.04891:288633 108.05169:15495 110.38334:3474 157.86942:3264 173.06021:13407 187.00667:2430946 188.01004:157186

eryl sulfate					208.96877:3183 219.84459:183302 227.07642:15360 247.52931:3167 255.01364:22782 255.0891:6483 255.23213:10952 255.82098:11549 256.01514:3517 256.15506:3371
Phenylacetylglutamine	7.592	263.10324	[M-H]-	263.10373	52.83709:1337 69.99505:3170 69.99838:2484 84.04388:2103 92.92769:2702 97.03892:1763 99.92404:1909 103.91844:1578 109.03845:7044 122.02354:1365 125.03384:5946 127.04964:59232 128.0329:13956 128.05241:2328 145.06055:514936 146.06366:19510 159.15082:1510 172.93938:2075 175.05521:2419 224.04987:1371 245.09488:2815 261.86353:1468 263.10165:50750 264.10843:5655
Phenylacetylglycine	8.117	192.06575	[M-H]-	192.06662	68.04352:9849 70.0762:20837 76.04041:5782016 77.04317:107957 89.06042:172399 91.05509:7762018 92.05794:374075 120.08117:117424 133.0862:16935 135.04419:49306 148.07605:56587 176.07164:37594 188.3567:10012 194.08073:121098 194.11688:133369 195.11375:14791
LPE(20:1)	12.539	506.3244	[M-H]-	506.32523	78.95717:442421 96.84058:17072 122.98404:5142 134.98439:10048 152.99435:166852 168.04176:257168 194.05663:4857 224.06888:1186380 225.07155:37809 242.08032:276709 243.08368:4903 281.24805:12079169 282.25272:1126699 417.2431:5357 506.32007:2617102 507.32672:408452
m-Aminobenzoic acid	6.347	120.04475	[M+H-H2O]+	120.045	68.43901:1630556 77.03941:625028 79.0553:332068 80.05057:335376 91.05513:3922874 92.04961:297642 93.07069:13137498 95.05016:1917392 102.047:262775 103.05463:49146788 104.05801:2402046 105.04555:278335 118.06535:1188694 119.07309:447088 120.08137:130397856 121.08478:5528035
Hexanoylglutamine	6.227	243.1348	[M-H]-	243.13503	58.02818:6656 59.01191:1615 66.03321:10875 68.0489:13653 68.4422:12222 82.02814:2831 84.04383:10469 86.05972:2963 88.98698:1584 96.83789:1634 105.31992:1447 110.02229:2100 112.07549:2810 118.96535:1800 128.03447:2078 129.10185:1635 130.04909:258288 130.08549:7072 131.05197:7533 141.10181:1802 143.11832:235459 144.12186:10635 146.95961:34984 153.03003:6280 171.1118:260653 172.11678:16889 174.95412:57115 179.11809:2993 181.13348:278632 182.13676:24566

					199.14395:1648 225.12547:2194 225.14789:1560 243.13405:647632 244.13712:50986
PyroGlu-Pro-Arg	12.616	383.20395	[M+Na]+	383.20377	57.07093:12650 69.59138:3300 69.59413:3623 81.0703:2855 89.06053:9351 95.08614:1929 96.84428:2157 104.10738:8135 121.06569:12971 133.08623:3590 165.09117:8067 184.07446:9770 207.06349:13340 211.05698:2881 225.07222:2396 239.38202:1950 267.12222:40940 268.12296:2650 271.07855:3277 271.15475:4436 281.13629:30911 282.14081:2732 327.07944:2556 327.14069:95783 328.14233:10830 338.34131:3714 383.20627:1218895 384.20468:191924
Deacetylovatifolin	6.821	263.10141	[M-H]-	263.09991	59.13422:1336 66.03336:49416 68.56978:1393 70.39104:1837 80.04813:1848 86.80202:1385 92.92681:2091 101.48629:1462 128.07059:19700 135.01721:1324 148.94814:1996 155.50514:1400 172.93932:1612 195.11227:1190592 196.11671:89419 236.05109:1594 263.10162:41616 264.10428:5522
4-(2-1,2,3,4-tetrahydroisoquinolylsulfonyl)thiophene-2-carboxylic acid	8.119	322.02368	[M-H2O-H]-	322.02133	71.04704:2514 74.02285:163921 91.05335:2820 106.9271:3328 117.03374:1593 127.96481:10999 129.05466:8197 143.95993:2487 146.95944:10647 157.97672:2723 173.97083:54457 174.0556:5303 174.95419:8566 175.98579:13822 192.06477:65195 203.98293:6108 230.01248:2206 239.96892:5655 246.00729:18824 248.02484:60566 249.02831:3197 250.03699:11630 258.00815:2425 276.01694:642952 277.01974:43451 283.96063:15554 292.01117:5488 294.02756:29274 322.02307:64909 323.02802:5612
3',5'-Cyclic CMP	8.121	306.04697	[2M-H]-	306.04858	70.08957:2110 72.99104:1519 74.02291:57142 99.92404:1697 103.91844:2430 104.15899:1615 130.98236:5729 132.01352:3376 141.99797:2278 146.93608:2455 152.92068:1496 158.97768:8740 159.9805:4931 160.00932:47705 160.9754:2846 161.01027:3400 192.06514:18798 206.06491:9662 214.92648:7135 232.04732:105439 233.04724:12081 234.06206:27958 235.06364:2437 260.04044:379531 260.93359:6925 261.04138:45687 269.55667:1537 278.05045:2283 278.94086:6101 306.0531:14174 307.05011:2149 307.13687:1712

Decarestrictine D	8.925	239.08945	[M+Na]+	239.08902	53.42403:1676 55.01875:1696 55.05516:27980 67.05524:2891 69.07069:2914 71.01749:1533 73.02942:2642 81.07098:1796 83.04987:53301 83.08656:12916 84.08146:2005 85.06562:16563 87.0447:11511 89.02408:10500 89.06061:52330 101.06007:58374 102.83071:1505 111.04482:226100 111.08137:2797 112.04781:11357 112.07622:1717 113.06055:5478 127.07569:18533 129.05504:84967 130.05774:2697 130.08588:2467 137.05942:3098 146.96135:1771 147.06616:7345 155.07007:372133 155.88634:1587 156.0733:22197 157.10069:1839 172.09631:2036 173.08134:339024 174.08324:27007 176.9725:1677 190.08722:1884 190.1071:1560 199.09758:35085 200.09239:2281 200.12729:7757 217.10785:316357 217.19577:3584 218.1097:24221 218.13995:2572
LPC(18:1)	12.537	566.34668	[M+FA-H]-	566.34631	55.05515:299934 58.06625:530289 60.08164:6452806 67.05472:284091 69.07069:325332 71.07372:426313 81.07098:365026 83.0864:356267 86.09713:19185798 87.10065:412570 95.08612:398174 96.85149:355451 104.10738:95992152 105.11088:3467219 124.9998:7118588 163.01456:281995 184.07439:112710144 185.07852:4108397 225.93611:426583 258.11136:1619362 339.28741:2100208 340.29166:272230 347.06506:260514 370.09207:247660 433.8894:253026 504.34323:9711253 505.34546:2407617 522.35388:39701476 523.36255:10787739
Pantothenic acid	6.474	220.11847	[M+H]+	220.11798	55.01875:20536 57.07082:161001 59.05006:158811 67.05532:86625 68.77655:40060 68.78034:33642 69.0707:244120 70.0295:63360 72.04509:569914 73.02946:98034 74.02438:22094 83.04978:21358 85.06546:437700 86.09711:73824 87.08131:115071 90.05566:6793084 91.05349:21184 91.05926:113683 95.05025:189629 98.02458:501129 100.03956:133915 103.07546:520703 104.07916:20282 113.06049:134043 116.03452:957854 117.0379:41923 124.07582:632581 125.07932:26260 131.0708:61967 142.08638:404643 143.0891:20883 156.10274:76608 160.0972:175554 166.08723:150832 174.11177:188591 175.08754:48590 184.09819:1441386 185.0999:118778 202.10744:2209195 203.1114:174213 205.15811:53776 220.11823:2675530

					220.17525:90505 221.12186:204974
N-(2-chlorophenyl)-2-(9-methyl-6-oxohydropurin-8-ylthio)acetamide	8.894	350.0484	[M+H-H2O]+	350.04727	52.15997:1241 68.80146:1219 71.0192:1875 102.65787:1433 103.07742:1250 138.59248:1436 163.0067:1944 176.09163:1988 181.01743:26448 183.03201:2471 184.00343:1850 199.02852:59994 200.03069:5942 204.03407:1432 205.09686:1685 217.03754:10576 222.04478:5923 244.97879:10578 260.06476:1240 262.16565:1477 262.98907:64416 263.99078:4347 267.99393:2996 280.9982:86493 282.00153:5060 286.00491:30213 287.00769:2736 288.04346:2428 299.01099:8238 304.01566:35092 305.01886:1798 308.96674:30807 309.02393:8867 309.96893:1736 326.97849:2555 327.03458:7112 331.98682:7540 349.99014:1440 350.18893:4779
Trans-Hexadec-2-Enoyl Carnitine	10.873	398.32693	[M+H]+	398.32703	55.05515:10518 57.03439:14351 57.07084:20353 60.04541:6031 60.08159:175616 67.05511:15482 69.07059:20134 70.03616:4211 71.08633:17712 73.02941:7715 79.05529:2866 81.07088:27813 83.08641:29495 85.02898:1361561 85.10193:7581 86.03265:35224 86.09707:11475 88.07632:6085 89.06052:7601 93.07066:10510 95.08604:26832 97.06519:5819 97.10148:16484 99.08143:2356 107.08636:8527 109.10165:15834 111.08135:3416 111.1169:6907 121.10107:20921 123.11793:7293 125.09657:3247 126.05482:2022 133.0862:2393 135.1171:22021 137.13248:6915 139.11229:3139 144.10239:40800 147.1169:2724 149.13254:11843 153.12874:3159 157.05026:7860 161.13206:2498 163.14886:7504 167.14197:2338 175.14687:2204 177.16467:3145 179.99141:2391 184.07443:3683 195.17322:2475 198.00165:3477 219.21048:19658 225.99733:10381 227.12608:2165 237.22183:25330 238.22351:2221 241.14447:8745 244.00746:2411 249.01402:2475 255.15913:7625 269.17529:10864 283.19235:11428 297.20474:3056 311.18875:3647 321.20721:2272 336.32578:8325 339.25262:60739 340.25595:8820 390.26025:2318 398.32373:798019 399.32907:166331

m-Toluamide	3.808	136.0757	[M+NH4]+	136.07568	55.93539:254263 67.05525:24679 70.60439:24738 72.93777:80880 73.94594:19405 81.07034:192480 89.94066:19748 90.94894:90691 91.05516:1693539 92.058:107307 95.08618:230630 107.04961:41080 107.95131:38232 109.0111:21347 109.06509:37859 118.06657:48182 119.04968:795432 120.05264:71578 125.96094:70760 135.9447:96199 136.02155:335474 136.03946:40392 136.06204:641970 136.07559:971602 137.04575:34575 137.07922:42243 137.10815:29322 137.13252:83011
LPE(P-18:1)	10.338	462.29776	[M-H2O-H]-	462.29846	52.38826:1368 67.54738:1494 69.99281:3103 69.99615:2088 74.02327:1577 78.9577:160618 84.55183:1464 93.13617:1441 96.96731:1922 97.86462:1439 98.94786:1449 99.88708:1389 107.93139:1474 110.98424:27844 122.98404:73325 150.9227:1661 168.04155:43185 180.87256:1586 182.05711:135590 186.52734:1565 283.26309:13094 297.38943:1765 329.78391:2412 359.19818:2539 373.21921:6062 391.22815:16121 403.22617:16408 404.2265:2028 430.27206:29088 462.30307:422950 463.30542:55653
Patulin	6.274	153.0155	[M-H]-	153.01932	54.39974:1380 67.02861:1418 68.0126:2883 68.99668:4359 69.03311:2298 71.01184:1416 81.03255:2006 82.02823:12838 83.01215:1587 83.04874:7430 83.99129:1259 85.02799:3139 87.04353:40626 88.04634:1528 91.02864:1924 97.02804:1430 102.98733:1604 108.98663:2143 109.02786:15575 109.03953:2734 109.06437:6274 109.99937:1950 110.02345:4490 110.05958:2845 111.01854:5320 111.04398:12119 112.03843:2984 112.98355:1604 114.69069:1338 125.0219:3086 125.03516:1805 125.05904:1456 126.01822:5712 126.05445:7014 127.036:2301 135.01874:3215 153.01578:170758 153.05557:18365 154.01338:5371 154.04964:9847
PC(18:0/20:4)	13.381	810.59943	[M+H]+	810.60071	57.0344:11263 58.06327:36197 58.06662:156098 59.05001:37627 59.07455:44879 60.08159:791986 67.05519:10337 69.07064:10366 71.07364:202089 71.08617:12433 81.07022:17384 85.10187:14610 86.09708:3526966 87.09519:15674 87.1004:143958 93.07059:13195 95.08605:13943 97.1014:12039 98.98439:113623 104.10724:328337 105.11085:12475 124.99963:1588116 126.00373:19277 143.01111:16230

					146.98141:258280 147.98541:12793 184.07417:35287324 185.07805:1434983 186.07767:220574 225.48891:10691 506.35776:28389 512.81635:11183 524.37451:33895 526.32733:10795 605.54401:35916 626.5235:16320 627.52612:92206 628.53625:9757 751.50732:64637 810.60028:1047124 811.60773:358249
Ectoine	1.15	165.06374	[M+Na]+	165.06342	102.05582:7330 105.00372:6232 120.08134:14249 121.06452:4399 164.92018:108637 165.06306:432711 165.10124:8273 165.13948:5381 166.04691:91578 166.0672:19356 166.08339:127436
3-Hydroxy-2-[[3-(3-hydroxy-6-methyloctanoyl)oxy-8-methylnonanoyl]amino]propanoic acid	11.865	449.32556	[M+H]+	449.32211	57.07082:3129 67.05524:7947 69.0707:2151 70.53242:2290 74.09693:6742 81.0703:2618 83.08641:2089 85.06553:31119 93.07068:2456 95.05009:2776 95.08614:6609 97.10149:2298 107.08637:15434 109.10182:5967 113.06061:21573 115.07552:1713 119.08543:2553 121.10115:8657 123.08038:2335 123.11665:1808 125.09659:3158 131.08505:2013 133.10078:3430 135.11717:5630 141.09238:1609 143.08588:10234 143.10684:16105 145.10187:64024 146.10506:6712 147.11707:25488 149.13278:1731 157.09998:11628 159.11629:33942 161.1321:3552 171.11771:9446 173.13289:10768 174.98872:1558 183.11658:2205 184.07446:3019 185.13303:2953 189.16348:1653 195.13704:2386 197.13255:3432 199.14816:2616 201.16237:1866 211.14716:3296 213.16348:2557 217.15936:1983 225.16191:3130 253.19524:146522 254.19879:20604 271.20554:600659 272.20801:82609 283.24069:3357 289.21545:230654 290.22086:31199 337.02301:2545 373.27274:20442 377.28543:2456 393.08331:3135 395.29538:3359 396.29608:1843 413.30829:24081 414.30612:7482 431.3125:94030 432.31607:24762 449.32425:120634 450.3313:26022
2-Thiocytidine	5.157	260.07025	[M+H]+	260.06998	55.01875:1988 57.03445:8952 69.03424:1505 73.02883:2396 84.08147:1659 85.02902:6900 97.02894:2453 111.00147:2325 112.05035:1391 115.03925:3404 121.35307:1404 128.02806:1032846 129.03281:28929 130.02428:14158 133.04982:9038 189.03278:2177 193.12587:1397 200.07092:1388 207.0439:2373 208.88416:8854 213.8885:2430 214.10524:1836 217.10172:2622 224.8802:1900 231.90018:9961

					236.87973:8439 241.88493:1731 254.91583:2023 259.8938:3304 260.07269:1894 260.14828:2224 261.09:6283 261.13016:169928
Betaine	1.317	118.08674	[M+H]+	118.08628	58.06583:14031314 59.07375:18614354 60.07719:265890 69.79829:231179 72.05669:230033 72.08163:310244 76.03982:290680 101.03486:109371 103.10419:66536 118.08604:92037464 119.09053:2594941
(4-formyl-2-methoxyphenyl)oxidanesulfonic acid	7.878	230.99617	[M-H]-	230.99692	61.24049:1413 70.57959:2037 75.492:1416 79.95578:3018 80.96336:2325 85.94473:1440 99.92396:9357 100.93193:1589 112.40253:1357 115.91861:10870 116.92685:6659 136.01514:84696 137.01813:2841 143.95163:2079 144.92133:2870 151.03876:532856 152.04185:16931 175.94125:11653 186.93382:2607 187.94177:12844 218.92296:1417 230.99481:71661 231.02916:13947 231.08803:1696 231.99214:17229
PE(16:0/16:1)	12.498	690.50745	[M+H]+	690.50677	55.05523:140595 57.03444:19039 57.07093:520250 59.05006:32143 67.05534:300970 69.07081:391484 69.09356:15799 69.09737:26624 70.07397:12642 71.04985:25718 71.08633:474880 72.08976:11277 79.05467:57678 81.07041:482754 82.07364:23837 83.0865:438343 84.08949:23059 85.06545:30695 85.10201:307610 93.07086:111711 95.08627:585664 96.08936:37777 97.10162:288150 98.10469:21402 99.08049:12853 99.11698:20703 104.10735:13011 107.08643:83066 109.10193:306915 110.10517:12227 111.08135:22998 111.11704:134753 121.10134:234343 122.10455:23014 123.1168:224492 125.1324:30035 135.11725:167908 136.12073:8353 137.13257:114669 139.14809:9079 149.13277:85303 151.14853:38138 153.12694:23533 155.01137:11929 163.14886:30172 165.16357:13221 184.07486:44635 211.20532:11717 219.21014:66134 221.22484:12926 237.22235:183818 238.22351:27234 239.23848:134414 240.24136:20321 280.26276:159608 281.26633:23574 282.28159:57373 308.29355:8846 311.25742:69207 312.25879:9459 313.27176:72520 548.47565:36661 549.48828:4024710 550.49121:987729

Arachidonic acid	12.694	305.24683	[M+H]+	305.24747	55.05524:64030 57.07088:112372 67.05531:117925 69.07075:119980 69.68121:16636 71.04986:18007 71.08629:78850 79.05463:132383 81.07035:171330 83.04979:19227 83.08649:81644 85.06546:15362 85.10194:26293 87.0444:8362 89.06052:9616 91.05512:137509 92.05798:6690 93.07074:259423 94.07333:5309 95.08618:118502 97.10159:31386 99.04497:6793 101.05991:16693 105.07013:255783 106.0731:7631 107.0864:143408 109.06507:23013 109.1018:57407 111.08025:5187 117.07038:61443 119.08553:307702 120.08881:21313 121.06449:19134 121.10117:162026 122.10457:5550 123.11664:25597 127.07574:9396 129.07033:41586 131.08513:123410 132.08858:9673 133.10085:270431 134.10402:24500 135.07993:25373 135.11722:72398 137.13249:16211 143.08601:51796 145.10216:42745 147.0798:65749 147.11702:202197 148.12051:20442 149.09651:69209 149.13277:18440 151.14795:7984 153.09102:15152 157.10077:64998 159.11627:16322 161.09535:170696 161.132:59864 162.10054:8452 163.14888:24028 165.09114:23835 165.16359:4971 167.10715:24028 171.11742:65741 172.11986:7096 173.13095:6855 175.11177:196129 175.14688:25677 176.11598:7099 177.1649:34262 179.10611:127456 180.11058:9393 181.12352:8694 185.13301:25723 189.12665:63383 189.16347:9093 190.13196:5111 191.18057:30699 193.1232:204176 194.12497:19363 199.14815:9031 203.14417:9964 203.1797:35229 205.19437:23072 207.13722:115542 208.14168:8421 221.15309:76042 235.17052:21314 243.21054:25911 245.2265:79572 246.22765:9302 249.18556:6326 259.2037:22932 259.24329:14038 269.22614:38546 287.23807:113609 288.24289:18122 305.15561:194522 305.24634:566699 306.15875:25645 306.24979:97520
Mmv062221	7.455	280.15408	[M+H]+	280.15567	53.72789:1816 57.03444:3275 57.07082:2947 60.08163:135012 61.08515:2886 69.85365:5762 71.04986:1909 73.02882:2150 82.39403:1762 84.12681:1851 85.02905:1066342 86.03278:39917 87.04517:7168 91.05505:63688 91.35252:1833 103.0397:1788 137.05942:2224 144.10255:38243 145.08536:2663 146.05991:49829 147.06447:6139 148.82234:1869 150.02805:3362 175.07436:1993 221.0816:119920

					222.08612:16025 234.03566:3459 234.96201:2169 252.04143:2138 257.96924:2619 280.15582:600669 281.15921:82452 283.03284:1901 283.06897:1861
2,4,7,9-Tetramethyl-5-Decyne-4,7-Diol Ethoxylate	9.316	332.28024	[M+H]+	332.27951	55.05515:6500 56.0502:3151 57.07083:1930 58.06584:3464 60.0455:173055 61.0485:2441 67.0554:15825 69.07071:12379 70.06571:40715 71.01466:2584 71.04986:1879 81.07042:46805 84.04491:1861 85.06547:3269 88.07639:33932 93.07068:8756 95.08633:54667 97.10149:9337 107.08638:6270 109.10198:24738 111.11691:3246 119.08543:2625 121.10115:6590 123.11674:11871 135.11717:8171 136.07558:3506 137.13251:2509 138.05553:1744 147.11693:5483 148.07608:2150 149.13278:2575 161.13403:2155 162.09273:2853 172.07706:1840 174.09239:10398 188.07022:5276 190.08723:5691 191.08279:10445 215.11913:8500 216.10431:11020 231.11253:6176 233.1283:1912 233.22623:2255 243.11346:6283 246.24232:2556 250.25223:6647 252.23131:3287 255.11281:2525 256.09723:1867 261.22208:2295 266.24664:22201 267.25067:2313 269.12927:6941 270.24234:7799 278.24857:88622 279.25342:12628 283.10965:2393 284.26068:7738 285.24048:2196 287.14145:7480 291.13275:3202 296.25937:109648 297.12704:2792 297.26419:16338 314.26779:134671 315.13617:20359 315.27399:18844 332.27951:312335 333.1488:49602 333.28027:54720
[3-heptadecanoyloxy-2-[-octadeca-9,12-dienoyl]oxypropyl] 2-(trimethylazaniumyl)ethyl	13.337	830.59161	[M-H2O-H]-	830.59003	70.00503:19351 78.95729:160103 152.99419:18432 168.04184:290640 224.06877:144062 279.23257:6325436 280.23535:615548 281.24817:2264870 282.25296:222197 283.26303:2186266 284.26526:182594 307.26443:43999 419.25531:12238 486.29572:14786 490.328:32721 506.31985:51448 508.33481:199279 509.33725:34207 770.57867:2554673 771.57336:584577 830.59735:13655

phosphate					
2-(acetylamino)-N-{[N-(2,6-dimethylphenyl)carbamoyl]cyclohexyl}-N-(2-indol-3-ylethyl)acetamide	9.126	533.27844	[M-H]-	533.27692	69.23427:3040 69.23809:2710 73.36089:1412 89.27113:1370 93.80985:1476 96.95837:31009 141.05894:1424 222.71982:1667 266.2627:1506 317.50519:1441 407.16376:1926 533.27313:697408 534.27936:109888
Trans-Zeatin	6.439	254.07945	[M-H]-	254.08141	67.29797:1236 68.04877:1772 68.24887:11872 68.25262:3150 71.01182:2389 71.91192:1375 75.59168:1306 83.19176:1323 88.03861:28327 88.98623:1639 94.02827:2188 95.03157:2272 96.83884:1964 97.76095:1305 112.03841:3273 117.88854:1330 118.91496:1379 124.03864:9124 125.04309:6288 134.02283:1955 136.99387:1235 138.0554:1207 142.0493:7431 146.08116:11387 177.44746:1553 186.04024:8433 186.07634:3225 187.10858:3066 208.96835:10691 218.10202:56636 219.10753:5733 224.11342:1783 254.12195:25766 255.12383:1623 255.16248:1566 255.23595:1591
Medicanine	1.38	160.09695	[M+H]+	160.09737	55.05505:48401 58.06624:8249 60.08159:128170 61.04079:10545 68.79278:49285 69.03422:11065 69.07068:9833 70.06618:30682 71.04984:10753 71.06916:12890 72.08163:7335 81.05851:8468 83.04977:40487 84.0815:49391 86.09712:14773 88.07632:8011 96.08127:64938 97.02891:10287 100.07661:29008 101.05987:193245 102.05579:10298 105.07004:8867 113.96425:75670 114.05556:32839 114.08327:32878

					114.09137:4842495 115.09528:246397 118.06532:41457 124.05872:15175 130.08725:12417 131.97488:35891 132.10152:12057 133.03523:24913 142.07024:11482 142.08644:76015 151.04576:10970 160.03583:41115 160.09724:4564081 160.1335:558713 161.07193:8682 161.10127:236603 161.13593:35867
Piragliatin	8.908	420.07855	[M-H]-	420.07901	70.78762:5621 72.11596:4927 79.95574:95389 80.96342:25666 107.04884:198102 108.0517:7237 110.78593:4571 187.00668:2391788 188.01009:83890 229.9265:4406 232.92155:5716 271.04874:4498
3,6-Dimethylmangostin	6.436	437.21326	[2M-H]-	437.20999	51.15186:1457 68.6516:11518 71.0126:10922 71.04872:1741 88.03865:181241 89.04198:2385 146.08005:62712 174.95415:3352 218.10205:381736 219.10461:18452 266.89844:1634 270.0062:14212 271.01056:2297 272.0202:18879 301.99997:2037 302.05472:3348 304.01096:7573 328.14279:1634 394.15158:2060
Benzofuran	3.807	119.04947	[M+H]+	119.04969	55.93536:4452 56.94319:129235 65.0397:32690 70.19209:9527 72.08107:6114 72.93791:182897 73.0845:16962 73.9381:6422 74.06065:16416 74.95362:55555 76.40462:4949 79.05463:7581 90.94827:256939 91.05531:562307 92.05799:29619 93.07068:8816 97.00809:5425 103.05459:10915 104.06206:8426 108.95852:45509 113.96467:33936 117.07034:28739 118.94261:56399 119.03614:5335 119.04996:197578 119.05955:21609 119.08544:261745 120.02394:6913 120.0439:18399 120.05605:38810 120.08133:10412 120.08881:22691
SM(d18:1/16:1)	12.828	745.55115	[M-H]-	745.55011	70.47908:3570 78.95767:350271 96.96822:6485 122.984:12370 168.04144:446763 169.04607:8760 181.75714:2206 255.23212:8476 256.23672:3665 280.23553:2681 281.24762:33529 282.2525:21988 283.26315:9659 284.26508:3318 447.30197:3119 685.52228:870972 686.52905:157768 745.54254:9921
Isoleucylhydroxyproline	6.26	245.1498	[M+H]+	245.15013	68.05045:79044 68.73753:80219 69.07068:196894 70.06564:16318 84.08146:71162 86.06077:560070 86.09715:6068766 87.06366:15135 87.09526:22460 87.10056:254750 114.05558:27580 132.06561:8501753 133.06888:357724 135.14845:10578 157.72067:11231 160.07619:16259 200.12729:27924 228.12285:16827 245.1503:1167647

					246.1324:23043 246.15437:75111
2-decyl-3-hydroxypentanedioic acid	13.472	311.18146	[M+2H]2+	311.18292	54.66439:20610 57.07088:291012 61.69002:14963 67.05525:42407 69.0707:43655 71.04021:35037 71.08626:189581 73.02883:32704 78.80988:18356 81.0703:86741 83.08642:38284 84.96065:68222 85.10188:153718 86.09715:80058 89.06053:74339 95.08625:134079 97.10149:69663 104.10769:156996 109.10182:42920 111.11803:25674 123.11794:25718 129.74461:16517 157.01671:18928 157.53674:60661 166.54314:34575 169.40471:17875 178.87219:16580 181.02417:20948 184.07446:102729 185.53265:21042 196.24828:17517 201.89769:17869 219.90762:67389 229.89114:23777 237.91698:48824 240.10027:296783 241.1019:17490 247.90253:201165 252.95473:21666 256.00397:75699 256.02341:119519 258.00079:17530 264.91254:17173 265.91299:229402 270.922:18362 271.98239:39979 274.03595:18726 314.03064:260773
PFSA-H	6.272	230.95897	[M-H]-	230.95561	69.54787:5633 84.93733:8629 85.02798:4769 85.94479:4816 87.92367:31849 88.98624:3153 99.92426:132273 100.93233:27351 103.91866:41005 104.92642:9513 105.93363:3673 106.94178:2989 115.91915:119660 116.89816:9607 116.92709:103418 123.94434:17812 128.92682:7949 129.93507:21038 130.94255:12754 130.97809:12482 140.96367:22860 141.93538:10039 142.94308:10608 142.97884:272695 143.95032:39259 143.98106:14439 144.92163:35011 147.94467:4071 156.9572:15559 157.92981:22496 159.93251:3520 174.96944:34349 175.94162:93510 181.89667:3474 184.95255:20913 186.93512:57111 186.96794:1161200 187.9417:127155 187.97057:70198 202.96445:20717 218.92238:20897 230.93501:5180 230.95815:68246 231.00826:4721
Glycodeoxycholic acid	10.18	448.30585	[M-H]-	448.30685	69.09837:9597 74.02322:562329 75.02634:6629 152.99409:3875 386.30634:23122 387.31006:2048 404.31873:10279 430.29697:3043 448.30829:1833082 449.30942:278169

Chenodeoxyglycocholic acid	10.172	450.3212	[M+H]+	450.32138	55.04663:2153 71.02034:3428 76.03984:33647 79.53951:2242 81.0703:4671 85.06548:3008 95.08614:4253 100.34686:2770 105.07008:3756 105.23882:2420 107.08639:3148 119.08543:4500 121.10116:8779 126.90475:2298 131.08507:2146 133.1008:2865 135.11719:3191 145.10031:2625 147.11694:8325 149.1328:3339 158.08174:15673 159.1163:3123 161.13211:11844 162.39064:2208 173.13315:2978 175.14912:8753 177.12669:4337 184.07448:2617 187.149:3200 189.16351:2539 201.1651:5133 211.14717:2667 215.179:4664 217.15938:2565 225.16193:3055 227.18135:2403 228.97818:2233 238.14339:2888 239.17844:2887 321.25668:25749 339.26562:30377 414.29861:75245 415.29968:4310 432.29956:3085
PC(15:0/20:4)	12.665	826.55878	[M-H]-	826.55988	59.01227:14357 69.00749:8045 69.24024:7585 69.24352:2309 78.95716:23443 83.02356:3109 152.99409:2766 168.04187:36002 205.19562:9351 224.06915:34995 255.23198:357428 256.23688:27199 259.24222:39893 277.21658:9210 279.23248:217226 280.23569:18440 281.24805:4186 285.22186:2765 303.23297:598713 304.23752:53845 305.24625:2447 376.91849:1928 462.30286:4149 480.30951:42063 481.30963:3610 504.31607:3472 510.2977:2392 512.51343:1889 528.30872:2748 766.53711:339597 767.54401:86233
beta-D-Glucopyranosiduronic acid, 4-methylphenyl	8.178	283.08209	[M-H]-	283.08231	55.01723:8676 57.033:91388 59.01241:504975 67.01715:17016 68.99667:7672 69.22117:5102 71.01243:211767 72.99174:65958 73.02771:35114 75.00732:414427 83.01206:30362 85.02797:934277 86.03099:24919 87.00716:178903 89.02283:114761 95.01225:280973 96.01532:5352 99.00764:391060 100.01028:7584 101.02246:21708 103.00215:120812 107.04877:1065012 108.05164:50218 111.00746:34447 113.02333:1669632 114.02599:62053 115.00224:105623 117.01804:231993 129.01845:155954 129.05324:8222 131.07054:5991 139.00203:30755 147.02901:6269 157.013:72674 163.06035:9201 165.91743:5117 175.02423:264761 176.02795:6041 239.96889:6258 265.07043:51646 266.07739:4974 283.08224:556370 283.1908:7261 283.96075:16259 284.08801:49137

Epoprostenol	11.405	351.21701	[2M-H]-	351.21771	57.03291:2562 57.85544:1268 59.01212:11949 69.03287:14245 71.01807:2220 71.89515:1311 73.02771:2440 83.04873:1280 85.02798:1760 89.45702:1407 95.04827:1917 97.02802:8876 100.05583:1254 103.81401:1219 106.51405:1392 107.08456:1779 109.06435:7186 119.08487:2056 121.10097:13674 123.07991:2292 125.09616:2190 127.07401:5046 127.1107:2604 137.49863:1271 139.07487:4771 139.1106:9562 148.97401:1489 149.09482:6856 151.11104:3045 153.08937:1998 155.07018:2306 163.11261:14100 165.09157:17083 165.12772:5000 167.10551:1705 174.1449:1381 180.43501:1171 183.10086:1991 195.10193:7732 206.13007:1455 207.10187:2331 207.55772:1362 225.11276:1452 228.46527:1494 333.20703:1556 351.21841:52062 352.21857:6894
PI(18:0/20:4)	12.304	885.547	[M-H]-	885.5498	67.76589:23192 72.99103:3514 75.00668:3876 78.95734:164494 96.84336:7350 96.96764:42197 112.98353:3996 114.9322:2379 115.91898:3718 116.92722:30682 134.98302:8341 152.99457:161776 158.58104:2248 205.19576:11408 223.00102:81695 231.2115:2550 241.01244:379769 242.0162:10683 259.02121:34696 259.24277:32959 260.24728:2751 279.23267:3767 281.24377:2685 283.26309:575547 284.26514:53298 285.22205:3443 297.03918:43649 303.233:367535 304.23676:44063 315.04581:25158 419.25449:103041 420.25861:12594 437.26343:8326 439.23074:13191 581.30627:85212 582.31763:12503 599.32477:24726 601.28162:21434 619.2804:3158 885.56079:865374 886.56073:242113
LPC(15:0)	12.021	482.32516	[M+H]+	482.32407	57.07085:242335 58.06666:225141 60.08158:1380856 71.0737:191372 71.08614:221656 81.07027:50301 85.10191:138311 86.09708:4349120 87.10064:51129 95.08611:141927 98.98515:48262 104.10733:20478138 105.11082:546400 124.99969:1571697 163.01651:145027 181.02643:52745 184.07428:25635550 185.07832:624047 199.03621:50091 258.11053:375178 299.25616:806191 300.26031:54174 405.24374:148453 464.31348:2042502 465.31927:205765 482.32822:6744968 483.32339:852500

methyl 4-[(3,4,5-trimethoxyphenyl)carbonylamino]benzoate	8.108	344.11179	[M-H]-	344.11392	68.14953:5035 68.1538:11488 74.02283:249483 91.05338:11377 96.84067:5867 103.91844:8202 129.05466:289153 130.05717:12585 139.99567:6752 183.98531:7210 188.01003:1852 192.06462:218244 193.06805:14685 214.04686:10402 226.07014:11573 232.04578:10418 260.04041:2959 276.02121:3291 276.04733:17477 277.05054:1989 281.96542:2850 344.11084:82262 345.11188:5002
Cinnamoylglycine	8.95	204.0656	[M-H]-	204.06662	69.11098:7145 75.00731:2151 79.95585:2081 82.02819:6791 98.90496:2341 106.0392:29117 107.0352:2111 107.04886:2175 116.04932:1716 117.01213:2242 117.06976:13856 119.04794:2088 123.04369:6786 124.04664:9264 128.04967:2940 130.06456:36292 131.06772:1890 132.07971:1830 133.06416:1324 142.0654:1745 143.79953:1474 147.04427:3084 157.99933:2778 158.05923:16437 158.9948:2172 160.07468:134245 161.0023:12378 161.07805:11871 174.05562:6313 176.97095:5282 178.06114:1986 186.05473:6501 197.60976:1613 203.93947:10429 204.00592:9171 204.06587:68775 204.94841:9203 204.99124:10654 205.06763:7749
3-Aminopicolinaldehyde	3.83	123.05556	[M+H-H2O]+	123.05529	50.0252:16684 53.03948:28047 67.05473:17398 68.05045:16907 70.6117:34346 78.03466:24337 79.05531:22819 80.04993:1346707 81.05369:20247 82.03841:16891 95.05009:817643 95.06152:64199 96.04485:603311 105.04556:24690 106.02974:126692 112.04022:29901 123.05579:9018879 123.09204:104192 124.03918:905078 124.05887:335111 124.07579:23562
LPC(14:0)	11.53	468.3096	[M+H]+	468.30847	57.07077:625635 58.06666:329770 60.08155:2648910 69.07068:121195 71.0737:374715 71.08614:589290 81.07027:108734 85.10191:260943 86.0971:8405111 87.10064:213208 95.08611:314830 104.10735:43565520 105.1077:207647 105.11076:1427593 109.10179:77248 123.11662:80482 124.99965:2732218 163.01651:244882 181.02643:93965 184.07436:53402120 185.07791:1538177 211.20531:126113 225.82339:109042 258.1105:790697 285.24039:1977889 286.2482:215510 391.22717:360675 450.30109:3697281 451.298:522699 468.30594:13263117

					469.31622:1791143
(2E)-N-[4-(acetylamino)phenyl]-3-(3,4-dimethoxyphenyl)prop-2-enamide	8.114	385.1395	[2M-H]-	385.1405	69.25998:6723 74.02325:390318 96.84067:1695 108.15617:1557 192.06464:289290 193.06853:18386
Berberine	8.905	336.12335	[M]+	336.12399	69.86249:11363 185.04939:2960 208.06761:2780 249.00653:3017 272.02911:3608 272.81378:2727 275.09299:10629 276.09976:2868 278.08142:10525 290.03806:4568 291.09027:6010 292.09604:303268 293.09781:42463 304.0961:64602 305.09961:10990 306.0777:105093 307.08011:23903 320.09085:363098 321.09811:819008 322.1044:128846 336.12125:3253674 337.12845:569460
Acetylcarnitine	1.41	204.12363	[M+H]+	204.12299	57.03444:50023 58.06625:44668 60.0816:4512444 61.08469:35653 68.87562:220376 70.06564:134962 71.06178:36673 71.06917:43399 76.03982:54209 84.0449:120887 85.02897:24345986 86.03268:742497 88.0873:119502 116.07123:161901 117.07513:133016 126.05482:104682 130.05069:143527 138.05551:43670 144.06482:37648 144.10234:920008 145.05043:4056377 146.05313:186513 159.13148:119536 187.10768:37826 204.12241:11468843 204.15034:523407 205.12732:814603
PE(18:1/18:1)	12.472	744.55426	[M+H]+	744.55377	55.05516:42496 57.03442:28070 57.07084:130196 58.06664:51239 59.05008:68298 59.07414:13080 60.0816:233152 67.05521:158742 67.54954:16454 67.55375:84272 69.07069:112713 71.07355:51976 71.08626:133195 79.05523:40852 81.07027:239233 83.0864:133070 85.10191:82102 86.09711:1026794 87.10027:37104 91.05509:17667

					93.0705:46839 95.08611:235515 96.0894:10801 97.10139:73520 98.98512:31048 99.08139:11419 104.10728:136200 105.07103:11153 107.08607:35899 109.10174:149598 111.11703:42511 117.0919:27735 119.08535:11637 121.10098:47331 123.11661:76461 124.99965:484595 133.10071:18870 135.11662:49539 137.13243:26822 149.13269:26049 161.13199:9298 163.14879:9186 184.07417:12178996 185.07802:460490 186.07745:71550 245.22653:7892 263.2355:37816 265.25214:21175 267.26706:21197 306.28009:42152 308.29343:10128 310.31061:8565 337.2753:18403 341.30588:24540 504.34296:31353 603.52716:1116513 604.54071:333925 744.58051:286808 745.58307:110762
5-Hydroxyindole acetate	6.306	190.05003	[M-H]-	190.05092	58.95737:11930 68.28365:8505 68.28793:13978 87.0072:5090 87.92344:12828 92.04871:20068 96.83794:4654 96.84337:4055 101.05907:5200 101.95058:23979 102.94682:266585 103.91843:2934 104.92641:2818 111.00773:19798 116.04937:21009 118.06483:249291 119.06763:11178 120.04337:9744 128.0497:134987 129.05325:14426 131.03644:204391 132.0394:12012 144.04353:1333203 145.04742:101953 145.94113:15587 146.05986:368823 146.93761:85474 147.06335:26920 162.89262:2958 164.03435:5004 172.039:179484 173.04276:16577 176.01025:5177 190.01369:3438 190.05078:60738 191.0188:2640 191.05385:9854
Monopalmitolein	12.011	311.25845	[M+H-H2O]+	311.25699	55.01913:1954 55.05521:48537 56.05815:1874 57.0345:51263 57.07084:66657 59.05006:2932 67.05524:66448 68.05843:1853 69.07072:113039 71.04986:8265 71.08632:28143 73.02946:46960 73.06554:2065 76.03989:28968 79.05527:23096 81.07034:95937 82.07365:5505 83.04979:3630 83.08643:101827 84.0895:2428 85.06548:12193 85.10213:14162 86.09714:3809 87.0444:1815 89.06055:56088 91.05514:8863 93.07072:46548 94.07333:1864 95.0862:90331 96.08945:3407 96.84246:3675 97.06519:22191 97.10154:71384 98.1047:2055 99.08144:9498 104.10737:6303 105.07006:3279 107.0706:1748 107.08631:38959 109.10191:42896 111.04473:2514 111.08026:18352 111.11711:24274 113.06054:2229 113.09589:2292

					115.07552:2177 117.07033:1820 119.08541:7713 121.1012:81393 122.10457:2601 123.11671:22868 125.09664:12985 125.13242:3389 127.11243:2305 131.08647:2807 133.08626:25499 133.10066:13075 135.11714:61699 136.12074:2692 137.09595:3449 137.13246:16862 139.1123:10229 141.12735:2440 145.10028:3433 147.11691:6886 149.0965:2260 149.13277:32388 151.14795:3347 153.0551:7630 153.12695:2912 157.10069:2134 159.11627:3252 161.13208:6044 163.14909:12355 167.14403:1870 173.13312:3637 175.14908:2315 177.11325:3034 177.16467:3068 181.04958:6457 184.07445:9090 187.14653:1834 199.14815:3152 209.11629:111109 210.12079:8791 215.17896:10139 219.21039:21840 225.73013:2532 229.19518:2446 233.18906:7070 237.1116:83104 237.22252:24730 238.11546:9637 239.17839:3351 240.10019:3106 247.20528:2624 250.06084:1634 251.20107:8684 255.12492:20118 257.18903:3234 267.14294:1990 275.20117:30218 276.20428:2822 283.1774:2446 293.17395:7153 293.21204:11950 311.18356:8449 311.25656:310194 311.63702:1843 312.25919:66392 312.32687:8420 312.36353:9078
Myrsinone	11.79	293.1759	[M-H2O-H]-	293.17529	61.74175:1341 70.18536:1929 74.60779:1385 78.95696:7285 96.95856:31218 99.00669:6789 106.03916:1789 114.2481:1416 126.55389:1384 136.08768:31713 156.13051:1472 192.11508:44594 193.15744:269248 193.74689:1450 194.16183:23291 196.03833:1697 221.15504:2965 232.73616:1369 236.10614:3256 249.18475:117358 250.18843:9437 288.24283:1416 293.1788:253662 293.6489:13744 294.18076:20711
Imidazolepropionic acid	11.213	141.0659	[M+H]+	141.06641	52.59969:4477 63.22873:4606 68.05046:21957 69.52426:4521 69.86362:12168 81.04547:129348 82.05326:57810 83.06129:6804 95.06072:235864 96.04482:5110 96.06446:10060 96.08144:5260 104.84776:4451 108.44445:4650 111.05588:366256 112.05936:21156 113.96323:5657 114.09142:10322 116.97191:5544 118.9672:51361 123.05585:501315 124.05876:29533 141.0654:434339 141.9836:9010 142.04941:7864 142.06868:26801 142.08635:30319 142.12169:6414

Cortisol	9.589	363.21661	[M+H]+	363.21658	71.01522:2084 79.05463:3060 81.07029:9001 83.04978:10679 91.05515:5294 93.07068:6821 95.08613:9927 97.06531:56289 98.06877:2625 99.04497:7030 105.07006:8593 107.08637:10273 109.06523:29548 111.08025:2770 117.07034:2526 119.08543:19768 121.06461:193427 121.10114:7295 122.06839:13838 123.08041:27035 124.08495:2131 129.07025:5830 131.08534:13155 133.06438:2566 133.10217:12040 135.08005:23577 135.11716:3378 137.09595:3454 141.07011:6282 143.08592:17029 145.06548:3449 145.10191:25320 147.08125:13694 147.11702:25998 149.0965:8742 149.13277:3137 155.08646:9241 157.10071:20173 159.08011:9807 159.11638:18347 161.09555:15681 161.13208:6645 163.11136:20611 169.10204:19012 171.08162:6738 171.1178:16960 173.09621:15645 173.13312:9485 175.11148:18860 177.12666:3469 181.10272:6842 183.1165:17314 185.09718:8274 185.13318:14056 187.11261:28493 189.12666:11137 195.11635:10398 197.09579:3326 197.13254:7057 199.11351:6359 199.14815:2590 201.12718:9355 203.10841:2009 205.1219:5390 207.11736:5860 209.13297:13062 211.11223:3520 211.14714:7510 213.12851:13206 215.14305:6936 219.13643:1993 221.13438:3353 223.11238:2606 223.147:11044 225.12682:18558 225.1651:5748 226.13274:2673 227.14235:7609 229.15898:3110 231.13918:3532 233.13165:2255 237.1254:2632 239.14334:17712 240.14607:1970 241.15842:29226 242.16345:3044 243.13861:2844 243.17458:10071 245.15384:1998 249.16289:14719 251.1451:15535 251.17841:7441 252.14394:2256 253.15706:5337 263.14258:5459 263.17905:3376 265.15805:6126 267.14294:2850 267.17581:52674 268.1817:14407 269.15857:3915 269.1918:42272 270.19177:5679 271.16742:3469 273.16461:2599 279.17624:12556 281.15454:7252 281.19034:19723 282.19025:3007 285.18582:11590 286.18857:2036 287.20145:6518 291.1748:20550 292.17673:3490 297.18555:34040 298.18881:3270 299.20227:7719 309.18369:64124 310.18567:12404 315.19507:10459 327.19696:90475 328.19992:17969 345.20822:45880 346.20816:8086 363.21414:307385 364.22009:77260

PI(16:0/18:2)	13.46	833.51654	[M-H]-	833.51849	75.00668:3386 78.95741:115340 96.84246:15118 96.9678:22865 134.98302:2864 146.95972:2541 152.99458:109901 158.97765:3309 168.04198:23661 174.95447:18843 223.00102:55106 224.06891:10908 225.07114:2791 225.59727:2993 241.01239:249380 242.01265:4689 253.21762:4189 255.23213:381301 256.23657:30353 259.02203:27800 279.2327:435985 280.23532:76023 281.24857:373130 282.25302:233380 283.26361:145046 284.26556:95922 285.26776:9274 297.04004:27287 315.04623:9708 391.22153:68328 392.22934:3314 409.23669:2827 415.22186:7735 508.34665:7907 509.34824:9015 553.27698:49074 554.27844:8463 571.2948:9838 577.27283:7554 772.57867:15957 773.58868:240287 774.59753:22822 796.06781:2507 833.51172:580366 834.51654:153511
Octamethylene diamine	13.431	145.17012	[M+H]+	145.16988	58.40471:9381 59.63844:9966 61.48924:10366 62.92982:103041 69.39963:24112 69.40347:38555 72.08167:11667 80.94032:16616 84.95992:14496 100.07666:24914 100.11275:133486 103.95644:63420 118.96721:11372 120.96478:12434 121.96647:720845 122.96522:53910 125.32649:10736 129.96356:10349 144.98248:124182 146.05988:19713
methyl 2-{2-[9-(4-methylphenyl)-6-oxohydropurin-8-ylthio]acetylamino}benzoate	8.907	448.11005	[M-H]-	448.10852	59.01194:3168 65.37255:1967 69.32069:4067 69.50664:2124 71.01241:2568 79.95584:72666 80.96358:27033 85.0281:15247 87.0072:2526 95.01226:3124 99.00671:3901 107.04887:158654 108.05168:3666 113.02226:37200 117.01812:2183 151.03888:60344 175.0244:13920 187.00667:1525042 188.01007:73577 188.99925:1837 258.9343:2512 260.92957:2500 273.0784:17907 289.0289:2901 368.9884:7908 449.11011:33558

Camalexin	0.961	199.03667	[M-H]-	199.03352	51.26761:15140 54.27703:14718 69.33876:19766 69.34205:64151 75.4274:15856 125.87209:28756 126.63362:14976 134.10971:14727 141.56403:13938 161.8409:167982 162.8374:6550182 163.83727:448483 164.83429:5936061 180.21915:16063 186.23778:17750 198.80598:443314 199.80403:10998255
2-[(3,6-dimethyl-2,4-dioxo-1,3-dihydropyrimidinyl)methyl]benzoic acid	7.661	273.09128	[M-H]-	273.08801	69.76346:6284 84.00746:17593 102.92093:1923 110.02344:7590 114.05395:136684 114.98714:1853 115.05734:3262 127.04967:15244 129.05464:2734 130.98245:10830 144.95782:7243 155.08096:21563 158.0276:95777 158.97765:25496 159.02899:5634 159.97855:3119 170.31433:1720 181.09657:5307 185.00987:18956 186.00897:8255 201.02032:2306 229.10213:52020 230.10512:2926 255.08134:1695 256.10837:3345 262.98032:1598 273.09174:108019 274.09177:8812 274.11758:5129
PE(17:0/20:4)	13.013	754.53326	[M+H-H2O]+	754.53809	55.05514:66362 56.05053:24083 57.03442:16201 57.07084:199759 59.05007:81104 60.08158:5329 67.05524:130521 69.07069:164265 71.07368:128937 71.08621:190203 72.08161:6594 79.05526:32261 81.07026:206559 82.07431:6407 83.08636:190229 85.06542:7771 85.10193:111457 86.09711:1314653 87.10046:40835 89.06048:5752 93.07048:52111 95.08609:245707 96.08941:5246 97.06515:6859 97.10147:112840 99.08139:21403 99.11694:5148 107.0863:37493 109.10175:122753 110.02168:23872 111.08131:4982 111.117:40052 115.07546:7585 117.0919:21517 120.96586:85737 121.1011:107104 122.10452:6574 123.1167:68266 125.00004:30446 125.13235:5487 127.11237:5824 135.11703:92878 137.13222:40253 146.98152:3107212 147.9852:42638 149.13327:26662 151.14789:15085 153.12689:12413 163.01646:19231 163.1488:6310 164.99251:53550 175.13362:5168 179.0083:13970 181.02638:5550 184.07428:460164 185.07799:21110 188.00879:100970 207.99905:339854 209.00148:13496 219.21017:19466 221.22787:5455 237.22186:57734 239.23755:42268 413.20438:4972 417.24066:6069 439.22589:89558 440.22369:12971 441.241:31629 476.31812:26009 478.3349:21253 498.29642:16888 547.47614:42837 548.47546:6350 549.48724:490480

					550.48987:118823 569.45197:12070 570.45856:5493 571.46735:1082397 572.4787:247953 667.42047:6505 695.46912:1026379 696.46252:304626 754.54437:1284546 755.54785:437621
Pivaloylcarnitine	6.982	246.17036	[M+H]+	246.17053	57.03444:4034 57.0708:57296 60.08164:164739 69.19334:4292 69.19772:7586 85.02904:1722899 85.06538:30367 86.03275:52544 103.07541:4699 144.10269:37582 173.09637:4461 176.96579:3758 187.09569:356769 188.09961:28859 246.16922:834723 247.13263:15867 247.17212:84150
(2E,4E)-Octadeca-2,4-dienoylcarnitine	11.002	424.34146	[M+H]+	424.34268	55.05517:63028 57.0344:30599 57.07072:21919 60.08162:314977 67.05529:33121 69.07069:169366 69.18516:14633 71.08678:11521 79.05529:4118 81.07034:57445 83.08638:104730 85.02902:2050276 86.03278:46374 86.09713:6618 91.05513:11028 93.07066:13360 95.08605:60067 97.06519:6007 97.10146:69764 103.03969:4446 105.07005:10680 107.08636:17947 109.10178:33406 111.1171:25274 119.08663:9465 121.10115:19502 123.11642:21838 133.10075:16781 135.11714:17070 137.09593:5081 137.13248:12670 139.11229:3986 144.10258:82717 147.11674:22614 149.13275:6334 151.14793:5171 157.05026:15558 161.13264:19411 163.14886:6280 165.12735:6034 167.14401:3854 175.14906:12115 181.15819:5746 189.16345:11548 213.11328:3504 241.14447:4150 245.22662:23222 253.14555:5006 255.15913:5545 263.23569:18202 269.17526:6189 321.22903:11967 323.22586:3668 339.24033:3812 343.1976:4711 365.27115:10117 424.33838:1331388 425.16006:30428 425.34341:258401
PC(18:0/18:2)	13.404	786.60266	[M+H]+	786.60071	56.05015:85974 58.06327:143949 58.06663:645595 59.07456:158201 60.0816:3297515 67.0552:66158 70.44873:81468 71.07363:749348 86.0971:13680861 87.10053:538661 95.08606:55935 98.98457:430656 104.10728:1432895 105.11086:55179 124.99967:6435288 126.00375:62261 184.07422:146566560 185.07814:5359064 186.07793:745414 502.32941:85803 504.34286:162593 506.35782:74803 524.37451:220686 603.54224:56652 683.57001:55726 786.6095:4486274 787.59521:1599760

Gibberellin A61	11.52	333.16663	[M+Na]+	333.1702	57.07082:2239 57.75654:1485 71.02544:2750 71.08623:1887 79.006:1363 89.06053:1343 95.08614:2351 98.98523:170549 100.9887:15793 116.9959:2402 140.01143:2434 165.88667:1381 176.99037:35260 178.99478:2260 180.71043:1408 199.64754:1431 207.22202:1356 217.1199:1332 234.48795:1627 244.74663:1470 254.9969:3204 276.09552:1650 277.10318:35896 278.10791:2767 296.20145:1406 311.0795:7045 333.16608:1053504 334.17181:181852
(R)-2-Hydroxycaprylic acid	10.003	159.10107	[M-H]-	159.10211	59.01237:14001 65.01309:4528 69.86913:12604 86.99238:39050 90.00764:3917 91.02863:4440 96.84246:4379 97.0642:40769 102.98734:320931 103.98753:26361 106.03918:13111 111.07957:12174 113.09525:1159709 114.09882:71458 114.98708:23756 115.07491:12244 118.03924:5643 119.0479:4223 130.98236:1104246 131.98315:117127 132.05522:17377 133.0394:10154 133.05104:16255 141.09091:34136 157.08614:22685 158.9778:70592 159.02899:15232 159.06554:71848 159.10127:1469559 159.97855:6711 160.03998:4758 160.0515:17784 160.10712:70632
beta-Alanine	1.264	90.05564	[M+H]+	90.05498	50.76519:12349 55.21202:12569 57.39978:13172 69.66302:59543 72.0451:51241 72.08166:22224 78.87629:14368 90.0557:197516
Di-N-propylamine	13.163	102.12832	[M+H]+	102.12772	56.05024:54682 57.07081:53185 58.06588:443336 59.06123:55066 59.06944:7555 59.0738:277348 60.04542:11434 60.05645:6686 60.06484:11969 60.08162:25582 72.08165:103188 73.08924:7457 74.09697:518213 75.1003:8647 84.04491:12946 84.08147:13169 85.02915:40042 87.0683:7238 102.05582:24604 102.09201:636548 102.1282:2327041 103.09528:32310 103.13184:119553
1-[[(2S,3R,11bR)-3-ethyl-9,10-dimethoxy-2,3,4,6,7,11b-	13.467	463.25867	[M+2H]2+	463.26022	67.05488:143720 69.07077:92977 69.39907:13474 69.40237:53009 71.08636:107835 79.05464:27918 81.07044:162990 81.15483:11964 83.08643:60528 85.10195:70954 89.349:10836 91.05434:49508 93.07069:71179 95.0863:216542 97.1015:65199 99.04499:10935 105.07008:47432 107.08639:59794 109.02837:14779 109.10204:100847 111.11693:14904 117.07035:20209 119.08543:62148 121.10116:64207 123.11666:53600

hexahydro-1H-benzo[a]quinolizin-2-yl]methyl]-7-methoxy-3,4-dihydro-2H-isoquinolin-6-one					131.08507:21059 133.1008:56750 135.11719:19842 136.93086:83076 137.13252:17463 147.11694:47994 154.942:225221 155.00958:19704 157.10258:11207 171.11772:18605 172.95198:298242 175.14911:14682 177.95683:17574 185.13304:11923 189.16351:17757 190.96346:92715 195.9696:45170 196.95157:20655 203.1799:67460 210.96687:72648 212.9482:15226 214.96355:58862 220.95053:27740 228.97847:180901 238.96109:60395 246.98799:22851 252.05666:12302 256.97406:72978 269.22562:62422 280.97583:55715 287.2384:63246 292.30472:13197 297.92044:12113 298.98203:318788 299.98431:17023 303.99081:13737 305.24634:24869 316.996:530027 317.99933:21397 322.00043:77092 335.00217:188127 336.00433:10892 340.01257:98186 341.30597:285694 342.30701:48384 343.19135:84235 358.01843:24974
3,4-dicatecholspermidine	6.27	418.19357	[M+H-H2O]+	418.19727	55.05517:58947 68.0451:59344 70.06618:9337 72.08164:1732271 73.08502:41868 76.03975:32852 84.04477:180227 87.0559:153573 96.04477:9666 102.05579:208881 103.05852:7710 113.07194:53972 115.05082:263876 118.08721:1379666 119.09042:51277 125.07133:10649 127.04987:44886 129.1022:658698 130.10553:27368 133.06145:142995 141.06526:146514 144.07626:25596 153.0657:34881 157.09706:92088 159.07639:75811 169.06013:991273 169.09766:20549 170.06525:54970 172.0726:102328 175.1073:1027823 176.11156:51158 180.07614:21453 181.06094:239295 182.06584:9907 186.12349:100461 187.07103:154773 187.10797:59604 190.08218:17530 198.08618:754921 199.08945:41688 200.06816:20044 210.08643:65038 214.12004:88243 226.08099:328827 227.08708:18080 232.12885:503972 233.13145:36954 237.09767:64264 238.08379:473079 239.08363:28813 242.10977:19372 243.14537:65613 244.09409:164333 245.09552:9603 255.10864:544376 256.11267:22529 265.09247:28722 271.14163:96344 273.1217:8715 280.12891:7445 283.10486:685615 284.10657:51345 289.1499:132822 299.13837:26356 301.11548:239561 302.11435:14907 319.12619:29785 336.16785:53199 354.17529:642476 355.17993:60172 372.18756:70530 382.16962:243544 383.17596:32983 400.18491:242443 401.18924:25821

					418.19632:278257 419.19614:35627
20-Hydroxyeicosatrieneoic acid	12.654	321.24246	[M-H]-	321.24353	59.01195:5130 69.90405:5733 72.04225:1484 78.95765:5751 96.84067:1684 96.95827:3190 116.92694:2880 165.12775:2950 167.10759:5473 181.12184:83344 182.12724:5862 182.69037:1428 191.10648:3335 197.11798:9397 209.09778:2508 209.11751:45727 210.11954:2340 242.11627:1715 259.24283:3027 277.25183:1528 303.2326:51490 304.2374:2043 321.24579:337700 322.24762:39163
N'-Formylkynurenine	6.38	237.08707	[M+H]+	237.08698	70.06564:4093 74.02427:45208 84.08144:4054 91.05513:11289 94.06554:349217 95.06941:14403 99.00845:68817 104.04964:21014 118.06541:88271 120.04508:86192 120.0812:31948 121.04806:3313 121.10239:3083 122.06106:10774 130.06474:6561 130.08585:3083 131.08638:84465 132.04504:17856 132.09:5643 136.07556:793136 137.07932:59352 146.05981:535595 147.06444:35661 148.03984:24515 150.05583:212745 151.05809:16268 156.04518:5449 158.06123:3821 163.08562:43452 164.06981:152554 165.07504:9880 174.05496:561687 175.05922:57012 191.08028:26702 192.0661:687344 193.07005:60789 195.1133:19379 202.05009:269646 203.05431:24843 209.09328:11035 220.05922:235669 221.0623:25522 222.14203:4924 237.0871:106984 237.12537:4544 237.16397:40232 237.22235:5215 238.09105:12657 238.14331:5696
1-O-Hexadecyl-sn-glycero-3-phosphocholine	12.75	482.36041	[M+H]+	482.3605	57.03444:7183 57.07079:51539 58.06584:30578 60.08159:189161 71.07371:20392 71.08622:42503 75.04475:54710 85.10188:31120 86.09711:478054 87.10065:7517 89.06057:38391 104.10735:4312998 105.10773:17376 105.1108:123167 124.99973:262910 133.08618:19371 181.02644:13413 184.0743:708617 185.0779:25979 283.17737:18836 464.35178:7296 482.35883:1232506 483.36472:184773
PE(16:0/18:2)	12.465	716.52155	[M+H]+	716.52246	53.03946:17961 55.05525:556596 57.03443:58657 57.07091:388792 59.05005:61120 60.0816:19715 62.06113:20596 65.03957:18701 67.05531:967536 68.05842:26982 69.07079:1580715 71.0501:85320 71.08631:245319 79.05476:276172 81.07038:1478305

					82.07362:48921 83.08647:1312002 85.06555:87962 85.10204:122762 86.09712:75342 89.0605:17593 93.07079:513320 95.08625:1573547 96.08943:69959 97.06533:89554 97.1016:1009777 98.10468:46544 99.08048:67556 101.09652:20093 107.08651:409072 109.10192:748970 110.10516:20339 111.08035:111785 111.11701:504290 113.09586:75550 121.10125:1175356 122.10454:61647 123.11679:373350 125.09655:60966 125.13239:64132 127.1124:18860 133.08618:22682 135.11725:1081207 136.12071:60558 137.13255:284456 139.11227:60179 149.13292:418129 151.14798:100642 153.12732:140864 155.01134:20689 163.14897:158525 165.16356:80392 167.144:61007 177.16518:82869 184.07448:959192 185.07565:23581 205.1971:18205 219.21043:213663 237.22244:490833 238.22348:45983 247.24226:131402 265.25201:508155 266.25476:28422 280.26266:118166 308.29388:471330 309.29706:25899 311.25632:207617 312.25876:26870 339.28802:187268 434.26672:18746 452.28339:20582 533.34851:16969 575.50574:10276330 576.50244:2698922 716.53192:27039
Bromacil	2.364	259.00711	[M-H]-	259.00876	59.90507:1436 64.32748:1242 66.55334:1262 69.81377:2684 85.60197:1354 87.00745:14077 97.52119:1218 102.94672:14298 111.00757:27946 125.00862:1743 126.98718:16129 127.98919:1756 139.70389:1421 146.93793:20958 154.98233:62348 155.98445:5518 168.99796:43006 169.99855:6108 186.55635:1305 190.92616:5722 191.01907:32097 213.00291:1925 220.84346:2454 221.84285:31352 222.84283:7016 223.83929:15478
Rhamnose	1.402	187.05809	[M+Na]+	187.05772	55.93536:3275 67.05473:3674 68.70036:7561 68.70414:12563 70.06621:11316 72.08166:3228 76.03984:13599 82.0659:3642 83.0857:3692 84.04493:60067 84.08147:5274 86.0608:3096 88.02213:23663 95.06065:6395 100.07665:12318 113.07196:5057 116.07126:2932 123.05579:12602 128.07053:22891 128.95076:4562 130.05072:3414 141.06519:19727 141.10193:16506 142.04941:6069 142.08635:18835 142.12329:3577 160.09735:4759 163.94037:3718 169.0477:3339 169.0623:10304

					170.0822:12380 187.05919:894184 187.10773:28362 187.12715:17627 188.06:42433 188.09314:19384 188.12895:11327
6-Chloropurine riboside	2.87	321.01498	[M-H]-	321.0163	55.36295:1228 56.33111:1132 69.39356:3163 71.14427:1172 87.92345:1584 96.83978:1493 102.9477:3081 103.91845:6900 146.93788:10362 146.95982:42310 159.91911:2740 174.95415:98527 264.89587:2901
1H-Indene-1,2(3H)-dione	3.809	147.04388	[2M+H]+	147.04405	55.67796:1526 57.03445:1362 69.03423:2064 69.97673:3554 71.04986:2041 72.93777:2315 84.04565:2250 84.08146:5444 85.02901:1838 90.94812:14197 91.05509:47231 92.05045:2213 92.05799:4876 97.00809:2240 101.05992:1769 103.0397:2185 103.05444:16957 108.95821:2489 111.06361:1519 113.96438:2281 118.94381:1815 119.04967:215871 119.08542:2897 120.04514:4193 120.05263:15059 120.08132:1954 123.96444:7132 130.02945:19661 136.95296:5895 139.01744:2413 147.04417:65284 147.09154:7801 148.03989:45329 148.07629:13694 148.09659:5091 148.11197:1697
Carnitine	1.315	162.1125	[M+H]+	162.11247	57.03444:37995 58.06667:143885 59.07417:29245 60.08162:4810143 61.08515:136772 69.18626:135500 71.04985:60791 84.08145:101031 85.02895:1166000 90.47287:29157 96.29794:26863 99.04497:58814 102.09199:1500460 103.03967:6228926 103.09527:40046 104.04268:193802 120.00397:36680 144.10255:61795 158.91847:27702 162.11223:27712884 163.03911:172226 163.11526:1541827
Methanone, phenyl(2,4,6-trihydroxyphenyl)-	13.551	229.05325	[M-H]-	229.05061	69.29661:9226 69.30044:9194 79.95588:589118 80.9635:50501 83.02362:11223 97.92986:5286 99.92403:5384 132.97975:18086 138.96301:59827 149.09656:1639856 150.09953:122571 154.95853:24558 160.97537:23249 166.9581:29752 181.89676:64591 182.95305:300408 183.95215:8729 184.87372:5654 185.94403:5847 202.88487:6409 210.94826:95936 228.96088:52414 229.05273:2931796 230.0555:248823
1,3,7-Trimethyl-9-	9.343	225.0612	[M-H]-	225.06293	69.47257:1671 69.47587:2464 78.16974:1207 80.96342:1758 82.70241:1318 89.02288:2407 96.83886:1457 106.75533:1261 114.06535:1289 114.27246:1132

Hydroxy-9H-Purine-2,6,8(1H,3H,7H)-Trione					122.89236:2026 123.90121:5008 125.89742:8834 127.89588:2140 136.90877:1313 139.89517:1255 140.1057:1453 141.91179:11880 144.90816:10202 158.08034:1841 179.05457:1710 180.96954:1305 198.0769:1902 207.10091:12234 224.80238:2806 225.09038:2387 225.11279:4684 225.79668:5880
Secobarbital	12.297	237.12418	[M-H]-	237.12442	65.0131:1936 66.0083:1559 67.63804:24700 76.97744:1466 96.07997:12650 96.84157:8638 106.03918:8362 110.05957:5587 116.92693:1548 120.0546:1808 130.03915:2278 157.06355:5930 166.12238:9143 167.08095:2463 170.05745:1673 172.0605:6611 183.06799:3176 194.06953:52651 195.07094:2869 196.0618:1938 210.07909:7559 211.06152:2104 226.05756:1874 237.12453:1050072 238.09024:2536 238.12866:103974
8-HETE	12.486	303.23135	[M+H-H2O]+	303.23099	53.00315:11463 55.05512:275317 56.05847:13172 57.07078:145879 59.05001:21909 67.05516:670739 68.05833:21326 69.07063:426153 71.04976:58250 71.08615:65403 73.06538:22837 77.0393:10152 79.05519:297537 80.05792:10850 81.0709:682173 82.07346:25318 83.04974:91178 83.08633:131836 85.029:23321 85.06535:98874 85.10194:61525 87.0451:28802 91.05502:831665 92.05791:47619 93.07057:448575 94.07409:23595 95.04906:14185 95.08604:528397 96.08939:26093 97.0651:46196 97.10147:42793 99.04511:23897 99.08129:13583 101.05989:112808 105.06998:1074898 106.07393:58151 107.08624:309856 108.08955:18794 109.06512:42144 109.10164:151469 111.0812:13112 113.06031:24289 115.07535:15632 117.07023:371393 118.0731:27492 119.08533:770279 120.08848:60251 121.06571:46423 121.10107:281237 122.10452:22768 123.08032:47148 123.11651:122869 127.07566:19786 128.06218:9302 129.07014:355340 130.07274:29079 131.08633:852320 132.08936:72131 133.0661:24384 133.10069:466218 134.10495:37729 135.08138:80501 135.117:128187 136.12054:13546 137.1324:114851 139.07474:19966 141.06996:197604 142.07326:15439 143.08575:191033 144.08922:14876 145.06526:17497 145.10176:607028

					146.10463:55086 147.07961:72481 147.11682:224192 148.12051:20919 149.09631:200638 149.13246:83144 150.0993:18704 151.14784:23372 153.09073:36844 155.08621:135288 156.08943:12101 157.10057:502870 158.10422:52030 159.08015:75054 159.1162:318061 160.11958:29676 161.09706:119174 161.13196:314726 162.1003:10851 162.13551:35363 163.11125:39463 163.14854:69859 165.09128:31504 167.10686:94249 169.10167:208729 170.10484:27747 171.11754:224879 172.11954:29695 173.09633:176677 173.1329:323810 174.09834:14596 174.13519:30783 175.112:59153 175.14891:358120 176.15103:36746 177.09077:42048 177.16435:29760 179.10603:79294 181.12096:30266 183.11641:485345 184.1196:55019 185.13292:70875 187.11244:269883 187.1487:175451 188.11369:31314 188.15312:14815 189.12866:17505 189.16341:213441 190.16623:23878 191.10759:170250 192.11142:19508 193.12337:61618 197.13243:206521 198.1368:26720 199.14813:47408 201.12704:545328 201.16476:291926 202.13205:73588 202.16769:37668 203.14458:29396 203.17973:137176 204.18309:16226 205.12186:187530 206.12553:15419 207.13692:33996 209.15302:21619 211.14716:74429 213.16374:47663 215.14301:202633 215.17767:75770 216.14626:27944 217.15932:33908 217.19557:29668 219.13928:332851 220.14319:44853 221.15312:63059 223.16965:24857 225.16479:41807 229.15898:44392 229.19484:17963 231.17551:17928 233.15512:297812 234.15764:43801 235.17018:12803 237.18385:29354 239.17802:44310 241.1942:207040 242.19919:33146 243.17438:22748 243.21034:164741 244.21466:21651 247.16827:89510 248.17355:16739 257.18866:104984 257.22778:63778 258.19308:15189 258.22852:11502 261.18533:36683 267.20898:300577 268.21436:56553 275.20123:28456 285.22195:531616 286.22495:98514 303.23288:4113140 304.23691:776660
Trimetozine	11.529	282.1311	[M+2H]2+	282.13358	55.05515:12877 57.07088:149870 58.07423:3606 60.08162:4411 65.87739:2490 67.05529:31190 69.07074:31396 71.02942:3660 71.08629:111513 72.08978:3802 81.07037:49182 83.08643:22488 85.10204:59050 86.09722:39361 86.10548:2920

					89.06057:210022 95.08628:76081 96.08946:3876 97.10149:16910 104.10745:59090 107.07062:2807 109.10183:42212 111.11691:9873 123.11665:20071 124.99977:3076 125.09659:3370 130.08588:5088 133.0862:80953 137.13251:4653 146.97318:5927 149.01884:6162 150.519:4167 151.09511:2686 156.51775:22070 165.0248:6355 165.52434:27887 166.54314:3059 176.53346:5959 177.03323:17710 177.11104:16295 178.91985:3273 181.02649:3107 184.07422:33921 190.87262:3224 193.53638:19726 196.93059:3192 202.54134:11298 203.42458:2568 205.04672:4741 208.88416:16861 211.20627:23378 213.88849:12395 214.93994:33187 218.86902:5714 222.94498:3007 224.877:3284 231.90112:51595 236.88004:32009 239.15036:2806 240.10078:26281 240.95639:11311 241.88527:26133 249.9109:5017 252.95494:43868 254.91582:12332 255.00848:3633 256.00388:257889 257.00516:20134 258.00473:4027 258.89926:3283 259.8941:45622 261.61942:25835 262.11731:9878 270.62323:20211 270.96429:16533 271.12509:3391 274.01471:30661 280.12903:4406 282.13031:22811 282.22174:9171 282.28018:17209 282.63138:5684 282.9108:20140 283.17291:17509 283.26331:10248
8-HEPE	12.272	301.21567	[M+H-H2O]+	301.215	55.05515:13836 57.07082:3022 67.05527:40515 69.07078:16997 71.03793:2854 73.02883:2975 79.0546:31306 81.07038:51090 83.04979:3677 83.08641:13549 85.06547:8331 89.06052:2645 91.05511:57185 93.07066:53677 94.07334:3719 95.08624:45581 97.0652:3857 97.10149:3152 101.05992:3237 105.07011:44228 107.08642:34764 109.10177:22544 113.06055:2826 117.07038:65152 118.07387:4063 119.08554:69084 121.10128:57580 122.10458:3362 123.08038:4960 123.11665:7997 129.07027:12051 131.08516:78270 132.09003:7509 133.10086:78928 134.10402:9140 135.07994:9656 135.11725:39302 141.07013:4359 143.08588:12649 145.10197:67408 147.08139:9446 147.11688:46932 149.09639:20253 149.13277:10779 153.09103:2784 155.08647:8859 157.10086:15171 159.11638:96728 160.12038:5353 161.09523:10644 161.1321:30381 163.11134:7955 167.10716:10262 169.1019:12662 171.11795:26349 173.09641:5356 173.1333:90998 174.13782:12362 175.11172:9976 175.1469:8829

					179.10611:13229 181.10042:11631 181.12354:4129 183.11702:17124 185.13254:18623 187.11256:12755 187.14926:26803 189.12648:4768 191.10793:7530 193.12332:12893 195.11636:10674 197.13255:12129 199.11351:9337 199.14789:23393 201.1272:12511 201.16493:18580 203.14143:3187 205.12192:4659 207.13998:10368 209.13347:11093 211.14716:2948 213.1279:15922 213.16348:4895 215.14305:4274 217.12294:4893 219.13643:14028 221.15311:13581 223.14792:15572 227.1456:4493 229.159:2676 231.13919:10677 235.17053:8303 237.1635:10336 239.17841:5204 241.15871:5295 241.19423:11900 245.15385:9933 255.17464:12548 259.16812:13945 265.19461:31429 266.19714:2925 273.18604:4324 283.20465:38605 284.21066:9534 301.21509:478724 302.21875:87247 302.26892:11512 302.3038:14194
Glycylleucine	6.426	189.12367	[M+H]+	189.12338	68.994:16736 72.34647:6029 84.08146:6668 86.09718:2660462 87.09526:7839 87.10061:91802 104.73582:5870 121.96648:6282 132.10164:2047192 133.10526:116049 136.95296:21026 143.11838:1193129 144.12103:60994 148.95329:6914 162.96918:7771 167.03137:6516 171.11133:19062 189.10181:10752 189.12453:113166 190.08722:12216 190.1071:29757
O-Desmethyltramadol	10.782	250.1776	[M+H]+	250.18018	57.07083:2911 67.05473:2857 67.81522:1651 69.07071:2089 71.04987:2326 74.87182:1700 81.0703:3275 95.08614:3451 96.84248:4971 98.98425:3173 99.23785:1531 109.10183:1803 111.04364:1639 133.06439:3127 133.10225:1612 135.08144:1590 140.21278:1782 183.44841:1546 191.10794:2789 193.08771:1893 204.92705:5730 222.93869:2348 227.94644:3709 233.11818:3825 233.18909:1907 250.1772:709702 251.12561:3451 251.18259:95694
12-HEPE	12.281	317.21112	[M-H]-	317.21222	57.03288:2678 59.01219:22738 69.03326:9776 69.64474:5009 73.91824:1226 96.07954:1413 106.04015:2529 107.0845:7818 119.08481:5299 119.95727:1433 121.10057:2554 131.6459:1451 133.10046:3107 135.11713:16418 137.09573:6262 147.1169:1712 149.13272:2084 151.11096:2350 161.09544:1906 163.11162:9140 164.11398:1326 177.09145:2526 179.10675:100837 180.11122:3352 187.46584:1414

					201.16319:9611 207.10187:19907 208.10922:13725 223.71925:1319 240.09924:2035 255.21288:39849 256.2132:2940 273.22:6123 299.20184:29848 300.20596:2454 317.21024:116984 318.21429:13278
Inosine	1.47	269.08704	[M-H]-	269.08807	59.01195:6125 69.68561:2408 71.01241:2051 89.02289:379007 90.02629:7973 96.95827:7318 103.91844:2569 122.89366:2795 125.8721:1822 133.01323:1963 150.90515:3238 154.89404:13670 156.91432:6413 157.00517:3205 157.01077:16108 168.88986:1641 178.91972:26193 179.92123:2579 181.05733:2018 200.92038:1706 201.0365:62049 202.04062:2082 207.03871:9931 225.04875:3188 231.96698:6691 268.83197:3169 268.95743:1856 269.07462:2308 269.21283:7199 269.83392:6215 269.95834:20422
Morpholine	12.333	88.07636	[M+H]+	88.07568	53.03949:20391 55.05519:219097 56.0502:56869 60.04542:48879 69.9612:23727 70.06623:320397 71.07373:39213 73.05312:12088 75.34533:8738 88.02228:9941 88.07635:2934647 88.11242:12619 89.07963:80935
[(2R,3S,4S,5R,6S)-3,4,5-Trihydroxy-6-[(2S,3R,4S,5S,6R)-3,4,5-trihydroxy-6-(hydroxymethyl)oxan-2-yl]oxyoxan-2-yl]methyl 8-methylnonanoa	6.286	519.24194	[M+H]+	519.24121	55.05513:5593 56.05048:37950 58.06623:17432 69.74356:14496 70.06618:5248 72.08163:359615 73.08507:5307 74.06063:359973 84.04484:74389 86.06076:7725 87.05581:44098 101.07146:87211 102.05572:109934 113.07191:17352 114.05554:6529 115.05083:150007 118.08721:368947 119.08168:18280 119.09031:13296 124.07574:6580 127.04996:79950 129.10228:290964 130.04926:7063 131.08195:27927 133.06142:17392 140.07114:253625 141.06482:52265 142.08627:7263 144.07791:12802 153.06583:5671 157.09695:51478 158.08185:44403 159.07623:77551 167.08252:13371 168.06598:181026 169.05966:68957 170.04477:34550 170.09264:14588 171.07732:7594 172.07268:235919 175.10735:850387 176.11264:38329 181.06065:30810 185.09239:153756 186.07544:22440 186.1236:15222 187.07082:58152 187.10764:12693 188.1044:7240 190.08218:20191 197.05638:6552 198.08878:90598 199.07082:22138 203.10294:934353 204.10619:34325 206.09209:5279 208.07043:13531 209.05598:13257 210.08896:5941 213.0871:53549 214.1199:24130 215.10417:35627 216.09789:17351 220.07155:5739

te					224.10173:7220 225.08815:6509 225.1362:14560 226.08122:67513 226.11655:5909 227.06761:12282 231.09912:682597 232.10196:34253 232.12878:110463 235.07129:5047 237.09763:7207 238.08107:43254 242.11372:66860 243.0983:39252 243.1456:161354 244.09305:31784 244.13037:11628 245.12465:5233 252.0983:13701 254.07631:13115 255.10875:85027 256.08936:5123 266.07816:25445 270.10757:118157 271.14175:289962 272.08868:15713 272.14404:18942 273.1217:19283 282.10922:14518 283.10446:68668 285.14444:8020 288.11774:263754 289.14999:701410 290.15494:53743 291.1091:16535 299.06479:7070 299.13367:32191 300.11725:8433 301.11572:26219 309.11655:8034 311.10019:7573 317.14575:18180 323.09909:18174 327.12964:63483 329.10837:6061 338.14648:16273 339.12769:7133 340.12494:7617 341.1087:5470 345.14124:217400 346.14087:16681 348.1283:7531 354.17615:42650 356.15683:26105 357.13968:5561 366.14001:59838 374.16327:15020 382.16962:16479 384.15442:175461 385.15591:17459 400.18536:30405 402.15912:110346 403.16278:6686 418.19653:48523 420.17194:26397 422.16611:6314 428.17758:7701 439.19189:6351 440.17996:16196 455.22598:8297 457.2037:6460 465.2077:30161 473.23572:46041 483.22421:63565 501.23218:140892 502.23352:21184 519.24274:599191 520.24353:89094
Dirithromycin	9.205	857.53979	[M+2H]2+	857.53455	60.04535:200601 69.07066:8288 70.02938:39454 70.06612:362036 71.06971:8244 72.08158:542740 73.08505:19766 74.06059:90393 83.06112:37908 84.04483:484929 84.08138:693659 85.04831:20964 85.0847:38772 86.09707:1111151 87.05588:102843 87.09522:6921 87.1003:50158 88.04024:19904 91.05515:44265 93.07063:7767 96.84242:9493 101.0714:351809 101.10782:23746 102.0557:144590 102.07532:15520 103.05394:28773 105.06692:174595 110.06132:6641 110.09637:6097 111.09242:13189 112.07616:5643 115.05089:5405 115.08717:14818 119.04963:18305 119.08146:28443 120.08118:1065540 121.08472:98207 126.05602:23528 127.04987:5480 127.08791:6290 127.12325:4849 128.10767:15288 129.06625:66024 129.10216:748090 130.05043:38887 130.06612:18134 130.10567:49239 131.11916:15187 136.07547:381728 137.07928:35283

					139.08734:18240 140.07088:26043 141.06688:20647 141.10188:54448 143.11827:5565 145.06044:4699 146.12843:7596 147.07623:17864 151.08621:6528 152.14339:24334 153.06581:17438 153.10173:15226 154.05118:6383 154.09837:37277 155.08093:65994 155.11761:44248 157.06142:8211 157.09689:5574 159.11429:19103 164.03366:23441 165.10303:71419 167.08249:21683 167.11731:18580 168.0659:4763 168.11343:7220 169.09746:80542 171.07736:29139 171.11337:18942 172.10742:23154 173.09244:24079 173.12859:224388 174.1312:22233 175.07208:5674 175.08746:12598 181.06079:85651 181.09802:12562 182.09331:57240 182.12874:15536 183.07639:51479 183.11368:39978 184.07199:7921 184.10753:20851 185.09242:25317 185.12816:11795 185.16521:30603 186.12305:57980 187.1076:18439 188.10191:12568 191.10281:18920 194.09154:17594 195.07712:42369 195.11356:35658 196.06058:7781 197.1272:13502 197.16396:4720 198.0885:52740 199.07089:52755 199.10808:13416 200.0735:6786 200.10303:23267 200.14064:47471 201.12431:161522 202.0822:30606 202.12086:6313 205.09674:20413 205.13295:12398 207.11415:59882 208.11026:7033 209.09322:19575 211.10922:7214 212.1046:58692 212.13989:6180 213.08665:38601 213.16042:7190 215.13995:15583 216.09773:205693 217.08035:15503 217.10332:23543 217.13472:64825 219.11479:4339 222.12312:21396 222.1608:14452 225.12334:6326 225.82977:4739 226.08426:5432 226.11974:5760 226.15497:84281 227.15846:5951 228.13591:29783 229.11906:43284 230.11215:63702 231.09575:5576 231.14906:4984 233.16541:43345 234.12369:49300 235.10822:25787 240.13489:65611 241.08347:54355 241.13728:5415 242.08473:13007 242.14868:35734 243.1313:4640 243.18166:6698 245.12105:24664 245.12833:64661 246.12497:17636 247.10829:28912 248.14044:5177 249.12204:6257 251.1028:19569 252.09825:18841 252.13622:17055 256.09271:26366 257.16153:20147 257.19556:24704 258.14615:15471 259.0928:25154 261.1619:15229 262.11719:19764 264.10864:4378 264.13211:26705 266.1517:21717 267.15109:6835 267.18423:4879 270.14496:40941 274.10034:6471 275.1059:4482 276.13452:60538 276.16934:24330 277.1163:8082

					282.10919:4996 283.14523:94336 284.12421:6953 284.15146:14808 285.15353:14177 286.14252:7423 287.1044:4698 288.15417:68193 292.12924:7894 294.14053:35694 295.10202:5528 297.15671:28049 300.19119:7645 301.14932:25779 304.13095:30211 306.1434:8311 309.19382:4772 310.10336:6220 311.1366:47947 311.17303:33933 312.11743:35838 313.11871:7379 315.16748:26369 316.12506:5761 322.14276:20638 323.1376:5125 323.17065:7882 327.12973:7289 329.14795:16546 329.18192:18487 330.18112:19201 331.12775:5489 332.12433:4506 334.14267:5777 337.19308:5030 339.1691:6439 341.1445:6047 349.19025:4727 351.16656:5084 354.25116:19404 355.25241:4715 358.17245:6771 359.16455:21743 369.17529:5226 375.20184:20843 377.181:6518 379.16196:33264 380.16513:5151 381.17947:4492 385.23511:43641 385.73718:7670 386.68018:21985 387.18549:15517 392.19763:19351 395.19083:4545 398.20322:22083 399.19229:5268 412.17865:4900 416.216:5877 417.2164:6060 426.20111:8063 428.25314:4534 440.17111:16085 441.16251:16269 442.15726:4457 444.21011:5558 455.21671:5556 458.18802:5950 464.21878:6859 464.29468:15569 465.29257:13293 476.2088:16921 476.71185:18673 477.20581:15451 482.31262:36537 485.21118:36628 485.72192:18033 494.2283:12927 496.77026:5895 498.23312:5968 509.20117:14801 520.29877:6039 522.24017:4481 527.21045:7206 528.19916:5049 541.76422:7261 544.30859:5633 555.25006:5315 577.29053:4513 579.24243:5560 586.32751:5624 597.33295:7227 616.34241:12261 616.83612:7351 633.28583:5902 641.24371:30811 642.2522:18547 650.4024:13361 659.26758:35433 659.84833:58047 660.26685:13611 660.3634:34452 660.86261:22156 716.38647:43971 716.89716:30486 717.38745:6718 754.32758:7239 755.33069:20423 765.41241:5466 769.46655:40567 770.45813:16667 772.3479:12976 773.34631:4802 789.9256:17801 790.43121:15477 798.11646:5478 798.43732:4384 830.41443:5398 846.95288:15872 847.44397:20541 847.95898:5207

2-Methyl-1,4-benzoquinone	3.803	123.04422	[M+H]+	123.04408	53.03947:8144 67.05524:17278 68.05042:30679 78.03465:8794 79.0545:27205 80.04993:407778 81.05368:6691 81.07029:18604 95.05011:247716 95.05984:28052 96.04488:245207 96.08141:16733 105.04555:22410 106.02961:37440 112.04034:67312 123.0506:125884 123.05577:2543788 123.09133:46945 124.03914:660725 124.05863:116585 124.07578:49325 124.08627:8482
Guaiacol sulfate	0.629	203.00381	[M-H]-	203.00192	54.31456:1320 70.0845:1355 70.99873:1488 76.98956:6930 80.98685:1455 87.00714:3029 89.02281:2473 114.98708:1864 123.87626:2627 127.2916:1403 128.967:1913 129.00316:154930 130.00354:17059 130.96364:47838 130.99933:2673 156.99812:558232 157.99918:63712 175.00894:6113 202.93956:4876 203.00539:1688 204.26213:1381
LPC(16:1)	12.233	552.33069	[M-H]-	552.33002	63.1542:1854 70.97834:1978 71.03111:2199 78.95718:94690 83.04871:2096 97.81229:1941 116.18218:2126 134.98448:1933 139.40521:1999 152.96725:2151 152.99434:61015 168.04178:58044 182.92003:1953 224.06886:324746 225.07109:10564 242.08018:81089 243.08382:2208 267.23358:3186147 268.23505:369345 327.23135:7405 403.22626:3887 421.24081:4414 424.66449:1964 429.92499:1929 492.31085:1668068 493.31754:274671 547.57001:2169 552.32825:9095
FT-sulfone; C6H9F3O4S	6.567	233.01149	[M-H]-	233.01009	79.95575:256128 80.96335:32264 96.95824:68473 99.9239:13643 100.92433:24565 101.92308:11192 103.91817:12067 107.04883:11533 115.91922:11980 116.91972:21133 117.9189:2981 117.9274:12200 120.01966:3498 123.04363:26042 132.86647:17463 134.86401:27015 135.04401:9007 136.0152:3474 136.93153:2696 138.03072:93468 139.03444:3455 144.94127:3489 144.95782:7655 145.04337:11887 145.92267:8371 151.03932:25425 153.05351:1709304 154.05687:127215 158.93202:3484 164.92589:11402 175.93938:2877 176.94162:24552 179.03407:8249 187.93324:13009 188.94307:22552 189.03368:47542 189.93915:3127 203.00026:143986 204.00499:3359 217.98601:3855 219.92497:2698 232.76311:7222 233.01259:1234695 233.1545:38902 234.01477:98482 234.15698:3430

Acarbose	10.645	644.24036	[M-H2O-H]-	644.24072	78.95766:19506 97.16686:1603 106.03918:2482 122.03374:1690 152.9942:27002 153.99704:1937 177.76401:1638 253.21706:44859 254.22186:38064 255.2321:1937 389.21143:9724 390.21109:10026 407.21838:1921 408.21783:2537 514.28137:10993 534.22052:68207 535.22705:12984 548.17828:1825 552.23096:188346 553.23016:32488 568.21204:2827 578.20795:2957 589.94861:1919 598.23157:204839 599.24506:36021 629.2807:1834 644.42859:11864
Prednisolone acetate	9.937	447.20477	[M-H]-	447.20239	59.94643:1507 69.59905:6241 74.02267:2868 93.20355:1389 96.95836:18227 127.52763:1340 128.59975:1410 153.13789:1462 174.95415:1696 207.43864:1830 233.71243:1699 240.64369:1405 279.23264:8916 294.30548:1693 419.19022:1694 447.20331:663906 448.20978:101000 448.30536:17707
3-Hydroxytetradecanedioic acid	9.421	273.17059	[M-H]-	273.1702	59.01195:1887 61.43646:1283 68.6144:10608 79.95585:5791 83.02364:1771 85.06445:1675 85.33894:1237 94.89731:1380 96.84248:2136 96.95835:18839 107.72964:1191 111.07959:2519 114.60221:1465 115.03864:2406 115.9486:1400 128.03319:2178 129.03659:1757 130.98218:20037 131.98187:1943 139.11226:1960 140.1655:1441 144.10913:2066 145.04893:5348 146.77574:1385 149.04651:2760 158.97748:36755 159.97858:1900 180.95338:1381 186.0788:1286 193.15994:21896 194.04401:2584 203.43968:1406 216.96707:1320 229.0027:14023 229.16125:4813 237.14844:40348 238.153:2481 255.15849:54132 256.16284:2900 273.12955:14775 273.17307:183625 273.62759:5894 274.00574:2216 274.13055:2144 274.17294:21519
Isonerylgeraniol-18-oic acid	12.485	285.22153	[M-2H2O+H]+	285.22101	55.05514:14154 57.07093:33997 67.05533:33700 69.07069:15861 71.04985:5697 71.08621:15643 73.06553:5473 75.02688:4010 79.05462:4215 81.0704:18637 83.04977:4529 83.0864:5179 85.06561:21860 85.10209:23364 89.06051:3813 91.05513:9389 93.03746:8846 93.07066:4778 95.08611:11239 97.06519:3786 105.07021:19086 107.08636:3856 109.1018:3779 117.07051:32072 119.0854:5368 121.10113:3910 129.07031:248028 130.07291:17094 131.08514:146567 132.08856:9145 141.07002:62166 142.07346:4526 143.08603:37089 145.1019:131250 146.10504:9285

					155.0849:56157 157.10085:139997 158.10458:14400 159.08026:60279 159.1162:20584 161.09715:4073 161.13205:4760 169.10187:73903 170.10529:5173 171.11781:95175 172.11984:10025 173.09656:52173 173.13297:50761 175.14906:10674 176.14278:29541 177.14677:5781 183.1167:177553 184.11986:20140 185.13289:44805 187.11273:39552 187.14896:48316 188.11421:4470 188.15092:5335 197.13269:95466 198.13647:13397 199.14828:34580 201.1273:138143 201.16519:101683 202.13231:16384 202.16728:13244 203.14413:3457 211.14716:56660 212.15169:4942 213.16351:29233 214.1676:4150 215.14333:45607 215.1787:36053 216.1823:3601 225.1646:33441 229.15889:16881 229.19514:5443 239.1433:13310 239.17838:8290 240.14958:3625 241.19423:114649 242.1996:22252 243.10263:10830 243.17455:3143 252.11732:8614 253.11887:110413 254.11096:196211 257.22812:11305 267.13931:36005 267.20929:281937 268.21451:56711 285.15222:129598 285.22211:1196161 285.2998:26088 286.15179:17157 286.22525:213019 286.27579:7415 286.30795:4883
Isoevernic acid	0.866	217.02899	[M-H]-	217.0273	69.27036:25648 78.95764:25959 79.95584:6060 96.84156:7812 96.92025:29311 96.95827:5872637 97.07881:5723 97.95745:33216 99.40125:5326 127.86822:43559 148.94817:19767 156.90312:7469 156.91431:33072 158.84656:5678 159.85963:21041 170.88248:94748 198.90706:10307 216.90936:254651 217.02768:9795
LPC(17:0)	12.872	510.3551	[M+H]+	510.35538	57.07082:93459 58.06373:19678 58.06667:68849 59.07417:18726 60.0816:483750 70.34458:30676 71.07372:94079 71.08621:90937 83.0864:20463 85.10193:32562 86.09711:1577065 87.10065:31453 95.08612:32056 98.98515:22755 104.10736:8200111 105.11088:166109 109.10181:26950 124.99975:470902 163.01653:28192 181.02414:18757 184.07433:10504974 185.07841:204897 240.1037:18767 255.30997:19283 258.11053:181470 327.2876:245063 433.27289:66813 492.34729:781906 493.34393:82914 510.35828:3195110 511.35461:384036
3-Nitrotyrosine	6.217	225.05081	[M-H]-	225.05173	57.70257:1257 71.00501:1430 80.96409:1450 87.92342:1380 89.02286:1418 90.66153:1219 99.00763:2251 99.92401:1279 106.71451:1249 109.59561:1373

					111.01865:152231 112.02154:5234 122.89233:5085 123.89987:5188 136.90874:2970 141.91161:15709 144.90817:7654 152.07048:2021 156.06578:1844 164.03433:1667 219.18753:1441 225.09033:2598
Galangin	6.218	271.05679	[M-H2O-H]-	271.06009	52.49776:1145 54.91133:1186 68.34844:6055 68.35273:6153 88.98623:1954 91.22498:1462 96.83884:2508 99.92401:2282 113.56553:1306 116.03269:2593 123.19006:1408 129.00319:1321 142.0493:7761 151.02475:2977 156.99817:9390 158.05957:1595 171.70355:1322 198.00813:1447 200.6424:1638 213.52574:1417 226.00258:30521 271.19263:1791 271.22653:7876
4-((9S)-3,5,14-trihydroxy-10-((E)-((1-hydroxybutan-2-yl)imino)methyl)-13-methylhexadecahydro-1H-cyclopenta[a]phenanthren-17-yl)furan-2(5H)-one	11.639	498.28244	[M+Na]+	498.28262	55.05515:17160 57.03445:22920 57.07087:166240 60.04544:99701 67.05514:31131 69.07079:42811 69.68396:12273 70.0295:17853 71.08624:157988 81.07103:76943 83.08646:76262 84.08144:96446 85.10191:112214 88.04028:788127 89.04299:8717 89.06052:9216 95.08609:105312 97.10142:36893 98.98508:131611 99.11793:5520 106.05032:409497 107.05379:6274 109.10178:68616 122.09689:7569 123.11673:38830 126.02262:7837 137.13249:14146 155.01134:399436 168.00607:5624 173.02089:110791 186.01532:17829 196.03723:6655 239.2383:88346 242.04198:17181 257.24771:8470 260.0528:19985 270.24234:7122 280.26236:29929 313.27136:1629822 314.27817:188466 326.26715:152451 327.27585:15536 337.25198:7696 393.23874:241834 394.24646:23645 462.27087:8660 480.27579:21116
methyl 3-oxo-2-[(3,4,5-	13.462	208.03955	[M+2Na]+	208.04182	56.96548:22755 69.66357:81728 84.96049:3368828 94.28312:18480 102.97118:409658 115.96453:227059 116.96111:25824 117.95935:963572 118.9672:21139 120.98118:72097

trimethoxyphenyl)methylene]benzo[b]furan-5-carboxylate					125.98621:938264 133.97452:41442 135.97006:336891 143.99739:1042098 144.97108:18747 158.98598:149777 162.00778:162702 167.01283:277022 167.9875:65070 168.28111:19332 174.98434:23328 184.9707:32665 185.02319:24145 185.99529:166666 209.01308:44464
LPC(18:2)	12.115	520.34003	[M+H]+	520.33978	55.05514:777783 58.06666:3856008 59.07416:1164582 60.08162:22456202 67.05523:2210103 69.07068:1029814 71.07362:4740214 81.0703:4120631 83.0864:840650 86.0971:79582224 87.09987:718238 93.07066:830586 95.08624:3648579 96.84425:2800158 96.85058:1057208 98.98515:807916 104.10735:382556096 105.11067:8579391 109.10179:727208 121.10112:883696 124.99966:29520478 163.01651:1993990 181.02644:727587 184.07434:481107552 185.07603:11593689 199.03621:729843 258.11078:7606820 337.27515:7965690 338.27631:1038063 443.25281:2505908 502.32953:33584552 503.3349:2527775 520.34381:143093664 521.34711:8711391
1-(Aminocarbonyl)-L-proline	1.15	181.0585	[M+Na]+	181.05832	69.13772:20500 136.07559:3621 181.05885:988732 181.13281:7794 181.95172:4735 182.06128:57145 182.07991:27094 182.1172:6820
Threonic acid	0.904	135.02826	[M-H]-	135.02992	59.01238:102869 69.55996:6770 69.56326:11480 71.01257:43643 72.99158:144780 75.00726:1765778 76.01042:22506 87.00718:7025 89.02282:51981 96.9673:6324 117.01807:149702 134.86406:317386 134.89365:159155 135.0291:946281 135.8934:6519 136.03174:33468
Tetrahydro-2,5-furan-diacetic acid	6.923	187.05984	[M-H]-	187.06065	56.20221:1584 59.01237:400834 60.01561:7726 69.09844:8938 74.02328:1933 83.04881:14044 84.93734:1976 87.92422:1880 97.06407:11932 99.92411:27266 99.9477:18590 100.93201:2270 115.91885:14353 116.04932:2678 116.92693:7359 123.07993:1837 125.0591:12473 125.0961:109280 125.87202:9688 126.09876:6185

					127.03871:8129 129.93515:1803 133.03796:1709 141.86679:13787 142.94147:3176 142.96254:7483 143.06964:269081 143.89769:2454 143.93709:24433 143.98615:1679 144.07333:14537 157.86191:5328 158.89215:5426 159.87691:10130 160.04961:6351 169.08571:2818 175.93942:2122 186.93153:2074 187.06088:50884 187.0965:117027 187.1329:5752 188.03447:5970 188.10052:7067
12 Hydroxy arachidonic acid	12.491	319.22742	[M-H]-	319.22787	57.0329:249388 59.01208:1976653 67.05358:94339 69.03333:493701 69.10659:206122 71.01239:221542 83.04872:107934 107.08466:861758 109.06434:82241 117.06974:91199 119.08486:88261 127.11069:76434 133.10052:110645 135.11707:1743513 139.11073:1070174 145.10027:75502 153.09116:111994 153.12733:598879 161.09552:247306 163.11168:1755177 163.14923:258452 164.11604:72966 177.09152:128466 177.16307:72400 179.10684:11430944 180.11133:617423 181.12198:230993 189.16443:96100 203.17859:1133097 207.10132:1192866 208.10889:1624004 229.1942:431267 240.09937:355620 257.22757:4702039 258.22849:397570 271.20956:83257 275.23611:519086 283.20435:67464 301.21521:5672926 302.21808:483854 319.22928:17158614 320.23218:1654684
Methyl 5,6-diacetyloxy-10-hydroxy-2,4b,7,7,10a,12a-hexamethyl-12-methylidene-1,4,8-trioxo-4a,5,6,6a,9,10,	1.695	280.99542	[M+H]+	280.99219	69.03423:9129 72.93776:7743 73.02942:10611 74.95359:2759 80.9486:13390 82.9458:7533 85.02915:18942 87.045:11970 90.94801:15595 96.92205:3027 97.02892:3155 108.95821:3320 109.02924:13334 113.96437:6675 114.94718:17117 116.96338:22885 118.94257:9940 119.95163:2493 127.03906:10961 128.96324:2788 132.95822:105015 134.97427:10823 136.95291:21192 140.9399:3421 140.96371:3202 141.9595:6677 143.92717:2695 144.95813:23473 146.97316:9622 149.94095:3731 150.96857:41573 151.93706:2932 155.9747:22948 156.95886:2243 158.97369:50059 159.96867:4037 160.95357:50185 162.96935:15948 164.91896:52493 168.96016:3769 170.97295:19276 172.95393:9820 174.96893:900342 175.97206:45185 176.98566:16560 178.96283:17458 181.98894:2577 185.95999:3458 186.96931:120219 187.9724:9054 188.98544:16395 190.96426:19978 192.98036:37864 196.95415:3030 198.96977:78491

10b,11-octahydronaphtho[1,2-h]isochromene-2-carboxylate					199.97163:3982 201.97932:9762 202.96297:49578 204.97972:18474 206.99585:2345 208.88414:3610 215.99544:56951 216.98044:43068 220.97528:139236 221.97572:8069 222.87784:12581 226.89255:3149 227.99538:11348 234.98926:120592 235.99356:10078 239.99438:10513 243.98944:15531 244.97556:2644 247.81834:2433 248.87993:3759 253.00032:80984 253.88062:8094 254.00342:8522 258.00482:22006 262.00061:9218 262.98447:34660 266.82346:16278 276.01718:12349 276.84976:2865 280.99341:81997 281.10089:3468 281.99701:9640
3-Hydroxy-2-methylpyridine-4,5-dicarboxylate	1.963	196.02188	[M-H]-	196.0246	71.01808:1653 72.2346:1436 78.95765:2838 88.04869:1404 96.95827:2214 103.91842:22890 105.86542:1317 112.03956:1414 116.92694:1305 121.66508:1434 128.03323:425674 129.03665:15732 143.0826:1351 150.9632:1557 151.89636:9343 152.89363:179336 152.95296:1797 158.84467:2228 160.83986:191809 161.84088:2239 168.01523:13740 168.88777:5225 168.96065:2764 183.87646:2845 195.80957:173113 196.00714:3349 196.88251:11083 196.96059:1366
tert-butyl 4-methyl-2-[3-phenyl-2-({1-[benzyloxycarbonyl]pyrrolidin-2-yl}carbonylamino)propanoylamino]pentanoate	12.078	566.32233	[M+Na]+	566.32251	56.05059:3862 58.06625:4338 60.08158:29399 67.05515:26279 69.07069:13050 71.07386:22275 79.05529:14033 81.07018:24787 83.0864:5691 86.09712:309365 87.09988:4639 91.05514:14608 93.07067:17977 95.08612:18780 96.84624:33541 104.10737:1872778 105.07005:12143 105.11081:54056 107.08636:5551 109.10181:5962 117.07033:4304 119.08541:16715 121.10114:6526 129.05495:6401 131.08647:6025 133.10222:5047 135.11716:4540 145.10193:3741 145.14836:3079 146.98157:351497 147.11691:3312 147.98553:4225 157.10255:3971 159.11816:2955 164.99261:4389 171.11769:3371 176.9921:21662 184.07445:9640 188.00906:4179 203.17986:4249 226.25533:3752 227.22676:2989 312.26407:6318 361.27228:25316 383.25528:35969 458.73752:2964 463.22125:30191 466.03613:3153 507.24551:1238759 508.25412:192779 566.3205:326819 567.33154:53002
7b,9-Dihydroxy-3-	11.674	432.23895	[M+ACN+H]+	432.23801	55.05477:6508 57.03405:13484 61.02905:6875 69.03426:102193 69.88301:5631 69.88633:8329 71.04987:6281 81.03432:16262 83.04988:33204 85.02902:24609

(hydroxymethyl)-1,1,6,8-tetramethyl-5-oxo-1,1a,1b,4,4a,5,7a,7b,8,9-decahydro-9ah-cyclopropa[3,4]benzo[1,2-e]azulen-9a-yl acetate					87.04442:16127 89.06053:15458 91.0395:17142 91.05515:17207 99.04498:7814 103.03971:5887 107.08641:103834 108.08866:4357 111.04465:37935 116.24461:3671 119.08545:1347726 120.08879:90782 129.05496:67776 133.06435:33786 133.08623:3680 135.07991:499136 136.08458:26057 147.06613:43613 177.09093:3738 281.13593:50508 282.14081:7365 296.12894:3856 397.30814:4910 415.20816:37040 416.21625:5580 432.18164:25667 433.33289:4156
PC(16:1/16:1)	13.09	752.5177	[M+Na]+	752.52002	55.05513:438319 56.05015:151181 57.07082:259668 67.05518:734150 69.07066:1068480 70.07674:35608 71.07372:534296 71.0863:125785 79.05529:223882 81.07027:1063900 83.08636:832337 85.06544:40542 86.09709:6072174 87.10046:178343 93.07071:370030 95.08607:1040699 97.10143:694279 99.08141:38601 107.08621:320709 109.1018:416459 111.08133:115355 111.11685:268378 121.10105:786888 123.11774:329087 124.99973:38138 125.09654:42182 135.11693:739656 136.1207:37491 137.1324:248694 139.11226:33533 146.98152:14863715 147.98523:166499 149.13243:265587 151.1479:30621 153.12691:44199 163.01649:77514 163.14883:108153 164.99245:238418 177.16463:30360 188.00891:340940 219.20975:209016 237.22185:504234 238.22346:30107 417.24072:72594 439.22668:565601 440.22375:72097 476.31494:148390 498.29648:116468 547.47693:2181526 548.47467:642544 569.45148:5800578 570.45825:1552090 693.43903:4710056 694.4444:1429905 752.52283:5493490 753.52283:2201788

Taurohyocholate	10.65	514.28387	[M-H]-	514.28387	51.00817:100702 54.29372:103960 69.60181:265912 85.53591:107924 124.00591:112493 175.83321:99259 214.57127:129645 283.18179:113801 298.05695:118734 395.46112:145606 403.14191:117626 514.15741:774224 514.27924:124270832 515.28699:18881852
Uralenneoside	6.265	285.05862	[M-H]-	285.06161	69.78226:5757 72.99163:2154 87.04364:35560 99.92404:4955 103.91844:1639 109.03845:6400 110.04207:2887 113.02229:8138 115.91901:1683 128.03456:1835 131.03377:237835 132.03654:7637 153.01588:74083 154.01883:2958 166.90904:1769 171.0143:15800 181.89668:7525 183.91209:25419 186.99217:5125 187.04553:1888 195.91089:1608 201.92082:1953 223.8782:1524 239.90196:2435 242.08412:3007 257.91385:6643 267.03506:6466 268.03528:1946 285.04523:21274 285.09424:7355 285.20844:3291 286.04626:2431
Creatine	1.353	114.06667	[M+H-H2O]+	114.066	59.05007:33657 68.05045:112562 69.03424:48836 69.0707:116624 70.06557:439819 72.04515:370704 72.08165:46777 79.05531:50450 86.06079:105118 86.07215:2139223 87.07523:50733 96.08142:36948 97.0652:32256 104.08202:225529 114.06712:15478381 114.09139:1248129 114.1284:42217 115.06976:476210 115.08723:55671
LPC(18:3)	11.599	518.32544	[2M+H]+	518.32465	55.05514:13109 58.06666:53226 59.07459:15359 60.0816:366012 67.05524:47798 69.07069:11427 70.65898:17928 71.07371:62164 81.07097:46054 83.0864:11551 86.09712:1175404 87.10065:20339 93.07066:46029 95.08611:21515 98.98515:10178 104.10736:5289066 105.07005:14899 105.11095:152336 107.08636:11452 121.10112:22957 123.11792:10818 124.99966:422806 135.11714:14625 163.01454:37533 181.02644:10630 184.07433:6953051 185.07602:194520 258.11093:96882 335.25867:72662 396.55801:10756 441.24176:19953 500.31729:434394 501.31683:60691 518.32349:1673507 519.33191:212181 519.44458:12959
Palmitoylcarnitine	11.172	400.34201	[M+H]+	400.34207	55.05514:8361 57.03449:47147 57.0709:77508 58.06582:8579 60.08163:727058 67.05523:24471 69.07068:30970 69.74689:16882 71.08624:87955 81.07025:51455 83.08647:55117 85.02903:6222634 85.1019:67046 86.03271:173547 88.07632:20434

					95.08622:63487 97.10147:28552 109.10201:37106 123.11662:20765 137.13248:8562 144.10262:162294 145.10522:7340 239.2382:118793 240.24136:10019 257.24768:25553 338.34152:57449 340.22614:7696 341.2706:375594 342.27115:65577 400.34491:3293781 401.34222:585935
Sebacic acid	9.472	201.11205	[M-H]-	201.11322	52.76717:1498 53.58136:1265 57.03288:6474 59.01233:7265 62.31999:1363 65.99706:2039 77.82538:1577 87.00715:1289 89.02281:3468 99.92396:2043 111.0795:7434 129.10185:1582 130.98227:1545 132.05515:1780 133.03932:1397 135.29285:1415 137.09573:1701 139.11061:270741 140.11488:21196 148.04874:1395 157.08586:1837 157.12134:8124 172.88533:3054 174.06636:2544 175.06166:2735 183.10081:138290 184.10425:9826 194.7482:1728 201.11218:472549 202.11459:37766 203.11517:1348
Pyrroline hydroxycarboxylate	1.956	128.03375	[M-H]-	128.03477	55.94059:7580 67.28857:6375 70.36363:10657 82.0282:35461 84.04315:6855 85.028:14367 96.83978:8515 99.23698:6802 99.8625:6907 106.13558:5921 109.245:6564 123.75093:6559 128.0332:1634836 128.87686:12141 129.03658:72574
3-Aminobenzene-1,2-diol	9.395	126.05529	[M+H]+	126.05495	53.03948:10777 55.01875:10620 55.05516:4098 56.0502:9159 56.96589:7705 57.03445:3824 68.05045:3813 70.06611:13376 71.04987:11650 71.06123:2306 80.05054:27708 81.03431:11805 81.04539:16653 81.0703:3747 82.0659:11700 84.04485:14477 84.08147:28955 84.96062:152910 85.07664:10283 86.09715:3930 98.02458:3558 98.0604:23034 98.09734:3390 99.04498:3529 99.0562:9021 99.09174:4146 102.94851:8480 102.97126:27505 108.04492:123012 109.02847:21020 109.04025:7900 110.02415:8491 110.06029:3980 120.98083:13729 125.98641:9820 126.05483:184594 126.06558:24575 126.09229:28613 127.03909:395085 127.04994:50243 127.08662:16023 127.12323:14960

Pyrenol	1.223	219.08298	[M+Na]+	219.08047	62.98244:1962158 69.56274:77518 69.56605:62962 80.94858:228199 82.9458:7703732 86.99362:1258915 90.97692:2104308 96.84517:46263 159.05156:10687267 160.03584:66125 160.0533:497766 177.06181:6538142 178.06491:185808 201.07307:214621 202.05585:636013 219.08414:23034472 220.08754:1294093
2-(3,4-dihydroxyoxolan-2-yl)-2-hydroxyethyl (9E)-octadec-9-enoate	13.231	429.31921	[M+H]+	429.32108	57.07082:8896 65.74067:1426 69.07069:1622 71.03111:1508 71.08622:6896 81.07029:1644 83.0864:2666 85.10193:2389 86.09714:2819 87.0444:3257 114.06135:1550 128.38458:1584 149.0965:6382 163.11195:12359 164.08383:2075 165.09129:57149 166.09557:2124 177.0909:1788 184.07404:14802 191.10789:2025 205.12189:6453 257.07953:1587 395.25806:1629 429.32269:2192666 430.32028:346178
2-Chloroacetamide	2.317	94.00612	[M+H]+	94.00542	55.05515:15626 56.96547:30033 58.0298:1098472 59.03759:167260 67.05528:77913 69.3054:38767 71.06137:79745 75.99583:44662 76.9798:569253 94.00587:4491088 94.06559:80338 95.00912:57855 95.01678:74480 95.05003:82601 95.06059:91473 95.08629:70233
cis-6-Nitro-p-mentha-1(7),2-diene	10.986	180.1019	[M-H]-	180.10245	51.48626:1331 59.01207:11089 66.03323:2053 69.88133:2630 71.01202:14829 73.02773:5747 83.02377:37467 86.98489:1463 89.02298:12387 92.9184:15757 94.02829:4929 98.02278:1791 101.02258:16308 101.91824:1383 108.04317:6616 109.03953:1609 112.92094:6847 112.98356:7547 113.58508:1341 135.07977:2952 135.91148:1819 136.07393:4462 136.08296:11232 136.90929:15090 136.98174:1527 137.03345:1613 138.01852:1539 138.0293:1660 147.61073:1378 156.492:1412 163.06038:2101 166.02699:5824 180.02887:2204 180.07469:9198 180.10219:273554 180.81264:1833 180.92346:18489 181.04808:4276 181.07169:38079 181.1057:23771
Glutamic acid	1.295	148.06044	[M+H]+	148.06049	56.05021:32728 59.18458:13024 68.48098:121677 69.03806:13477 72.91533:17243 84.04501:2477061 85.02903:29462 85.04854:127624 97.18232:14900 102.05594:743425

					103.05857:23730 116.97551:12934 130.04944:505328 131.05377:56830 137.53549:13466 148.03851:21396 148.0612:120989 150.92969:15676
2-Propylglutaric acid	8.848	173.08044	[M-H]-	173.08194	57.03291:1608 59.01208:9421 65.0126:6336 65.9966:1961 68.50253:4245 68.50629:6992 81.04431:1537 85.02798:1545 89.02288:2940 93.03313:5584 96.84157:2601 99.92422:12439 105.01823:1484 106.03919:1656 111.07971:69230 112.08343:2912 115.00236:1508 115.91899:9072 116.92693:7026 119.02328:8109 127.11072:2845 128.87689:69249 129.09091:180740 129.87616:1795 130.06462:59790 130.09402:7117 131.03497:3079 131.08044:9560 132.05524:6706 144.0435:2995 146.035:16287 146.05989:1401 154.94574:6383 155.0705:18376 155.94568:1470 172.05194:1787 172.82941:6760 173.02136:1873 173.0471:14800 173.07967:206017 173.11443:10975 173.95546:2257 174.01857:2317 174.05519:42999 174.08591:10785
Prostaglandin E2	10.252	351.21698	[M-H]-	351.21771	57.03291:2131 59.01194:5428 67.05359:1872 69.0331:1654 71.0493:1624 85.02798:1599 86.44266:1492 95.04827:2605 109.06435:9339 113.09515:13759 121.064:2790 135.07974:2424 137.09583:1627 159.08035:1839 161.09554:2154 163.11171:5392 171.1161:1803 173.09474:3141 174.1035:2017 175.1127:17464 177.09155:2595 187.11102:2088 189.1274:88420 190.13048:6375 191.14404:2607 195.10199:17791 203.14275:1663 206.13008:1714 207.10187:6099 217.15811:7228 233.11707:13561 235.1317:13920 253.19467:1543 269.1918:1480 271.20532:174558 272.20752:16077 315.19461:87089 316.20047:10677 333.20706:67640 334.20728:6711 351.1427:1529 351.21759:9374
2-(3-methyl-2,6-dioxo-1,3,7-trihydropurin-	0.871	223.04553	[M-H]-	223.04732	59.01214:137198 69.94564:3932 71.01205:143016 72.99124:65150 73.02789:37137 75.0073:15648 78.95765:5362 81.03255:4408 83.01215:6237 85.02813:39842 87.00739:20338 89.02298:105665 90.02629:3444 94.92381:58209 95.01226:5070 96.84157:4607 96.92056:34320 96.96838:20691 97.02837:30420 99.00672:17074 99.04317:2855 101.02265:112232 102.95561:4911 103.00235:408309 104.00591:10362 105.01721:5480 113.02248:45696 115.00237:11306 115.03746:3015 119.03314:9805

7-yl)acetic acid					125.0234:23887 129.01727:22847 131.03355:17181 133.01335:152386 136.91637:3469 143.03386:42736 145.0125:10674 147.02904:3700 154.89819:8287 154.92757:38500 161.04515:5665 163.0229:81305 164.83444:20761 165.03917:47328 176.83481:2871 177.04018:3637 180.85414:9889 186.8661:8927 198.84856:2938 205.03419:4318 223.04492:194232 223.83054:14580 224.04985:13355
O-(17-Carboxyheptadecanoyl)carnitine	10.218	458.34787	[M+H]+	458.34818	55.05514:6383 57.03444:10046 57.07077:15208 58.06581:17481 60.0816:177641 67.05511:19570 69.07064:19298 69.62772:6997 71.08624:21637 81.07095:36952 83.08633:23137 85.02898:819081 85.10192:10661 86.03265:20260 89.06043:20476 93.07066:4286 95.08614:52573 97.06519:8914 97.10133:20337 99.08143:2760 104.10735:3680 107.08636:9500 109.1016:25533 111.08135:10528 111.118:8614 115.07551:7494 121.10089:23574 123.11657:20346 125.09636:15407 129.09109:6963 133.08586:12595 135.11699:21330 137.13248:6708 139.11229:11623 140.14348:3497 143.10695:8088 144.10234:51500 147.1169:3172 149.13242:21051 151.14793:3581 153.12694:11473 157.05026:4184 157.12309:7004 161.13206:4007 163.14856:14576 165.12735:2811 167.14401:9747 171.1389:3052 177.16466:8720 179.14246:3370 181.15819:7551 184.07443:2948 185.1545:2992 195.1732:5741 233.22559:26472 241.14447:2845 243.21051:2588 251.23831:42332 255.15913:3158 261.22073:14458 269.17526:3508 279.23245:29913 283.19092:6441 297.20959:2626 297.24091:15846 299.13843:3505 315.25238:190583 316.25922:20578 353.26636:26876 381.26352:31049 382.26889:3559 399.27567:8593 458.34598:1092212 459.35269:189784
4-Ethylphenylsulfate	10.261	201.02202	[M-H]-	201.02216	59.01235:8269 69.87022:5867 79.95578:255855 80.96336:47227 89.02284:7568 99.924:7975 106.04019:7196 121.06393:793469 122.06812:47162 127.11203:2216 133.04955:2929 139.1122:10074 157.1214:3033 159.067:2289 172.88539:8471 174.06642:2348 175.06172:2233 183.10083:3582 201.02281:1283211 201.11026:19815 201.15009:5556 202.02438:82335 202.07323:1869

Theobromine	1.284	219.02681	[M+CH3OH+H]+	219.02786	52.04974:11490 62.98244:254937 68.13892:11290 69.3147:41903 69.31853:18194 78.94209:10652 80.94844:72712 82.94581:2435837 86.99361:141507 90.97691:735494 115.87935:10306 159.05151:1273599 160.05315:51156 177.06183:795402 178.06494:20348 197.95676:10591 201.07309:14145 202.05542:80223 219.08415:2739812 219.17336:20058 220.08786:150993
LPC(18:0)	13.451	524.37073	[M+H]+	524.37109	55.05513:6536 57.07082:52438 58.06623:12724 60.0815:45918 67.05522:6840 69.07068:10733 69.093:10012 69.09682:5937 71.07372:33737 71.08627:50827 81.07086:27018 83.08637:20256 85.10201:39251 86.0971:506584 87.10063:12572 89.06049:4905 95.08617:35197 97.10236:16669 104.10735:2641494 105.11066:69322 109.10203:22896 123.1166:13163 137.13246:5300 146.98152:612371 164.99257:14175 176.99254:24904 184.0744:6836 188.00902:17837 341.30563:316003 342.30692:40180 443.25403:43343 487.27884:1830221 488.28036:258392 546.34979:737632 547.35547:134561 547.47797:13728
2-Methyl-4'-(methylthio)-2-morpholinopropiophenone	2.383	280.13852	[M+H]+	280.13657	55.05515:3944 69.03423:20683 70.55995:5880 72.08173:157813 73.02882:12615 84.08145:54910 85.02898:31009 87.0444:6541 97.02923:41552 99.04498:6745 100.07569:4820 101.06004:24201 102.09206:28017 112.04021:5075 112.07622:6342 114.09145:23240 118.08612:224852 119.09035:6746 127.03913:55262 130.086:128411 142.08632:5958 143.05827:28496 145.0489:13689 150.09081:6231 152.10773:4625 154.08571:12117 156.10262:4305 160.09709:27150 161.06815:26443 166.08746:3766 174.96919:54859 180.10141:7196 186.96941:5494 196.09679:44190 198.11292:93149 199.11617:5346 208.88414:7786 216.12219:422307 217.12617:36435 220.97542:4447 226.89255:20340 234.13394:104676 235.13632:6064 244.11957:390258 245.12213:46099 262.12933:970179 263.13043:90809 280.13831:56944 280.99365:4012 281.1456:6140
Mmv676476	11.96	379.15793	[M-H]-	379.15512	67.53424:21565 78.95699:1949 84.93735:6778 96.84248:8965 99.92425:219896 100.92432:9656 100.93201:18863 113.59426:1799 115.91917:90690 116.92707:239264 117.92767:10519 172.7045:1566 184.01845:1654 229.01656:1807 274.21234:1483

					293.21225:1774 311.16837:8847 311.22043:1834 317.15683:5132 322.0885:1607 335.16556:7953 335.25858:25103 349.14801:1756 361.11063:2152 361.14969:4753 367.15332:5286 375.12769:9170 379.15662:293585 379.25467:7915 380.16095:43862
TG(18:1/18:1/22:4)	9.154	952.82434	[M+Na]+	952.8327	69.07066:4602 70.0295:22702 70.06613:274001 71.06915:13647 72.0816:329250 73.08506:13095 74.06113:55474 82.06586:5242 83.06118:25628 84.04485:397331 84.08141:585728 85.04832:11989 85.08454:31763 86.09709:659514 87.05588:58770 87.10046:39692 88.04025:14029 91.05512:32769 93.07063:4716 98.06044:4811 99.09262:5790 101.07147:239010 101.10807:14267 102.05578:140550 102.07533:15719 103.05453:19580 105.06692:101352 110.06023:5861 110.09747:15505 111.09354:8207 112.07616:5517 113.10725:3613 115.05089:11994 115.08718:6642 119.04963:16253 119.08157:25242 120.08123:654118 121.08458:53336 126.05611:14875 127.05124:5494 127.08656:6442 128.10768:13700 129.06596:40525 129.10219:681809 130.05061:36342 130.1057:41304 131.11774:14256 136.07549:275653 137.0791:24237 137.10808:4873 139.08736:13112 140.07089:23628 141.06529:14599 141.10176:49805 143.11827:4511 146.12845:11191 147.07625:7105 151.08623:3664 152.10765:3691 152.14355:22210 153.06581:17229 153.10175:12161 154.09842:28082 155.08084:62071 155.11725:36625 157.06142:12268 157.09689:3857 159.1124:5596 164.03387:6652 165.06694:4305 165.10298:54443 167.08044:17273 167.11732:12763 168.0659:5069 168.11345:5558 169.09755:55730 170.09262:4378 171.07724:24669 171.11337:16805 172.10909:16655 173.09212:21212 173.12877:170289 174.13121:12966 175.08748:4851 180.07613:4831 181.06097:51156 181.09572:12109 182.09354:34199 182.12874:15734 183.07671:50916 183.11424:35537 184.07437:14717 184.10754:11684 185.09286:31703 185.12816:12519 185.16635:21875 186.08986:6418 186.12347:48572 187.10736:22771 188.10437:15563 189.08693:5982 191.10281:15056 194.08897:8186 194.13002:5222 195.07767:35688 195.11345:26176 196.07881:3790 197.12721:12709 198.08894:39306 199.0712:52328 199.10809:14574 200.10329:23784 200.14061:40988 201.08652:13713 201.12428:122282

					202.08257:30427 202.12088:5249 205.09709:22483 205.13297:13833 207.11449:38001<br>208.11028:6057 209.09323:13921 211.10632:11594 212.10481:48334 213.08665:36553<br>213.16043:14141 215.13995:7125 216.09789:164721 217.08037:12045 217.10159:16454<br>217.13452:60885 218.1368:5085 219.11172:4243 221.09064:5134 222.12404:19887<br>222.16081:17790 223.07121:5841 225.12335:11224 226.08427:4100 226.11975:7108<br>226.15501:91509 227.15848:5075 228.13606:23103 229.11882:29716 230.11241:51393<br>231.09576:5278 231.14908:4098 233.16531:17147 234.12367:24389 235.10933:26521<br>238.12234:5791 239.14673:11379 240.09657:9165 240.1356:57885 240.17068:4599<br>241.08025:58572 241.1373:6112 242.08116:5828 242.14774:22115 243.17807:8450<br>244.13034:3760 245.12849:44012 246.12498:13713 247.10771:20439 249.12206:5705<br>251.10281:19315 252.13623:12715 256.09323:30207 256.1282:4005 257.16153:13183<br>257.19727:24529 258.14615:6386 258.20123:4723 259.08859:35723 262.11719:12476<br>264.13281:22276 266.15173:20359 267.14697:16003 270.14581:41324 271.14615:3793<br>274.10464:13632 276.13452:45387 276.16934:27115 277.11633:5894 282.10919:3823<br>283.14563:79254 284.12421:6087 284.15149:7211 285.15811:4713 286.13794:5710<br>288.1546:54034 289.15927:6073 292.12924:6152 293.16431:10987 294.14551:25726<br>295.10684:5791 297.15543:30381 300.19119:15103 301.15063:25033 302.15417:3893<br>304.13208:19237 305.16525:3565 310.10336:13344 311.1366:38737 311.17303:35321<br>312.11752:45057 313.11874:12117 315.16702:22032 316.13043:6585 321.13068:5030<br>323.1376:12280 323.17065:15205 325.18567:4163 328.11398:12695 329.1423:14191<br>329.18195:19248 331.12775:4298 334.14267:11845 336.23792:6554 337.18723:4864<br>339.1691:3790 341.18036:18470 342.14468:4421 349.19025:11016 351.16656:3623<br>354.24997:25479 355.16354:4617 355.25244:4002 359.17166:20667 362.13306:4300<br>369.18204:13032 371.27646:3972 375.20187:5896 377.181:6507 379.16226:32880<br>381.1795:5924 382.2121:3954 385.23358:35853 385.73721:16857 386.24066:4296<br>386.68018:12395 387.18549:14305 392.19766:14220 398.20322:18752 410.24109:4144

					412.17868:5619 416.216:6845 417.21643:5349 423.155:5119 426.20114:14366 439.20056:13088 440.1799:15912 441.16168:22443 444.21014:3967 455.22592:3843 457.28711:3410 464.29471:16112 465.28308:4221 467.70065:6749 468.19989:4697 473.23648:4931 476.21866:11375 476.71185:10905 477.20581:3915 478.2258:3858 482.31058:34863 483.31461:5150 485.21326:30174 485.71179:14130 494.22833:15600 498.23315:3925 499.28958:4602 500.28568:4373 509.2012:5821 510.19434:3561 520.28754:6556 522.24023:5195 527.21045:3919 528.19922:5521 540.2486:4252 541.76422:13282 542.75824:4245 544.29657:11138 545.2251:11238 555.23767:4531 597.33295:11677 624.20996:9846 641.24847:36077 642.25226:14383 643.24133:3723 648.39349:3480 650.29236:6735 650.40247:10428 659.25464:29824 659.86475:6634 660.26685:5038 706.36346:4077 710.42261:4862 717.4057:4471 719.41461:3939 730.39093:5943 737.31036:6748 754.34729:6125 755.33063:36884 756.33582:12082 769.44501:37491 770.45813:14723 772.3479:15001 789.92566:5028 798.43732:4044 806.41364:3690 847.44403:5860 884.4837:3643 903.49573:3850 936.49133:5138 937.68842:4901 943.48846:3785
3-(sulfooxy)benzoic acid	6.557	216.97964	[M-H2O-H]-	216.98122	59.01237:2829 69.11478:7116 69.1186:2301 71.04839:20541 72.93665:1826 85.02798:2107 87.92345:13081 88.03864:24636 90.94714:1947 99.92403:9844 100.93201:2679 103.91867:24144 104.92642:6012 105.93363:2551 106.94178:2364 114.94746:75894 115.91911:28694 116.89816:2277 116.92702:83482 117.92743:3531 120.95291:2240 123.94438:6192 125.87076:1867 127.86858:30388 128.99915:114423 130.94257:3391 132.95795:5586 142.97876:7007 146.08179:10047 146.95961:15585 148.94818:9464 158.93774:7020 159.85956:25485 160.98895:1772 170.97389:64016 172.86174:9233 172.98901:629727 173.99245:32201 175.93941:5825 190.87115:2023 198.97095:1719 199.85004:15356 200.85016:1803 205.8609:3296 205.98703:5874 216.85207:23470 216.97943:35157 217.03072:2760 217.85181:3565 217.97995:3182 218.10129:11593

3-(2-Methylpropyl)pyridine	13.141	136.11218	[M+H]+	136.11263	54.03473:15915 67.05524:4409 67.78185:41406 68.05045:5354 81.04538:5473 81.07098:15063 91.05432:13301 93.07068:14078 95.06069:28983 95.08614:16604 96.05641:10847 96.06892:17887 96.84699:13116 97.06429:6462 107.07377:12359 109.01109:19232 109.07687:26085 110.07131:23194 119.03614:17859 121.08853:196528 121.10241:14479 122.09178:15817 136.02141:472823 136.06198:525598 136.07407:17284 136.08789:80222 136.11172:1321332 137.02516:34444 137.04576:54757 137.06551:13913 137.08174:79039 137.10661:99818 137.11575:110544
1,2-Cyclohexanediol, 4-[(3E)-5-hydroxy-1,5-dimethyl-3-hexen-1-yl]-1-methyl-	1.317	279.19162	[M+H]+	279.19302	58.06667:6424 60.08165:49151 68.94185:44753 86.09715:37848 102.092:8767 103.0397:42523 104.10738:144263 149.02402:34962 159.05157:26572 162.1123:2271704 163.11566:142624 164.03023:58339 177.06155:65009 178.06494:12525 180.90411:9260 203.03981:10689 219.08415:7074 220.06842:358027 221.01891:258068 221.06908:82150 279.09583:7322 280.0928:145215
Anthranilate	13.457	102.03436	[M+NH4]2+	102.034	56.96553:5485268 58.5056:307162 67.51063:1412005 69.74581:491281 70.0134:190232 76.37994:121673 76.51614:974998 79.01871:2105568 81.555:115009 82.97162:116042 85.02904:297594 88.02391:2401804 90.52679:547799 97.02895:158667 99.53197:526679 102.09202:156749
Icosa-2,4,6,8,10-pentaenoic acid	12.49	301.21701	[M-H2O-H]-	301.21732	57.0329:3152 59.01218:95770 67.05358:3225 69.03255:8783 83.048:2845 93.06886:3035 106.03916:3148 107.08455:6166 113.09522:1932 117.06853:9140 119.08486:3533 121.10062:1914 127.11069:7429 130.03912:1614 131.08475:1709 135.11696:3043 141.09073:1616 147.11696:1678 149.13106:6321 161.13237:1848 163.14751:10092 175.14745:2241 177.09152:1558 177.16307:8390 189.16441:5247 203.17863:102681 204.18173:9916 215.18065:5576 223.17131:8089 229.1946:14607 255.21272:3000

					257.22766:345568 258.23218:42907 271.20532:8383 275.10593:2090 283.20432:7995 301.21515:532264 302.11447:1676 302.21887:69281
Hyodeoxycholic acid	12.204	391.28552	[M-H]-	391.28537	67.79976:10570 67.80347:54798 96.83882:20142 96.95824:23039 152.99419:11193 255.23151:71311 327.27017:114614 329.28375:72684 343.26144:39509 345.28189:354600 346.28009:45029 347.29285:103685 348.29553:11458 355.26605:100493 365.30487:175497 366.30725:22489 373.27557:56980 389.26962:30289 391.28778:16585340 392.28812:2461178
N-Nitrosomorpholine	13.028	117.06625	[M+H]+	117.06588	55.05516:16334 57.04556:21390 58.02977:5663 58.06585:57535 59.06127:11194 59.07378:76782 67.66557:6990 67.66979:32281 71.06918:11123 72.0451:9688 72.08168:134377 73.06555:2936 75.05591:181228 76.05933:2423 95.0501:4779 96.84972:5556 100.07665:10339 100.11275:4530 105.04557:2490 117.02232:4960 117.06555:4250 117.1028:4196 118.06536:13897 118.08606:375700 118.12379:2849
Korseveriline	11.782	454.32828	[M+H-H2O]+	454.32913	57.03444:17633 57.07082:61179 58.06589:37686 60.08159:290242 69.68064:21278 71.07371:33294 71.08629:65993 75.04473:95674 85.10203:34549 86.09712:725001 87.10064:10729 98.98515:11837 104.10736:6253010 105.1108:185216 124.99975:405010 184.07431:1011096 185.07805:30007 271.2648:7037 436.32538:9353 454.32474:1590742 455.33649:248024
PC(16:0/20:5)	13.078	808.58356	[M+H]+	808.58508	57.03442:18990 57.07092:185955 58.06665:63055 59.05015:78423 60.08162:308386 67.05524:283574 69.07059:168299 71.07371:249329 71.08617:179535 79.05463:66497 81.07032:345160 82.07361:15873 83.08644:166996 85.10201:108776 86.09715:3084822 87.10052:114451 89.05968:15787 91.05428:21252 93.07059:89217 95.08614:364259 97.10143:129340 98.98512:51640 99.0814:16683 104.10713:139659 105.07104:14548 107.08633:43445 109.10179:164048 111.11686:64347 117.09191:13690 121.10091:75479 123.11671:96772 124.99973:625293 135.1171:49767 137.13243:49204 146.98158:4161858 147.98558:85483 148.98599:13928 151.14789:20483 163.0145:44696

					163.1488:13789 164.99275:80928 175.1468:14206 184.07433:14034822 185.07834:480935 188.00888:136760 265.25217:44458 465.23273:88518 466.23639:24240 469.27786:16509 502.32953:21788 506.35797:15771 603.52881:508913 604.54034:157960 623.50061:19847 624.50586:22162 625.51379:1741486 626.52228:536367 748.50616:21658 749.51721:1533982 750.51099:494203 808.59058:2355863 809.59485:849333
5-Butyl-2-methylpyridine	12.333	150.12802	[M+H]+	150.12773	56.05019:5686 71.0368:1900 89.06048:11927 95.08613:2383 104.05299:5194 105.07006:2450 108.04496:2199 109.04779:6370 109.07696:2613 111.07915:5865 121.08842:18980 122.06107:2724 123.08167:1949 123.09203:2570 127.05808:5507 133.0309:1948 134.09665:2190 135.10355:11348 142.52908:1999 150.03676:31833 150.05527:15295 150.07686:13482 150.09081:14931 150.12746:1044634 151.05609:4613 151.06139:33981 151.08629:18726 151.09862:8433 151.13327:67450
1-Ethylpyrrolidine	13.427	100.11257	[M+H]+	100.11208	51.25271:6338 53.58773:7738 55.01876:7106 55.05521:310010 56.05027:47903 57.05857:8384 58.02977:11088 58.06584:30993 59.06131:280083 59.93129:12707 60.04586:5958 66.11333:5923 68.92666:9445 68.92992:37442 70.06621:21504 71.02999:15264 72.0451:29936 72.08171:269671 78.03793:10785 82.0659:42497 82.24175:7038 83.02469:7797 83.08643:16011 85.08929:11711 100.02445:8027 100.03963:12501 100.05103:8181 100.07667:390726 100.11275:748992 101.03494:46747 101.0715:15798 101.07921:9192 101.11584:26950 101.65221:7377 102.1811:6193
Glycocholate	10.087	488.29785	[M+Na]+	488.29822	55.05514:22730 67.05523:21214 67.78622:173671 69.07068:23665 76.0399:764041 79.05461:21874 81.07041:110651 83.0864:22160 85.06543:131916 93.07062:77000 95.08627:134885 105.07003:105571 107.08638:158466 109.10171:84610 114.05556:22270 119.08549:116764 121.1014:86328 123.08035:48713 123.11791:40981 125.09655:17073 131.08661:91626 133.1008:144794 135.11697:101361 137.09592:20946 143.08595:75094 145.1019:117962 147.11694:158471 149.09647:45812 149.13274:21844 155.08643:20170 156.06552:24685 157.10075:140176 158.08191:473280

					159.11649:230807 161.13242:129037 163.1113:24785 169.10185:49646 171.11769:105925 172.09628:37871 173.13264:95075 175.11168:56879 175.14961:75841 177.12663:20617 183.11653:78831 184.0981:64246 185.09715:49542 185.13316:131314 187.11252:44842 187.14893:68934 189.12643:54986 189.16344:22367 191.14296:19909 195.11632:38024 197.1328:94185 198.11266:78817 199.1481:330748 201.16495:98359 203.14413:23603 209.13338:639508 210.11209:39874 210.13519:64478 211.14755:165631 212.12823:43616 212.14874:29218 213.16348:477408 214.16759:53534 215.17828:76101 217.1593:16891 223.14728:110961 224.12721:24988 225.16516:183926 227.14259:564620 227.17714:283007 228.14568:44162 229.15887:120068 231.1758:43517 237.16388:112315 239.17854:87439 241.15881:116517 241.19409:170723 243.17429:107265 245.1902:18731 253.19521:26440 255.17393:93282 263.17899:57676 264.15756:21513 265.19485:18107 277.19516:64870 279.21075:16071 281.19028:25633 293.22629:93684 295.20789:103186 295.24158:137321 302.17416:50877 304.19186:69169 309.22485:22179 309.2558:81579 318.20905:63250 319.2399:959071 320.2485:144636 337.25192:2103975 338.25858:285460 355.2648:132731 370.24063:21037 394.27606:52711 412.2821:3577256 413.29202:623135 430.29456:649345 431.29559:107189 448.30508:62474 467.41312:19065
4,1-Benzoxazepine	7.068	146.06001	[M+H]+	146.06004	56.05019:2230 64.92831:6848 69.03423:2095 69.19827:5745 71.04986:5039 81.0343:2890 82.06596:14766 83.04979:5178 84.08146:7407 86.06078:2665 91.05511:23112 100.04816:7876 100.07666:67258 100.11272:7340 101.07919:2010 103.05458:2742 110.06027:2614 117.05832:6110 118.06531:72280 119.0694:5931 123.9645:41274 128.04997:223926 128.0706:7848 129.05353:20847 146.05986:902040 146.07993:21293 146.11678:7151 146.97993:6720 147.04417:2746 147.06488:85620 147.09174:14929

6-Hydroxy-3,4-dihydro-2(1H)-quinolinone	13.461	164.06711	[M+CH3OH+H]+	164.0706	58.9989:26977 69.43143:81206 72.93777:124898 82.01449:1009799 83.01536:46355 90.94813:39184 100.0245:3571101 101.02621:318645 113.96442:657760 118.03501:1688977 119.03613:165714 123.04028:783045 124.04173:55126 131.97508:279908 140.97168:124236 141.05104:415423 142.05261:28308 154.99129:43600 164.04393:29982 165.06503:37843
PFSM-perfluoroalkyl_sulfonamide	3.797	247.96185	[M-H]-	247.96217	52.94288:1259 57.19995:1502 71.03967:2060 87.92345:17107 89.93967:1485 90.93234:1714 90.94715:2531 102.92194:1598 103.91848:130744 104.92645:27079 105.93468:5093 106.9418:8459 107.9496:15042 112.98356:1893 117.92744:1847 123.94438:31844 130.94257:3208 136.90878:1558 144.95786:1711 146.97327:4868 151.94052:17913 157.97672:10711 158.97377:11548 160.95216:3052 174.96738:1956 175.97687:17797 180.06554:9517 189.95659:2976 191.97137:6012 201.88002:1569 201.95349:2657 202.96487:17017 203.97191:23535 238.76068:1426 247.96002:10205
beta-D-3-Ribofuranosyluric acid	6.292	301.07816	[2M+H]+	301.07843	55.0188:23176 57.03455:50110 57.07042:3489 59.05007:3806 61.02905:13457 69.03443:27680 69.40894:13219 71.01352:11088 73.029:45917 85.02922:41272 87.04518:11271 97.02913:44728 115.03933:57841 126.03062:32526 126.05483:4044 133.04987:80705 141.04112:21719 151.0863:26333 152.00992:14307 169.0352:1960152 170.01909:17156 170.03801:76863 187.04703:4948 198.0889:6885 210.06305:3725 211.04533:4926 235.04744:5592 283.12772:3754 301.07626:11046 301.14069:16534
Prolylhydroxyproline	1.903	229.11826	[M+H]+	229.11829	70.06621:2920672 71.0692:96456 86.06079:30589 103.02483:6609 115.96458:7920 116.07125:6092 131.01961:10054 132.0656:725309 133.06903:37811 149.02919:8347 167.03957:6445 175.97643:6458 182.99208:18231 201.00281:6629 210.9843:20208 228.98473:23588 229.11951:1262149 229.1557:38786 230.11243:27082 230.12236:114078
Indoxyl Sulfate	8.029	212.00156	[M-H]-	212.00232	79.95584:1366680 80.96344:1223784 81.96269:7218 92.04877:66132 118.0283:19982 120.04337:15908 129.05464:21215 132.04375:381924 133.04668:21027 212.00108:2470625 213.00597:187075

PC(18:1/18:2)	13.358	784.58875	[M+H]+	784.58508	56.05056:25462 57.07079:22041 58.0637:36175 58.06664:160495 59.05003:56715 59.07457:55328 60.08161:901745 61.08511:16548 67.05521:27172 69.07066:23463 71.07362:197898 71.08618:18250 81.07093:51916 83.08636:23917 85.10188:20389 86.09712:3675950 87.10048:147395 95.08607:29197 97.10143:17334 98.98455:122147 104.10731:382077 124.99968:1536929 146.98154:431623 147.98544:19055 163.01645:15940 184.07423:41384248 185.07812:1646706 186.07761:217324 207.99924:15741 309.28146:46643 502.32947:58923 504.34:86175 522.35333:47948 579.52008:63743 600.50226:46479 601.50635:159706 602.50305:16771 724.51068:21682 725.48987:140634 737.46216:17555 784.58466:1590588 785.58655:548413
1,10-Epoxygermacrone	12.473	235.1692	[M+H]+	235.1698	57.0708:406182 58.07421:11718 69.67623:3247 69.67954:6951 86.09713:2889 103.03968:2780 123.04411:21348 179.10609:546673 180.11047:62467 219.13947:10152 235.17041:2962493 235.21838:17879 236.17245:387794 236.23769:29575
Bicifadine	12.198	174.1277	[M+CH3OH+H]+	174.12773	57.03444:3240 57.07092:13261 65.03959:1579 69.03477:1592 71.02998:2526 72.08164:2594 73.02941:1859 81.07029:3029 82.06589:2311 84.04491:2805 84.08145:7603 86.06078:6226 86.09714:7043 87.0445:10240 91.05514:2682 95.08612:2116 100.07568:3532 106.06584:9844 112.07631:22279 114.05557:3536 114.09139:2000 118.0651:47186 119.06815:1930 119.08541:3196 128.07074:16110 128.10757:14798 130.06616:2640 130.08586:5550 131.07365:5025 132.08139:176255 133.08475:9406 133.10077:2183 142.07828:1810 144.08095:15707 145.08865:28555 146.05983:3320 146.0816:14617 146.09666:10006 147.05431:1770 147.1169:2268 156.06554:6418 157.09695:3117 158.09682:11437 159.1049:43535 174.05502:9051 174.0768:16920 174.12692:1241499 175.05019:2096 175.12054:206889
PC(14:0/16:1)	12.296	704.52313	[M+H]+	704.52246	55.05513:5972 57.0708:17640 58.0633:6234 58.06665:34290 59.05005:13456 59.07458:12132 60.08161:158011 67.05522:17677 69.0705:28825 71.07376:29553 71.0862:14510 81.07011:29478 83.08638:21565 85.10191:7797 86.09711:713618

					87.10063:20662 89.06049:7202 95.08623:28819 96.84244:7120 97.10146:17061 98.98514:22335 104.10744:77198 109.10178:19326 111.11798:5362 121.1011:8080 124.99971:310113 135.11713:6228 149.13272:5428 184.07426:8402226 185.0782:258743 225.59825:6079 519.44244:35817 520.44519:6886 535.48096:13086 549.48663:52270 550.49115:7451 563.50659:62329 564.5097:6946 704.51575:260446 705.52747:81468
2-Acetolactate	6.28	131.03343	[M-H]-	131.03444	59.01233:3319 69.03312:103440 85.02793:4023 85.06456:18793 87.04339:1054852 88.04696:39271 102.98726:9324 113.02342:89160 114.026:3483 131.03355:243715 131.07019:12569 132.03644:11838 132.05515:7202
Milrinone	0.888	210.06445	[M-H]-	210.06732	71.01808:6456 87.00721:8613 92.92619:868730 94.9238:881779 105.23087:5176 127.86848:72347 129.86635:27513 131.08069:854597 132.08476:43229 152.88301:22988 152.99384:64454 159.85774:27610 161.85651:6941 164.8343:25281 165.8425:24536 173.10342:7180 174.0863:420463 175.09035:22109 211.00046:56483 211.03246:9584
Tryptophan	8.369	227.07623	[M+NH4] 2+	227.07912	56.0506:2203 56.96589:3988 69.0707:2153 70.06625:29886 84.04493:458141 85.04836:19088 91.05515:2444 100.11274:9130 116.07115:43670 119.03122:6514 120.08126:32748 128.0361:30932 129.0661:3580 137.04065:48268 137.5416:3010 139.54434:12522 140.002:2510 146.05507:22061 146.54657:2367 148.55038:13772 150.54703:2637 154.01863:11236 154.04945:2498 157.563:4501 158.05373:2279 160.54399:2274 162.05554:2215 172.02759:18241 181.09813:2370 181.95171:3055 184.02948:4244 186.95731:2376 190.03955:63045 191.0428:3486 199.14551:2173 200.09241:3378 201.57159:7109 202.03926:3569 206.56158:2748 208.04999:40762 209.05319:2645 209.16504:3095 210.11215:2529 213.05487:12253 215.56822:60400 216.07086:9729 224.57306:31209 225.07542:9296 227.07744:28994 227.13986:16020 227.17809:9628 227.57805:7990 228.06725:4142 228.12192:14075 228.23341:36224
7-Hydroxy-6-methyl-8-	6.263	327.09494	[M-H2O-H]-	327.09409	51.21125:1200 53.55797:1311 57.04067:1323 66.25823:1199 69.28676:3153 69.29004:2972 71.01183:1737 74.52779:1523 75.00668:3005 78.98426:2495 85.02798:1753 102.94695:24131 103.91842:1578 113.02341:1666 115.05032:2632

ribityl lumazine					125.92433:1417 136.90874:1819 144.02873:1777 146.93796:35549 149.04492:250546 150.04903:8364 190.92606:19775 212.11235:5358 214.98921:1495 219.67154:1318 267.09708:1765 281.08728:2335 327.07391:11683
D-Arabinose	0.96	149.04417	[M-H]-	149.045	57.03287:2995 59.01246:91554 69.03306:8615 71.01178:7080 72.99173:22938 73.02766:7746 75.00739:202337 80.41888:1646 83.01208:1778 85.02811:16436 87.00714:3224 89.02281:2116 101.0224:6875 103.03881:1971 113.02219:1778 120.95282:2840 121.02855:3413 129.01701:1693 131.03355:69208 132.03642:1754 149.02396:6781 149.04465:231346 150.05602:20918
Uracil	6.21	113.03498	[M+H]+	113.03458	58.06626:10197 60.05645:10080 67.05525:42114 68.05045:144627 69.07074:74039 69.86085:38567 70.02955:986719 70.06621:16064 71.94086:143140 72.0451:15244 72.08166:43769 72.93782:607940 73.04778:11890 79.0546:88849 85.08482:18349 86.06087:88995 90.9481:220436 91.05845:32862 95.0246:79225 95.05009:13500 96.00832:515174 96.04482:45569 96.08143:55532 97.06612:13370 108.9593:19432 113.02349:127867 113.03546:2041245 113.06055:55413 113.07224:83764 113.07994:34748 113.96452:118849 114.03827:54670 114.05561:284290 114.06606:102813 114.09142:783783 114.12841:12535
PC(16:0/18:1)	12.367	804.57629	[M-H]-	804.57599	55.05512:185836 57.07079:92129 58.06371:121225 58.06664:521554 60.08164:2931016 67.05521:208080 69.07066:287455 71.07362:533194 81.07025:285430 83.08636:292333 86.09715:11695178 87.10043:510741 95.086:387669 97.10144:100500 98.98464:438968 104.10741:1450208 109.10176:81877 121.10108:236384 124.99973:5709500 135.1171:94779 184.07431:142649568 185.07584:6907550 186.07785:1150059 339.2876:96801 496.34:240273 575.50354:1093706 576.50208:295616 577.5274:89894 760.57654:4236432 761.59027:1258071
Aerugine	2.776	210.06114	[M+NH4]+	210.05827	70.06639:8679 71.01524:1605 83.0498:1234 84.08147:2247 85.06548:2054 86.06081:1946 88.07635:2528 88.65302:1266 95.08615:1318 101.86639:1496 106.08682:14375 114.0556:2088 118.08605:2244 119.07188:1453 125.02365:1631

					126.05486:2186 129.01894:68604 130.0226:2457 136.11172:1986 139.0034:13459 139.05167:1326 146.0582:1255 147.02911:40839 148.07439:1220 150.09259:1695 157.01334:40697 160.82993:1188 164.06984:2208 164.1078:4827 165.10124:2056 166.08546:1495 175.02371:11195 182.1172:1925 186.96703:1905 192.06619:2583 192.10153:2890 193.03429:1367 193.0979:1247 209.93896:2764 210.07751:8245 210.11217:26898 210.14973:2185 211.10832:23781 211.14423:15949
Thymidine	6.314	287.08801	[M+FA-H]-	287.0885	62.40594:2136 68.89021:2149 70.3395:3861 90.7836:2154 95.56002:2071 101.10527:2058 109.03965:26262 118.06471:2187 125.0338:54590 151.04936:118656 152.05261:4697 176.27176:2448 188.13464:2233 198.07675:5586 214.09198:2386 230.75854:3021 241.08318:667297 242.08386:49872 242.17683:2833
4-(3-Hydroxybutyl)-3,3,5-trimethylcyclohexanone	12.183	211.16965	[M-H]-	211.1698	65.01311:4688 66.0078:1572 70.03784:1991 70.04173:2324 89.01253:2710 90.00767:1704 91.02782:1973 97.06431:5418 99.92404:1665 106.03921:18214 111.07959:6065 113.09526:1641 115.07507:13801 116.02324:1626 125.92704:1563 129.09116:12229 130.03917:2130 142.0397:2043 145.0506:2239 154.09827:10995 157.05028:10831 158.03513:1853 167.10555:2012 167.14243:2485 168.114:8894 169.05028:2307 171.06734:2255 183.17319:9201 184.04922:3128 184.06429:8786 185.04544:11462 185.08134:2598 210.96852:6327 211.05864:5154 211.16917:323729 212.05736:10482 212.0919:2762 212.17422:34375
alpha-D-Glucose	0.886	179.05489	[M-H]-	179.05612	57.10406:3533 59.01205:158863 60.75393:3949 71.01191:75492 76.54308:4139 76.76714:3891 80.91508:4068 85.02799:4834 89.02288:69421 101.02248:18855 113.02229:4449 119.03315:8982 141.86647:15893 143.86333:24496 161.87408:8196 178.81395:58288 178.83209:18553 179.84116:7789
Urobilin	8.724	591.31689	[M+H]+	591.31769	87.04438:11880 89.06072:65091 124.07574:2469 133.08633:25591 136.07552:4539 164.06998:46917 165.07501:3044 177.1132:7429 180.10146:21692 211.12379:7172 225.13939:4593 256.12045:2303 257.12637:3321 269.12897:13041 271.14194:2994 283.14728:15738 284.15152:8879 299.13892:15399 301.15546:4534 303.16794:2883

					330.15842:2216 342.15671:7110 343.16745:3463888 344.17047:399417 345.17178:6999 394.20993:16538 438.198:15234 449.2337:7097 450.2287:3284 452.21951:2538 466.23633:240397 467.24069:187505 468.24744:31331 591.31482:1172885 592.32495:263797
5-Hydroxymaltol	4.626	143.03406	[M+H]+	143.03444	55.01875:19385 58.06583:45724 69.03434:60577 69.76735:9108 69.77011:18143 70.06564:41557 71.04986:36607 84.04491:40460 84.08154:1266400 84.96065:13415 86.06078:11433 97.02893:17483 98.0605:125337 98.09734:163984 99.04498:9400 115.08723:11275 116.97171:76145 117.98022:14268 125.02348:85424 126.09108:41112 143.03392:2026267 144.03696:113164 144.06647:50068 144.10251:878085 144.13866:10650
Butyrylcarnitine	6.478	232.15446	[M+H]+	232.15428	57.03444:9746 60.08165:592448 69.48412:32191 71.04984:65500 85.02905:5818215 86.03275:182577 144.10254:125419 163.97617:13674 164.97855:7964 173.08138:1291753 174.08359:89263 208.95573:8566 232.15579:2782246 233.15895:333210
PC(18:1/14:0)	12.003	732.55286	[M+H]+	732.55377	55.05512:101140 56.05056:55501 57.07079:114747 58.06371:71335 58.06664:357347 59.07414:60225 60.08163:1722632 67.05519:251577 69.07069:320729 71.07367:400366 71.08619:54273 79.0546:108070 81.07039:350826 83.08647:313878 86.09714:7359620 87.10027:188898 88.04024:53863 93.07063:121385 95.08615:375614 97.1013:229892 98.98438:195073 104.10735:806439 107.08632:102688 109.10176:150706 111.11685:53565 121.10106:255300 123.11787:64128 124.99972:3271991 135.11691:216615 149.13269:42450 184.07434:79684040 185.07585:2893072 186.07767:340375 237.22301:182883 311.25635:143037 324.25333:127621 476.31723:41674 496.34:61842 547.47729:1432842 548.47565:430856 732.5495:2179548 733.54767:658343

Licochalcone C	10.211	337.14664	[M-H]-	337.14453	67.80773:4855 67.81197:13464 78.95761:188025 96.83706:2520 96.84338:5264 96.96821:449802 100.95222:1748 102.94666:42377 120.38843:1504 144.94131:2097 145.93944:1809 146.93759:80055 190.92853:25389 191.93102:1666 253.27499:1523 337.14108:81703 337.20541:13151 338.14752:5147
Psicose	1.293	203.05247	[M+Na]+	203.05263	50.38383:205071 54.43894:179508 56.17778:151460 57.31079:153260 60.50656:172652 66.49635:149233 70.74415:310551 75.99521:156605 107.88008:159235 117.30979:187344 146.31133:175040 199.67967:181681 203.05353:23218056 204.05621:1005931
Bilirubin	9.518	585.27124	[M+H]+	585.27075	69.48904:18796 89.06048:22231 120.08126:19898 134.06125:10427 136.0755:19878 152.07028:24028 166.08737:21875 172.07481:18497 174.09195:156991 186.09225:7326 189.10153:89935 196.11264:15605 199.12405:7158 201.10274:7558 210.09181:25539 211.0847:41132 211.12376:9031 212.093:26719 212.10765:25762 212.12816:9531 213.1017:31333 215.116:7100 222.09172:29343 224.10883:53779 225.10358:182934 226.08748:39616 226.11005:56404 227.11942:290116 228.12273:27923 229.13254:10450 236.10657:38865 237.10103:8974 238.12546:30769 239.11848:111984 240.10361:37216 240.12479:24049 241.13374:177749 242.13831:9754 243.15019:30105 250.08696:18875 251.11826:30214 252.10204:7179 253.13309:34075 254.11852:17945 255.14706:31472 257.12958:29341 258.11087:45919 260.12823:7971 264.10062:33650 267.11368:587731 268.09781:46582 269.12888:101948 270.09894:34650 271.108:78752 271.14612:90677 272.11417:8020 273.12167:19943 273.16019:26716 279.11313:57100 281.1275:96460 282.1131:432675 283.11401:58821 284.11966:29261 285.12146:2037262 286.12866:358842 287.14117:845328 288.14557:101238 289.1546:7456 297.12207:152696 298.13007:26325 299.13818:3244730 300.14178:377374 301.15543:291880 302.16055:29544 326.14993:15137 327.15216:25016 355.14322:37531 373.15488:45685 376.16177:9224 383.16348:7843 387.17102:24371 390.17969:8911 394.17944:19217 395.16101:24751 396.1687:7147 397.17447:32906 412.18591:81648

					420.17401:22421 421.17236:7200 422.1907:19008 432.17142:26495 433.17691:34100 434.1889:31115 435.19421:82572 436.20276:77516 439.20926:7217 445.17953:29036 448.1969:33276 449.19873:29842 450.21155:34950 451.21783:20128 456.1936:78836 457.18506:17943 459.19424:8197 462.21548:37459 463.21472:19249 467.22134:26881 472.20203:65140 473.20715:9112 475.18542:17908 477.20578:8781 480.19568:20077 490.20953:190431 491.20831:45022 493.19827:9774 494.20743:9911 495.21887:122604 496.22168:32005 497.22208:7180 504.22482:24925 505.22498:8087 507.23438:20835 508.22113:134456 509.23392:53924 512.24426:18982 522.23932:36692 524.26025:57869 525.25262:22420 532.22888:62745 533.23157:8922 540.24683:37386 550.23431:174490 551.24011:43082 567.25653:45628 568.24207:1640423 569.24585:346261 585.26501:2257289 586.27362:494182
PC(14:0/14:0)	13.322	678.50818	[M+H]+	678.50677	57.03414:19043 57.0704:13907 58.06665:19147 59.05022:100209 59.07415:6295 60.05334:4270 60.08176:109230 67.0547:4554 71.07362:19873 71.0862:11909 81.03151:12890 81.07027:10482 83.08566:9652 86.09726:470550 87.04459:19131 87.10063:15399 89.06049:8824 95.0861:5572 98.98514:14076 99.08047:13883 104.10747:62387 108.08648:9124 109.10178:5972 115.07549:13680 117.09095:23334 121.1011:4411 124.99982:208168 126.00246:3978 157.12306:3823 175.13365:11616 184.0743:5773175 185.07584:183588 186.07785:3843 309.28156:9261 422.27307:3884 493.42798:29941 496.34009:12438 678.51276:158957 679.52252:41114
Troxilin B3	11.169	353.23276	[M-H]-	353.23279	57.0329:2314 58.00431:1581 59.01253:9510 69.03315:11003 73.0277:1833 81.14057:1499 84.2914:1682 92.66653:1366 96.95825:5149 97.06428:2604 99.92401:2668 116.92691:6632 125.05901:1482 126.98981:1333 134.07288:6370 137.26953:1321 139.11057:30887 147.07974:3141 149.0948:1847 149.11725:1323 151.11057:16555 152.9942:1863 153.09109:53222 153.12711:6337 154.09314:1739 155.10681:1678 161.09552:5448 165.09149:6305 167.1055:6356 169.1232:6901 177.09109:19663 181.08499:3106 183.10071:46172 184.10602:1511 185.11472:2152

					195.10178:81783 196.10603:2810 206.09357:7281 213.11205:7136 221.0802:3159 255.21274:5613 273.22443:1514 317.2103:3118 335.22345:16582 353.13565:2936 353.23615:98170 354.23734:12492
Taurodeoxycholic acid	9.758	498.29031	[M-H]-	498.28943	68.83926:25772 69.95557:4387 72.71497:3508 89.80788:3992 101.7505:3842 103.46969:3743 124.00581:5147 142.71632:3661 193.05803:4168 253.03792:5714 411.1748:4732 498.29086:5214144 499.29446:792353
delta-Valerobetaine	1.406	160.13356	[M+H]+	160.13318	55.05512:1119452 58.06667:103823 59.04999:167869 60.0816:4521424 61.08515:85358 69.15734:98501 83.04967:679259 84.05367:32612 101.05994:6305214 102.06332:230880 109.976:28747 113.96436:106829 114.09139:35295 132.08138:25879 160.03395:489104 160.13383:18457714 161.13635:1196246
2-Naphthol-6-sulfonic acid	10.642	223.00594	[M-H]-	223.00702	69.81378:6058 79.95583:10799 116.92694:5451 123.89989:17386 125.89922:14472 143.00633:6857 143.04851:470942 144.05171:32998 179.10684:7505 187.00186:5728 194.9857:2119 222.98178:59882 223.00703:309898 223.0955:2657 223.7981:14624 223.98625:3907 224.0085:28816
Dihydroergotamine	10.648	582.27032	[M-H]-	582.27209	69.78168:13191 69.785:7317 122.0593:7511 213.1032:9346 239.11826:96627 240.12059:77335 241.13303:23925 242.13763:30892 285.12201:184086 286.12885:154618 287.1275:7125 297.24341:44379 494.26468:9412 514.2016:42336 514.27905:6365050 515.28687:1205607 518.23956:7179 522.31403:13228 536.25177:350136 537.25476:85486 538.24554:45171 582.26434:330197 583.28094:63175
Leukotriene C4	6.372	624.29858	[M-H]-	624.29602	59.50774:1532 60.76332:1467 69.75845:4865 74.02322:12270 97.03886:1679 100.00265:2091 110.02329:13122 125.07089:2760 129.06569:6957 143.08072:13800 153.06604:14543 164.10602:2074 169.09811:10410 187.07187:32800 187.10851:1939 248.10637:2352 266.11349:44353 282.14496:3412 284.1286:8472 436.22327:28190 437.21985:2339 454.19739:10054 546.23553:1507 611.37598:1773 624.2948:199903 625.30225:33676

3-Hydroxyisovalerylcarnitine	6.249	262.16504	[M+H]+	262.16544	59.05002:39086 60.08165:117754 68.73177:21164 70.0662:9082 83.04979:5223 84.04491:3922 84.08146:5983 85.02905:1112545 86.03281:36051 88.03951:3214 101.05992:12294 103.0397:11534 144.10254:40942 145.04901:310887 146.05363:20918 159.0649:4814 167.3389:3109 176.06944:4035 185.08075:55725 203.09195:13861 204.12177:18071 216.0347:4764 217.09563:3409 239.89569:3782 262.16574:1172102 263.09808:10314 263.16727:134375
4-Oxo-L-proline	12.156	130.05009	[M+H]+	130.04987	50.19206:5460 56.05022:42488 70.61115:8073 71.04987:6114 84.04494:1895737 84.08143:357615 85.04827:70931 85.06548:28343 85.08482:9749 91.05515:12791 95.31784:5701 102.05582:8619 112.07631:49224 115.16222:5001 130.0507:270167 130.06479:12501 130.08589:222733 131.05376:11301 131.08507:5632
LPC (O-18:0)	12.227	510.3931	[M+H]+	510.39178	57.03444:13507 57.07084:35832 58.06594:17889 59.07459:3852 60.08164:144830 61.08469:3211 67.5996:19349 67.60329:22954 69.07068:3618 71.07368:19873 71.08627:36614 72.08163:3311 75.04466:33033 85.02901:26335 85.1021:21449 86.09718:357139 87.10064:11297 88.07632:3959 89.0605:4479 95.08611:3119 98.98421:5276 101.07146:3327 104.10741:3007643 105.11081:84824 109.10179:3150 124.99984:186175 184.07439:598183 185.07625:55740 186.07724:26468 228.23067:3258 313.27609:34019 492.33734:4231 510.39191:880640 511.39847:117766
LPE(18:1)	12.506	478.2941	[M-H]-	478.29391	78.95723:256939 96.84069:60874 96.95826:14718 133.73927:6704 140.01007:245392 152.99446:70283 196.03596:888942 197.04185:15415 208.14035:7027 214.04771:186696 225.8578:7561 281.24838:8564411 282.25296:846870 287.91824:6942 325.8212:7432 406.013:7005 417.24341:9757 478.29007:1985289 479.29324:310147
Gly-Tyr	3.139	237.08795	[M-H]-	237.08801	69.91902:2318 73.02773:5140 73.03897:6998 83.02292:1846 85.03915:6739 93.03313:2883 95.02369:3191 96.95856:12866 103.91844:6355 107.04886:3098 108.92191:3328 111.01862:74988 111.64916:1513 113.03368:5580 115.91901:2772 117.01824:17014 118.02102:1359 119.04934:8896 119.73462:1258 131.04515:16680 133.02779:5011 135.04256:1459 148.07619:1945 150.17989:1260 163.03867:7206

					163.59491:1374 164.91263:1469 165.05533:7231 174.05562:2200 176.07004:21805 178.04987:1500 180.06512:23289 193.09636:1871 193.89539:2624 198.33893:1436 201.06633:4925 209.0433:1716 216.45572:1359 219.0766:45000 219.98381:1620 220.07977:2192 236.96533:6583 237.08653:96290 237.87091:2118 238.02408:10954 238.08891:10278
Jasmine ketolactone	10.854	207.10144	[M-H]-	207.10211	59.01237:8804 65.01309:1998 66.03323:3222 69.8503:3171 73.02766:15071 81.03323:30978 87.92343:9899 99.92402:5889 101.02246:2428 103.91826:15040 106.04018:16771 107.03518:8022 109.02762:338245 110.03065:14112 115.91898:2526 118.99129:29058 122.03623:113227 123.94437:7570 125.02189:2430 150.03154:86807 151.03708:7637 161.04504:22997 162.98129:73005 163.11171:6255 177.05356:2617 179.1067:377448 180.10887:34530 189.09044:5669 191.06888:2804 192.0773:32223 206.97243:21929 207.10187:1381796 208.06348:8659 208.10623:130647
Taurocholic acid	10.585	498.28781	[M-H2O+H]+	498.28699	67.66399:41063 67.66821:13919 81.07099:6431 85.06548:14198 93.07069:4964 95.08614:11674 96.84428:16827 105.07008:6409 107.08639:19248 109.10183:5709 119.08543:3987 121.10116:5194 126.02274:154156 131.08507:11784 133.10078:5258 135.11719:5373 136.06255:25706 143.08589:5231 145.10196:19084 147.11693:14938 149.09653:4221 159.11661:25948 161.1321:7305 169.10191:4997 171.11772:12091 173.13315:5274 175.14911:4168 183.11659:14912 185.13304:17564 187.14899:7486 197.13257:4334 199.14771:35465 201.16508:7290 208.06488:61624 209.13347:46879 211.14717:16295 213.16379:34313 215.17899:11742 223.14719:5470 225.16193:11576 227.14264:64008 227.1769:30354 228.18498:3830 229.15901:11169 237.16353:5181 241.15874:6447 241.19426:19315 243.17461:12160 248.09612:3849 255.17467:6139 293.22638:7845 295.20319:6726 295.24167:13128 309.25592:6536 319.24023:91384 320.2485:15366 337.2522:248064 338.25259:33127 352.16122:4474 368.1886:5524 459.68021:4221 462.2713:442306 463.27261:63815 464.28549:4326 480.2756:35412 516.18152:6441

4-[5-[[4-[5-[Acetyl(hydroxy)amino]pentylamino]-4-oxobutanoyl]-hydroxyamino]pentylamino]-4-oxobutanoic acid	11.932	478.29208	[M+NH4]+	478.28711	55.05526:117659 57.03456:125426 57.07088:43549 62.06067:15634 67.05481:310840 69.07079:304915 69.43635:7674 69.44019:16561 70.07397:8348 71.04984:19168 71.08636:31817 79.05478:69426 81.07039:452281 82.07433:12342 83.04977:18481 83.08649:204224 85.06544:21268 85.10192:8192 86.0971:47903 91.05532:50817 93.0708:118035 95.08621:426839 96.08943:17107 97.06548:38865 97.10153:141898 99.08048:20063 105.0702:43558 107.08631:84180 109.10191:218025 111.08134:23026 111.11717:27163 113.09701:7113 119.08548:55188 121.10125:97067 123.08035:15889 123.11669:102549 125.09655:9062 133.10069:83543 135.11725:66573 137.09592:14559 137.13258:53252 139.11227:6550 147.11674:48150 149.13287:37839 151.11269:7463 151.14792:25329 153.12692:8502 155.01135:8107 161.1324:50681 163.01651:14227 163.14908:36267 175.14981:37841 177.16464:8675 181.16049:8091 184.07442:16003 189.16344:15095 198.05183:19468 216.06175:24253 245.22662:26449 263.23584:61542 306.28088:90766 337.2753:3460737 338.27628:526159 478.32614:88125 479.33871:7528
Ncgc00385798-01_C31H43N7O6_	12.26	608.31891	[M-H]-	608.32019	51.53184:1462 59.0498:1450 66.49675:1824 68.99397:2699 72.62983:1439 79.95517:1544 89.0229:38985 96.83796:8680 112.98357:439560 113.02115:2960 113.98798:5834 114.018:2144 114.15322:1411 201.35597:1662 226.00922:2072 255.23186:13268 279.2327:2904 480.31012:5253 504.30576:2496
Isopropylbeta-D-Glucopyranoside	1.585	221.07693	[M-H]-	221.07559	62.12358:1330 66.41811:1181 67.10517:1165 68.28365:1145 69.50883:2693 69.51212:3452 74.88791:1311 87.00719:2377 88.03849:5535 89.02287:7362 93.6558:1337 96.84065:1328 96.95826:1530 99.92402:2136 114.01798:2330 118.89898:2052 133.94489:1418 148.95329:44473 148.98933:16426 149.95494:1742 152.89375:1774 159.92482:1537 164.86639:1391 177.93497:7365 195.94467:1705 220.93881:9526 221.01158:38622 221.84303:30494

2-Ethyl-3-hydroxy-2,4,4-trimethyloxazolidine	1.968	160.1335	[M+H]+	160.13321	55.0551:427464 56.94305:14893 58.06625:16595 59.04995:80982 60.08159:1771490 61.08515:44506 69.07178:67361 72.93835:50221 83.04971:329058 86.09714:13618 90.9481:23994 97.96936:15136 101.05988:2530428 102.06364:80939 106.99289:19008 113.96436:367610 114.09135:140002 118.06534:60249 124.05873:14210 125.00358:76880 131.97478:117441 132.02039:108097 132.08138:19943 132.10153:17924 133.0076:37724 133.01634:66819 141.9595:16945 142.0863:26699 154.98941:20654 159.96867:18270 160.09819:191784 160.13371:7644742 161.00276:98081 161.13606:481294
9-methyl-8-{[3-(trifluoromethyl)phenyl]methylthio}hydropurin-6-one	6.675	339.05682	[M-H]-	339.05301	51.98779:1327 58.44847:1605 70.3625:1777 72.00746:2168 102.94769:1639 103.91844:3396 106.60484:1608 108.91328:2763 112.9836:38174 116.04961:10723 117.20083:1335 122.89236:8869 123.8999:7791 130.06448:1823 135.92351:2291 141.91158:15304 142.06378:1743 146.93777:6677 152.49649:1680 158.92445:6597 159.09155:10417 161.90346:44934 163.91872:23229 203.08212:224473 204.08562:16919 221.97777:7359 225.0645:151019 226.06749:11267 234.04494:1421 264.40991:1478 269.05368:1674 271.06979:120106 272.07162:7463 292.97879:125140 293.04852:22380 293.97971:13462 294.05692:1651 310.99081:30117 327.13046:1539 338.98627:2072
8-bromo-6-chloro-3-(4-hydroxyphenyl)-4-methylchromen-2-one	0.694	362.9408	[M-H2O-H]-	362.94299	68.37685:55992 96.84064:16525 102.9873:31815 112.98351:35733 114.98689:108835 130.98241:895149 131.98352:64636 158.97762:5172292 159.97832:432326 226.96594:1224458 227.96812:104494 294.95297:29579
7,7-dimethyl-1-{[(4-	0.947	307.11487	[M-H]-	307.11221	58.02831:15075 59.01211:33778 66.03323:2546 69.3404:7278 70.02782:6249 71.01237:16833 74.02267:2736 78.95698:6904 82.02834:95055 84.04401:111205

pyridylamino)sulfonyl]methyl}bicyclo[2.2.1]heptan-2-one					87.00733:202697 88.03857:33277 89.02311:28935 92.92614:24242 94.92374:144915 96.92042:54688 96.95861:32268 96.96822:28043 98.05981:19533 100.03875:7363 101.02259:50630 101.07063:5780 102.05399:7651 104.03407:3425 112.03845:17298 113.07027:42122 115.08544:3121 118.05019:3596 119.03326:14107 125.03382:3558 125.071:33069 126.05444:10638 127.04957:10045 128.03329:31646 130.049:28807 130.06024:25644 136.91635:3540 143.04524:6729 143.08102:107378 144.06482:7898 144.0845:3298 145.06061:207689 145.09698:8792 145.99803:28439 146.04489:97818 146.06323:3912 149.07065:3066 151.04941:4300 154.92377:25851 167.08064:12850 169.06105:12951 170.00908:9637 170.04489:95635 176.89949:3849 180.08615:239507 181.08965:8097 187.07216:825640 188.02058:58493 188.05647:17502 188.07359:33533 190.01616:3444 200.05656:16236 217.08209:34197 218.06497:15756 229.08228:2580 307.11676:29442 308.09772:13705
LPC(20:2)	12.756	548.37134	[M+CH3OH+H]+	548.37164	55.05514:5417 57.07081:5069 58.06372:2814 58.06666:12187 60.08163:91656 67.0552:19939 69.07072:18726 71.03622:4373 71.07371:14654 71.08621:4169 81.07029:23778 83.08626:16984 86.09713:313946 87.10064:9711 89.06051:3777 93.07066:8861 95.08606:27961 97.10147:9619 98.98515:5326 104.10735:1460575 105.11075:39211 107.08636:4703 109.1018:12838 121.10112:9029 123.11791:3018 124.99973:110151 135.11714:4736 137.61349:2545 163.01454:8432 184.0743:1886576 185.07796:47898 199.03621:3566 258.11102:27858 286.06:2577 365.30551:25077 471.28989:3804 530.35852:116193 531.36609:22542 548.36603:450315 549.37823:86619 549.48816:15149
13-Hydroxyoctadeca-9,12-dienoic acid	12.37	295.22787	[M-H]-	295.22787	62.86155:1567 71.02831:1257 85.72607:1342 88.61403:1568 106.0392:1623 113.09526:3407 140.15134:1604 171.1013:26650 179.14323:1604 181.12204:1404 195.13823:85269 196.13995:6334 216.54333:1306 233.18475:1364 251.23825:2588 259.20328:1347 277.21686:127089 278.22116:15845 295.22708:497063 296.23041:70072

[5-formyl-4,8-dihydroxy-3-(3-methoxy-3-oxoprop-1-en-2-yl)-8a-methyl-2,3,4,4a,5,6,7,8-octahydro-1H-naphthalen-2-yl] 3,4-dihydroxy-2-methylidenebutanoate	10.359	425.18118	[M-H]-	425.18173	57.03291:2100 59.01213:76651 67.8056:14911 67.80984:4893 71.01205:37708 72.99126:104788 73.02794:25230 83.01215:2918 85.02808:45104 87.00721:2593 89.02302:50932 95.0125:13572 96.83705:2173 96.84247:4530 99.00672:5754 101.02261:42070 103.00232:69656 103.03889:2579 113.02245:201664 114.02724:4249 129.01721:16517 131.03369:62198 133.01347:20272 149.04477:3081 157.01331:19091 163.02257:11273 175.02443:55079 176.02798:2273 193.03525:645067 194.03885:22980 249.14757:44755 250.15024:2147 425.17969:8169 425.26285:6643
Thymine	6.348	127.05053	[M+H]+	127.05018	53.03947:20613 54.03469:35457 55.01887:41448 55.05527:37174 56.05027:51462 57.04548:8001 68.93262:16587 69.07069:7704 71.04987:47384 81.03432:50534 81.04537:20659 82.02932:52071 82.06591:201932 84.04494:216749 84.08146:17322 86.06083:48824 96.0091:7441 99.04497:17277 99.05619:15180 100.07662:70157 109.0285:83100 109.04023:185091 110.02413:730380 110.06012:33206 111.02808:22224 127.03906:1374059 127.04993:1882834 127.08661:29988 127.12331:22791 128.04312:68695 128.05412:72867 128.07068:170672 128.1076:52592
1,2-Cyclohexanedicarboxylic	8.474	185.08057	[M-H]-	185.08194	57.03292:1414 59.01237:2707 65.01311:1593 69.91403:5304 74.02287:30034 75.02641:8042 81.06924:3113 83.02363:8144 87.92345:2085 91.02864:1717 95.04829:5467 97.96792:10763 116.92694:3102 121.05012:3001 121.06402:1671

acid, 4-methyl-					123.08006:208633 124.08304:14177 125.09486:3123 128.03317:1484 138.88843:2167 139.09358:1449 141.09087:148276 141.10985:7999 141.95786:2385 142.08627:5242 145.0506:1497 157.09718:2083 158.03552:10694 159.06706:2453 167.07071:7967 185.04552:7665 185.08133:275820 185.10043:75158 186.08362:16270 186.11412:11343
Glucuronic acid	7.754	193.03436	[M-H]-	193.03532	57.03292:1729 59.01214:82701 71.01205:50808 72.015:1507 72.99128:105013 73.02795:34806 75.00669:2091 81.04362:2027 83.01215:5691 83.02363:2380 85.02813:69352 89.0231:27399 89.9243:1483 90.93176:38061 94.99119:7520 95.0124:13659 95.02369:2061 96.95827:3295 99.00672:2878 101.0226:36861 102.93184:1845 102.9467:8300 103.00234:36900 104.9509:1903 105.91813:1502 108.04317:5751 109.03953:1865 112.93572:5584 113.02246:134619 114.02608:2720 123.05403:1469 124.242:1466 129.01712:8892 129.05466:3159 131.03381:16407 133.01283:11326 139.00337:1748 146.92088:6045 146.96819:2674 147.02734:2863 148.94128:2158 150.05429:6004 157.01317:8824 163.02286:2452 164.89455:1593 166.06148:2546 175.02444:3287 178.02509:2791 193.03543:29605 193.08363:2062
9,10,13-TriHOME	10.078	329.23312	[M-H]-	329.23279	57.0329:2940 70.08175:2224 75.43173:1449 79.73994:1513 87.03336:1344 90.93149:3392 99.0799:12336 113.25509:1329 127.11204:5655 139.11079:20285 157.1214:5090 171.10126:63424 172.10542:1738 183.13844:8296 193.12175:2397 197.06026:10124 199.13336:1583 209.11491:1983 211.13419:82575 212.13577:8361 229.14459:54767 241.05136:9162 282.36542:1744 285.03937:7295 293.21213:2112 311.22034:3312 329.23361:388091 329.67029:2413 330.23871:33249
Arginine	1.251	175.11908	[M+H]+	175.11897	60.05643:12879756 61.05345:88930 61.0598:97958 70.06614:14584301 71.04964:91678 71.06915:458978 72.0816:1003821 73.08502:43769 84.08138:369543 88.07607:108404 97.07683:145751 98.06038:130057 112.08743:1379394 113.07176:159223 113.09123:48317 114.10284:363525 115.08719:330878 116.0712:11827783 117.07394:416952 130.09283:439106 130.09706:4359136 131.10109:125703 133.09827:103474 140.08231:98112 141.06555:116975 157.10823:750553

					158.09325:3880838 159.07616:41523 159.09584:165202 175.11835:17140938 176.12354:839910
LPE(18:2)	12.092	476.27887	[M-H]-	476.27823	59.01231:6550 67.64719:53067 78.95711:145870 122.0004:5560 140.00995:138482 152.99428:40342 196.03569:514697 197.03905:12866 214.04742:121197 245.81311:3396 261.22137:4440 279.23242:5113300 280.23517:438841 465.33408:3525 476.28165:1171231 477.27982:134770
Indole-3-Lactic Acid	8.736	204.06555	[M-H]-	204.06662	68.12231:20593 72.99169:332932 75.00735:53191 96.84335:4450 106.03909:29505 116.0493:457881 117.01208:4202 117.05299:30427 123.04367:18002 124.04675:29462 128.04955:78261 129.05183:4571 130.06448:137951 131.06767:12525 132.04388:62776 133.04811:4305 142.06535:345205 143.06807:31753 144.04364:63026 157.05231:10016 157.99927:14536 158.0596:1699294 158.9586:4135 159.06343:130940 160.07452:4884 161.00221:24817 172.03922:42104 176.97089:4708 186.05472:814093 187.05771:76011 202.04872:12154 203.93866:12058 204.06587:3344928 204.99178:19868 205.06775:304032
Prostaglandin F2b	10.055	353.23276	[M-H]-	353.23279	59.01217:9773 69.03257:2220 69.27583:7921 71.01241:5413 83.04903:10398 96.95827:2891 99.07964:2810 111.07951:15400 113.09525:8074 125.05903:1969 137.09584:2456 165.12749:26079 171.1011:27096 173.13148:7004 181.12177:11225 191.14406:8475 193.12175:51705 193.15964:17696 194.12604:1931 209.1176:27908 211.16917:3034 217.12476:8393 219.17526:3380 221.11766:7985 229.19414:3006 235.13174:13652 237.18689:1790 247.20815:34147 248.21033:2882 255.21278:8690 263.20178:11447 265.21918:6386 273.19022:4084 273.22446:15179 281.21249:7597 291.19815:56894 292.20053:3097 299.20218:12457 309.20944:125737 310.21283:12460 317.20996:21876 335.22382:31718 336.2272:2390 353.23645:499410 354.23959:48527
3,4-Methylenediox	6.899	149.02644	[M-H2O-H]-	149.02386	50.10676:1249 59.32924:1323 63.1928:1290 64.11565:1214 65.9966:1254 67.19495:1266 76.6448:1267 101.02248:2327 120.01969:1486 120.95303:10447 121.02752:10107

ybenzaldehyde					122.02354:1733 123.0178:1583 149.0257:17179 149.04478:2111 150.01768:1302 150.02989:2220 150.05402:16719
15-Deoxy-PGJ2	12.012	317.21075	[M+H]+	317.21109	55.05515:5562 57.07082:1875 60.04541:3679 67.05527:19980 69.07071:13500 71.04986:3783 79.05463:15368 81.07034:26491 83.04979:3602 83.08641:8092 85.06546:14410 91.05502:29757 93.07066:33253 95.05009:2143 95.08614:18425 97.06532:16720 97.10239:2649 99.08051:7686 101.05992:2685 105.07005:32935 107.0496:2513 107.08633:13722 109.06508:7913 109.10181:3467 111.0816:13685 117.07031:38192 118.07386:2340 119.08533:34298 121.06576:6592 121.10125:26178 123.08066:10834 123.11664:2366 125.09658:3695 129.07016:27544 131.08644:39474 132.08858:2886 133.06438:2860 133.10091:29723 135.08139:23977 135.11717:15656 137.05942:2098 137.09619:11071 141.06996:14981 142.07347:1664 143.08578:22771 145.1019:40983 146.10506:3272 147.07976:10061 147.11702:14235 149.09642:29328 150.10127:1808 151.11272:3263 155.08627:13006 157.10062:34918 158.10461:2202 159.08011:3715 159.11629:35579 161.0972:14619 161.13208:6445 163.11082:14923 165.09116:3267 167.08461:1764 167.10715:2593 169.10176:22043 171.11768:51501 172.12201:3594 173.09576:12885 173.13298:20527 175.11191:18541 175.14909:3347 177.0909:2035 177.12666:3400 179.10611:3756 181.10042:9451 183.11652:43309 184.11946:3181 185.09718:3053 185.13295:30477 186.1357:2304 187.11229:26532 187.14847:14656 188.11668:2941 189.12683:15952 191.10791:3583 193.12331:7735 195.11635:8922 197.13245:32699 198.13651:2913 199.1106:19732 199.14815:7578 201.12717:62334 202.13187:8544 203.10567:3083 203.14417:3650 205.12222:17061 206.12584:2070 207.11736:3250 211.15005:3399 213.12805:9285 215.14302:31213 216.14616:2568 217.12292:7085 219.13943:60157 220.14285:9875 221.13127:8259 231.13918:2548 233.15518:40912 234.15796:3077 235.14999:2997 237.1635:6447 239.14333:3277 239.17841:8312 243.13861:1848 253.16087:3274 253.19525:21647 254.19934:3116 257.18903:2741 263.17889:32653 264.18207:7154 271.18015:1949

					271.20517:19700 272.20801:2048 273.18604:2451 281.19022:83984 282.19373:15403 289.17807:2408 299.20193:214749 300.20529:40238 317.21021:165060 318.21445:28536 318.29877:19950
Lidocaine	7.54	235.18033	[M+H]+	235.18048	57.07082:3098 58.06591:22116 71.01125:2708 81.07029:2716 86.09715:2463453 87.04517:2785 87.09749:4742 87.10057:97508 89.06052:3980 103.0397:8406 160.105:1905 179.10609:2926 193.93016:2338 207.05518:2278 211.94377:2902 212.96584:4190 217.19576:1952 235.18082:582424 236.09329:3371 236.1834:68027
Tobramycin	10.087	485.2901	[M+2H]2+	485.29291	67.7115:61921 76.03996:76763 81.07028:8048 85.06546:18584 95.08612:6105 105.07005:6999 107.08531:6759 119.08541:6128 121.10114:7441 131.08646:4824 133.10077:8869 135.11716:14380 145.10193:9180 147.1169:8168 157.10255:7270 158.08194:61820 159.11613:30948 161.13206:14061 171.11769:14396 173.1331:6934 175.14908:4438 183.11655:5310 185.13301:8985 187.14896:5945 195.11633:7329 197.13252:7571 198.11269:6985 199.14803:33950 200.15144:5030 201.16504:8314 209.13364:86913 210.13811:8338 211.14713:21301 213.16403:58279 214.1676:6290 215.17894:6886 223.14714:22897 225.16508:24659 227.1427:88025 227.17747:42317 228.14571:7192 229.15897:16486 237.16348:14632 238.16774:4703 239.17839:5137 241.15869:15931 241.19417:28208 243.17456:7020 255.17461:8115 281.22607:4597 293.22632:8805 295.24161:6562 296.2449:5125 309.25583:7145 318.20911:7030 319.24005:183057 320.24347:37815 337.25195:378107 338.25809:85621 339.25812:6092 370.23392:4929 394.27612:13065 412.28207:817011 413.29202:202998 414.28986:41572 430.29434:222625 431.29553:64715 432.29947:16945 442.26324:6565 448.30453:43703 449.30606:8299 450.22882:6314 458.26263:6062 466.31296:9065 468.24811:23933 469.24911:13594 476.27798:8080 476.78113:5303 485.22501:4752 485.29202:27934 485.79318:14107 486.24841:206627 487.25815:58679
PFSA-ether	0.694	430.92792	[M-H2O-H]-	430.92642	67.76324:31104 70.99763:4094 96.83883:10016 112.98481:19558 114.98679:19235 130.98239:166640 131.98326:13132 158.97755:1432541 159.97829:123239

					226.96593:590089 227.96808:63176 294.95343:70362 295.95483:4405
GlcNAc	0.948	256.05844	[M+Cl]-	256.0593	59.01201:26086 59.96228:1911 62.42421:2419 66.81934:2106 69.27418:10451 71.0124:3945 78.91772:2865 78.95782:16187 80.91505:3035 89.02287:13468 92.92597:4086 94.92359:3381 96.84246:2262 96.96822:3219 100.03912:20344 101.02253:25757 119.03315:40144 119.08118:2228 136.03175:9376 136.91634:8879 138.91945:2213 140.91606:3877 142.0493:2372 180.0334:3044 219.84457:3062 220.08282:5387 239.11443:2661 256.05786:78309 256.87589:3840 257.05951:4726
7'-Carboxy-gamma-tocotrienol	11.844	331.19092	[M-H]-	331.19095	51.46453:1377 59.01221:10210 59.83126:1275 67.6006:6349 67.60482:19676 71.85065:1282 83.04874:2379 96.83885:6781 96.84428:1957 103.53468:1456 123.04366:2428 135.07976:1350 189.12744:1492 189.16446:4802 215.14178:1352 233.15433:2422 243.21008:21094 269.19214:20385 270.1958:1938 280.22217:1285 287.20126:69434 288.20575:6639 313.18091:9990 331.19147:95147 332.19577:10838
Altretamine	10.78	211.16928	[M+H]+	211.16658	55.01875:2228 55.05521:58431 57.03445:6615 57.07092:10151 58.06583:5713 67.05527:68083 69.03423:1863 69.07077:79551 69.31908:7655 70.06626:13876 71.04993:24643 71.08622:3261 72.08165:7103 79.05531:16546 81.07036:103966 82.07365:5448 83.04979:9157 83.08649:96565 84.08146:5278 84.0895:5432 85.0655:23506 86.06078:5159 86.09715:5702 87.0444:1991 91.0552:20713 93.07077:44671 94.07333:2300 95.04921:2225 95.08624:196062 96.08945:8088 97.06525:45072 97.10161:60290 98.06048:6995 98.10471:2920 99.04404:2624 99.08144:9152 101.06088:3223 105.07008:23073 107.08641:26434 109.1019:112690 110.10519:5906 111.08155:25388 111.11691:8505 113.06054:2406 113.09704:1856 115.07552:7159 119.08556:58381 119.95786:3214 120.08881:5014 121.1015:17129 123.08045:16178 123.11687:13425 125.09658:3400 127.07574:2950 129.09113:2146 133.10094:65242 134.10402:5490 135.11711:18030 137.09628:20958 137.96786:6652 138.10014:2066 141.05423:6497 141.09077:2827 147.11708:31099 148.07608:1662

					149.13284:32652 151.11095:7982 151.14801:32053 155.10661:2851 165.10323:7042 165.12737:3167 166.08543:7206 167.99368:2852 175.1492:137809 176.15155:17774 183.11421:2477 184.09814:1928 193.09785:2322 193.12331:2166 193.15909:56287 194.00958:8219 194.08138:2869 194.16089:5171 195.07759:1882 211.10931:54254 211.1326:29632 211.16898:40626 212.01974:12506 212.09018:6868 212.14293:14563 212.16637:3052
4-ethyl-7,9-dimethyl-3-phenyl-5,7,9-trihydro-1H,4H-1,2,4-triazino[4,3-h]purine -6,8-dione	10.211	337.14218	[M-H]-	337.14182	69.57209:1292 71.0266:1862 78.95765:181937 96.96825:456334 97.68127:1354 102.94675:27851 108.97908:1759 133.51808:1348 145.06219:1655 145.92105:1262 146.93756:49120 147.93958:1387 167.93016:1372 174.33255:1541 190.92805:14785 236.10966:1398 265.90466:1400 337.14145:88165 337.20529:51038 338.14752:3236 338.20651:1979
LPC(16:0)	12.385	496.33917	[M+Na]+	496.3403	57.07082:4834143 58.06667:4160182 60.0816:25868768 71.07372:3375955 71.08622:3400755 85.10194:1761011 86.09715:83546472 95.08613:1970930 104.1074:429726432 105.11093:5876262 124.99983:29601584 163.01457:3424834 184.07445:524532480 185.07628:6715236 225.90071:1913422 258.11118:10157058 313.27136:17125828 419.25269:3629570 478.32599:38947216 479.33881:4011202 496.3407:168345408 497.33859:19366290
Phosphocholine	12.267	184.07352	[M+H]+	184.07327	58.06625:4132 60.08159:214872 71.04985:5220 71.07372:17967 74.9382:3536 75.26474:3041 78.37572:2805 86.0971:968721 87.10039:28839 91.62558:3041 96.84246:7801 98.98516:20315 101.73389:2813 104.10714:22307 106.03684:6574 113.96436:3394 114.95605:26405 115.96434:23038 116.97191:35065 117.98022:83418

					120.96474:4944 124.07577:3742 124.99973:574294 126.00383:4697 132.9666:25486 133.97449:6360 138.09244:4185 139.98784:17008 140.99545:16398 141.98357:3892 143.01122:7118 147.18462:3030 149.07059:4707 155.98236:3535 161.9689:21057 166.02657:5765 166.08745:4005 167.08051:6499 184.0743:2388698 184.98497:5886 185.07629:80879 185.13062:4282 185.16406:10775
cis-Aconitate	3.24	173.00764	[M-H]-	173.00912	59.01192:6339 61.9868:6828 69.86469:5179 85.02796:428042 86.03082:12397 89.02285:7740 111.00733:45450 111.07956:43860 112.01141:1679 115.91896:6886 118.96381:13147 127.11204:1508 128.87665:220262 129.01826:101239 129.09077:2989 130.02084:2134 130.08562:9910 131.08189:5857 146.99518:1551 154.94569:5407 155.07016:1960 156.06577:9125 172.82936:6249 172.866:4588 172.93932:7804 173.00766:13781 173.08125:43982 173.11848:5835 174.07536:9649 174.11:1792
PE(21:0/22:6)	10.935	834.60596	[M+NH4]+	834.60071	60.08158:14540 81.07101:46710 85.06542:38706 86.09702:89761 95.08624:52509 101.06084:26878 105.06992:48225 107.08646:65160 109.10176:28771 119.08536:24566 121.10108:35287 123.11788:29115 124.99953:49934 131.08632:47292 133.10205:48606 135.1171:35450 145.10185:82194 147.11678:90370 149.13271:34327 153.09096:11922 157.10062:27659 159.1163:164098 161.13182:84764 163.1488:11439 167.10707:36323 171.11761:14454 173.13298:53499 175.149:43882 181.12344:27250 184.07431:940072 185.07561:35250 185.13293:43934 187.14888:31717 189.16339:15961 193.12315:56766 195.13695:13082 199.14822:113492 201.16498:59693 205.12181:12027 207.13785:48680 209.1333:94941 211.15009:47318 213.16336:258639 214.16753:14817 215.17892:63877 221.15295:52105 223.15022:23611 225.16499:42242 227.14249:111232 227.17792:110335 229.15887:33329 231.17574:26919 235.17041:12954 237.16339:26928 239.17828:12091 241.19382:120937 243.17445:12780 245.15369:352830 246.15793:39960 247.16879:111807 254.20308:30794 255.20943:59474 259.16733:90794 261.18604:108685 273.18454:71321 287.20129:14284 295.24149:26443 299.20215:31152 301.21487:14048 309.25571:33976 313.21814:78630 319.23953:254764 320.24289:36579

					337.25159:465523 338.25949:79343 355.26498:8972113 356.26437:1423676 373.27249:5560714 374.27985:879941 391.28571:366911 392.28574:75821 409.29706:11275 426.31805:1250160 427.32236:180544 781.57007:21356 799.57697:55007 800.55988:12513 817.57983:58398 818.58185:15344 834.59937:51743
Racivir	7.711	246.03809	[M-H]-	246.0354	55.52898:1427 63.21805:1576 68.48933:10959 72.33952:1377 73.74441:1565 81.48482:1327 83.02291:2282 96.84067:2479 102.03347:2137 103.04187:1329 104.92642:1525 116.92693:3094 118.94205:1788 134.05978:202586 135.0639:16424 162.83745:1654 162.93208:2247 163.69218:1553 167.03384:9765 167.07071:2215 178.04988:955193 179.05257:67273 192.0507:9811 200.92038:1621 201.99159:2142 203.04944:2949 218.02864:25195 219.03069:7267 220.04573:14417 221.04596:1436 246.02551:15452 247.04248:2705
Palmitoleic acid	12.293	255.23209	[M+H]+	255.23187	55.05522:63317 57.03445:4505 57.07091:123328 67.05521:27723 69.0708:143314 70.06577:50247 71.04986:4482 71.08631:83856 73.06554:3946 79.05463:5419 81.07034:70401 83.04979:6804 83.08652:149140 84.08163:55198 85.06547:6218 85.1019:53624 89.06053:5069 93.07087:30752 95.0862:62819 96.84789:24558 97.06523:37549 97.10162:92740 98.09641:5900 98.10471:3769 99.08051:4134 101.05992:5004 107.08549:44791 109.10187:40647 111.08042:26280 111.11707:36208 115.07553:7188 121.1012:55752 123.11671:24499 125.09668:28577 125.13242:4237 133.08621:4229 135.11714:60884 136.11925:4687 137.13251:16025 139.11249:26455 149.1326:56005 151.14972:3704 153.12701:24769 157.12125:4218 163.14871:32164 167.14201:23241 177.16469:11647 181.15822:13353 195.17583:4510 219.21075:76871 220.21413:14126 225.50838:5374 237.22237:40537 238.25145:13358 239.14685:4794 255.11665:5988 256.2644:871726
N-Carbamoylsarc	1.582	131.0448	[M-H]-	131.04567	59.01194:1492 61.34794:1364 69.0331:1528 70.11794:2109 79.83392:1274 81.33294:1281 85.02798:1667 85.06458:9198 87.0436:25242 88.0386:64494 89.04199:1967 99.89466:1230 110.06613:1356 113.02226:1470 131.03371:10767

osine					131.06911:8132 132.05522:3143
1-Tetradecanol hydrogen sulfate	13.399	293.17938	[M-H]-	293.17923	70.39382:8412 78.9576:5815 79.9558:8403 96.9582:1610281 106.03913:26443 108.98872:5608 171.06509:6860 171.23924:6547 224.09425:6251 293.17847:7174505 293.64587:21946 294.18063:671877
Cytidine	6.192	302.09921	[M-H]-	302.099	53.28209:1304 70.11461:4917 75.00727:12175 92.6344:1554 124.05043:363958 125.05376:13647 130.50735:1377 131.03354:2059 144.95784:5536 158.97765:2242 187.08675:1794 188.94749:2947 191.84274:1452 234.75287:1417 256.09283:8200 295.04913:1381
N-{4-[(1-methylimidazol-2-yl)carbonyl]phenyl}(3,4,5-trimethoxyphenyl)carboxamide	8.366	396.15317	[2M+H]+	396.15536	69.64206:2562 69.64536:5498 70.06564:3956 89.06053:3411 133.67792:1792 138.05257:146743 139.0563:6684 142.06384:1948 152.03125:9686 179.19026:1607 249.58524:1671 253.09558:17842 261.04599:1704 267.11395:2794 281.09189:51317 282.09137:3881 299.09937:267157 300.10239:32795 368.15506:1919 396.15402:1106159 396.6405:2634 397.15793:176960
5-O-Methyllicoricidin	10.086	477.30148	[M+2H]2+	477.29758	69.11263:21741 76.03996:61555 81.07029:7887 85.06546:14018 95.08613:5523 105.07006:4950 107.08532:14734 109.10181:15020 121.10114:12907 131.08647:6960 133.10077:7021 135.11716:7810 145.10028:7348 147.11691:14962 158.08203:62124 159.1163:29328 161.13208:14326 169.10188:5724 171.11769:4638 173.13312:4889 175.14688:4643 183.11656:6100 185.13301:7833 189.12646:5879 189.16347:4723 197.13254:7595 198.11005:5346 199.14903:34729 200.15144:6309 201.16505:4940

					209.13329:86262 210.13812:5829 211.14656:27853 213.16388:47654 223.14716:13195 224.12726:4591 225.1651:21969 227.14261:78939 227.17955:27007 228.14572:13842 229.15898:15024 237.1635:15153 239.1819:4384 241.15871:6570 241.19423:8763 243.17458:8087 249.16318:4816 255.17462:5311 263.17902:6349 281.19031:4653 293.22632:7260 295.20795:10574 295.24164:16634 304.19193:7802 309.25586:7851 318.20911:4485 319.23993:133964 320.24295:29142 335.23456:5822 337.25177:330795 338.25909:60473 355.25891:4804 394.27615:5947 408.2782:6408 412.2822:676082 413.28433:165611 414.29007:20175 424.26358:15687 426.28476:16712 430.29446:200923 431.29517:49403 434.25671:44936 435.25555:21005 444.29913:6917 448.30753:32338 449.30609:9003 452.2641:108078 453.26892:39266 454.26962:8301 466.31299:18213 470.27283:213933 471.27979:75024 472.27136:31064 478.26566:6708 478.3252:5776
5-Bromo-6-methyluracil	1.104	204.95718	[M+H]+	204.961	62.98244:8216 68.05199:49944 73.02943:4761 90.97693:1815965 91.98014:14502 96.64124:4181 205.06902:18767 205.09409:63285 206.08096:7511
Vitispirane	10.779	193.15921	[M+H]+	193.15924	55.05515:7271 57.03449:11213 57.07084:10469 58.06584:17639 67.05525:51477 69.05491:4335 69.05872:2583 69.07075:12793 71.04995:22254 79.05522:16062 81.0343:1714 81.07034:73385 82.07436:2440 83.04979:8143 83.08635:18074 85.06542:15080 91.05502:23975 93.07079:46064 95.05009:3209 95.08618:119322 96.08945:3504 97.06528:20519 97.10149:2995 98.06049:2902 99.08157:11002 105.07016:40906 107.08636:41937 107.96087:2115 108.04496:1869 109.06508:7336 109.10185:57951 110.10519:2483 111.08151:20133 113.09704:2772 117.07034:1577 119.08548:76966 120.08132:6643 121.10138:28677 123.08044:15750 123.11664:7372 125.09658:5693 126.05483:1812 128.96463:1735 130.01695:1856 131.08647:4923 133.1008:75853 134.1055:6037 135.04419:47174 135.11723:27863 136.12074:1986 137.05942:1613 137.09608:13688 146.97482:16482 147.11691:40350 148.07608:9794

					148.11197:3453 149.06026:2848 149.13271:59148 150.13791:2761 151.11272:2570 151.14796:2390 151.97971:1885 161.0584:1880 162.05537:20444 165.10323:2011 165.12738:1893 165.1636:1805 166.08543:2060 169.98953:22004 175.08755:2656 175.14903:228613 176.07166:5167 176.15126:22375 193.00627:2908 193.049:25707 193.09697:18665 193.15894:262516 194.07947:18641 194.11708:111167 194.16441:29724
PFSM-amine	3.238	333.05103	[M-H]-	333.05133	70.35299:2332 83.31137:1421 87.9236:10333 103.91858:44371 103.95462:2255 104.92645:14486 105.93364:6821 106.86631:1598 106.94179:3469 123.9446:16110 125.94849:1431 127.9549:2116 130.9426:48424 144.95786:5754 145.96622:31959 155.95123:2590 156.01036:2926 157.96539:42101 158.97417:21100 163.97661:8838 171.94499:1683 174.95421:1589 186.02104:5507 189.95656:32439 199.99986:43048 201.00415:2310 201.95625:14063 202.96512:11138 218.72972:1455 220.90578:6248 236.89961:2822 243.05522:116293 244.05736:7206 262.91565:5582 264.89587:9068 272.69839:1412 287.04459:148982 288.05246:12993 306.90762:10846
LPE(16:0)	12.357	452.27716	[M-H]-	452.27771	69.27966:5527 78.95715:53624 140.01004:50589 152.99448:15374 196.03587:184315 197.03925:3352 214.04739:35322 255.23207:1845119 256.23666:143349 452.27798:486543 453.28168:53537
LPE(18:3)	11.569	476.27698	[M+H]+	476.27719	55.05515:11857 57.03445:7530 57.07083:2681 62.06075:132165 67.05531:47630 67.64287:9421 67.64657:25180 69.0707:14987 79.05472:22423 81.07035:44736 83.08635:14682 85.06547:2245 86.09715:3034 89.06053:9183 91.05512:12363 93.07069:37179 95.08619:56676 97.10149:3259 98.98518:13066 102.12821:3886 105.07018:16827 107.08637:21678 109.1018:24867 119.0854:19110 121.10111:29490 123.11665:11031 131.08505:8600 133.06441:45543 133.10103:17868 135.11723:19091 137.13251:2763 142.02692:9109 145.10196:9357 147.11734:15470 149.13278:9902 155.01118:13462 159.11629:3457 161.1321:12301 163.1489:4094 173.02135:13788 173.13313:3901 175.14911:3121 184.0721:3137 198.0519:8618 216.06444:18716

					243.21056:8916 261.22156:30590 279.12234:2882 297.13049:12932 304.2626:47570 305.26657:2670 317.24768:9931 335.25781:410581 336.26138:54906 415.22742:4026 429.3819:2484 458.26135:19591 459.27853:3073 476.27805:14180
Leucinic acid	8.135	131.07002	[M-H]-	131.07132	55.15179:3389 59.86684:2604 65.0126:4559 67.02808:4606 67.14743:2499 69.03311:10056 69.37163:13814 77.01332:3033 79.63:2770 85.02798:4246 85.06454:210716 86.06734:10818 87.04348:26119 89.01252:5606 91.02863:5603 99.09647:2721 102.98733:9105 111.72752:3061 112.8072:2932 113.0599:3231 131.03528:42779 131.06914:165628 132.05524:16351 132.07394:6570
N-Acetylalanine	2.99	154.04733	[M+Na]+	154.04742	51.94093:4949 55.9354:387406 67.05524:17905 68.94347:7811 69.70879:7529 70.06564:23502 71.92982:5640 72.93779:78596 73.94594:52228 80.05057:6137 81.07029:22244 82.0659:7936 84.04491:8092 84.08146:20684 85.02901:4423 89.94057:53204 90.9481:135336 91.95587:6690 94.06554:6449 95.08613:7273 96.08141:8669 98.06048:8494 100.04057:5971 105.93538:33065 107.95126:183692 108.04496:22767 108.05668:4343 108.08125:44770 108.95927:28255 109.02836:9392 109.06508:8540 109.10181:17510 111.04473:16476 112.03909:15818 112.07622:8434 113.96437:27095 117.93497:38162 118.94257:16083 123.94611:23428 125.96093:277659 126.05499:44684 126.09075:39823 127.0867:28848 128.95103:28873 130.96698:15751 131.97491:8703 135.94615:776795 136.03944:7168 136.07555:68618 136.94687:18997 137.06094:7770 137.09595:6655 146.96133:16510 148.9776:28756 149.96184:14877 153.95517:661820 154.048:133523 154.08571:167296 154.1232:40346 154.9071:19364 154.95831:16781 155.04434:8657 155.08052:111296 155.1176:15712 155.15425:25637
1H,1H,2H,2H-Perfluorohexanol	1.636	263.00821	[M-H]-	263.01239	69.07664:8104 89.02298:327551 90.02546:7635 130.96565:13276 142.96413:3093 144.94458:6934 144.98112:46065 148.97574:7592 152.89377:47590 160.95406:3364 162.99133:39644 170.95897:38219 172.93965:26197 172.976:188896 173.97935:6606 188.9698:24312 190.98634:264543 191.98897:3232 200.97115:19000 203.01372:2875 208.88245:23266 212.97049:2918 214.98607:99647 215.98825:3071 216.96701:7472

					217.00337:54835 218.98174:54552 222.86177:2721 232.99582:249121 234.00073:16597 234.91667:6176 234.97473:8787 235.01241:299252 236.01665:14071 262.89069:44191 263.00861:641216 264.01059:34788
16-Hydroxy-10-oxohexadecanoic acid	12.039	285.20685	[M-H]-	285.20657	51.49921:1482 59.01194:1515 68.57194:9479 81.45203:1565 82.03945:1428 96.84155:3000 100.16413:1468 115.91898:2066 116.92692:2241 156.11943:1760 164.80418:1810 166.56798:1567 183.11026:1699 194.97276:3155 196.97104:1870 202.83005:1595 211.15459:1510 223.20615:554069 224.20905:57082 241.21881:18129 261.84338:1719 267.1965:550240 268.19772:53105 285.20828:1380456 285.90402:2529 286.21158:146629
3-Amino-2,2-dimethylpropanoic acid	1.452	118.08673	[M+CH3OH+H]+	118.08626	55.05517:407880 58.06589:1782372 59.0738:2626367 61.52355:43693 70.84187:66060 72.0817:4044108 73.0851:271414 88.91087:46339 91.05434:43765 118.08727:12194820 119.09142:417391
PC(16:0/18:2)	12.464	758.56903	[M+H]+	758.5694	58.06372:528060 58.06666:2263231 59.07416:475926 60.08165:11180120 71.07359:2344355 86.09716:46512976 87.10064:1331174 96.84245:806223 98.98515:1295717 104.10735:4301052 124.99975:20191356 184.07433:464700704 185.07829:11979399 225.55008:508291 494.33264:691614 758.57104:11905194 759.58289:3611544
2-oxo-Butanal	2.957	87.04483	[M+H]+	87.04405	53.00287:25342 53.03949:12627 55.05524:77706 56.0506:10452 57.03405:22523 57.07042:7561 58.06626:6945 59.05007:13285 60.04542:35805 65.27004:6215 68.98314:36580 68.99781:11852 69.03432:67397 69.07068:91266 70.03785:9573 70.0657:139951 70.074:13747 70.56277:12222 71.01353:24800 71.04987:33107 71.07373:8554 75.04478:10055 86.93977:10445 86.99354:89668 87.03364:7702 87.04442:272389 87.06433:118293 87.0814:12787 87.0975:9909 87.10059:218444 88.02228:13250 88.0403:29146 88.07634:1033705

N-Methyllysine	1.243	161.12836	[M+H]+	161.12848	56.05059:7880 60.08161:20600 67.05473:6675 70.06564:9220 71.02657:5714 72.08173:72751 84.08147:3101003 85.06546:28068 85.07961:7302 85.08482:107895 98.09736:210205 99.10015:6478 100.11272:6165 102.0558:19346 102.09102:12948 115.12237:7985 116.07124:7176 116.10695:115061 125.10719:8476 126.09107:20371 130.08595:1816280 131.08943:104448 143.11833:26256 144.10257:125356 161.12828:654042 162.05161:18237 162.11227:21727 162.13185:26237
Citrulline	1.324	176.103	[M+H]+	176.10298	60.05644:34085 69.86139:111044 70.06615:7055422 71.06926:229333 86.06078:130532 87.09216:118125 97.07697:160248 113.07185:9612392 114.05553:1719591 114.07493:349498 115.05911:44129 115.08714:2610379 116.07116:1735067 116.0902:55818 117.07392:133591 130.09723:168885 131.08218:43069 133.09756:213643 141.06534:341952 142.05045:226217 159.0762:16383132 160.08037:726607 176.02954:134517 176.10266:449365 176.1226:106625 177.06183:122177
N-[4-[(9S,13R)-16-hydroxy-7,9,13-trimethyl-5-oxapentacyclo[10.8.0.02,9.04,8.013,18]icosa-6,18-dien-6-yl]-2-methylbutyl]ac	12.387	478.32782	[M+Na]+	478.32913	53.1506:12062 55.05513:31892 56.05049:47416 57.03439:11122 57.0708:234076 60.08155:5276 65.03953:28414 67.05511:37962 69.07061:48050 70.06612:10300 71.07366:260012 71.08617:193719 81.07088:83400 83.04974:20531 83.08631:87090 85.10188:139006 86.09705:3285721 87.09517:4987 87.1005:94842 95.08605:152358 97.10147:48289 98.98506:31622 99.11783:8801 104.10726:5250 109.1017:81418 111.1179:9359 123.11779:41682 124.99966:707529 125.13229:10391 126.00371:4580 136.99988:37154 137.13225:15163 143.01108:9066 151.0157:13604 151.1478:4934 163.01631:1038763 164.0199:26607 166.0264:10573 181.02623:266093 182.03067:4712 184.07419:311581 185.0779:8709 239.23796:57621 240.09996:10149 283.26303:89109 284.26489:11654 295.26227:16813 337.27512:8483 419.25235:689638 420.25555:104249 478.25537:30238 478.3248:5474187 479.32849:867468

etamide					
Ornithine	0.888	131.0811	[M-H]-	131.08263	69.26107:39682 83.05952:11941 83.89282:7786 85.07561:13939 87.0434:17824 88.03848:59507 92.10062:7297 113.07017:13334 114.01801:7757 114.05381:8771 115.00237:12839 124.48667:8237 131.03215:13493 131.08055:1977530 131.56009:7877 132.02934:18374 132.08446:80288
Tropic acid	6.365	149.06	[M+H-H2O]+	149.06	66.25119:4610 71.01978:7390 77.03943:5627 79.05527:142820 80.05805:5637 84.36811:4733 91.05515:29917 93.0707:261168 94.07421:16481 95.05009:20419 100.8166:5204 103.0546:585483 104.04998:44184 104.05804:36088 105.07008:5233 107.04959:253713 108.04498:7237 108.05243:10542 117.25317:4359 121.02914:7008 121.06575:188103 122.06876:17127 131.04948:69078 135.50681:5065 149.0237:35911 149.06035:128320 149.10689:6712 150.05421:11597 150.06467:7609 150.07687:7823 150.09084:17578
1,2-Dimethylimidazole	13.321	97.07658	[M+H]+	97.07603	53.03911:7767 54.03476:71149 55.05515:7902 56.05025:276222 57.04553:91629 57.05815:57328 68.05034:34806 69.04567:161659 70.0295:8768 70.06624:239016 70.75091:9573 71.06123:19524 80.01391:21221 80.05055:161662 81.04536:193615 82.05321:101069 97.02984:9638 97.03983:306961 97.07698:1083967 98.04852:29914 98.06093:100229 98.07153:408136 98.07983:37882 98.09713:31289
N-Lauroylglycine	12.096	256.19156	[M-H]-	256.19128	57.34548:1227 61.85373:1370 66.75309:1202 70.57233:1718 72.04399:1391 74.02332:300564 75.02641:2706 84.04388:1555 93.74184:1241 103.84313:1455 116.92694:3372 155.94016:1314 212.20026:16525 213.20361:1436 239.1671:1938 256.19034:212969 257.17029:26741
Shikimic acid	12.429	192.08411	[M+H]+	192.08662	67.0766:1631 68.14742:17391 74.88963:1527 87.0444:2057 95.08613:2558 96.84518:3443 109.10181:3440 121.06576:1603 121.10114:1699 133.06438:2363 133.10223:2050 136.02138:7002 137.09595:2387 146.09668:7391 146.97318:2084

					149.02899:26208 150.03705:27332 155.83835:1753 162.03792:9802 163.04597:66502 164.0479:3586 169.98964:3048 177.05962:3195 192.08385:1788308 192.17485:9635 193.04951:6756 193.08752:164975 193.12331:7083 193.14368:8908
PS(16:0/18:2)	10.465	758.49811	[M-H]-	758.49774	78.95716:312709 96.96841:45652 134.98291:9186 152.9942:546129 171.00385:15855 253.21727:1199120 254.22148:82439 281.2478:466440 282.25229:29334 389.21075:368104 390.21036:24018 407.21786:146001 408.22531:8848 417.24194:84666 418.24725:9458 435.24881:17274 671.45923:515500 672.46765:76238 758.49011:140515 759.49854:27680
1-Hydroxy-2-butanone	6.272	87.04351	[M-H]-	87.0446	59.01238:3121 59.98388:2233 68.03017:23255 87.00723:130717 87.0434:120855 88.00249:36381 88.03851:10555
Acetaminophen glucuronide	6.324	326.12369	[M-H]-	326.12726	71.01181:5984 72.00741:22776 73.02773:23808 84.00732:4139 101.02251:220303 112.03838:8356 144.08127:25030 147.04419:158226 148.04707:4639 149.05853:20481 164.07021:1990734 165.03113:4332 165.07346:88709 178.07233:4296 193.0574:31505 194.06703:58079 206.08386:49109 208.08339:45285 211.10799:14188 220.97421:5245 221.05524:6317 236.07687:481224 237.07777:21941 248.09111:28854 260.09201:14111 278.10312:14913 280.03964:30989 326.11142:305576 327.11438:27551
4-Vinylcyclohexene	8.814	109.10162	[M+H-H2O]+	109.10117	53.00286:9014 55.05521:125085 65.0396:33652 66.49171:11008 67.05524:1413672 68.05842:53114 69.07064:66947 70.36418:13464 72.93777:12847 77.99937:38275 79.05463:46447 81.07029:450570 82.06589:76169 86.00702:74091 86.50604:136159 87.00438:811799 89.507:12418 92.05045:9466 94.07854:13019 95.51141:31197 96.00999:149176 98.01446:29508 98.51231:169370 105.00371:8933 107.08622:68466 109.06509:10556 109.07697:64847 109.10181:889133 110.02067:55265 110.06026:243873 110.105:48152 111.96827:13147
N-Fructosyl	6.373	328.13885	[M+H]+	328.13907	69.03438:65203 70.0662:10284 70.19208:12397 85.02913:54739 87.03208:43364 89.06052:10754 91.05501:55909 97.02897:110782 99.04497:26840 105.07006:8788

phenylalanine					112.04021:23835 120.08137:449010 121.08471:31379 127.03909:126945 129.07025:8077 130.06476:14277 131.04947:36385 132.08138:278923 133.08475:30080 134.09665:9699 143.0582:67423 144.08139:47017 145.0489:22848 149.06024:9451 156.08041:11810 161.06792:97944 162.0905:51377 166.08553:379800 167.09135:42437 178.08751:194754 179.09019:8414 186.09235:14910 196.08376:63538 198.09154:42219 202.08554:29694 210.09192:10935 221.56854:7726 228.10316:15665 244.09853:81179 246.11349:133996 264.125:570741 265.12512:62444 282.13174:63515 292.11984:697726 293.12167:89317 310.12939:1355044 311.13187:186577 328.1423:13177
Homocitrulline	1.402	190.11852	[M+H]+	190.11858	56.05059:42206 62.06116:35237 82.0659:14553 84.04491:20756 84.08145:1430788 85.08482:61120 98.06033:89609 100.03963:56477 100.07665:145940 102.05582:40415 110.06028:51825 126.09109:15093 127.08663:2609008 128.07065:292548 128.09006:114117 129.10365:20146 130.0507:14308 130.08559:131664 144.06485:39578 144.11378:119382 145.09694:73484 147.11336:110566 148.00433:37844 172.09633:12641 173.09207:2419879 174.0968:137726 190.07233:13563 190.12004:228025 191.04036:104447 191.0773:94298
2,2-Dimethylsuccinic acid	6.745	145.04916	[M-H]-	145.05008	64.47848:1376 69.85087:2744 69.85419:1784 75.63348:1161 81.03275:10207 83.04881:99409 84.05192:5806 95.87184:1208 101.05917:103198 102.03348:1898 102.06281:4777 106.64348:1510 115.91901:1397 118.02831:1820 118.03926:3180 119.0233:1661 127.03872:5181 137.27415:1271 145.04893:57618 146.02307:7160 146.03313:3196 146.05153:1716
2-[4-hydroxy-3-(sulfooxy)phenyl]acetic acid	6.359	246.99152	[M-H]-	246.99124	59.01237:2065 69.59631:3330 79.95554:20561 80.96342:524920 81.03324:2451 81.96269:2734 93.03312:3133 95.01225:1945 99.04316:1884 99.92419:12641 103.91843:2735 108.01973:77770 109.02833:12958 116.92693:7655 117.05415:3450 118.94204:2538 121.02738:237791 122.03102:14932 123.04362:95230 124.04652:6502 137.02278:6654 146.95967:31524 147.04424:2200 162.93207:2748 164.07013:25759 167.03415:11473 180.06549:8069 187.1086:1865 188.97214:1947 201.00682:3289

					203.00008:223902 204.00511:17243 246.99097:388064 247.99564:30618
m-Hydroxyhippuric acid	6.861	194.04509	[M-H]-	194.04591	56.17305:1533 66.03321:37887 69.26162:5645 80.04883:1648 82.02819:1504 91.02864:1768 92.92769:9243 93.03316:77331 93.05186:1975 94.03607:2666 101.70776:1405 108.04424:6944 117.16834:1345 122.05933:1670 123.04368:2156 128.07069:12547 137.0228:1826 148.03909:10276 150.05434:310913 151.0582:17921 167.0441:1856 192.24916:1402 194.04408:135928 194.08246:6197 195.04878:10308 195.11246:215712
Diflufenzopyr	6.261	333.07974	[M-H2O-H]-	333.08051	57.03289:7186 59.01236:9938 68.28096:12483 71.0126:15262 72.99161:2119 75.00687:108945 85.02808:79156 87.00719:23850 89.02285:3422 95.01223:6176 96.84063:5523 99.00762:8719 112.01498:20692 113.02238:74820 117.01809:2289 129.01729:13893 130.99654:2197 141.05417:2417 154.99509:3307 155.0555:3044 157.01306:15150 175.02438:5847 189.07561:5892 205.07018:14497 229.06906:2487 247.08292:8614 265.09213:984657 266.09781:52723 287.06247:77888 288.06653:8677 333.08133:38823
5-Hydroxyhexadecanoic acid	12.308	271.2272	[M-H]-	271.22733	59.01194:1938 70.36754:2665 72.83279:1310 82.03946:1631 99.92403:1667 120.65445:1314 127.40618:1269 175.22446:1439 225.22153:20993 226.22528:3269 226.26079:1629 231.56538:1445 253.21667:10405 269.2128:2280 271.22659:1006268 272.229:123867
Olivetolic acid	9.842	223.09665	[M-H]-	223.09763	56.02479:1368 59.01212:20909 69.88796:1275 69.89129:3272 96.95826:2035 99.92402:2032 107.08561:1744 109.03951:1241 110.74388:1212 123.89986:8960 125.89896:10223 135.11586:14834 142.77788:1323 151.07582:5957 161.09554:2863 176.9776:1626 177.08931:1665 179.10684:65520 180.10902:7470 193.86206:1270 196.06438:1451 205.08713:11398 222.97858:2772 223.0607:1705 223.09546:7937 223.13339:1712 223.79874:6910 223.84003:2763

Terazosin	8.744	386.18109	[M-H]-	386.18332	55.01067:1543 69.82983:2437 96.83886:1632 102.05418:27865 127.53312:1482 128.03323:155738 146.04477:43318 158.97766:1661 172.23189:1614 174.95639:2520 177.12741:19650 195.138:43536 196.13995:1629 211.13425:2752 239.12787:26604 257.13776:151322 258.14182:8748 262.1813:1945 268.85287:6364 272.0419:10315 280.19107:2912 289.92111:2156 312.84097:2951 324.18195:1857 342.19196:15248 349.52576:1590 368.16943:25454 386.18503:287941 387.18707:25671
N-[(1E)-2-(3-nitrophenyl)-1-azavinyl]-2-(3,5-dioxo(2H,4H-1,2,4-triazin-6-yl))a cetamide	6.676	317.06259	[M-H2O-H]-	317.06403	72.00745:2169 74.02328:1739 84.35016:1610 92.77747:1628 96.83794:8145 96.84518:3118 99.92403:6222 102.98732:1820 111.98782:5129 116.04812:6864 129.99837:3174 130.06447:5763 132.01349:8588 139.9832:15717 141.99794:2472 157.9937:143450 158.99492:9836 159.09178:3256 160.00929:37903 173.9968:2527 203.08257:31497 206.6109:1467 218.99686:1914 222.3735:1494 243.06212:23927 244.06456:2435 260.10403:2462 261.07352:7207 271.05713:159452 272.05856:22154 289.06635:207540 289.91641:1800 290.07074:24973 317.06042:158204 318.06467:30738
Derwentioside B	10.319	467.15836	[M+H]+	467.15479	57.07096:39277 67.05472:9669 69.07069:7220 71.08634:47310 81.07047:27114 83.0864:8586 85.10198:27999 95.0863:35692 97.10148:3299 109.10181:8221 116.05344:11908 130.03226:49578 137.13248:3399 140.94341:38137 141.10202:42230 155.14391:18163 195.01814:10035 201.10551:10702 205.04111:7708 221.03461:10787 223.04938:241859 224.05406:9787 239.23802:3596 246.06813:25974 263.02802:23701 265.04327:410720 266.04517:25093 281.03879:67199 283.05533:946863 284.05634:74138 297.06873:8993 304.05609:8406 306.06729:235991 307.07465:17920 467.15439:991546 467.41254:78512 468.16104:147711 468.4115:9164
LPE(18:0)	13.465	480.30939	[M-H]-	480.30902	78.95716:154824 96.84116:20010 96.95862:18565 121.99919:7966 140.00998:110486 152.99452:46218 158.9776:8230 168.04158:8565 174.95631:4555 196.0358:418841 197.03917:12191 214.04759:97566 224.06895:47345 242.08043:7839 253.21753:10867

					255.23204:539938 256.23663:51573 283.26309:4466496 284.26944:414226 419.25525:7308 480.3103:1368712 481.31076:193241
3-Indolehydracrylic acid	8.731	206.08127	[M+CH3OH+H]+	206.08171	69.74413:12665 115.96455:19262 117.0583:5130 118.0653:777136 119.06923:42635 121.06573:6303 130.06471:613534 131.06937:44126 132.08138:167260 133.08473:16327 133.97447:4886 142.06541:18342 143.07285:13129 144.08119:154963 145.08368:18492 146.05983:649082 147.06441:41200 160.07613:1125685 161.07991:94403 170.06102:350802 171.06432:36150 188.07018:1100278 189.07487:107427 206.08083:805305 206.15387:4700 207.08372:82204 207.11449:6887 207.1767:4815
Tetrahydroharmol	10.321	203.12106	[M+H-H2O]+	203.11844	59.99109:56783 60.04541:4511 69.2529:19066 72.08165:7191 86.00632:139491 87.00976:5449 88.02232:513498 89.02546:9019 99.04498:4126 112.07622:5174 116.05351:1762557 117.05646:57217 126.05483:4690 130.0325:2221251 131.03586:87358 143.08586:5073 203.08371:5572 203.12762:53226 204.13913:38995

Differences in metabolites between the YGS group and the ND group

Metabolite name	Rt(min)	Expreiment Mz	Adduct type	Reference m/z	MS/MS spectrum	PPM
cis-6-Nitro-p-mentha-1(7),2-diene	10.986	180.1019	[M-H]-	180.10245	51.48626:1331 59.01207:11089 66.03323:2053 69.88133:2630 71.01202:14829 73.02773:5747 83.02377:37467 86.98489:1463 89.02298:12387 92.9184:15757 94.02829:4929 98.02278:1791 101.02258:16308 101.91824:1383 108.04317:6616 109.03953:1609 112.92094:6847 112.98356:7547 113.58508:1341 135.07977:2952 135.91148:1819 136.07393:4462 136.08296:11232 136.90929:15090 136.98174:1527 137.03345:1613 138.01852:1539 138.0293:1660 147.61073:1378 156.492:1412 163.06038:2101 166.02699:5824	-3.053817424

					180.02887:2204 180.07469:9198 180.10219:273554 180.81264:1833 180.92346:18489 181.04808:4276 181.07169:38079 181.1057:23771	
Evodol	10.004	529.17059	[M-H]-	529.17151	57.03298:19943 59.01246:115411 68.79816:11163 71.01253:95420 72.99157:15108 73.02771:10409 75.00741:58070 78.95764:3216 83.01214:2618 85.02802:273164 86.03099:7808 87.00725:39934 89.02283:26082 95.0123:87752 96.84156:3367 99.00681:104426 101.02246:2513 103.00228:45547 109.02763:3137 111.00743:7377 113.0234:549356 114.02605:20788 115.00229:26910 117.01807:56618 124.0059:2307 129.01857:39379 133.0132:4132 139.00215:12864 157.01236:12612 175.02422:118927 176.02797:3588 267.06396:3495 269.04526:8098 270.05255:38991 271.05716:2997 283.05951:88965 284.0654:17665 295.05908:47120 296.06598:3659 304.23737:6560 310.11935:3591 321.11438:43572 323.09406:11228 338.11761:45344 339.11649:4026 353.14188:784999 354.14459:110420 395.14804:9800 396.1557:2223 482.25278:2303 512.27179:10271 529.17529:77829 529.27783:9528 530.17981:16225 530.27246:50664	-1.738566765
Methoxyphenyl acetic acid	7.232	165.0546	[M-H]-	165.05573	50.93742:1291 59.01212:50687 65.01261:6052 65.99712:3146 66.03339:13534 67.02861:2054 68.0126:1531 69.51434:6161 72.99163:2906 78.95699:2945 81.04362:2898 82.03947:5135 93.03336:34509 94.02829:5117 94.99119:7856 95.02281:3121 96.00731:1575 96.0439:1558 96.95827:7691 96.96828:15841 97.03892:2078 98.02277:1628	-6.846172502

					102.29301:1379 108.93593:1747 109.03953:1357 119.04861:11652 121.02739:7367 121.06413:46996 122.02354:1684 122.03504:2418 122.05934:8391 122.89236:1649 123.01779:1596 123.05404:2246 124.05046:4970 125.03384:2404 129.42952:1410 136.93021:91167 137.08214:2207 138.01852:2622 138.02776:2261 138.05544:1605 139.05002:6152 147.04469:17001 164.8345:51574 164.89464:14156 164.92635:16193 165.02917:6109 165.05331:210631 166.01279:1634 166.02495:1528 166.06165:17342	
Cynaratriol	11.066	281.13937	[M-H]-	281.13889	69.89346:5483 87.0071:340945 88.01102:5325 96.95841:11314 163.1115:16896 191.14349:31561 193.12151:17178 213.15028:6131 219.13774:63545 220.14153:5313 233.32646:1515 235.13524:4962 237.14861:4929 238.21904:2471 280.62097:25681 281.14044:672782 281.24478:18590 281.62421:7244 282.1449:72628	1.707341165
Aldosterone	9.659	405.19122	[M-H]-	405.19189	58.60857:1313 90.74675:1365 112.44896:1534 125.05902:1760 137.0593:5303 189.35707:1430 201.82286:1314 219.30763:1344 257.09863:1488 299.16302:3070 301.18027:29132 302.18427:1533 311.16312:9218 327.15964:12625 329.1767:120682 330.18112:13647 341.17514:1494 405.26917:1953	-1.653537538
FT-sulfone; C6H9F3O4S	6.567	233.01149	[M-H]-	233.01009	79.95575:256128 80.96335:32264 96.95824:68473 99.9239:13643 100.92433:24565 101.92308:11192 103.91817:12067 107.04883:11533 115.91922:11980 116.91972:21133 117.9189:2981 117.9274:12200	6.008323502

					120.01966:3498 123.04363:26042 132.86647:17463 134.86401:27015 135.04401:9007 136.0152:3474 136.93153:2696 138.03072:93468 139.03444:3455 144.94127:3489 144.95782:7655 145.04337:11887 145.92267:8371 151.03932:25425 153.05351:1709304 154.05687:127215 158.93202:3484 164.92589:11402 175.93938:2877 176.94162:24552 179.03407:8249 187.93324:13009 188.94307:22552 189.03368:47542 189.93915:3127 203.00026:143986 204.00499:3359 217.98601:3855 219.92497:2698 232.76311:7222 233.01259:1234695 233.1545:38902 234.01477:98482 234.15698:3430	
(4-formyl-2-methoxyphenyl)oxidanesulfonic acid	7.878	230.99617	[M-H]-	230.99692	61.24049:1413 70.57959:2037 75.492:1416 79.95578:3018 80.96336:2325 85.94473:1440 99.92396:9357 100.93193:1589 112.40253:1357 115.91861:10870 116.92685:6659 136.01514:84696 137.01813:2841 143.95163:2079 144.92133:2870 151.03876:532856 152.04185:16931 175.94125:11653 186.93382:2607 187.94177:12844 218.92296:1417 230.99481:71661 231.02916:13947 231.08803:1696 231.99214:17229	-3.246796537
5-Methoxysalicylic acid sulfate	6.972	246.99159	[M-H]-	246.99182	69.90848:3091 79.95592:88320 96.84155:2329 96.95847:175458 103.91842:3816 108.01981:56294 116.92692:2676 118.94203:9519 123.04372:155268 124.04651:6066 132.0437:1665 152.00999:300159 153.01405:14935 162.93205:3258 167.03381:2156506 168.03738:153765 178.94778:2120 179.03181:4024 198.04251:1962 200.87984:2236 202.99997:62467 204.005:1688 213.06824:13682 246.99083:446153	-0.931204928

					247.99553:29772	
Medicanine	1.38	160.09695	[M+H]+	160.09737	55.05505:48401 58.06624:8249 60.08159:128170 61.04079:10545 68.79278:49285 69.03422:11065 69.07068:9833 70.06618:30682 71.04984:10753 71.06916:12890 72.08163:7335 81.05851:8468 83.04977:40487 84.0815:49391 86.09712:14773 88.07632:8011 96.08127:64938 97.02891:10287 100.07661:29008 101.05987:193245 102.05579:10298 105.07004:8867 113.96425:75670 114.05556:32839 114.08327:32878 114.09137:4842495 115.09528:246397 118.06532:41457 124.05872:15175 130.08725:12417 131.97488:35891 132.10152:12057 133.03523:24913 142.07024:11482 142.08644:76015 151.04576:10970 160.03583:41115 160.09724:4564081 160.1335:558713 161.07193:8682 161.10127:236603 161.13593:35867	-2.623403495
3-Oxooctadecanoic acid	13.211	297.24298	[M-H]-	297.24298	57.4399:1344 59.01211:189397 60.0156:1913 64.23315:1503 69.05704:2753 69.06086:4205 86.54566:1361 89.68456:1286 93.91769:1460 96.84156:1896 106.03918:6441 129.71767:1384 172.06264:1686 176.04568:1301 183.01161:2791 243.91273:1286 268.19345:1539 297.15131:202017 297.24347:181566 298.15463:27473 298.24725:24408	0
1-Ethylpyrrolidine	13.427	100.11257	[M+H]+	100.11208	51.25271:6338 53.58773:7738 55.01876:7106 55.05521:310010 56.05027:47903 57.05857:8384 58.02977:11088 58.06584:30993 59.06131:280083 59.93129:12707 60.04586:5958 66.11333:5923 68.92666:9445 68.92992:37442 70.06621:21504 71.02999:15264	4.894514228

					72.0451:29936 72.08171:269671 78.03793:10785 82.0659:42497 82.24175:7038 83.02469:7797 83.08643:16011 85.08929:11711 100.02445:8027 100.03963:12501 100.05103:8181 100.07667:390726 100.11275:748992 101.03494:46747 101.0715:15798 101.07921:9192 101.11584:26950 101.65221:7377 102.1811:6193	
N-{4-[(1-methylimidazol-2-yl)carbonyl]phenyl}(3,4,5-trimethoxyphenyl)carboxamide	8.366	396.15317	[2M+H]+	396.15536	69.64206:2562 69.64536:5498 70.06564:3956 89.06053:3411 133.67792:1792 138.05257:146743 139.0563:6684 142.06384:1948 152.03125:9686 179.19026:1607 249.58524:1671 253.09558:17842 261.04599:1704 267.11395:2794 281.09189:51317 282.09137:3881 299.09937:267157 300.10239:32795 368.15506:1919 396.15402:1106159 396.6405:2634 397.15793:176960	-5.528134215
5-Hydroxyferulic acid	7.231	209.04453	[M-H]-	209.04552	50.51936:1166 51.83491:1373 59.0121:8585 65.9778:1240 68.01208:1461 71.01299:1434 89.02289:1731 93.03313:2413 96.00732:1519 103.91845:7111 104.9193:2492 113.83349:1197 121.06403:6282 123.9444:1618 124.03868:2652 125.03385:1279 127.86847:6126 130.48758:1457 159.85966:5452 164.83434:1755 164.9267:1664 164.98497:2593 165.05525:59949 165.84251:5083 166.05743:2575 180.95802:2018 208.95448:1860 208.97453:2332 209.04332:7245 209.08345:5432	-4.73581065

3-Oxotetradecanoic acid	12.304	241.18062	[M-H]-	241.18037	54.59089:1957 59.01234:953301 60.01554:22356 60.99175:3471 65.67184:2478 65.9971:2261 69.64699:8388 87.00719:12589 99.30262:2345 106.03918:8216 121.05009:2278 134.03461:1968 142.60005:2121 146.4726:2008 157.05234:3261 158.47125:2058 160.0515:2262 172.06262:2935 177.16309:3108 181.15877:78057 182.16219:4201 199.07477:3167 200.09366:2507 214.0713:9270 223.17134:2341 225.14801:3463 241.17909:479372 242.06978:2360 242.18477:63980	1.036568606
1-(Diethylamino)ethanol	13.184	118.12286	[M+H-H2O]+	118.12264	53.03948:1691 55.05524:66182 56.05856:2745 58.0659:43039 59.06141:11671 59.07378:51293 71.02942:1709 72.08168:181108 73.06556:54364 73.0851:7260 74.06911:1488 77.03493:2009 79.05531:1500 87.04442:4862 87.05597:1987 91.05507:13411 95.05009:7723 96.04482:6022 100.11276:226869 101.11558:13225 117.07034:1672 118.06522:12917 118.08604:204491 118.12258:73997 119.06093:18173 119.08572:24333 119.12611:2512	1.862471072
2-Ethyl-1-hexanol sulfate	13.495	209.08458	[M-H]-	209.08476	65.01305:3430 68.35537:4408 68.35912:9908 78.95692:8509 79.95505:11322 82.0394:11609 95.95105:6307 96.9584:265774 118.99118:2425 124.03857:2891 126.01812:2254 156.0546:2436 164.92654:2983 164.98683:9392 180.96478:2974 182.04553:3432 183.03027:3285 183.06503:11514 208.82257:8326 208.9772:6146 209.08331:1039015 210.04138:2310 210.08881:60614	-0.860894883
Methyl 3-[(1E,3E)-3,5-dimethyl-1,3-	11.675	437.1925	[M+Na]+	437.19342	52.73198:2168 58.06582:12020 60.0816:2622 65.09783:2350 69.82485:5289 86.09713:3394 89.06059:26607 95.08612:2749 98.98515:2602 99.62807:2399 133.08644:15653 140.14348:3369 155.01137:8940	-2.104331762

heptadien-1-yl]-8-hydroxy-6a,8-dimethyl-6-oxo-6a,8,9,9a-tetrahydro-6H-furo[2,3-h]isochromene-9-carboxylate					177.11324:2493 184.07443:4712 195.12151:2887 210.18434:2707 239.15048:20930 303.12286:61841 376.25882:3022 419.17938:2415 422.16617:2395 437.19122:3435821 438.1983:557010	
1,2,3-Heptadecanetricarboxylic acid	9.538	371.24344	[M-H]-	371.24393	57.0329:2162 59.01236:3681 71.01239:1898 71.04872:8196 73.02773:17523 83.04872:6047 84.01974:6675 96.83884:11225 99.07986:16184 113.09522:8185 114.05493:4849 123.08051:11091 125.059:7813 125.09643:17197 127.11082:10117 128.03452:3477 129.09077:2339 135.11697:5717 137.09613:18449 141.09093:31301 149.0948:2057 153.12711:9397 155.10655:19559 167.10535:31909 169.08566:2034 171.13745:16474 179.10741:18201 180.14571:2258 197.1178:59600 198.12181:2877 215.1268:73150 216.12982:3588 233.19144:1890 235.20703:18321 236.2094:1892 242.14836:5893 251.20041:5472 253.21664:12530 257.09467:7348 261.18555:3141 279.19708:60486 280.19989:3405 293.46945:1816 297.20456:127574 298.20834:13679 353.2301:6500 371.24396:696979 372.15466:2706 372.25006:80503	-1.3198869
8-{[2-(4-ethoxy-3-methoxyphenyl)-5-methyl(1,3-oxazol-4-	8.379	440.14368	[M-H]-	440.13983	69.84422:4972 114.05611:63305 127.04939:25158 128.03455:3615 136.93004:1894 149.03441:10103 158.97766:2892 170.09741:3201 172.04552:3143 174.95638:3786 193.49907:1736 213.10324:3485 214.08698:14438 229.10202:2008 244.10072:1838 251.08345:7506 257.09296:131863 258.09854:8377 279.07343:5302 291.08429:17242	8.747220173

yl)]methylthio}-2,9-dimethylhydropurin-6-one					302.08966:2884 303.06976:15043 311.08826:19290 321.08203:1897 328.1647:12181 329.09708:24037 330.10226:1966 335.05981:34242 336.06354:2004 350.14633:7012 358.70804:1867 366.1279:2774 372.15387:78445 373.15771:9417 394.13681:412062 395.14069:44244 412.13354:101296 413.13611:14508 440.13074:119582 441.13068:17732	
Aspartylphenylalanin	6.715	281.11295	[M+H]+	281.11319	70.02956:13270 71.0192:2311 73.02959:11662 84.96065:2157 87.04462:11396 88.04028:157090 89.04379:3261 91.05492:25282 95.08614:2743 103.05459:2561 107.0496:3676 120.08136:337356 121.08463:24136 130.06491:18292 131.04944:18747 136.06203:3163 149.06003:16265 157.06523:3282 166.08551:338856 167.0907:23455 172.07529:19418 175.08763:111888 176.0894:10005 182.06125:9377 189.10182:3651 190.0878:13392 200.07109:144569 201.07269:11996 217.09859:17802 218.08191:8426 219.08722:1935 221.09088:68367 222.09499:9397 228.06725:8204 235.10915:114167 236.11023:12006 245.09262:13592 246.07732:78203 247.08:11146 263.10263:29600 264.1088:3303 281.11496:46393 282.11832:3423 282.21722:2294	-0.853748627
Octamethylenediamine	13.431	145.17012	[M+H]+	145.16988	58.40471:9381 59.63844:9966 61.48924:10366 62.92982:103041 69.39963:24112 69.40347:38555 72.08167:11667 80.94032:16616 84.95992:14496 100.07666:24914 100.11275:133486 103.95644:63420 118.96721:11372 120.96478:12434 121.96647:720845 122.96522:53910 125.32649:10736 129.96356:10349 144.98248:124182 146.05988:19713	1.653235506
1-O-Hexadecyl-sn-glycero-3-phosphocholine	12.75	482.36041	[M+H]+	482.3605	57.03444:7183 57.07079:51539 58.06584:30578 60.08159:189161 71.07371:20392 71.08622:42503 75.04475:54710 85.10188:31120 86.09711:478054 87.10065:7517 89.06057:38391 104.10735:4312998 105.10773:17376 105.1108:123167 124.99973:262910 133.08618:19371 181.02644:13413 184.0743:708617 185.0779:25979 283.17737:18836	-0.186582442

					464.35178:7296 482.35883:1232506 483.36472:184773	
3beta,6beta-Dihydroxynortropane	1.404	144.1019	[M+NH4]+	144.10245	58.06586:1307798 69.84534:83234 70.0662:45016 71.04986:101348 72.08165:46487 84.08147:2024258 85.02901:48448 85.08482:62671 98.06048:157340 98.09721:328173 99.04507:343119 102.05585:287405 144.10254:18762762 145.10544:1191167	-3.816729001
Palmitoleic acid	12.293	255.23209	[M+H]+	255.23187	55.05522:63317 57.03445:4505 57.07091:123328 67.05521:27723 69.0708:143314 70.06577:50247 71.04986:4482 71.08631:83856 73.06554:3946 79.05463:5419 81.07034:70401 83.04979:6804 83.08652:149140 84.08163:55198 85.06547:6218 85.1019:53624 89.06053:5069 93.07087:30752 95.0862:62819 96.84789:24558 97.06523:37549 97.10162:92740 98.09641:5900 98.10471:3769 99.08051:4134 101.05992:5004 107.08549:44791 109.10187:40647 111.08042:26280 111.11707:36208 115.07553:7188 121.1012:55752 123.11671:24499 125.09668:28577 125.13242:4237 133.08621:4229 135.11714:60884 136.11925:4687 137.13251:16025 139.11249:26455 149.1326:56005 151.14972:3704 153.12701:24769 157.12125:4218 163.14871:32164 167.14201:23241 177.16469:11647 181.15822:13353 195.17583:4510 219.21075:76871 220.21413:14126 225.50838:5374 237.22237:40537 238.25145:13358 239.14685:4794 255.11665:5988 256.2644:871726	0.861961322
LPC(P-16:0)	12.595	480.34473	[M+H]+	480.34479	55.05515:16695 56.05058:16000 57.03444:47315 57.07082:39786 58.06625:26038 60.08158:304102 62.06105:72813 67.05524:10642 69.07069:53358 70.33955:17645 71.07368:97273 71.08621:37372 81.07098:15465 83.08644:64255 85.10193:30310 86.09709:1726370 87.10065:46137 95.08612:36751 97.10148:50102 98.98515:50272 104.10734:3543513 105.11057:72924 107.08636:11113 109.1018:18935	-0.124910275

					111.1169:14931 124.99973:973547 151.01585:8973 155.00954:13342 181.02631:735446 184.07426:1524312 185.07567:49791 240.09988:677860 241.10541:29420 265.25226:10768 308.29355:32150 318.55392:9096 339.28775:303645 340.29166:38651 350.3754:9060 480.34528:4430660 481.3454:691261	
4-(3-Hydroxybutyl)-3,3,5-trimethylcyclohexanone	12.183	211.16965	[M-H]-	211.1698	65.01311:4688 66.0078:1572 70.03784:1991 70.04173:2324 89.01253:2710 90.00767:1704 91.02782:1973 97.06431:5418 99.92404:1665 106.03921:18214 111.07959:6065 113.09526:1641 115.07507:13801 116.02324:1626 125.92704:1563 129.09116:12229 130.03917:2130 142.0397:2043 145.0506:2239 154.09827:10995 157.05028:10831 158.03513:1853 167.10555:2012 167.14243:2485 168.114:8894 169.05028:2307 171.06734:2255 183.17319:9201 184.04922:3128 184.06429:8786 185.04544:11462 185.08134:2598 210.96852:6327 211.05864:5154 211.16917:323729 212.05736:10482 212.0919:2762 212.17422:34375	-0.710328844
Cinnamoylglycine	8.95	204.0656	[M-H]-	204.06662	69.11098:7145 75.00731:2151 79.95585:2081 82.02819:6791 98.90496:2341 106.0392:29117 107.0352:2111 107.04886:2175 116.04932:1716 117.01213:2242 117.06976:13856 119.04794:2088 123.04369:6786 124.04664:9264 128.04967:2940 130.06456:36292 131.06772:1890 132.07971:1830 133.06416:1324 142.0654:1745 143.79953:1474 147.04427:3084 157.99933:2778 158.05923:16437 158.9948:2172 160.07468:134245 161.0023:12378 161.07805:11871 174.05562:6313 176.97095:5282 178.06114:1986 186.05473:6501 197.60976:1613 203.93947:10429 204.00592:9171 204.06587:68775 204.94841:9203 204.99124:10654 205.06763:7749	-4.99836769
1-	13.467	463.25867	[M+2H]2+	463.26022	67.05488:143720 69.07077:92977 69.39907:13474 69.40237:53009	-

[[(2S,3R,11bR)-3-ethyl-9,10-dimethoxy-2,3,4,6,7,11b-hexahydro-1H-benzo[a]quinolizin-2-yl]methyl]-7-methoxy-3,4-dihydro-2H-isoquinolin-6-one					71.08636:107835 79.05464:27918 81.07044:162990 81.15483:11964 83.08643:60528 85.10195:70954 89.349:10836 91.05434:49508 93.07069:71179 95.0863:216542 97.1015:65199 99.04499:10935 105.07008:47432 107.08639:59794 109.02837:14779 109.10204:100847 111.11693:14904 117.07035:20209 119.08543:62148 121.10116:64207 123.11666:53600 131.08507:21059 133.1008:56750 135.11719:19842 136.93086:83076 137.13252:17463 147.11694:47994 154.942:225221 155.00958:19704 157.10258:11207 171.11772:18605 172.95198:298242 175.14911:14682 177.95683:17574 185.13304:11923 189.16351:17757 190.96346:92715 195.9696:45170 196.95157:20655 203.1799:67460 210.96687:72648 212.9482:15226 214.96355:58862 220.95053:27740 228.97847:180901 238.96109:60395 246.98799:22851 252.05666:12302 256.97406:72978 269.22562:62422 280.97583:55715 287.2384:63246 292.30472:13197 297.92044:12113 298.98203:318788 299.98431:17023 303.99081:13737 305.24634:24869 316.996:530027 317.99933:21397 322.00043:77092 335.00217:188127 336.00433:10892 340.01257:98186 341.30597:285694 342.30701:48384 343.19135:84235 358.01843:24974	3.345851712
PE(18:1/18:1)	12.472	744.55426	[M+H]+	744.55377	55.05516:42496 57.03442:28070 57.07084:130196 58.06664:51239 59.05008:68298 59.07414:13080 60.0816:233152 67.05521:158742 67.54954:16454 67.55375:84272 69.07069:112713 71.07355:51976 71.08626:133195 79.05523:40852 81.07027:239233 83.0864:133070 85.10191:82102 86.09711:1026794 87.10027:37104 91.05509:17667 93.0705:46839 95.08611:235515 96.0894:10801 97.10139:73520 98.98512:31048 99.08139:11419 104.10728:136200 105.07103:11153 107.08607:35899 109.10174:149598 111.11703:42511 117.0919:27735 119.08535:11637 121.10098:47331 123.11661:76461 124.99965:484595	0.658112308

					133.10071:18870 135.11662:49539 137.13243:26822 149.13269:26049 161.13199:9298 163.14879:9186 184.07417:12178996 185.07802:460490 186.07745:71550 245.22653:7892 263.2355:37816 265.25214:21175 267.26706:21197 306.28009:42152 308.29343:10128 310.31061:8565 337.2753:18403 341.30588:24540 504.34296:31353 603.52716:1116513 604.54071:333925 744.58051:286808 745.58307:110762	
alpha-Ketoisovalerate	6.696	117.05738	[2M+H]+	117.05458	55.05516:13429 58.06584:39010 59.07381:44193 69.96897:10589 70.06565:6147 71.06918:12034 72.0817:268918 76.93372:29008 91.05518:146130 117.05721:120332 117.07035:24217 117.95958:8784 118.06536:821017 118.0872:239102	23.92046514
10-(Phosphonooxy)decyl methacrylate	11.129	321.14694	[M-H]-	321.14725	52.12137:1759 60.9263:1902 62.15658:1897 68.83661:11068 74.02631:1990 78.95764:677667 81.04432:1879 88.14672:1661 94.02829:9133 96.96824:1211006 98.79776:1859 101.50184:1900 130.08559:3966 130.47908:1803 146.95973:8412 174.95395:14980 186.74503:2127 195.24937:1870 241.81284:1778 286.57465:1959 321.14755:189820 321.20761:13998 322.14801:19356	-0.965289287
Decarestrictine D	8.927	217.10747	[M+H]+	217.10709	53.42403:1676 55.01875:1696 55.05516:27980 67.05524:2891 69.07069:2914 71.01749:1533 73.02942:2642 81.07098:1796 83.04987:53301 83.08656:12916 84.08146:2005 85.06562:16563 87.0447:11511 89.02408:10500 89.06061:52330 101.06007:58374 102.83071:1505 111.04482:226100 111.08137:2797 112.04781:11357 112.07622:1717 113.06055:5478 127.07569:18533 129.05504:84967 130.05774:2697 130.08588:2467 137.05942:3098 146.96135:1771 147.06616:7345 155.07007:372133 155.88634:1587 156.0733:22197 157.10069:1839 172.09631:2036 173.08134:339024 174.08324:27007 176.9725:1677 190.08722:1884 190.1071:1560 199.09758:35085	1.750288302

					200.09239:2281 200.12729:7757 217.10785:316357 217.19577:3584 218.1097:24221 218.13995:2572	
Agomelatine	6.262	244.13559	[M+Na]+	244.13318	55.05515:127779 57.05815:28735 60.05644:39574 68.05045:94019 68.82644:196775 69.07069:150964 70.02949:185803 70.06622:2120391 71.06917:140238 72.08167:1665224 73.08509:31347 82.02924:32118 84.08149:1221035 85.08481:53857 86.0609:461503 86.09717:4476924 88.04028:1132224 97.03992:259970 98.02457:128016 99.00852:41494 112.08747:779616 113.09133:29068 115.08723:130790 118.08606:252939 118.57924:27414 125.0351:165191 126.01859:29810 127.07423:164177 129.10236:1129953 130.10558:43120 132.06555:5885034 141.0717:48495 143.04543:323188 147.11353:104685 150.01866:229149 150.07686:27854 153.56279:36032 155.56012:34528 164.07379:45839 167.56084:33822 168.02879:53758 171.03917:85783 172.12201:103930 185.05659:54408 194.12497:43571 195.07758:38502 202.78484:30624 208.78682:36246 213.08675:36269 218.11855:99763 218.45195:33461 224.22913:23685 226.15477:275488 229.15569:100749 244.10873:57567 245.14973:767991 246.17963:252891	9.871661034
Madecassic acid	12.127	487.34268	[M+H-H2O]+	487.34201	69.07069:7937 71.0294:2503 73.0294:8049 81.07021:22302 83.0498:12940 83.0864:3899 86.09713:3143 89.06051:8217 91.05513:2436 93.0705:12659 95.05007:11433 95.08622:63594 97.06519:7764 104.10755:13739 105.06998:21969 107.08625:36158 109.10171:28062 111.08024:3670 119.08545:61968 120.08879:3205 121.10118:42751 123.11674:16258 131.08502:2962 133.1008:45364 135.07991:3257 135.11714:46710 137.09593:2592 137.13248:7986 139.07484:17145 139.11227:3684 145.10194:18746 147.11745:13633 149.09648:8406 149.13275:9411 153.091:11073 157.10068:9517 159.1161:18968 161.0952:7946	1.374804524

					161.13234:15220 163.07576:8171 163.14885:7948 165.09113:3975 171.11743:21912 173.13312:133702 174.1356:9724 175.11169:8859 175.14906:167257 176.15143:10809 177.09088:8248 177.12665:3731 177.16486:15247 179.14246:2944 185.133:4076 187.14886:180100 188.15092:10223 189.12643:3437 189.16348:70889 190.16675:4079 191.1431:21482 191.18066:109401 192.18239:9332 193.15894:17557 197.13251:10591 199.1487:20092 201.12715:7291 205.15819:43587 206.16229:4461 207.17387:12289 209.19081:7229 213.12802:3360 213.16344:4345 215.14296:109448 215.17593:2587 217.15956:44846 219.13652:21699 231.13914:2638 231.17581:4350 233.15518:266765 234.15741:22115 235.17029:39378 237.14949:117554 238.15028:8814 241.19774:2268 243.13844:83878 243.17453:4173 244.14127:8436 251.16208:28178 259.17203:3839 261.15012:115776 262.14944:7049 269.19202:10857 279.15756:22215 287.20139:2665 311.19916:2776 315.19437:11917 333.20627:16121 405.31342:8687 423.32837:25485 424.32983:3234 433.3071:7841 441.33865:17077 442.34262:3585 447.20883:2447 451.31741:23034 469.33572:56765 470.33069:11826 487.34039:700095 488.34271:118285	
3-Hydroxyhexadecanoic acid	13.075	271.22717	[M-H]-	271.22733	59.01211:214414 60.01516:1747 65.0131:4264 65.9966:2611 66.00779:2344 69.00755:2688 70.60439:2577 78.95698:1841 82.03945:2468 83.0229:1880 96.47383:1201 99.92403:1252 106.03918:7312 116.92693:1374 120.05585:1765 121.0501:1398 131.04492:1449 133.0394:1670 148.04883:1571 157.05048:2358 185.04549:1487 187.06248:1614 191.01628:1762 225.22174:2997 244.08263:1617 271.09528:2453 271.22659:178540 272.2291:19868	-0.589911054
2-	8.461	184.09694	[M-H]-	184.09737	69.06467:6317 69.06847:4053 74.02328:897435 75.02023:2053	-

Hepteneoylglycine					75.02636:15284 83.02361:7872 95.91546:2057 97.06421:20557 98.02263:15633 112.03841:2590 116.92699:13149 123.07991:8148 138.09087:56272 140.06952:9790 140.10728:197500 141.09074:29800 141.10963:12698 157.05048:7475 166.08582:6739 183.91119:15019 184.09663:2074078 185.04309:2363 185.0813:14777 185.10054:149309	2.335720494
7b,9-Dihydroxy-3-(hydroxymethyl)-1,1,6,8-tetramethyl-5-oxo-1,1a,1b,4,4a,5,7a,7b,8,9-decahydro-9ah-cyclopropa[3,4]benzo[1,2-e]azulen-9a-yl acetate	11.674	432.23895	[M+ACN+H]+	432.23801	55.05477:6508 57.03405:13484 61.02905:6875 69.03426:102193 69.88301:5631 69.88633:8329 71.04987:6281 81.03432:16262 83.04988:33204 85.02902:24609 87.04442:16127 89.06053:15458 91.0395:17142 91.05515:17207 99.04498:7814 103.03971:5887 107.08641:103834 108.08866:4357 111.04465:37935 116.24461:3671 119.08545:1347726 120.08879:90782 129.05496:67776 133.06435:33786 133.08623:3680 135.07991:499136 136.08458:26057 147.06613:43613 177.09093:3738 281.13593:50508 282.14081:7365 296.12894:3856 397.30814:4910 415.20816:37040 416.21625:5580 432.18164:25667 433.33289:4156	2.174727762
Cortisol	9.589	363.21661	[M+H]+	363.21658	71.01522:2084 79.05463:3060 81.07029:9001 83.04978:10679 91.05515:5294 93.07068:6821 95.08613:9927 97.06531:56289 98.06877:2625 99.04497:7030 105.07006:8593 107.08637:10273 109.06523:29548 111.08025:2770 117.07034:2526 119.08543:19768 121.06461:193427 121.10114:7295 122.06839:13838 123.08041:27035 124.08495:2131 129.07025:5830 131.08534:13155 133.06438:2566 133.10217:12040 135.08005:23577 135.11716:3378 137.09595:3454 141.07011:6282 143.08592:17029 145.06548:3449 145.10191:25320	0.082595348

					147.08125:13694 147.11702:25998 149.0965:8742 149.13277:3137 155.08646:9241 157.10071:20173 159.08011:9807 159.11638:18347 161.09555:15681 161.13208:6645 163.11136:20611 169.10204:19012 171.08162:6738 171.1178:16960 173.09621:15645 173.13312:9485 175.11148:18860 177.12666:3469 181.10272:6842 183.1165:17314 185.09718:8274 185.13318:14056 187.11261:28493 189.12666:11137 195.11635:10398 197.09579:3326 197.13254:7057 199.11351:6359 199.14815:2590 201.12718:9355 203.10841:2009 205.1219:5390 207.11736:5860 209.13297:13062 211.11223:3520 211.14714:7510 213.12851:13206 215.14305:6936 219.13643:1993 221.13438:3353 223.11238:2606 223.147:11044 225.12682:18558 225.1651:5748 226.13274:2673 227.14235:7609 229.15898:3110 231.13918:3532 233.13165:2255 237.1254:2632 239.14334:17712 240.14607:1970 241.15842:29226 242.16345:3044 243.13861:2844 243.17458:10071 245.15384:1998 249.16289:14719 251.1451:15535 251.17841:7441 252.14394:2256 253.15706:5337 263.14258:5459 263.17905:3376 265.15805:6126 267.14294:2850 267.17581:52674 268.1817:14407 269.15857:3915 269.1918:42272 270.19177:5679 271.16742:3469 273.16461:2599 279.17624:12556 281.15454:7252 281.19034:19723 282.19025:3007 285.18582:11590 286.18857:2036 287.20145:6518 291.1748:20550 292.17673:3490 297.18555:34040 298.18881:3270 299.20227:7719 309.18369:64124 310.18567:12404 315.19507:10459 327.19696:90475 328.19992:17969 345.20822:45880 346.20816:8086 363.21414:307385 364.22009:77260	
5-Oxooctadecano	12.495	297.2431	[M-H2O-H]-	297.24298	52.92169:1742 65.9966:1689 69.67071:5676 85.00642:1458 183.01163:6901 251.23824:6051 253.21764:1821 265.1987:1597 279.23111:11318	0.403710123

ic acid					295.22711:2784 297.15134:297928 297.24341:884144 298.09601:1738 298.15494:35946 298.24713:108517	
6-Hydroxy-3,4-dihydro-2(1H)-quinolinone	13.461	164.06711	[M+CH3OH+H]+	164.0706	58.9989:26977 69.43143:81206 72.93777:124898 82.01449:1009799 83.01536:46355 90.94813:39184 100.0245:3571101 101.02621:318645 113.96442:657760 118.03501:1688977 119.03613:165714 123.04028:783045 124.04173:55126 131.97508:279908 140.97168:124236 141.05104:415423 142.05261:28308 154.99129:43600 164.04393:29982 165.06503:37843	-21.27133076
A-Homo-3a-oxa-5beta-olean-12-en-3-one-28-oic acid	12.877	469.332	[M-H]-	469.33234	64.85825:1707 69.20643:6337 69.2097:2303 76.7397:1561 79.93821:1454 85.74638:1663 91.37807:1428 96.84247:1595 99.92403:1669 115.28125:1662 115.91899:2991 123.86327:1423 186.93394:3327 329.245:1641 399.02802:1600 425.34464:50600 426.34619:9274 431.95294:1759 469.33014:1789485 470.33429:290989	-0.724433351
Monopalmitolein	12.011	311.25845	[M+H-H2O]+	311.25699	55.01913:1954 55.05521:48537 56.05815:1874 57.0345:51263 57.07084:66657 59.05006:2932 67.05524:66448 68.05843:1853 69.07072:113039 71.04986:8265 71.08632:28143 73.02946:46960 73.06554:2065 76.03989:28968 79.05527:23096 81.07034:95937 82.07365:5505 83.04979:3630 83.08643:101827 84.0895:2428 85.06548:12193 85.10213:14162 86.09714:3809 87.0444:1815 89.06055:56088 91.05514:8863 93.07072:46548 94.07333:1864 95.0862:90331 96.08945:3407 96.84246:3675 97.06519:22191 97.10154:71384 98.1047:2055 99.08144:9498 104.10737:6303 105.07006:3279 107.0706:1748 107.08631:38959 109.10191:42896 111.04473:2514 111.08026:18352 111.11711:24274 113.06054:2229 113.09589:2292 115.07552:2177 117.07033:1820 119.08541:7713 121.1012:81393 122.10457:2601 123.11671:22868 125.09664:12985 125.13242:3389 127.11243:2305 131.08647:2807 133.08626:25499	4.69065771

					133.10066:13075 135.11714:61699 136.12074:2692 137.09595:3449 137.13246:16862 139.1123:10229 141.12735:2440 145.10028:3433 147.11691:6886 149.0965:2260 149.13277:32388 151.14795:3347 153.0551:7630 153.12695:2912 157.10069:2134 159.11627:3252 161.13208:6044 163.14909:12355 167.14403:1870 173.13312:3637 175.14908:2315 177.11325:3034 177.16467:3068 181.04958:6457 184.07445:9090 187.14653:1834 199.14815:3152 209.11629:111109 210.12079:8791 215.17896:10139 219.21039:21840 225.73013:2532 229.19518:2446 233.18906:7070 237.1116:83104 237.22252:24730 238.11546:9637 239.17839:3351 240.10019:3106 247.20528:2624 250.06084:1634 251.20107:8684 255.12492:20118 257.18903:3234 267.14294:1990 275.20117:30218 276.20428:2822 283.1774:2446 293.17395:7153 293.21204:11950 311.18356:8449 311.25656:310194 311.63702:1843 312.25919:66392 312.32687:8420 312.36353:9078	
Ceanothic acid	12.143	485.32791	[M-H]-	485.32721	67.50043:31664 73.02773:5759 82.71668:1578 85.0281:12091 85.06451:27277 96.83796:5442 96.84428:6005 136.5388:1598 138.64346:1656 141.00975:1537 152.99423:2083 200.13664:1568 201.11229:3357 338.88565:1923 355.26569:70006 356.26562:9945 407.3042:1781 423.33148:7677 424.33282:2081 441.34448:83172 442.33942:15613 485.3309:1331434 486.3266:224854	1.44232589
Sphingosine 1-phosphate (d16:1-P)	10.586	350.20953	[M-H]-	350.21017	60.41801:1766 69.87467:3481 78.95761:1779342 87.00797:1581 96.96817:23190 143.99596:1786 222.65997:1850 305.52484:1578 350.20831:810657 351.21127:82901	-1.827474057
PI(16:0/20:4)	13.461	857.51904	[M-H]-	857.51849	78.95732:267966 96.84032:26912 96.96765:56591 134.98299:6584 152.99449:196629 168.04163:69513 174.95412:12462 205.19571:10655 223.0004:95831 241.01219:484152 242.01616:10661 255.23195:660052	0.641385587

					256.23685:51138 259.02203:44464 259.24274:46491 279.23206:35593 281.24817:14620 283.26288:29910 284.26501:17422 297.03912:54234 298.92749:5701 303.23285:479411 304.23697:49032 305.24625:53279 306.25021:41805 307.2644:12642 315.05063:29153 391.22824:124888 392.22922:6359 409.23657:5350 439.2211:13436 553.27618:122123 554.27832:13265 571.29248:30050 573.13812:11218 601.2746:24224 629.20563:10874 797.66479:121749 798.65149:18094 857.5246:1121944 858.526:303761	
PI(18:0/20:4)	12.304	885.547	[M-H]-	885.5498	67.76589:23192 72.99103:3514 75.00668:3876 78.95734:164494 96.84336:7350 96.96764:42197 112.98353:3996 114.9322:2379 115.91898:3718 116.92722:30682 134.98302:8341 152.99457:161776 158.58104:2248 205.19576:11408 223.00102:81695 231.2115:2550 241.01244:379769 242.0162:10683 259.02121:34696 259.24277:32959 260.24728:2751 279.23267:3767 281.24377:2685 283.26309:575547 284.26514:53298 285.22205:3443 297.03918:43649 303.233:367535 304.23676:44063 315.04581:25158 419.25449:103041 420.25861:12594 437.26343:8326 439.23074:13191 581.30627:85212 582.31763:12503 599.32477:24726 601.28162:21434 619.2804:3158 885.56079:865374 886.56073:242113	-3.161877514
Glycylleucine	6.426	189.12367	[M+H]+	189.12338	68.994:16736 72.34647:6029 84.08146:6668 86.09718:2660462 87.09526:7839 87.10061:91802 104.73582:5870 121.96648:6282 132.10164:2047192 133.10526:116049 136.95296:21026 143.11838:1193129 144.12103:60994 148.95329:6914 162.96918:7771 167.03137:6516 171.11133:19062 189.10181:10752 189.12453:113166 190.08722:12216 190.1071:29757	1.533390531
Aminoadipic	1.401	162.07631	[M+H]+	162.07608	55.01861:103337 56.05024:108235 60.08173:210059 69.22339:50337	1.419086641

acid					70.06632:129826 71.04995:110787 84.08153:94174 85.02901:43154 98.06054:4954169 99.0449:188596 99.06371:200290 102.092:59631 103.03979:271659 116.07125:2430648 117.05472:43498 117.07415:97566 117.98022:41734 131.08505:20305 144.06641:1802471 144.10249:165935 145.06895:86554 162.05135:106548 162.07718:567809 162.11226:1617273 162.13924:96815 163.03806:189490 163.11386:74299	
4,5-Diferuloylquinic Acid	9.326	543.15155	[M-H]-	543.15082	57.03292:3478 59.01211:33269 69.272:2929 71.01198:20943 72.99163:5420 75.00687:16263 85.02816:71455 87.00739:11399 89.02288:5607 91.01711:5704 95.01241:15318 96.84067:3283 99.0069:30830 103.00257:11607 113.02243:149132 115.00237:9012 117.01856:10491 129.01733:12079 135.00687:30403 153.01773:19032 157.01317:2708 175.02443:31861 231.10135:18222 297.03958:9307 298.0459:20564 309.03909:16969 335.08987:10223 339.12003:14707 352.09326:8723 367.11987:375210 368.12283:51901 543.15082:67960 544.16101:11703	1.344009754
[(2R,3S,4S,5R,6S)-3,4,5-Trihydroxy-6-[(2S,3R,4S,5S,6R)-3,4,5-trihydroxy-6-(hydroxymethyl)oxan-2-yl]oxyoxan-2-yl]methyl 8-methylnonanoate	6.286	519.24194	[M+H]+	519.24121	55.05513:5593 56.05048:37950 58.06623:17432 69.74356:14496 70.06618:5248 72.08163:359615 73.08507:5307 74.06063:359973 84.04484:74389 86.06076:7725 87.05581:44098 101.07146:87211 102.05572:109934 113.07191:17352 114.05554:6529 115.05083:150007 118.08721:368947 119.08168:18280 119.09031:13296 124.07574:6580 127.04996:79950 129.10228:290964 130.04926:7063 131.08195:27927 133.06142:17392 140.07114:253625 141.06482:52265 142.08627:7263 144.07791:12802 153.06583:5671 157.09695:51478 158.08185:44403 159.07623:77551 167.08252:13371 168.06598:181026 169.05966:68957 170.04477:34550 170.09264:14588 171.07732:7594 172.07268:235919 175.10735:850387 176.11264:38329 181.06065:30810 185.09239:153756 186.07544:22440 186.1236:15222 187.07082:58152 187.10764:12693	1.405897656

					188.1044:7240 190.08218:20191 197.05638:6552 198.08878:90598 199.07082:22138 203.10294:934353 204.10619:34325 206.09209:5279 208.07043:13531 209.05598:13257 210.08896:5941 213.0871:53549 214.1199:24130 215.10417:35627 216.09789:17351 220.07155:5739 224.10173:7220 225.08815:6509 225.1362:14560 226.08122:67513 226.11655:5909 227.06761:12282 231.09912:682597 232.10196:34253 232.12878:110463 235.07129:5047 237.09763:7207 238.08107:43254 242.11372:66860 243.0983:39252 243.1456:161354 244.09305:31784 244.13037:11628 245.12465:5233 252.0983:13701 254.07631:13115 255.10875:85027 256.08936:5123 266.07816:25445 270.10757:118157 271.14175:289962 272.08868:15713 272.14404:18942 273.1217:19283 282.10922:14518 283.10446:68668 285.14444:8020 288.11774:263754 289.14999:701410 290.15494:53743 291.1091:16535 299.06479:7070 299.13367:32191 300.11725:8433 301.11572:26219 309.11655:8034 311.10019:7573 317.14575:18180 323.09909:18174 327.12964:63483 329.10837:6061 338.14648:16273 339.12769:7133 340.12494:7617 341.1087:5470 345.14124:217400 346.14087:16681 348.1283:7531 354.17615:42650 356.15683:26105 357.13968:5561 366.14001:59838 374.16327:15020 382.16962:16479 384.15442:175461 385.15591:17459 400.18536:30405 402.15912:110346 403.16278:6686 418.19653:48523 420.17194:26397 422.16611:6314 428.17758:7701 439.19189:6351 440.17996:16196 455.22598:8297 457.2037:6460 465.2077:30161 473.23572:46041 483.22421:63565 501.23218:140892 502.23352:21184 519.24274:599191 520.24353:89094	
3-hydroxy-4-methoxy-2,3-	13.462	167.01276	[M+NH4]2+	167.01662	56.96548:40471 57.52578:17806 60.2015:17035 63.56096:18012 70.35411:41237 71.31301:17977 78.00068:18824 84.93838:87919	-23.11147238

dihydropyran-6-one					84.96065:7433332 85.96396:23239 93.35234:18267 102.94852:114071 102.97129:1032308 114.06599:19027 118.94382:24266 120.9811:184132 125.965:203425 125.98628:650776 126.95629:38333 131.97206:20813 140.35144:18758 140.97484:74089 143.96651:116040 143.99747:602471 144.96613:27906 162.00861:82644 166.95567:28399 167.01297:104151 167.08054:31199 168.0784:25064 168.10321:27126	
Melibiose	1.251	377.08508	[M+Cl]-	377.0856	57.19011:1304 59.01195:6441 69.55888:2263 69.56219:5181 71.01241:3435 87.00721:1682 89.02299:17536 92.92683:1676 96.95827:2412 101.02248:5849 113.02229:6111 119.03315:6942 147.48834:1603 152.36441:1468 152.89378:1391 157.07291:1431 160.83989:2065 161.04517:6286 164.19987:1514 165.65024:1431 168.88777:2320 169.68579:1398 172.86606:1539 175.83986:1475 179.05408:15632 186.854:1762 215.0313:11772 240.88115:1414 248.03242:1328 260.87216:12411 306.19968:1421 311.68964:1914 318.83484:7368 318.88342:1940 341.10907:62704 342.11551:5720 377.08554:61714 378.08752:2308	-1.378997236
Glycyrrhizin	10.041	821.39429	[M-H]-	821.39655	57.03292:7177 59.0124:45001 69.09844:5120 71.01247:64342 72.99168:52864 73.02773:10655 75.00734:56712 83.01215:6582 85.02797:71453 87.00714:17054 89.0228:17664 95.0123:16229 96.84157:3085 99.00682:30115 101.02275:13097 103.00213:31335 113.02332:176085 115.00244:14687 117.01801:12585 129.01851:8367 131.03368:18739 133.01323:7848 139.00182:2285 157.01317:11459 163.02286:3671 175.0239:51135 193.03484:113579 194.0363:2318 235.04654:7920 253.21765:2587 261.06155:2705 289.05597:14836 307.0654:3248 333.04562:4084 351.05435:259841 352.06055:14479 469.33017:2347 583.37201:2500 627.35382:4272 645.3631:8702	-2.751411605

					695.39801:2298 753.46289:3035 759.4021:3331 821.38934:653085 822.39484:171463	
Medicagenic acid base -H2O + O-HexA	10.87	661.35901	[M-H2O-H]-	661.35931	57.03251:8310 59.01151:30832 69.10388:8056 71.01247:27278 72.99104:4327 73.02712:3213 75.00728:14712 78.95698:7268 85.02788:105737 85.06426:13257 87.00643:8342 89.02208:6446 95.01224:36824 99.00747:25400 103.00172:18167 113.02298:267412 114.02608:4088 115.00118:8956 117.01692:9634 129.01711:6460 152.99422:3207 157.01129:7732 168.04164:7618 175.02293:161687 193.03305:22526 355.26593:38696 423.33145:3723 441.33722:50923 442.34586:6519 485.33078:1419824 486.32669:212048 568.18634:3236 570.21033:25695 571.17828:7279 603.15887:9423 661.3595:210158 661.53693:6444 662.36206:38644	-0.453611215
7-Methylnonanoylcarnitine	9.641	316.2485	[M+H]+	316.24878	57.03445:4846 57.07082:10440 60.08162:189420 61.08515:4662 69.48907:4914 71.08603:17509 81.07029:11666 85.02901:1620691 85.10194:10842 86.03265:53422 95.08614:19423 144.10243:37937 155.14322:73537 156.14702:5124 173.15472:4083 211.17043:7770 256.11664:3256 257.17328:172395 258.17755:23219 299.13812:32440 316.24774:875655 317.25293:151376	-0.88537891
D-Lysine	1.077	147.11292	[M+NH4]+	147.11336	56.05021:12935 69.29392:23546 69.2972:24054 76.52576:10949 84.08159:1422878 85.08494:82460 95.18208:9930 107.15895:13370 120.55824:9036 129.10228:15795 130.02963:15129 130.08603:339994 131.08934:16205 147.05605:20099 147.11186:18188 148.04115:69816 148.07611:13581 148.89301:10862	-2.990890834
Propionylcarnitine	5.391	218.13881	[M+H]+	218.13924	57.03445:7305 60.08166:174301 70.11963:6358 73.02942:11741 85.02905:1364282 86.03288:41546 87.04517:6984 101.05992:13822 111.04474:6673 114.05558:3937 121.10114:4503 129.05496:13204	-1.971218017

					144.10263:40147 147.06447:6232 159.065:261243 160.06851:16153 200.12729:12965 203.1442:55275 218.14008:626420 219.14413:48867 219.17351:418856	
9,10-Dihydroxystearic acid	12.175	315.25378	[M-H]-	315.25409	68.63155:10760 96.83976:2935 127.11072:9349 141.12733:8891 143.10695:5087 155.10684:2443 171.10127:7605 171.13737:5454 201.11229:8845 279.2327:9082 297.24344:79503 298.24741:6273 313.23993:32842 315.25293:1083664 316.25861:128624	-0.983333793
Racivir	7.711	246.03809	[M-H]-	246.0354	55.52898:1427 63.21805:1576 68.48933:10959 72.33952:1377 73.74441:1565 81.48482:1327 83.02291:2282 96.84067:2479 102.03347:2137 103.04187:1329 104.92642:1525 116.92693:3094 118.94205:1788 134.05978:202586 135.0639:16424 162.83745:1654 162.93208:2247 163.69218:1553 167.03384:9765 167.07071:2215 178.04988:955193 179.05257:67273 192.0507:9811 200.92038:1621 201.99159:2142 203.04944:2949 218.02864:25195 219.03069:7267 220.04573:14417 221.04596:1436 246.02551:15452 247.04248:2705	10.93338601
(R)-2-Hydroxycaprylic acid	10.003	159.10107	[M-H]-	159.10211	59.01237:14001 65.01309:4528 69.86913:12604 86.99238:39050 90.00764:3917 91.02863:4440 96.84246:4379 97.0642:40769 102.98734:320931 103.98753:26361 106.03918:13111 111.07957:12174 113.09525:1159709 114.09882:71458 114.98708:23756 115.07491:12244 118.03924:5643 119.0479:4223 130.98236:1104246 131.98315:117127 132.05522:17377 133.0394:10154 133.05104:16255 141.09091:34136 157.08614:22685 158.9778:70592 159.02899:15232 159.06554:71848 159.10127:1469559 159.97855:6711 160.03998:4758 160.0515:17784 160.10712:70632	-6.536682637
Citrulline	1.319	198.08527	[M+Na]+	198.08492	69.5391:64724 80.94861:343656 82.94581:87047 92.04794:71419 137.06856:168367 138.05249:3238807 139.0563:72940 153.06409:4095166	1.766918956

					154.06758:176442 155.07922:1301813 164.03197:71476 174.96019:44595 180.07393:77010 181.05872:2323626 182.06125:59712 198.08624:7562861 199.08965:334404	
Niranthin	11.674	415.21252	[M-H2O+H]+	415.211	51.02411:5726 53.03981:5047 65.03905:4613 69.03419:14520 69.07065:3898 76.03981:46337 77.03936:9858 78.04702:3735 79.05457:12276 81.03458:17043 81.07024:15559 83.04973:9564 85.06541:4027 87.04434:3926 89.06052:50151 91.05502:113193 93.07061:14240 95.08617:19758 103.05451:14642 104.06299:15518 105.06999:4041 107.08617:34908 109.02828:4004 109.10174:10701 115.05554:4624 117.0705:33992 119.08535:8743274 120.0887:548970 121.10117:20276 127.03898:5051 129.05487:9979 131.08638:3873 133.06578:126095 133.08572:31535 133.10214:11256 134.06859:4646 135.08139:48315 135.11697:18618 145.10184:3181 147.11681:8628 149.13184:19611 158.08188:12030 159.11806:4549 161.13197:9471 163.14877:5228 173.133:4736 177.12878:8985 184.07433:5597 187.14883:3892 189.16335:9873 191.14287:4898 201.16492:3701 203.17973:3420 213.16333:5288 215.17882:3184 227.1779:4092 229.19502:4861 245.19008:3931 252.16275:3845 323.2746:33831 341.28094:43313 369.1214:3981 415.21719:67020 415.30737:12482 416.21591:13129 416.31189:80195	3.660789334
Indole-3-acetaldehyde	6.7	160.07594	[M+H]+	160.07568	55.93529:20167 56.9357:12878 56.94304:4310 58.06624:11548 69.07068:17717 69.14751:11238 70.06563:6721 71.04984:6609 72.08163:5151 72.93765:19288 84.08144:6202 86.06077:5455 86.09712:13299 87.04439:6706 88.07632:3596 90.94809:13108 91.05512:5272 96.0814:3994 98.09731:4676 99.04495:5785 100.07661:9906 102.05579:12306 105.07004:6590 111.05581:3668 112.05931:17448	1.624231738

					113.96428:100544 114.05556:6241 114.09135:79469 114.12831:22192 115.05443:18349 117.05472:17353 117.05712:53488 117.0703:4788 118.06535:1357036 119.06944:84563 119.95784:5743 124.05871:24086 125.00239:4761 128.95071:6743 129.95227:13756 130.06461:43069 131.06937:64621 131.97498:47642 132.04393:17417 132.08136:677344 133.08481:441419 134.08778:11804 135.94615:4138 138.03412:38552 141.95947:4750 142.06668:46710 142.08585:28863 143.06822:56706 147.96332:11068 153.95515:3998 154.9894:12329 159.97057:6122 160.07616:1281888 160.09537:993659 160.13269:31579 161.0506:10194 161.07968:160448 161.09714:36974 161.13205:3355	
Tenuazonic acid	9.18	196.09669	[M-H]-	196.09792	62.32889:1396 66.03338:120547 67.0364:1354 70.17924:4935 83.02361:5084 94.0274:1664 96.04298:1321 106.03917:1885 108.08055:17925 128.03337:10428 129.05463:3073 150.09094:185418 151.09534:10547 152.07065:20199 152.89195:3016 152.9045:4621 152.95293:7077 153.09117:2530 169.05022:2047 178.08588:6850 182.02002:2381 194.08241:3142 196.05797:13808 196.09564:320574 196.94481:1427 197.08119:4621 197.09956:23990	-6.272376576
5-butyl-3-methyl-7H-furo[3,2-g]chromen-7-one	11.947	257.11502	[M+H]+	257.11719	55.05516:4888 57.03445:3047 57.07084:9499 67.05525:2733 69.0707:8268 69.13226:5634 70.06565:2549 71.08624:3334 81.071:3163 83.08642:5320 84.08147:5610 85.10194:2847 86.09715:1503 88.07635:1613 93.03748:6737 95.08614:6005 97.1015:2631 98.09734:1533 104.10739:15326 109.10183:2939 117.07035:1888 119.08543:2110 123.11666:1387 131.08649:1610 135.08144:1632 145.1003:2594 150.13618:1345 159.70613:1582 161.13405:1373 173.13098:1424 183.11659:2668 187.07617:1514 211.11214:15733 212.11365:2267 215.07155:10635 229.12283:2357 234.45052:1385 239.10764:30001 240.09671:2753	-8.439731315

					242.0956:1435 257.11478:487042 257.26703:76276 258.11902:68829 258.24466:1823 258.27222:2797	
Vanilloylglycine	6.899	224.05594	[M-H]-	224.05644	70.92112:1368 74.02302:13220 82.47604:1287 88.0017:1457 89.02289:3031 91.01711:7473 99.92404:2222 100.00202:231490 101.00515:6089 108.02011:17604 123.04391:100872 123.90035:15469 124.04654:3277 125.89902:13488 126.27354:1458 139.89519:1922 139.91559:1782 144.90822:7554 148.0386:2180 157.01132:1434 164.0345:20895 165.04135:34587 166.04526:1550 180.06563:43417 181.06888:2434 204.5367:1389 223.79567:11533 224.0562:74774 224.80241:1736 225.05836:7289	-2.231580579
Monooctyl phthalate	12.27	277.14401	[M-H]-	277.14453	60.99129:5487 70.00613:3148 106.03938:31807 107.04895:16649 121.02859:126126 122.03117:6196 127.11069:167040 128.11414:10801 129.12695:1814 134.03604:385599 135.03954:18089 147.00719:19890 151.03882:6350 165.01907:8771 173.11632:7428 190.99805:15533 205.15935:25141 231.13812:1780 233.15407:41188 234.15697:6025 249.14755:6794 277.00671:6979 277.14227:229102 277.1817:18280 278.14716:23818	-1.876277334
alpha-(4-Fluorophenyl)-4-(5-fluoro-2-pyrimidinyl)-1-piperazine butanol	7.033	349.18369	[M+Na]+	349.18344	50.92508:3429 69.67072:6375 76.47814:2892 83.14102:3080 106.17679:2925 143.34106:3023 148.40472:2992 160.07619:4479 349.18423:4495494 350.18872:554344	0.71595606
4-(Decan-4-yl)benzenesulfonic acid	6.904	297.15442	[M+H]+	297.15302	59.05012:143922 69.31524:8933 73.06553:14513 77.06054:57527 87.04445:693233 88.04811:17138 89.06055:1618571 90.0638:56723 103.03973:2156068 103.07535:146605 104.04293:65890 107.07052:70907	4.711377323

					119.07095:29478 121.04932:18775 121.08617:39819 130.08572:47928 131.07083:437377 132.07417:22781 133.08627:634580 134.08968:36660 136.062:24315 147.06441:136600 147.10167:7365 151.09685:14790 163.09755:106299 165.11328:57067 175.09637:189944 176.10045:8424 177.11327:183235 178.11674:18622 191.09009:38661 193.10802:17788 195.12389:26894 207.12338:33804 235.11578:12772 239.15031:16910 251.14796:51451 269.15897:57872 279.14441:14751 297.15619:56779	
LPC(20:4)	12.091	544.34076	[2M+H]+	544.34033	58.06624:293962 60.0816:2207742 67.05524:142686 71.07371:333489 74.02075:88434 79.05529:128463 81.07098:185812 86.09707:7454935 87.10065:122439 91.05513:298591 93.07066:126387 96.84245:171733 104.10733:34792376 105.11077:916079 119.0854:120687 124.99965:3095424 147.1169:88724 181.02644:146631 184.07426:42582840 185.07802:1089863 199.03888:81789 225.95543:103854 258.11084:514345 379.79294:101173 485.2554:140313 526.32776:2941274 527.33691:322658 544.34424:10849694 545.34625:2005276	0.789946981
Theobromine	1.284	219.02681	[M+CH3OH+H]+	219.02786	52.04974:11490 62.98244:254937 68.13892:11290 69.3147:41903 69.31853:18194 78.94209:10652 80.94844:72712 82.94581:2435837 86.99361:141507 90.97691:735494 115.87935:10306 159.05151:1273599 160.05315:51156 177.06183:795402 178.06494:20348 197.95676:10591 201.07309:14145 202.05542:80223 219.08415:2739812 219.17336:20058 220.08786:150993	-4.793910692
Lauryldiethanolamine	9.89	274.27435	[M+H]+	274.27408	57.07092:302500 58.0658:14387 62.06065:35158 70.06572:416687 71.08631:207375 74.05819:12318 74.06181:23976 85.10193:85774 86.06074:14150 88.07639:1199813 89.07961:28629 96.84965:19514 102.09203:338315 103.09521:11414 106.08664:1153315 107.09052:32906 127.07568:15319 137.05934:27901 212.23662:34343 225.50504:12480	0.984416756

					230.24809:120469 231.25241:13185 256.26434:1081434 257.26743:151074 274.27264:20783166 275.27908:3099136	
Kaempferol 3-Rhamnoside	9	433.11307	[M-H]-	433.11288	57.03291:8149 59.01247:58738 70.47571:3137 71.01247:34311 72.99162:9862 73.02772:3828 75.00742:43839 78.95767:32529 83.01215:2307 85.028:155938 87.00726:18502 89.02295:14547 93.03312:2599 95.01233:46882 99.00769:56247 103.00218:35570 107.04884:2647 111.00744:2084 113.02337:433414 114.02608:9768 115.00224:16713 117.01804:30129 129.01845:22471 139.00337:3485 150.03162:2938 151.03865:299188 152.04202:11887 152.99411:139928 157.01315:10440 163.03864:3650 171.00578:2128 175.02409:171098 176.02797:5573 179.88231:10356 187.00621:35214 206.97475:2180 257.08249:236996 258.08649:19035 273.05319:8632 299.09433:7205 352.97842:2986 371.11362:22515 415.10153:3282 433.11292:172351 433.24103:20111 434.11472:20773	0.438684714
LPE(P-16:0)	12.558	436.28241	[M-H]-	436.28281	69.33492:8066 78.95767:400886 121.99946:15658 134.9845:6970 140.00984:319297 152.99408:76360 153.99704:6877 166.02695:7014 196.03569:505908 197.0396:13181 237.22151:9572 239.23718:335242 240.24046:31105 277.15549:6767 375.23111:73001 376.2326:4163 393.24063:7778 436.28586:1456352 437.29047:170912	-0.91683649
Indane	11.675	119.08557	[M+H]+	119.08607	53.03948:3182 56.05034:10059 65.0396:3189 72.08166:1753 73.0851:8654 74.06076:17116 79.05472:18713 91.05518:196833 92.05045:7132 92.058:8864 93.07067:15601 95.0502:10704 96.04482:7995 96.05285:3276 102.05582:2795 103.05462:37789 104.06325:26210 105.04556:2679 109.06509:2482 115.05446:6950 117.07037:49021 118.07388:2697 119.06078:18337 119.08545:425696 120.04515:7892 120.05637:30732 120.08133:48013 120.08882:34504	-4.198643888

Glutamine	1.269	147.07646	[M+H]+	147.07639	56.0502:35452 71.02828:7570 84.04496:2602059 84.08154:200003 85.02902:12114 85.04832:99250 85.08482:8200 88.04029:13131 101.07156:155972 102.05584:153508 130.05077:1655539 130.0858:54035 131.05399:94662 134.68622:5363 147.0762:74167 148.00433:11671 148.04021:11100 148.0607:8804	0.475943148
Glutaminyl-Isoleucyl-Lysine	7.254	388.25476	[M+NH4]+	388.25549	59.05004:15686 68.02541:95411 73.06547:122887 87.04512:393275 89.06047:11517534 90.06354:302119 91.07552:71729 107.07051:165739 117.09192:36312 130.08722:230605 131.07074:182514 133.08612:7295258 134.0891:337305 135.10222:32570 151.0968:44493 175.09619:72143 177.1131:2230816 178.11618:132256 195.12376:79092 221.13753:324261 222.14198:32903 239.14978:152057 265.16599:95299 283.17712:307787 284.17874:17977 309.19388:27321 327.20221:280797 328.20422:34533 353.2164:43766 371.22876:2849020 372.22803:435933 388.25714:40016	-1.880205223
Indole-3-acrylic acid	6.696	170.05981	[M+H-H2O]+	170.06004	55.05515:8023 55.93535:24321 69.34919:23803 70.06619:10291 70.95849:27050 72.04512:96403 83.0864:24854 84.0449:28133 85.06545:29300 86.06086:66512 88.953:19439 88.96888:48534 89.07167:134220 97.00807:31823 98.06047:12274 100.07663:6498 105.93542:6875 107.08531:7612 111.98511:20336 112.0762:8096 113.96436:9834 114.97138:29316 115.05441:260943 116.05819:12555 120.02392:10744 124.07579:59390 126.05482:8401 126.09106:9776 128.95074:467678 128.97156:9206 129.06607:7693 130.0085:25281 130.05069:9003 142.06544:646165 142.0863:27169 143.06853:37912 144.08122:8303 146.96136:409203 146.98161:36571 148.01797:67426 152.07034:31021 160.07625:38150 161.06032:7265 169.97763:90629 170.0611:4486852 170.11742:127666 171.06328:338875 171.09009:21483 171.11343:9282 171.14951:249722	-1.352463518

5-Bromo-6-methyluracil	1.104	204.95718	[M+H]+	204.961	62.98244:8216 68.05199:49944 73.02943:4761 90.97693:1815965 91.98014:14502 96.64124:4181 205.06902:18767 205.09409:63285 206.08096:7511	-18.63769205
Betaine	1.317	118.08674	[M+H]+	118.08628	58.06583:14031314 59.07375:18614354 60.07719:265890 69.79829:231179 72.05669:230033 72.08163:310244 76.03982:290680 101.03486:109371 103.10419:66536 118.08604:92037464 119.09053:2594941	3.89545678
Eudesmin	6.696	409.18665	[2M+H]+	409.18918	69.64096:14493 69.64482:12878 144.08125:7644 146.06009:99819 159.09155:24244 188.07048:619350 189.07449:47509 205.09715:213656 369.75479:7244	-6.182959188
Acetanilide	7.712	134.05956	[M-H]-	134.06113	65.0131:6373 65.99712:6983 71.00787:2441 92.02364:21217 107.02364:9382 107.03426:48631 134.02434:7786 134.04051:27700 134.04494:290385 134.05943:24727 134.86418:827585 135.01874:6182 135.02916:7061 135.04256:11465	-11.71107539
Prostaglandin F2a	10.354	353.2327	[M-H]-	353.23334	51.52026:1620 61.98636:1642 64.54285:1264 65.99712:1717 69.09898:6386 78.95765:1568 83.04876:9851 85.06444:1513 96.95827:2318 99.08057:2309 99.92403:3056 100.93105:1511 102.94669:1448 103.89232:1569 105.06928:8285 106.03919:5633 107.03519:2121 111.04406:3164 115.919:1817 116.92693:5224 120.05711:2664 122.03503:2072 123.07993:8105 129.05457:151964 130.05745:1993 133.04961:1664 134.03464:1412 142.48064:1387 146.93776:2338 150.02988:1517 152.99423:1357 155.04457:1697 173.13367:1864 187.14761:12501 189.16447:2393 199.06148:1427 205.15944:33545 212.93517:1543 217.12172:2638 219.17526:1453 226.0769:1557 235.13161:18614 236.13373:1484 253.14433:16218 264.16949:1698 273.22449:2803 299.2023:6908 309.20978:1713 317.20877:15435 318.21478:2184 326.12827:1545 335.22375:32504 353.14203:6787 353.23642:273216	-1.811833504

					354.13229:1816 354.24051:29448	
Hippuric acid	7.711	178.04971	[M-H]-	178.05092	56.01243:27535 69.29054:36045 90.99636:140181 102.03339:16081 121.02854:41436 132.04396:67710 134.05954:3940433 134.9859:542635 135.06339:235935 148.03847:14735 150.9525:17121 160.03795:27601 178.04971:3293351 178.97726:143434 179.05249:222071	-6.795808749
Oleamide	10.617	282.27969	[M+NH4]+	282.27914	55.05519:91550 56.05028:240249 57.03444:18431 57.07091:110142 58.02982:49172 60.04547:48833 67.0553:147256 68.05044:17533 68.05843:6281 69.07076:178583 69.11481:22640 70.06582:28476 71.04984:21629 71.08624:86274 72.04512:30310 74.06063:8169 79.05465:82405 81.07037:157438 82.06588:71317 83.04985:44356 83.08646:155576 84.08144:6977 84.08949:6343 85.10198:47976 86.06088:40828 87.0444:5647 89.06059:298468 91.05513:6834 93.07084:100805 95.08625:168471 96.08141:38021 97.0652:28235 97.10165:79283 100.07674:37578 107.08637:64042 109.1018:85625 110.0975:12386 111.11681:28261 121.10119:73463 123.11662:21627 130.08585:6430 133.0863:125365 135.11729:50799 137.13248:7536 149.13275:19394 177.11313:24917 211.20532:16283 239.14679:6109 252.26923:411118 253.27158:58653 260.63043:16743 263.13446:7135 264.2677:315959 265.27597:40099 272.1398:19340 282.28012:3471469 283.17285:17678 283.28162:580232	1.948425945
2,2-Dimethylsuccinic acid	6.745	145.04916	[M-H]-	145.05008	64.47848:1376 69.85087:2744 69.85419:1784 75.63348:1161 81.03275:10207 83.04881:99409 84.05192:5806 95.87184:1208 101.05917:103198 102.03348:1898 102.06281:4777 106.64348:1510 115.91901:1397 118.02831:1820 118.03926:3180 119.0233:1661 127.03872:5181 137.27415:1271 145.04893:57618 146.02307:7160 146.03313:3196 146.05153:1716	-6.342636971

5-Hydroxyhexadecanoic acid	12.308	271.2272	[M-H]-	271.22733	59.01194:1938 70.36754:2665 72.83279:1310 82.03946:1631 99.92403:1667 120.65445:1314 127.40618:1269 175.22446:1439 225.22153:20993 226.22528:3269 226.26079:1629 231.56538:1445 253.21667:10405 269.2128:2280 271.22659:1006268 272.229:123867	-0.479302731
Glycyrrhetinic acid	12.863	471.34698	[M+H]+	471.34689	69.07068:8137 81.07032:22418 83.04977:7684 83.08639:7958 93.07079:18146 95.04919:4872 95.08626:70269 97.06518:3929 97.10146:3959 105.07106:7442 107.08639:42535 109.10188:34890 119.08561:35164 121.10126:55671 123.11657:24656 125.09522:3302 133.10088:27268 135.0799:13125 135.11719:89928 137.13246:10225 139.11227:13307 145.1019:10342 147.11703:20361 149.09657:42486 149.13274:21068 151.11092:3126 153.12692:9688 157.10066:4382 159.11591:13872 161.09712:3764 161.13187:28248 163.14955:16762 171.11766:3985 173.13335:25482 175.14954:50517 176.15141:7326 177.16479:40852 179.14244:8396 184.0744:11428 187.14893:11755 189.16364:132150 190.16673:7846 191.18073:29373 193.15636:3395 197.13249:4186 199.14857:18287 201.16231:9252 207.17397:15228 213.16342:11288 215.14299:9559 215.17891:4255 217.15952:62358 218.16129:4606 221.15305:10755 227.18126:4852 229.15892:3518 229.19513:4187 231.1758:8295 235.17041:116910 236.17241:11623 241.19417:4961 243.21048:3310 245.15378:12563 245.19017:17179 253.19519:11995 255.20937:3582 257.18896:4850 259.20761:4185 261.15039:13279 263.16312:43479 267.21329:7772 271.20544:37298 273.18167:4225 277.18201:7548 285.22211:7682 289.2153:3992 291.19394:8815 299.20221:11401 303.198:3943 313.21341:3549 317.21011:124030 318.21536:18346 329.21033:9213 331.22498:7877 389.3187:12065 407.33374:42015 408.33289:9248 425.34341:50694	0.190942174

					426.34311:7852 435.32437:10868 453.33273:25031 454.34299:4296 471.34814:1949834 472.34946:390248	
Skimmin	0.904	323.07855	[M-H]-	323.07721	59.01236:1716 61.00846:2772 71.01239:2148 75.00729:1876 78.95763:3185 85.00873:11002 87.00729:16052 94.92359:9400 99.00685:24514 101.0032:2650 103.01904:1565 113.02226:7246 115.01989:6168 116.0161:9341 116.03386:17370 120.01218:1594 124.00589:6795 135.02777:45838 138.91945:3289 143.0144:2722 145.03069:2688 145.0605:2424 146.0448:6802 146.86513:2456 148.86198:1912 154.92741:3105 157.02994:2907 161.02577:47218 186.04024:5453 187.04063:3027 203.03586:18973 204.00223:5251 233.0462:44031 263.05692:21571 287.98776:2333 323.07758:321906 324.08109:20547	4.147615364
Xanthosine	6.235	283.06842	[M-H]-	283.06842	65.9971:2925 68.74028:7638 68.74406:3714 96.06621:2190 96.83974:3236 99.92402:3055 108.01861:24611 151.02477:3083012 152.02899:102272 183.91191:3726 193.03526:7259 283.06876:901964 284.06979:76709	0
Acetyl hexamethyl indan	11.918	245.18976	[M+H]+	245.18999	55.05515:8238 57.07088:36439 67.05524:7936 69.07084:15840 69.43636:6597 71.08634:12246 79.05478:12432 81.07032:10800 83.08641:5888 85.06546:8380 85.10207:25846 91.05515:43080 93.07076:13273 95.0862:13667 97.06536:10979 97.10149:3565 99.08055:10401 105.07014:83991 106.07414:2574 107.08637:7716 109.10181:3136 117.07039:58626 119.08549:29761 121.10114:9067 129.07051:20046 131.08513:51488 132.08858:6881 133.1008:37716 134.10402:3068 135.11716:8934 143.08551:19989 145.10199:28715 147.07985:53708 147.11703:45492 149.0965:6222 149.13277:3037 157.10072:37078 159.11627:9542 161.09532:34701 161.1322:143845 162.13605:11732 163.11331:3144 171.11775:36561 172.11987:2592 173.13312:5675 175.11195:33223 175.14909:21521 177.12666:2722	-0.938048083

					185.13313:17901 189.12646:6735 189.16348:8069 199.14815:3393 201.16512:79861 202.16678:12926 203.17987:6862 217.19653:17316 227.17819:72105 228.18097:9914 245.10179:11141 245.19029:654432 246.19456:87270 246.24231:6787	
3,4-dicatecholspermidine	6.27	418.19357	[M+H-H2O]+	418.19727	55.05517:58947 68.0451:59344 70.06618:9337 72.08164:1732271 73.08502:41868 76.03975:32852 84.04477:180227 87.0559:153573 96.04477:9666 102.05579:208881 103.05852:7710 113.07194:53972 115.05082:263876 118.08721:1379666 119.09042:51277 125.07133:10649 127.04987:44886 129.1022:658698 130.10553:27368 133.06145:142995 141.06526:146514 144.07626:25596 153.0657:34881 157.09706:92088 159.07639:75811 169.06013:991273 169.09766:20549 170.06525:54970 172.0726:102328 175.1073:1027823 176.11156:51158 180.07614:21453 181.06094:239295 182.06584:9907 186.12349:100461 187.07103:154773 187.10797:59604 190.08218:17530 198.08618:754921 199.08945:41688 200.06816:20044 210.08643:65038 214.12004:88243 226.08099:328827 227.08708:18080 232.12885:503972 233.13145:36954 237.09767:64264 238.08379:473079 239.08363:28813 242.10977:19372 243.14537:65613 244.09409:164333 245.09552:9603 255.10864:544376 256.11267:22529 265.09247:28722 271.14163:96344 273.1217:8715 280.12891:7445 283.10486:685615 284.10657:51345 289.1499:132822 299.13837:26356 301.11548:239561 302.11435:14907 319.12619:29785 336.16785:53199 354.17529:642476 355.17993:60172 372.18756:70530 382.16962:243544 383.17596:32983 400.18491:242443 401.18924:25821 418.19632:278257 419.19614:35627	-8.847499172
Sphingosine	10.614	300.28946	[M+H]+	300.28967	55.05529:32922 56.05027:75324 57.07098:37549 58.02977:9064 60.04498:8820 67.05486:35782 67.60279:8596 67.60648:68502	-0.699324755

					69.0707:56342 71.08633:30846 72.0451:5753 79.05463:23576 81.07046:47892 82.0659:23884 83.08663:45646 85.10194:15891 86.06079:16709 89.06092:26331 93.07082:27868 95.08619:51595 96.08053:6556 96.84248:7609 97.06521:15575 97.10166:32024 100.0757:9165 107.08533:19129 109.10182:21102 111.11692:8744 121.10115:26496 126.02129:5766 133.08623:7991 135.11569:14370 211.20537:19068 252.26913:144550 253.27171:21276 264.26779:102421 265.2728:14646 280.10681:6752 282.28015:1090808 283.17743:15462 283.28149:199247 300.2912:36811 301.07132:8537	
Histidine	1.256	156.07689	[M+H]+	156.07678	68.05044:7643 70.06625:40336 71.02429:7598 82.05297:56580 83.0612:165635 93.04507:101466 95.06062:453528 96.06443:9336 110.0723:6895125 111.05582:30497 111.07468:254594 112.08745:27089 138.06627:11627 139.05006:7224 156.07668:1576289 157.0484:10048 157.08102:63735	0.704781326
N-Lauroylglycine	12.096	256.19156	[M-H]-	256.19128	57.34548:1227 61.85373:1370 66.75309:1202 70.57233:1718 72.04399:1391 74.02332:300564 75.02641:2706 84.04388:1555 93.74184:1241 103.84313:1455 116.92694:3372 155.94016:1314 212.20026:16525 213.20361:1436 239.1671:1938 256.19034:212969 257.17029:26741	1.092933374
1-Methylhydantoin	3.427	115.05046	[M+H]+	115.05018	55.01875:43773 55.05515:30241 57.93555:15373 59.0501:78742 69.03421:74604 69.0707:30419 69.9562:30919 70.06621:732582 71.04983:51486 72.0451:33354 73.93085:15162 86.9936:13236 87.04453:95268 87.05597:1505652 88.04029:17670 88.0591:17978 92.94903:8866 97.06611:9603 97.077:15675 98.06044:72759 105.00371:46803 115.03924:49021 115.05096:733302 115.08723:59636 115.09544:76916 116.05346:17067 116.07121:180930 116.10683:31654	2.43372066
Dehydromenth	10.643	165.09129	[M+H]+	165.09155	55.05515:4559 61.02904:2652 67.0553:47710 69.03423:8521 69.8913:8268	-

ofurolactone					70.06564:2920 79.05529:4444 81.0704:17990 83.01534:2937 83.04978:11066 91.05513:9627 93.07066:9707 95.04919:6736 95.08625:15928 96.04479:3561 100.02538:11910 101.02602:29563 102.0227:22636 105.07005:3011 107.04958:7166 107.08531:3517 108.04495:4097 109.10185:113437 110.10517:6944 115.96456:10333 118.03492:4346 119.03602:15987 119.08546:59164 120.03262:14204 120.08136:208545 121.06575:17771 122.07272:30812 123.04411:3767 124.04169:8942 125.0382:4218 131.04945:6925 133.97449:9141 137.0594:8066 137.09601:335087 138.05551:9496 138.10023:21073 142.96745:110291 143.04852:4033 147.04416:4123 147.08136:3600 148.07605:3409 165.09111:1414447 165.98405:25210 166.05069:13033 166.09679:109734 166.12399:8209	1.574883754
LPE(20:1)	12.539	506.3244	[M-H]-	506.32523	78.95717:442421 96.84058:17072 122.98404:5142 134.98439:10048 152.99435:166852 168.04176:257168 194.05663:4857 224.06888:1186380 225.07155:37809 242.08032:276709 243.08368:4903 281.24805:12079169 282.25272:1126699 417.2431:5357 506.32007:2617102 507.32672:408452	-1.639262574
Korseveriline	11.782	454.32828	[M+H-H2O]+	454.32913	57.03444:17633 57.07082:61179 58.06589:37686 60.08159:290242 69.68064:21278 71.07371:33294 71.08629:65993 75.04473:95674 85.10203:34549 86.09712:725001 87.10064:10729 98.98515:11837 104.10736:6253010 105.1108:185216 124.99975:405010 184.07431:1011096 185.07805:30007 271.2648:7037 436.32538:9353 454.32474:1590742 455.33649:248024	-1.870890383
Benzeneacetonitrile	6.696	118.06545	[M+H]+	118.06567	55.05516:12025 58.06582:61499 59.07379:73067 69.12245:38418 72.04568:9180 72.08168:357795 76.93372:14297 91.05513:423036 92.058:13825 117.05841:52919 117.07035:10374 118.06536:2238259 118.08716:310780 119.06937:136281 119.08507:132703	-1.863369767

Reserpine	7.845	609.30933	[M+Na]+	609.30676	70.06571:20522 72.08173:16265 74.80118:2157 84.04489:2299 84.08144:2303 86.09717:14080 90.05561:2534 115.14812:2065 118.08722:2514 129.10222:2531 160.74477:1956 178.22275:2251 181.06108:2373 194.38684:1942 218.43353:2085 225.95541:2749 408.63043:2120 609.30695:3450258 610.30652:623352	4.217908234
8-Methylquinoline	6.701	144.08099	[M+H]+	144.08078	55.01875:9879 69.30923:27977 69.31306:16765 70.0662:18144 71.0498:57747 81.03431:13031 84.04491:18832 84.08149:712909 85.08482:33484 98.06052:114520 98.09731:168336 99.04496:132365 99.10109:10730 103.05459:13417 115.05446:47377 116.0499:10082 117.05713:14518 117.07036:282631 118.07387:20085 121.9664:60733 127.03908:13422 143.07286:186592 144.04375:40685 144.08124:3118929 144.10182:511720 145.06712:59918 145.08383:271813 145.10527:33009	1.457515708
Pro-CDCA	11.693	512.33411	[M+Na]+	512.33459	57.03444:9062 57.07082:1709 58.06625:9005 59.05006:1831 60.08165:43522 69.07069:2088 71.07372:7104 72.04509:2134 75.04476:5831 81.07098:1804 83.0864:2502 86.09715:144435 87.10065:3398 89.06052:10699 95.08612:1904 98.07153:2013 104.10738:846242 105.11079:24361 124.99975:65224 133.0862:3290 148.17523:1685 157.12309:1823 165.03081:2304 181.02646:2760 184.07439:640371 185.07585:16855 199.03624:3219 204.991:1700 258.1102:16539 329.26706:7438 494.32239:51657 495.32449:8244 512.33289:330700 513.33557:47618	-0.936887748
Indole-3-Lactic Acid	6.696	188.07053	[M+H-H2O]+	188.07001	68.60086:48896 89.03969:23894 91.05441:89446 115.05373:112441 117.05808:57212 117.0702:338145 118.06524:4937352 119.06928:268290 132.08127:113607 142.06525:623698 143.07289:633788 144.04509:206941 144.08113:5265154 145.06529:43742 145.08365:389948 146.05972:26348994 147.06277:1708392 155.0609:55334 160.07597:297690	2.764927805

					170.06094:1882608 171.0645:149462 188.0701:9518505 189.07475:824157	
Lincomycin	9.84	405.2049	[M-H]-	405.20642	51.07285:1406 52.5765:1329 63.00927:1299 71.01069:2033 86.77213:1223 120.70096:1418 125.09616:5112 151.11104:3031 162.91035:1332 166.48033:1567 169.08568:123126 170.08899:7553 177.09154:2342 185.50232:1185 195.10201:75572 196.10605:3113 204.55606:1506 215.06995:1333 227.34924:1498 289.17807:5515 301.77115:1594 307.19296:2596 325.20224:9560 331.57474:1869 339.25867:1459 369.22928:14011 405.13763:3295 405.26913:3133	-3.751174525
4-Hydroxybenzaldehyde	7.718	105.03386	[M+H-H2O]+	105.033	50.0158:8099 51.0239:29646 53.03959:413642 55.01875:21705 69.58642:19676 77.0394:129637 79.05463:8732 81.03428:38237 95.05006:1527981 96.05276:59874 103.05458:9058 105.0343:1055544 105.04524:463063 105.06995:48624 106.03795:48349 106.04928:14571	8.18790285
Indole-3-carboxyaldehyde	6.696	146.06003	[M+H]+	146.06007	50.41438:11829 61.17401:11307 69.85642:14169 69.85975:24986 88.37107:10700 91.05497:149343 100.07665:59673 101.13224:11284 117.05714:20493 118.06535:2838708 119.06944:185911 123.96446:13991 126.04411:16684 135.03679:10959 146.05986:790382 147.06281:68059 147.09155:12360	-0.273859926
Arachidoyl Ethanolamide	11.07	356.3515	[M+H]+	356.35229	55.05531:27006 57.0709:17805 67.05524:4025 69.07086:42113 69.25124:9878 70.06577:52416 71.08621:9539 73.02882:11296 76.03981:3241 81.07027:4087 83.08656:33609 85.10192:2963 88.07645:163295 89.06051:9747 95.08611:9723 97.10163:23231 102.09221:39610 106.08675:130492 109.1018:2876 121.10112:3323 135.11714:6979 136.062:3317 184.07442:3907 247.24211:3267 265.25223:2834 274.27264:2641 283.17285:2790 296.29324:4006 309.90155:2453 312.32782:14334 313.3291:2671 338.34106:139895 339.34741:25943 356.35367:2953709 357.3573:618098	-2.216907319

Naringenin-7-O-Glucuronide	8.704	447.0929	[M-H]-	447.09274	59.0121:12487 70.17759:3416 71.01185:5534 72.99164:1496 75.00731:3362 85.028:29920 87.00722:1908 89.02289:2024 93.03313:1706 93.90649:1370 95.01234:10030 96.95919:1610 99.007:10356 103.00221:3287 107.01211:1383 113.0224:83544 115.00121:1524 117.01814:2978 119.04917:2781 129.01852:2248 129.20636:1555 151.00195:23862 169.66484:1725 174.95421:2594 175.02448:20919 242.4024:1366 258.58694:1735 271.06146:80655 401.80185:1657 429.07251:1541 447.0965:40406 447.20245:25455 448.10168:2803	0.357867587
Mono(2-ethyl-5-hydroxyhexyl) phthalate	10.125	293.13925	[M-H]-	293.13943	71.04872:24658 78.95765:9445 83.04874:1871 96.95822:21768 96.96812:15267 97.02804:6072 99.04317:9367 111.04406:2548 115.0386:327373 116.0422:8275 125.02322:2749 137.05931:2545 137.09584:5801 147.07977:2921 149.06032:2116 150.06648:8619 162.06755:157689 163.07477:27487 165.08951:7216 175.07497:2197 176.08337:7194 177.09154:265424 178.09477:21206 191.06889:2906 203.10709:2936 213.12708:11029 216.11475:56783 217.1187:5943 231.13824:114474 232.14102:13375 245.1167:2979 275.12762:2334 293.14081:12724 293.17874:75952 293.62564:15296 294.14301:13804 294.18106:5087	-0.614042267
2-decyl-3-hydroxypentanedioic acid	13.472	311.18146	[M+2H]2+	311.18292	54.66439:20610 57.07088:291012 61.69002:14963 67.05525:42407 69.0707:43655 71.04021:35037 71.08626:189581 73.02883:32704 78.80988:18356 81.0703:86741 83.08642:38284 84.96065:68222 85.10188:153718 86.09715:80058 89.06053:74339 95.08625:134079 97.10149:69663 104.10769:156996 109.10182:42920 111.11803:25674 123.11794:25718 129.74461:16517 157.01671:18928 157.53674:60661 166.54314:34575 169.40471:17875 178.87219:16580 181.02417:20948 184.07446:102729 185.53265:21042 196.24828:17517 201.89769:17869	-4.691774214

					219.90762:67389 229.89114:23777 237.91698:48824 240.10027:296783 241.1019:17490 247.90253:201165 252.95473:21666 256.00397:75699 256.02341:119519 258.00079:17530 264.91254:17173 265.91299:229402 270.922:18362 271.98239:39979 274.03595:18726 314.03064:260773	
Tryptophan	6.696	205.0972	[M+H]+	205.09718	68.59937:19622 68.60314:9446 74.02437:128564 91.05416:23854 115.05545:37646 117.05772:32484 117.06995:42614 118.06521:682310 119.06925:40399 130.06453:131238 132.08124:631765 133.0845:46303 142.06523:111230 143.07256:97279 144.04532:66795 144.08112:1600218 145.08369:130208 146.05971:9367509 147.06445:666450 159.09138:1253027 160.07596:117445 160.09517:112436 161.07755:11244 170.06094:541881 171.0645:50392 188.07002:15168452 189.07454:1524609 205.09671:197686 206.10027:13370	0.097514749
1,5-Naphthalenediamine	6.696	159.09174	[M+H]+	159.09167	55.93542:653896 69.5864:44322 70.95848:30760 72.93785:102035 73.94591:35786 76.93369:33110 81.04562:58880 86.09712:14370 88.96879:92070 89.94062:41127 90.94796:110310 95.0606:135853 105.9354:28370 107.95113:90515 108.95926:29989 111.05582:426279 111.98509:13729 113.07972:65156 113.96437:123320 114.09138:73286 114.97134:52565 115.05437:63915 117.05838:101749 117.95954:123068 118.06532:737533 123.05569:500514 125.00238:44157 125.96099:91900 128.95067:661224 130.06465:539508 131.06815:55297 131.08217:17000 131.11775:45351 131.97488:48759 132.08127:3289891 133.0847:182382 135.94612:323796 135.97021:43841 141.01929:51366 141.06519:532862 142.06511:436997 143.0696:36769 146.96121:399006 148.9774:14198 153.95682:259327 158.08383:29831 158.96214:49198 159.09142:5969646 160.07614:1242589 160.09535:559580 160.13379:30579 161.07967:31217	0.439997896
3-	10.835	187.13258	[M-H]-	187.13342	59.01234:522809 60.01559:8230 65.01308:2394 65.99709:5277	-

Hydroxycapric acid					69.10169:8750 72.99161:2328 83.02361:1756 89.02286:6583 91.02862:2088 97.06428:3170 99.92397:16774 99.9477:14631 107.04883:2234 115.07489:9321 115.91897:8878 116.92691:6025 118.03922:2487 118.97401:1655 121.05009:2033 123.0799:2967 125.09608:142396 126.09873:6744 133.03938:7077 141.1273:6947 143.1069:2579 143.93695:7229 145.05055:2643 146.03474:2364 160.05:18184 161.0451:5128 169.08566:6223 187.00667:2093 187.06094:12740 187.09622:118337 187.1328:139242 188.05888:2577 188.10048:6561 188.13756:12787	4.488775976
6,8-Dihydroxypurine	6.267	153.04089	[M+Na]+	153.04124	55.01874:4891 55.05514:11289 65.03958:3702 67.05524:4013 68.94347:12284 69.07069:4545 70.06564:11542 70.94261:33751 70.95849:13040 71.94079:51525 71.95238:8450 79.05528:4720 80.04987:9758 81.07097:8671 82.06588:9904 83.04977:10814 84.0449:13938 84.08144:10743 88.95322:16030 88.9912:8410 89.95196:9035 93.03406:8854 93.07074:16433 94.06554:9448 95.05007:4026 97.06522:21660 98.06046:11151 102.02254:39204 107.00168:16552 107.04969:17170 107.06113:12233 107.08606:27374 108.045:33845 108.0556:14056 108.0812:39847 109.02942:8307 109.06506:7518 110.03512:197090 111.04504:31355 111.05582:12454 111.96936:73280 111.98504:18017 112.00646:8316 112.07619:14521 112.96693:51351 118.52659:10806 120.03265:13452 125.0024:5019 125.06074:26139 125.07135:26887 126.05481:36107 126.06689:10092 126.09234:24806 128.04587:59853 129.97911:46079 130.01677:77096 130.9783:25785 135.04419:13088 135.05609:14538 135.08141:4236 135.09183:14452 135.11714:8841 135.94617:10159 136.01382:284017 136.03943:12906 136.07549:38231 137.01833:9210 148.02821:20182	-2.286965265

					153.04071:1841647 153.06465:77374 153.091:16040 153.10178:33722 153.12694:13911 153.95697:9249 153.99321:11544 154.02589:451432 154.04398:42158 154.07298:7629 154.08569:116000 154.12155:24657 155.02869:15620	
5-Heptyl-3-methyl-2-furanpentanoic acid	11.933	279.19565	[M-H]-	279.19601	57.03307:17630 59.0121:170551 69.03254:10359 69.14856:9812 71.01237:12708 83.04871:4400 96.95824:5118 97.06427:2984 99.0799:35199 107.08453:8361 113.09529:79831 121.10061:2747 127.11067:4193 133.10086:27007 135.11702:153195 136.12054:10496 139.11063:4579 140.1151:2633 153.09114:3097 161.0955:8573 163.07414:4580 163.1474:71758 175.14743:3160 177.08926:4070 179.1068:404515 180.10881:24003 181.12196:3150 189.16435:109745 190.16774:4283 217.19452:203780 218.19911:19117 235.20737:35127 236.20937:2651 243.17369:11677 261.18552:298406 262.1893:34084 279.19714:605689 280.12869:9874 280.20026:77806	-1.289416708
LPC(16:0)	12.385	496.33917	[M+Na]+	496.3403	57.07082:4834143 58.06667:4160182 60.0816:25868768 71.07372:3375955 71.08622:3400755 85.10194:1761011 86.09715:83546472 95.08613:1970930 104.1074:429726432 105.11093:5876262 124.99983:29601584 163.01457:3424834 184.07445:524532480 185.07628:6715236 225.90071:1913422 258.11118:10157058 313.27136:17125828 419.25269:3629570 478.32599:38947216 479.33881:4011202 496.3407:168345408 497.33859:19366290	-2.276663813
4-Hydroxybenzoylcholine	13.413	224.12833	[M]+	224.12756	58.06623:115784 60.0816:83886 61.08515:2413 73.02942:4120 77.04005:2542 79.05524:87077 80.05872:2960 84.08146:2349 91.05502:21258 93.07066:258183 94.0742:11687 95.05009:2728 103.05557:3685 119.04958:23634 121.06575:208829 122.06875:17627 137.9648:2267 153.9588:3400 155.97499:29878 156.98311:4462	3.435543581

					159.94565:3025 164.08185:2324 165.05501:482024 166.05907:30245 167.99367:7715 174.9931:3843 177.95642:20067 178.99022:4007 184.07445:3046 200.97339:31725 206.08093:3952 224.12737:1038366 225.13016:105004 225.19714:3863 225.56618:3824	
methyl 3-oxo-2-[(3,4,5-trimethoxyphenyl)methylene]benzo[b]furan-5-carboxylate	13.462	208.03955	[M+2Na]+	208.04182	56.96548:22755 69.66357:81728 84.96049:3368828 94.28312:18480 102.97118:409658 115.96453:227059 116.96111:25824 117.95935:963572 118.9672:21139 120.98118:72097 125.98621:938264 133.97452:41442 135.97006:336891 143.99739:1042098 144.97108:18747 158.98598:149777 162.00778:162702 167.01283:277022 167.9875:65070 168.28111:19332 174.98434:23328 184.9707:32665 185.02319:24145 185.99529:166666 209.01308:44464	-10.91126774
N-Lactoylvaline	7.379	188.09167	[M-H]-	188.09229	69.5022:2915 71.01236:5551 72.00742:2121 73.02767:2016 79.95579:1846 88.03844:2685 91.02858:2561 99.92397:7683 99.94804:12360 100.07504:27938 100.95142:26071 103.91837:2705 104.92635:2448 106.04016:1994 109.02782:26383 115.91892:6238 116.0696:71411 116.92686:3068 116.96284:2289 125.87202:7613 126.09109:13438 126.09868:11171 141.86636:7576 142.08456:4408 142.98184:9779 143.93709:16125 144.10098:116459 144.92134:2872 144.94148:11997 144.95784:56425 145.10518:3495 157.8618:1940 159.8768:6725 160.03798:7422 162.06557:2141 188.03391:18348 188.09064:467012 188.98418:22498 189.09552:32526	-3.296254195
Eumitrin D	6.67	665.18237	[M-H]-	665.1875	67.63223:6001 67.63593:27453 72.00765:51611 74.02328:9066 96.83974:12969 103.91859:56991 104.92641:8962 106.94176:8565 113.92682:4797 115.94266:10096 116.04934:35835 123.94567:4881 127.94252:11747 129.95784:17882 130.06447:11895 131.03638:20445 142.06537:12808 143.93861:9278 144.04349:4742 145.95279:11977	-7.712111247

					152.93858:13661 154.95303:14496 156.06792:16543 157.07661:12938 158.9245:18211 159.09251:16678 160.94051:4954 198.94466:28336 200.9603:32736 203.08223:140902 204.08517:11327 213.01164:205136 214.01192:13226 217.98907:13100 218.96977:24834 220.00488:15793 220.98672:9762 229.00665:9296 231.02156:5089 240.02177:4396 256.99979:25899 259.017:166351 260.02048:9869 264.01886:10391 275.01071:114939 276.01682:4678 284.01028:101890 285.0166:13859 287.03571:29411 290.00067:8550 292.01584:9807 302.0249:53471 329.02411:5550 330.01675:303646 331.0202:22405 332.03314:68745 348.03073:75587 388.07608:26586 403.78754:10430 406.08188:18681 415.08548:11663 417.09796:10272 419.05188:3995 432.06363:12916 433.09613:4033 435.11203:4477 459.07706:5242 460.09561:11737 461.08951:2235110 462.09558:328217 479.10428:3903 665.18365:68189 666.19287:12816	
(1R,2R,4aS,8aS)-2-[(2R)-2-Hydroxybutyl]-1,3-dimethyl-1,2,4a,5,6,7,8,8a-octahydro-1-naphthalenecarboxylic acid	11.92	263.20078	[M-H2O+H]+	263.20001	55.05523:307950 57.07092:145280 59.05005:15730 67.05531:349293 68.05843:14550 69.07076:317184 71.04987:84246 71.0863:118651 73.06552:32854 79.05473:465193 81.0704:622907 82.07363:30583 83.05002:87768 83.08641:144216 85.029:36550 85.06547:88301 85.10192:41810 87.04439:35730 91.05513:373401 92.05797:17366 93.07075:997343 94.07418:39590 95.04919:31005 95.08627:431872 96.08944:16285 97.06518:50702 97.10143:112058 99.04495:20352 99.08142:20341 101.0599:49116 105.07017:658900 106.07412:55518 107.08643:502699 108.08969:18221 109.06519:81290 109.10189:282303 111.08134:15092 113.05938:31786 117.07048:174574 119.08552:809183 120.08876:69987 121.06448:46883 121.10123:469246 122.10455:33003 123.08035:56886 123.11668:143712 127.07599:65874 129.07042:146702	2.925531804

					131.08517:302907 133.06435:32830 133.10088:808376 134.10379:85404 135.08006:64295 135.1172:358851 136.11922:32349 137.09593:45527 137.13248:54947 143.08585:180618 145.10188:127784 147.07979:252621 147.11694:656395 148.08459:15120 148.12039:66808 149.09656:152065 149.13272:302413 150.13614:19601 151.14793:13789 153.091:43180 157.10094:176659 158.10458:17898 159.11624:57391 161.0972:490564 161.13208:567252 162.10051:19806 162.1357:64026 163.11163:59143 163.14905:218534 165.09105:84764 165.12733:13239 167.10712:58096 171.11787:182701 172.11984:14631 173.13309:49682 175.11189:475199 175.14864:94701 176.11595:57552 177.16466:12376 179.10628:359906 180.11055:40428 181.1235:38210 185.13287:67190 189.12663:145197 189.16344:18650 193.12325:425209 194.12494:52235 199.14812:13950 201.16496:133799 203.14413:47065 203.17998:401476 204.18336:33389 207.13728:272014 208.14165:34177 217.15924:126040 217.19505:81260 218.19797:13400 221.15355:101702 227.18123:192825 228.18491:43692 245.19006:403516 245.22665:109746 246.19464:51293 246.23128:18973 263.19928:1716350 263.23569:203381 264.20239:285224	
beta-Ureidoisobutyric acid	1.389	129.0661	[M+H-H2O]+	129.06586	55.05515:36836 56.05024:67089 57.03445:15786 57.07082:7995 58.06583:8039 67.05524:29388 69.59633:17213 69.60018:15984 70.06564:9761 71.04986:28580 72.0451:7276 83.06126:2021380 84.04492:1306561 84.05953:7731 84.06454:75157 84.08144:402521 85.02901:8997 85.06552:103147 86.06079:10050 95.04921:7058 106.74352:8116 112.07624:113927 119.02998:8203 129.01881:40578 129.06606:606508 130.05067:208172 130.0704:13871 130.08592:484629	1.859515754
E-64	7.148	358.20728	[M+NH4]+	358.20847	59.05005:93990 69.74248:21063 73.06554:11845 77.06058:24539 87.04514:593978 88.04811:8374 89.06049:1496836 90.06342:36254	-3.322087833

					103.03967:2244687 103.07553:90371 104.04291:75408 107.07055:65495 119.07036:71784 121.08599:99160 130.08728:25109 131.07074:265016 133.08614:1081246 134.08923:61415 147.06615:273312 163.09741:89493 175.09634:168402 176.10046:11260 177.11311:230785 178.11452:18736 191.09038:120471 207.12332:132828 209.13934:60246 219.12411:32742 221.13737:48197 235.11945:135061 239.15033:29516 253.12648:27024 279.14444:10287 283.17288:23923 295.17496:79834 297.15616:19768 313.18689:71535 323.1705:58104 341.18045:606121 342.18652:94469	
1,2-Dihydroquinoline	6.695	132.08081	[M+NH4]+	132.08078	69.58422:19713 86.0608:8640 86.09727:169403 87.0999:9181 90.05564:10558 91.05515:7316 104.04998:10255 105.0702:77503 115.05441:108508 117.05715:358916 118.06049:28753 130.06503:47229 131.07368:31947 132.04413:45586 132.08139:1479135 133.06584:13507 133.08485:126441	0.227133728
LPE(P-18:1)	12.718	462.29797	[M-H]-	462.29846	50.75413:1338 60.08224:1469 62.76144:1421 63.57374:1326 69.53631:6668 78.95773:140986 96.84245:1713 121.99921:2701 129.5273:1455 138.98781:1395 140.00986:115884 152.99411:12909 159.89989:1366 196.0383:181950 197.03922:1869 236.08551:1566 237.2215:1541 265.25162:86882 266.25449:6236 283.26312:1699 290.94443:1366 372.64545:1398 377.62766:1612 391.50827:1606 394.10706:1517 401.24472:26072 419.25534:1778 462.30307:511415 463.30426:48917	-1.059921333
cis-5-Tetradecenoylcarnitine	10.452	370.29575	[M+H]+	370.29575	55.05514:3433 57.03455:11396 57.07082:6913 60.08162:190153 67.05525:14131 69.07076:19980 71.08678:6055 79.05462:6803 81.07091:17317 83.08637:13156 85.02901:1435437 85.10193:3586 86.03272:39641 87.04517:3900 89.06055:50467 93.07069:15365 95.08602:20291 97.06534:19591 97.10165:11869 103.03969:8246 107.08642:15114 109.10182:26025 111.1169:6519 121.10112:37638	0

					123.11662:6019 125.09657:8230 133.08615:28980 135.11749:27457 136.0621:37679 139.11229:2784 144.10248:41910 149.13275:8117 153.12694:2766 157.05026:7601 177.11099:6058 191.1804:44468 192.1824:6030 209.19081:37662 210.19301:3034 225.37993:3082 255.15913:2800 311.22012:90484 312.22693:15440 370.29468:881977 371.29709:160060	
Di-N-propylamine	13.163	102.12832	[M+H]+	102.12772	56.05024:54682 57.07081:53185 58.06588:443336 59.06123:55066 59.06944:7555 59.0738:277348 60.04542:11434 60.05645:6686 60.06484:11969 60.08162:25582 72.08165:103188 73.08924:7457 74.09697:518213 75.1003:8647 84.04491:12946 84.08147:13169 85.02915:40042 87.0683:7238 102.05582:24604 102.09201:636548 102.1282:2327041 103.09528:32310 103.13184:119553	5.874996524
Tetradecyldiethanolamine	10.436	302.3056	[M+H]+	302.30539	55.05471:3621 57.0708:149965 58.0662:5452 62.0611:17068 70.06612:234359 70.35964:4842 71.08617:107391 72.08971:4206 74.05816:3777 74.06179:4628 85.1018:60772 86.06071:5992 88.07626:652442 89.07958:14413 95.08604:3720 102.09192:172910 103.09518:3801 106.08649:573362 107.09048:14249 240.26935:21715 256.26422:9169 258.28009:60508 259.28268:12218 284.29672:525232 285.2995:81566 302.30365:10132405 303.30804:1856447	0.694661779
Metolachlor	12.247	284.14072	[M+H]+	284.14117	57.07081:8176 65.36414:1960 67.05523:2324 69.07068:2889 71.0862:2571 73.0294:10986 73.06554:128222 74.06909:3270 76.97979:3252 81.07027:2964 81.37923:2065 85.06544:2621 89.06046:41088 90.06371:8284 95.08611:3258 109.10179:3010 119.08539:2715 121.10111:2077 133.0862:18726 134.09769:11763 135.0799:1942 137.13246:1945 146.09644:13399 158.09703:8996 160.11273:12132 174.12906:3031 176.14261:377951 177.14685:43326 184.05336:11866	-1.583719811

					188.1093:1949 194.0732:13013 211.07727:2022 212.08418:71159 213.0867:3387 233.89644:2033 239.15106:17871 240.14955:1972 243.11339:4031 252.11362:3112214 253.11884:420320 284.14249:188301 284.29242:50361 284.32877:9757 285.07596:6793 285.14526:27826 285.181:5682 285.21408:14255 285.29977:3740	
LPC(18:1)	12.541	522.35522	[M+Na]+	522.35596	55.05515:299934 58.06625:530289 60.08164:6452806 67.05472:284091 69.07069:325332 71.07372:426313 81.07098:365026 83.0864:356267 86.09713:19185798 87.10065:412570 95.08612:398174 96.85149:355451 104.10738:95992152 105.11088:3467219 124.9998:7118588 163.01456:281995 184.07439:112710144 185.07852:4108397 225.93611:426583 258.11136:1619362 339.28741:2100208 340.29166:272230 347.06506:260514 370.09207:247660 433.8894:253026 504.34323:9711253 505.34546:2407617 522.35388:39701476 523.36255:10787739	-1.416658479
LPC (O-18:0)	12.227	510.3931	[M+H]+	510.39178	57.03444:13507 57.07084:35832 58.06594:17889 59.07459:3852 60.08164:144830 61.08469:3211 67.5996:19349 67.60329:22954 69.07068:3618 71.07368:19873 71.08627:36614 72.08163:3311 75.04466:33033 85.02901:26335 85.1021:21449 86.09718:357139 87.10064:11297 88.07632:3959 89.0605:4479 95.08611:3119 98.98421:5276 101.07146:3327 104.10741:3007643 105.11081:84824 109.10179:3150 124.99984:186175 184.07439:598183 185.07625:55740 186.07724:26468 228.23067:3258 313.27609:34019 492.33734:4231 510.39191:880640 511.39847:117766	2.586248548
PE(18:1/18:2)	12.313	742.53961	[M+H]+	742.53809	55.05516:131584 57.07082:85955 58.0637:15818 58.06664:76424 59.05003:24081 59.07413:13305 60.08158:301516 67.05518:397437 69.07065:300318 71.07347:81669 71.08621:66222 79.05516:92432	2.047033035

					81.07092:514849 82.07359:11881 83.08633:255423 85.06541:23865 85.10173:44410 86.09707:1442700 87.1006:29039 91.05537:46864 93.07059:153869 95.08607:508129 96.08939:33158 96.8424:14462 97.06513:24008 97.10142:165637 98.98447:44432 99.08044:20627 104.10722:148652 105.07101:29924 107.08621:120668 109.1018:247686 111.08018:24404 111.11648:53804 119.08534:26699 121.10096:157696 123.11785:128120 124.9996:637624 127.11235:11338 133.1019:52042 135.11707:165879 137.13206:47997 146.98152:12664 147.11682:28409 149.13277:68570 151.14786:24861 161.13197:32166 163.14877:42101 165.1635:12871 175.14897:23796 181.02635:12824 184.07411:16273722 185.07794:482230 245.2265:10062 247.24199:36189 263.23517:70526 265.25214:97184 306.27939:96960 308.2934:28700 319.26672:10775 337.2739:54377 339.28778:52779 480.34537:36505 502.32874:95024 601.52124:1928488 602.51593:535246 742.5791:479052 743.57831:166984	
13-Hydroxy-6-(1-hydroxyethyl)-3-(hydroxymethyl)-12,14-dimethyl-15-nonyl-9-propan-2-yl-1-oxa-4,7,10-triazacyclopentadecane-	12.442	522.34979	[M+H]+	522.35303	56.05058:20756 57.07081:69133 58.06666:89888 59.07459:20248 60.08159:386542 69.49291:59833 71.07366:94474 71.08621:48583 81.07028:53798 83.0864:17079 85.10192:20711 86.09711:2128128 87.09988:18765 95.08611:73909 98.98515:21013 104.10737:2542684 105.11092:61947 124.99976:720281 163.01663:131679 181.02644:17202 184.07443:15434511 185.07584:424187 196.93054:21780 211.03368:53593 214.93982:89629 222.08865:18877 239.0451:14652 240.10016:79090 252.95467:62643 256.00372:95572 258.11084:53371 313.27127:76506 339.28772:49155 387.14224:16793 417.1438:26274 419.25266:79719 435.16205:86627 478.32571:917853 479.3288:77920 492.23376:25257 493.23999:49031 494.22864:392966 496.33908:186631 504.34354:111528 512.24445:59248 514.29578:60199 522.35461:388849 523.36273:68632	-6.202701648

2,5,8,11-tetrone						
Psicose	1.293	203.05247	[M+Na]+	203.05263	50.38383:205071 54.43894:179508 56.17778:151460 57.31079:153260 60.50656:172652 66.49635:149233 70.74415:310551 75.99521:156605 107.88008:159235 117.30979:187344 146.31133:175040 199.67967:181681 203.05353:23218056 204.05621:1005931	-0.787973049
Troxilin B3	11.169	353.23276	[M-H]-	353.23279	57.0329:2314 58.00431:1581 59.01253:9510 69.03315:11003 73.0277:1833 81.14057:1499 84.2914:1682 92.66653:1366 96.95825:5149 97.06428:2604 99.92401:2668 116.92691:6632 125.05901:1482 126.98981:1333 134.07288:6370 137.26953:1321 139.11057:30887 147.07974:3141 149.0948:1847 149.11725:1323 151.11057:16555 152.9942:1863 153.09109:53222 153.12711:6337 154.09314:1739 155.10681:1678 161.09552:5448 165.09149:6305 167.1055:6356 169.1232:6901 177.09109:19663 181.08499:3106 183.10071:46172 184.10602:1511 185.11472:2152 195.10178:81783 196.10603:2810 206.09357:7281 213.11205:7136 221.0802:3159 255.21274:5613 273.22443:1514 317.2103:3118 335.22345:16582 353.13565:2936 353.23615:98170 354.23734:12492	-0.084929828
2-Benzimidazole carbamic acid	1.244	178.05896	[M+CH3OH+H]+	178.0611	50.70042:4713 53.87986:5725 62.98252:64334 71.64256:4645 81.02879:4635 87.66933:4806 98.07892:6430 105.04251:5239 117.98753:8268 120.00409:77623 133.03676:138680 134.06871:5245 137.06853:71157 160.04741:8039 160.98285:29243 161.00221:5384 178.0088:40048 178.05824:913647 178.08525:34504 178.12582:18760 179.06296:51899 179.09184:65463	-12.01834651
Isoevernic acid	0.866	217.02899	[M-H]-	217.0273	69.27036:25648 78.95764:25959 79.95584:6060 96.84156:7812 96.92025:29311 96.95827:5872637 97.07881:5723 97.95745:33216 99.40125:5326 127.86822:43559 148.94817:19767 156.90312:7469	7.787038773

					156.91431:33072 158.84656:5678 159.85963:21041 170.88248:94748 198.90706:10307 216.90936:254651 217.02768:9795	
N-palmitoyl-phosphoethanolamine	11.429	378.2403	[M-H]-	378.24094	69.6018:1780 69.61723:7182 78.95761:2015322 96.95824:14429 96.96828:27646 303.94058:1912 311.16827:3640 378.24072:950020 379.24811:97175	-1.692043172
4-Methyl-11-(4-sulfophenyl)tridecanoic acid	7.379	383.19165	[M-H]-	383.18973	57.0329:3297 59.01214:80480 61.02803:51853 69.10005:9427 72.99123:93488 75.00684:32228 75.04364:4863 96.84154:2833 101.02264:163475 105.05412:107892 254.06427:1924 383.19366:928346 384.19882:76137	5.0105727
(2E,4E)-12-hydroxy-13-(hydroxymethyl)-14-methoxy-3,5,7-trimethyl-14-oxotetradeca-2,4-dienoic acid	10.877	303.19238	[M+H]+	303.19501	52.72691:1624 56.96548:7473 57.07091:12876 58.06584:11480 60.04553:17095 69.25509:8171 71.08623:8037 81.0703:2769 83.08642:2468 85.10194:3454 86.09715:7986 88.9204:1655 91.05531:34119 95.08614:8962 104.10757:28147 105.11095:1815 109.10183:2197 111.51457:1665 112.99104:2769 129.52484:4043 130.02681:2329 130.05072:3221 131.00113:6679 131.05234:2141 138.5291:14424 139.03143:3177 140.52507:2105 140.54245:1948 148.06071:7567 148.53125:13074 149.01195:2231 149.54819:10566 150.03853:3372 151.55237:1846 157.53694:63608 158.03896:24159 158.53917:3971 158.55432:8442 159.534:2722 166.5433:32171 167.04417:12714 168.53813:3914 169.04588:23217 169.54701:8434 170.06326:2508 171.04132:1992 178.05142:8544 184.07425:23512 185.07571:4023 210.96687:2918 212.23718:30715 228.97816:3376 240.10023:11205 241.10191:3682 255.29066:2035 267.28796:2398 273.16037:6411 282.16333:45409 282.66293:6501 283.66165:1907 285.29617:24991 291.17093:35554 291.66998:12069 292.17203:2183 293.67453:3973 297.05908:1839 300.17654:2426 302.67822:16736 303.23315:3836 303.27322:3377	-8.67428524

					303.30814:39549 304.2977:157247 305.96582:1917	
Phosphocholine	12.267	184.07352	[M+H]+	184.07327	58.06625:4132 60.08159:214872 71.04985:5220 71.07372:17967<br>74.9382:3536 75.26474:3041 78.37572:2805 86.0971:968721<br>87.10039:28839 91.62558:3041 96.84246:7801 98.98516:20315<br>101.73389:2813 104.10714:22307 106.03684:6574 113.96436:3394<br>114.95605:26405 115.96434:23038 116.97191:35065 117.98022:83418<br>120.96474:4944 124.07577:3742 124.99973:574294 126.00383:4697<br>132.9666:25486 133.97449:6360 138.09244:4185 139.98784:17008<br>140.99545:16398 141.98357:3892 143.01122:7118 147.18462:3030<br>149.07059:4707 155.98236:3535 161.9689:21057 166.02657:5765<br>166.08745:4005 167.08051:6499 184.0743:2388698 184.98497:5886<br>185.07629:80879 185.13062:4282 185.16406:10775	1.358154826
3-Methylene-indolenine	6.699	130.06514	[M+CH3OH+H]+	130.06567	55.01874:15296 55.05514:16962 56.05051:47582 57.03444:6953<br>57.07081:17917 58.02975:13731 58.06624:6748 60.98746:6418<br>67.05518:25154 70.06618:15030 71.04974:43451 71.93039:17023<br>72.93834:7492 78.99794:36689 84.04489:1769699 84.08143:463453<br>85.04313:6250 85.04829:70388 85.06548:112918 85.0848:22862<br>86.09713:5867 88.953:14276 89.94063:24230 91.05486:26534<br>95.05007:8124 97.00806:318637 103.05451:73239 107.95092:28578<br>112.07614:163670 116.06293:7064 120.02387:121798 125.96068:38738<br>128.04997:13662 130.05147:263809 130.06471:1632809 130.08569:533045<br>130.12245:15837 130.96696:13550 131.05373:16660 131.06892:113934<br>131.0909:24858 131.11777:6872	-4.074864643
Primidone	10.324	219.11597	[M+H]+	219.11278	56.05058:5902 58.06582:17824 59.99116:5180 61.0114:14087<br>69.72424:11293 70.06563:4052 72.08163:11635 73.0294:3863<br>76.02227:24980 86.06068:30151 87.04439:4298 88.02225:4379	14.55871264

					88.07632:6854 91.05431:4462 102.03728:19097 104.01701:23028 104.05306:35044 105.07107:5236 111.09245:4577 112.09983:7066 113.07192:5003 116.05344:12023 118.03248:4190 119.0854:4054 121.10112:4428 130.03239:4485 132.04826:176556 133.05125:3939 134.06276:3748 140.13089:12255 141.10185:102451 145.10191:4918 146.02654:25036 159.11624:5795 161.13205:3554 170.14107:11246 173.07451:20178 183.11653:10656 190.07681:26596 201.12985:5405 203.14386:60706 204.1474:4587 205.15871:40565 219.11487:3545809 219.17296:499319 220.11818:337272 220.17668:70384	
Ornithine	0.888	131.0811	[M-H]-	131.08263	69.26107:39682 83.05952:11941 83.89282:7786 85.07561:13939 87.0434:17824 88.03848:59507 92.10062:7297 113.07017:13334 114.01801:7757 114.05381:8771 115.00237:12839 124.48667:8237 131.03215:13493 131.08055:1977530 131.56009:7877 132.02934:18374 132.08446:80288	-11.67202703
3-Hydroxymyristic Acid	11.552	243.19594	[M-H]-	243.19601	59.01237:1415 69.31194:5636 83.02363:1853 85.20749:1291 99.92403:2339 106.03919:1326 110.02345:1978 116.92694:1497 121.70837:1324 123.8999:5201 132.95941:1427 138.6636:1529 146.95973:2309 174.95419:2326 181.15903:2173 197.18877:14293 218.08072:1454 225.14806:2568 225.1833:6430 232.68571:1305 241.17928:2028 243.03:2117 243.10252:11022 243.19539:635073 244.19847:78051	-0.28783367
Bromacil	2.364	259.00711	[M-H]-	259.00876	59.90507:1436 64.32748:1242 66.55334:1262 69.81377:2684 85.60197:1354 87.00745:14077 97.52119:1218 102.94672:14298 111.00757:27946 125.00862:1743 126.98718:16129 127.98919:1756 139.70389:1421 146.93793:20958 154.98233:62348 155.98445:5518 168.99796:43006 169.99855:6108 186.55635:1305 190.92616:5722 191.01907:32097 213.00291:1925 220.84346:2454 221.84285:31352 222.84283:7016	-6.370440907

					223.83929:15478	
Trimethadione	6.24	142.04951	[M-H2O-H]-	142.05042	52.25079:1157 54.1986:1193 54.29295:1214 71.0368:1251 75.00729:1307 76.79903:1202 81.83405:1246 99.04315:1660 100.03875:1232 126.27082:1383 133.71727:1261 134.78688:1266 137.22226:1129 142.0493:225946 143.03387:1403 143.05312:11029	-6.406176061
alpha-D-Glucose	0.886	179.05489	[M-H]-	179.05612	57.10406:3533 59.01205:158863 60.75393:3949 71.01191:75492 76.54308:4139 76.76714:3891 80.91508:4068 85.02799:4834 89.02288:69421 101.02248:18855 113.02229:4449 119.03315:8982 141.86647:15893 143.86333:24496 161.87408:8196 178.81395:58288 178.83209:18553 179.84116:7789	-6.869354703
[5-formyl-4,8-dihydroxy-3-(3-methoxy-3-oxoprop-1-en-2-yl)-8a-methyl-2,3,4,4a,5,6,7,8-octahydro-1H-naphthalen-2-yl] 3,4-dihydroxy-2-methylidenebutanoate	10.359	425.18118	[M-H]-	425.18173	57.03291:2100 59.01213:76651 67.8056:14911 67.80984:4893 71.01205:37708 72.99126:104788 73.02794:25230 83.01215:2918 85.02808:45104 87.00721:2593 89.02302:50932 95.0125:13572 96.83705:2173 96.84247:4530 99.00672:5754 101.02261:42070 103.00232:69656 103.03889:2579 113.02245:201664 114.02724:4249 129.01721:16517 131.03369:62198 133.01347:20272 149.04477:3081 157.01331:19091 163.02257:11273 175.02443:55079 176.02798:2273 193.03525:645067 194.03885:22980 249.14757:44755 250.15024:2147 425.17969:8169 425.26285:6643	-1.293564519
Salicylsulfuric acid	6.816	216.98048	[M-H]-	216.98122	69.09572:6336 79.95564:9998 83.04874:2327 84.00735:1661 87.00721:2518 88.03928:2672 93.03313:74529 94.03607:1631 96.95836:44147 103.91844:2727 120.95291:2361 127.86843:25133 137.02278:569868	-3.410433401

					138.0262:34964 144.94461:1649 146.07999:2998 146.95978:17239 148.94798:10956 159.85973:21440 172.98903:25045 173.99899:2007 193.11162:1667 199.09612:2270 205.86092:2493 216.9792:160864 217.10655:2218 217.98303:9659	
Quinolinol	9.14	144.0441	[M-H]-	144.04552	68.46437:1450 70.54704:2299 75.70653:1374 83.04874:7106 99.07964:2494 100.07498:9643 100.95221:1912 101.05908:7653 102.05401:2950 115.919:2908 116.04932:2960 118.03931:26056 126.05445:1404 144.04355:670716 144.06483:32296 145.04732:63474 145.08539:2151	-9.85799489
2-Hydroxyphenyl ethanol	1.317	121.07223	[M+H-H2O]+	121.07	53.03949:3042 56.32502:2574 57.05367:2382 67.49433:2279 68.8215:17691 74.06066:2449 75.0639:5672 79.05465:4558 82.84633:2429 91.05436:31712 93.07078:110155 94.06557:3998 94.07422:3845 95.05017:17133 95.06065:3760 102.80107:2340 103.0546:3941 105.03435:6016 105.04558:5606 112.03912:3250 121.02916:9666 121.03926:11366 121.0648:39293 121.0759:3781 121.09351:24886 122.05982:28514 122.07133:4154 122.09692:4132	18.41909639
3-Indolehydracrylic acid	8.731	206.08127	[M+CH3OH+H]+	206.08171	69.74413:12665 115.96455:19262 117.0583:5130 118.0653:777136 119.06923:42635 121.06573:6303 130.06471:613534 131.06937:44126 132.08138:167260 133.08473:16327 133.97447:4886 142.06541:18342 143.07285:13129 144.08119:154963 145.08368:18492 146.05983:649082 147.06441:41200 160.07613:1125685 161.07991:94403 170.06102:350802 171.06432:36150 188.07018:1100278 189.07487:107427 206.08083:805305 206.15387:4700 207.08372:82204 207.11449:6887 207.1767:4815	-2.135075451
3-(Indol-3-yl)propionic acid	9.796	190.08626	[M+H]+	190.08627	55.01869:194508 56.02195:3630 69.94621:5424 84.08146:2876 87.04517:5337 91.05514:2807 99.04497:8962 105.96336:5605 122.09049:2434 126.09107:2470 130.06479:1615859 131.06946:110018 131.08362:2954 144.08122:13738 144.10255:10620 162.11424:2589	-0.052607692

					167.05798:3248 172.07507:390757 173.07996:31644 190.0872:304951 191.08786:21976 191.14299:3574	
3-(2-Methylpropyl)pyridine	13.141	136.11218	[M+H]+	136.11263	54.03473:15915 67.05524:4409 67.78185:41406 68.05045:5354 81.04538:5473 81.07098:15063 91.05432:13301 93.07068:14078 95.06069:28983 95.08614:16604 96.05641:10847 96.06892:17887 96.84699:13116 97.06429:6462 107.07377:12359 109.01109:19232 109.07687:26085 110.07131:23194 119.03614:17859 121.08853:196528 121.10241:14479 122.09178:15817 136.02141:472823 136.06198:525598 136.07407:17284 136.08789:80222 136.11172:1321332 137.02516:34444 137.04576:54757 137.06551:13913 137.08174:79039 137.10661:99818 137.11575:110544	-3.306085556
N-[1-(2-Methylbenzyl)-1H-Pyrazol-4-Yl]-3,4-Dihydroisoquinoline-2(1H)-Carbothioamide	7.146	363.16241	[M+Na]+	363.16379	62.01109:1875 71.02145:3070 261.12994:4658 305.15549:705436 306.15891:76000 363.16171:3566724 364.16776:478647	-3.799938314
Glycerophosphoglycerol	0.889	245.04288	[M-H]-	245.04263	59.01215:52658 69.89738:2844 71.01197:16529 73.0277:1803 78.95763:7130 87.00719:4971 89.02297:45324 92.92596:1955 94.92359:2533 96.84155:1389 101.02267:84113 102.02563:1431 102.95461:5080 113.02226:4938 119.03328:111355 120.03712:5560 124.99036:9824 125.00862:2086 126.90299:1656 128.8907:2521 130.99086:1283 149.04474:5121 152.99425:28611 157.03554:5609 164.83455:18315 171.00577:13382 176.89723:2340 176.91037:9037	1.020230643

					179.05453:1621 186.86607:1770 187.07217:5627 191.05634:2723 209.06606:34119 210.0675:1744 216.25343:1435 245.04381:70037 245.75119:7110 246.04742:3365 247.27092:1491	
8-HETE	12.486	303.23135	[M+H-H2O]+	303.23099	53.00315:11463 55.05512:275317 56.05847:13172 57.07078:145879 59.05001:21909 67.05516:670739 68.05833:21326 69.07063:426153 71.04976:58250 71.08615:65403 73.06538:22837 77.0393:10152 79.05519:297537 80.05792:10850 81.0709:682173 82.07346:25318 83.04974:91178 83.08633:131836 85.029:23321 85.06535:98874 85.10194:61525 87.0451:28802 91.05502:831665 92.05791:47619 93.07057:448575 94.07409:23595 95.04906:14185 95.08604:528397 96.08939:26093 97.0651:46196 97.10147:42793 99.04511:23897 99.08129:13583 101.05989:112808 105.06998:1074898 106.07393:58151 107.08624:309856 108.08955:18794 109.06512:42144 109.10164:151469 111.0812:13112 113.06031:24289 115.07535:15632 117.07023:371393 118.0731:27492 119.08533:770279 120.08848:60251 121.06571:46423 121.10107:281237 122.10452:22768 123.08032:47148 123.11651:122869 127.07566:19786 128.06218:9302 129.07014:355340 130.07274:29079 131.08633:852320 132.08936:72131 133.0661:24384 133.10069:466218 134.10495:37729 135.08138:80501 135.117:128187 136.12054:13546 137.1324:114851 139.07474:19966 141.06996:197604 142.07326:15439 143.08575:191033 144.08922:14876 145.06526:17497 145.10176:607028 146.10463:55086 147.07961:72481 147.11682:224192 148.12051:20919 149.09631:200638 149.13246:83144 150.0993:18704 151.14784:23372 153.09073:36844 155.08621:135288 156.08943:12101 157.10057:502870 158.10422:52030 159.08015:75054 159.1162:318061 160.11958:29676 161.09706:119174 161.13196:314726 162.1003:10851 162.13551:35363	1.187213748

					163.11125:39463 163.14854:69859 165.09128:31504 167.10686:94249 169.10167:208729 170.10484:27747 171.11754:224879 172.11954:29695 173.09633:176677 173.1329:323810 174.09834:14596 174.13519:30783 175.112:59153 175.14891:358120 176.15103:36746 177.09077:42048 177.16435:29760 179.10603:79294 181.12096:30266 183.11641:485345 184.1196:55019 185.13292:70875 187.11244:269883 187.1487:175451 188.11369:31314 188.15312:14815 189.12866:17505 189.16341:213441 190.16623:23878 191.10759:170250 192.11142:19508 193.12337:61618 197.13243:206521 198.1368:26720 199.14813:47408 201.12704:545328 201.16476:291926 202.13205:73588 202.16769:37668 203.14458:29396 203.17973:137176 204.18309:16226 205.12186:187530 206.12553:15419 207.13692:33996 209.15302:21619 211.14716:74429 213.16374:47663 215.14301:202633 215.17767:75770 216.14626:27944 217.15932:33908 217.19557:29668 219.13928:332851 220.14319:44853 221.15312:63059 223.16965:24857 225.16479:41807 229.15898:44392 229.19484:17963 231.17551:17928 233.15512:297812 234.15764:43801 235.17018:12803 237.18385:29354 239.17802:44310 241.1942:207040 242.19919:33146 243.17438:22748 243.21034:164741 244.21466:21651 247.16827:89510 248.17355:16739 257.18866:104984 257.22778:63778 258.19308:15189 258.22852:11502 261.18533:36683 267.20898:300577 268.21436:56553 275.20123:28456 285.22195:531616 286.22495:98514 303.23288:4113140 304.23691:776660	
15-Methylheptadecanoylcarnitine	11.553	428.3743	[M+H]+	428.37399	57.03443:18962 57.07083:48077 58.06581:2753 60.08162:342780 67.05522:7697 68.10316:18532 69.07067:8990 71.08617:49057 81.07098:19166 83.08649:18338 85.02898:2741952 85.10188:28944 86.03264:75391 95.08608:38317 96.84876:3398 97.1015:15700	0.723666719

					99.11697:2870 103.03967:2641 109.10193:18484 111.11687:11355 123.1166:11426 126.05479:4119 136.06198:2794 137.13245:8111 144.10233:64729 165.09146:13195 184.0744:3059 198.90842:3383 203.12756:3648 204.13077:3701 267.26715:40201 285.27692:7630 369.3031:140898 370.30103:24220 393.27542:3931 411.29126:4378 411.33875:5209 428.371:1567151 429.37314:277203	
PyroGlu-Pro-Arg	12.616	383.20395	[M+Na]+	383.20377	57.07093:12650 69.59138:3300 69.59413:3623 81.0703:2855 89.06053:9351 95.08614:1929 96.84428:2157 104.10738:8135 121.06569:12971 133.08623:3590 165.09117:8067 184.07446:9770 207.06349:13340 211.05698:2881 225.07222:2396 239.38202:1950 267.12222:40940 268.12296:2650 271.07855:3277 271.15475:4436 281.13629:30911 282.14081:2732 327.07944:2556 327.14069:95783 328.14233:10830 338.34131:3714 383.20627:1218895 384.20468:191924	0.46972398
Hesperetin 7-O-glucuronide	8.87	477.10358	[M-H]-	477.1033	57.03292:8969 59.01213:54213 69.272:8528 71.01204:32540 72.99104:6926 75.00684:27548 85.02805:139987 87.00738:14426 89.02289:13410 95.01235:53196 96.83886:2548 99.0069:46950 101.02248:2338 103.00224:32298 111.00744:2446 113.02235:378666 114.02609:12621 115.00225:14788 117.0181:22066 125.0233:13668 129.01704:15799 139.00182:6188 151.00203:17040 157.01317:8939 164.01024:15536 174.02946:5142 175.02438:118716 176.02577:3545 187.00671:2970 195.99934:2649 199.04018:6253 201.01765:7029 215.06999:2767 217.04892:2136 241.05142:2067 242.05925:25037 257.08264:17748 258.05139:7065 283.05981:7185 286.04584:44352 287.04916:2783 301.07129:654177 302.07458:62308 343.0838:2043 395.50592:1894 477.10226:147846 478.1124:18710	0.586875002
Octaethylene	7.255	393.21017	[M+Na]+	393.20953	52.60728:2725 56.96302:2666 69.19062:6172 69.19444:9213 149.024:2688	1.627630948

glycol					170.87381:2498 195.43208:2838 228.97813:2949 254.53409:2824 297.68643:3421 393.20892:3692326 394.20923:455757	
Bidwillon A	7.371	407.18811	[M+Na]+	407.18643	70.03339:3959 70.03728:3863 77.68346:2688 115.51229:2485 146.5062:2728 150.58031:2534 310.33151:2626 324.99124:2826 349.18436:528952 350.18887:55488 407.1853:2216501 408.19238:289866	4.125874234
6,15-Diketo-13,14-dihydroprostaglandin F1alpha	9.829	353.23166	[M+H-H2O]+	353.229	53.00322:4458 55.01864:14516 55.05516:16554 57.03444:3855 57.07081:7075 67.05513:32971 69.07056:27962 71.04983:12360 71.08617:302770 72.08981:13023 79.05527:28139 81.0709:88719 83.04977:11777 83.0864:10562 85.06532:25694 87.04516:4479 91.05508:44062 93.07063:48673 95.05007:8777 95.08616:32235 97.06507:13343 97.10147:3864 99.04495:3792 99.08141:349496 100.08411:19006 101.06087:2321 105.07001:104973 106.07412:2916 107.04955:35882 107.08624:32173 109.06507:45872 109.1018:7195 111.08145:12477 113.06052:2542 115.1118:3032 117.07029:60345 118.07384:2913 119.04957:41135 119.08527:30263 120.09003:2699 121.0658:36196 121.10112:12059 123.08034:16591 123.11662:3145 125.09656:2499 127.07595:16056 129.07014:21493 131.08646:44560 133.06448:15144 133.10069:22746 135.08153:61519 135.11714:4433 136.08307:4114 137.0594:3685 137.09593:3195 137.13284:16230 139.11234:49872 141.07005:22788 141.09235:3285 141.12732:3088 143.0858:41475 144.08942:4500 145.06544:7432 145.10193:21860 147.08153:13307 147.11674:16379 149.09648:8165 149.13274:2397 151.0757:3327 151.11249:24214 152.07922:2433 153.091:10859 155.0867:33706 157.06519:7799 157.1008:78386 158.10458:7680 159.07991:22846 159.11633:30220 161.09541:29461 161.13205:3760 163.07574:2948 163.11131:7030 163.14885:2956 165.12743:21091	7.530525523

					167.08452:14591 167.144:3926 168.09282:3180 169.10191:22608 171.11769:17649 173.09666:15474 173.13319:39754 175.11192:42234 175.14906:2947 177.0912:15756 177.12663:7675 179.08603:14360 179.10611:12563 179.14246:3469 181.10081:22412 183.10008:3541 183.11653:91222 183.13768:33560 184.11928:12428 185.09718:94596 185.13298:7881 186.09955:9249 187.11252:7508 187.14894:7607 189.0919:3855 189.12668:22200 191.10818:19268 191.14311:28221 192.09389:2765 193.10037:36398 193.12328:11442 194.10442:3631 195.13701:4110 197.09576:9872 197.13251:8007 199.11122:13847 201.12732:123816 202.13174:18301 203.10587:125032 203.14374:14473 204.11134:15495 205.1581:8276 207.11732:16455 207.13712:17436 209.15353:13307 211.1122:10000 211.14711:3047 215.1071:8218 215.14301:12414 217.12289:11220 217.1593:3160 219.1396:67055 220.13971:8168 221.11513:25280 221.15306:4539 225.12662:3681 227.18127:6581 233.15242:44170 234.15791:6504 237.16692:3166 239.18187:7409 245.19022:7663 251.16327:3061 253.1945:17191 255.17458:10266 257.189:2711 263.17859:31162 264.1861:2795 271.20551:18133 273.18561:25308 274.19083:3063 281.19052:73901 282.19467:12149 289.21533:7415 291.19424:24931 292.20038:3847 299.20255:112702 300.20203:24504 317.21063:117419 318.21475:20579 335.22314:61457 336.22659:13007 353.22903:97433 354.23331:17341	
Methyl 5,6-diacetyloxy-10-hydroxy-2,4b,7,7,10a,12a-hexamethyl-	1.695	280.99542	[M+H]+	280.99219	69.03423:9129 72.93776:7743 73.02942:10611 74.95359:2759 80.9486:13390 82.9458:7533 85.02915:18942 87.045:11970 90.94801:15595 96.92205:3027 97.02892:3155 108.95821:3320 109.02924:13334 113.96437:6675 114.94718:17117 116.96338:22885 118.94257:9940 119.95163:2493 127.03906:10961 128.96324:2788 132.95822:105015	11.49498141

12-methylidene-1,4,8-trioxo-4a,5,6,6a,9,10,10b,11-octahydronaphtho[1,2-h]isochromene-2-carboxylate					134.97427:10823 136.95291:21192 140.9399:3421 140.96371:3202 141.9595:6677 143.92717:2695 144.95813:23473 146.97316:9622 149.94095:3731 150.96857:41573 151.93706:2932 155.9747:22948 156.95886:2243 158.97369:50059 159.96867:4037 160.95357:50185 162.96935:15948 164.91896:52493 168.96016:3769 170.97295:19276 172.95393:9820 174.96893:900342 175.97206:45185 176.98566:16560 178.96283:17458 181.98894:2577 185.95999:3458 186.96931:120219 187.9724:9054 188.98544:16395 190.96426:19978 192.98036:37864 196.95415:3030 198.96977:78491 199.97163:3982 201.97932:9762 202.96297:49578 204.97972:18474 206.99585:2345 208.88414:3610 215.99544:56951 216.98044:43068 220.97528:139236 221.97572:8069 222.87784:12581 226.89255:3149 227.99538:11348 234.98926:120592 235.99356:10078 239.99438:10513 243.98944:15531 244.97556:2644 247.81834:2433 248.87993:3759 253.00032:80984 253.88062:8094 254.00342:8522 258.00482:22006 262.00061:9218 262.98447:34660 266.82346:16278 276.01718:12349 276.84976:2865 280.99341:81997 281.10089:3468 281.99701:9640	
gamma-Glutamylphenylalanine	7.025	295.12921	[M+H]+	295.12888	53.80005:2615 59.42974:2513 61.3181:2623 69.12463:15761 84.04494:222871 85.04836:4535 91.05515:2539 102.05483:5207 103.03971:2634 103.05459:16753 107.04961:10143 120.08135:966341 121.08485:65005 130.0508:101845 131.04938:49007 136.06203:4465 149.06023:36947 166.08549:639284 167.09084:47407 186.09238:86387 187.09557:4801 204.10318:2771 209.36026:2448 232.09555:93723 233.10129:3394 253.88063:3858 278.1037:84200 279.10468:6002 295.13101:80960 296.13376:11868	1.118155567
4-	13.466	185.02348	[M+CH3O	185.02092	57.93597:23424 58.94349:56249 67.74052:175309 74.93836:145921	13.83627322

Hydroxycoumarin			H+H]+		75.94622:44969 84.94654:22036 84.96059:165684 92.94903:56642 96.8488:28611 98.96158:104436 99.96944:62226 102.95644:61938 102.97031:22164 113.96439:17513 114.95631:875686 115.96473:614476 116.97198:1071830 117.98031:2319480 118.9832:29455 120.96496:111908 125.97301:56616 132.967:705622 133.97472:261207 135.98981:49898 139.98793:435738 140.07117:21373 140.99567:417749 143.95976:127532 143.98125:68116 143.99763:29009 148.96193:20957 150.97717:47876 155.98239:79578 156.99089:102998 161.96953:684263 162.00664:47747 171.97862:14217 179.97984:95620 184.98506:151938 185.09244:25423 185.16409:18095 186.11404:14436	
3-Hydroxysuberic acid	8.251	189.07591	[M-H]-	189.07681	56.9623:1698 57.96035:1656 59.01237:6909 65.99711:2313 83.02362:1709 91.02863:3399 96.84071:14919 99.0797:94797 100.08337:2741 100.92431:2284 100.95229:66171 101.9231:2678 101.95046:48454 102.92193:2475 103.91843:7647 104.92641:2119 106.03918:2959 114.98715:2002 115.89177:1414 116.96291:5550 117.94564:7043 119.02328:2836 120.04462:7168 121.03873:1819 121.9494:1674 127.07533:132947 128.07852:7123 129.0546:431588 130.0574:17928 131.06912:2488 135.05444:2324 142.98199:7637 143.86331:2664 144.0435:1700 144.89496:2751 144.92143:1981 144.94136:16252 144.95773:87893 145.08531:24424 145.94093:38880 145.95949:2902 147.06622:2210 148.05054:8622 160.89212:2324 161.8936:2005 162.03044:7828 162.06566:6731 164.03435:2078 171.06497:35094 172.03909:8163 172.06906:2073 189.01895:5504 189.07561:345931 189.84984:3362 190.04846:1855 190.06339:3057 190.07768:22572	-4.7599703
Palmitoylcarnitine	11.172	400.34201	[M+H]+	400.34207	55.05514:8361 57.03449:47147 57.0709:77508 58.06582:8579 60.08163:727058 67.05523:24471 69.07068:30970 69.74689:16882	-0.149871833

					71.08624:87955 81.07025:51455 83.08647:55117 85.02903:6222634 85.1019:67046 86.03271:173547 88.07632:20434 95.08622:63487 97.10147:28552 109.10201:37106 123.11662:20765 137.13248:8562 144.10262:162294 145.10522:7340 239.2382:118793 240.24136:10019 257.24768:25553 338.34152:57449 340.22614:7696 341.2706:375594 342.27115:65577 400.34491:3293781 401.34222:585935	
Prostaglandin E2	10.284	335.22223	[M+H-H2O]+	335.22198	55.01878:17671 55.0552:10554 57.07083:2848 67.05524:20988 68.77927:4584 68.78306:6213 69.07072:43845 71.08623:96930 72.08978:2484 79.05532:9544 81.07092:22748 83.0498:5968 85.06547:2537 85.10194:1918 87.04518:2107 91.05507:31735 93.07079:17463 95.05009:3544 95.08614:12879 97.0652:7461 97.10149:1507 99.08141:69526 100.08424:3021 101.05993:1571 105.03432:1828 105.07011:20500 107.04961:6890 107.08633:10425 109.06509:5365 109.10182:3226 111.11691:2621 113.09705:2128 115.05563:1855 117.0703:45790 118.07387:5585 119.08537:15343 121.06538:9885 121.10078:10113 123.08038:6708 123.11665:6501 125.09526:1775 127.8976:1446 129.07014:15413 131.08655:20994 133.06438:6230 133.10211:11497 135.08127:13238 137.09596:1712 139.11223:59114 140.11519:3647 141.07013:9500 143.08568:21011 145.06549:2573 145.10216:12306 147.07977:12644 147.11693:6979 149.09651:5760 149.13278:2688 151.11243:11431 155.08653:13671 157.10059:32133 158.10461:3602 159.08012:8874 159.11781:14720 161.09711:31950 161.1321:3116 162.10056:2681 163.07579:2659 163.11134:2799 163.14691:1987 165.09117:3459 165.12738:1415 167.08463:6321 169.08731:1359 169.10159:12981 171.08162:1608 171.11771:6873 173.09641:8908 173.1329:11313 175.1116:18502 175.14909:1484	0.745774487

					177.09091:5336 177.12668:1761 179.1084:5754 181.10274:2055 183.08133:1543 183.11638:21336 184.07446:7303 184.11948:1553 185.09726:21757 185.13303:2316 186.09959:2748 187.11256:5657 187.14899:2892 189.08949:5472 189.12895:2944 191.10793:2842 191.14301:2700 193.10297:1679 193.12332:2585 197.09843:1734 201.12761:24780 202.13188:2901 203.10548:23541 203.14418:5997 205.16093:1867 207.11737:1825 207.13716:2626 211.11224:2777 215.14307:7235 217.12294:1788 219.13896:17008 221.11882:5892 225.12666:2472 227.17809:3324 229.19519:3108 233.11816:2354 233.1553:2970 237.16698:1747 239.17842:2986 245.19028:6274 250.25223:2491 251.12936:6097 253.19528:6668 255.17465:6910 257.18906:5235 263.17905:2980 271.20474:13349 272.20801:2565 273.18604:9783 275.20139:1478 278.64972:1624 281.18924:16715 289.21539:7377 291.1937:12768 299.20193:46977 300.20615:3305 317.21014:41976 318.21454:8179 335.22235:63084 336.22412:15420	
(2S,3S)-3-decyl-2-hydroxy-2-(3-methoxy-3-oxopropyl)butanedioic acid	12.614	361.22278	[M+H]+	361.22208	57.07073:42168 68.99796:15335 69.0707:2670 87.00822:3337 111.00775:16539 129.0188:1059611 130.02113:44100 139.00342:32079 140.00671:2244 147.02896:10929 157.01297:98343 184.07446:7267 185.08054:628907 186.08508:48755 203.0892:7694 211.76248:2150 213.07497:7617 249.06624:3200 259.1524:32465 260.15625:2887 313.45547:2165 333.28137:2173 361.15079:16680 361.233:224291 362.23798:45061 362.32709:16779	1.937866035
3,4,5,6-Tetrahydrohippuric acid	8.317	182.08099	[M-H]-	182.08171	66.03323:6172 68.0126:2514 70.02728:3578 70.89167:1324 74.02289:21188 82.03947:1733 83.02386:13170 92.91834:2325 93.91856:2833 94.91502:48651 96.00731:2658 97.03892:2838 98.02277:2974 103.91844:1696 111.01854:1710 112.0508:2306 113.03368:1776	-3.954268663

					114.01801:1691 123.03074:1768 125.03384:3198 127.04958:1949 136.07539:14496 136.89359:2338 136.90877:1578 137.90939:6432 138.05389:6287 138.09088:97936 138.90552:37840 138.96298:8804 139.05002:2055 139.09514:5636 140.04439:2365 154.94757:1992 155.03358:3016 156.05472:2165 164.07024:1641 181.89647:11050 182.08064:357917 182.92009:6857 182.95294:7960 183.08536:25921	
Acetylcarnitine	1.41	204.12363	[M+H]+	204.12299	57.03444:50023 58.06625:44668 60.0816:4512444 61.08469:35653 68.87562:220376 70.06564:134962 71.06178:36673 71.06917:43399 76.03982:54209 84.0449:120887 85.02897:24345986 86.03268:742497 88.0873:119502 116.07123:161901 117.07513:133016 126.05482:104682 130.05069:143527 138.05551:43670 144.06482:37648 144.10234:920008 145.05043:4056377 146.05313:186513 159.13148:119536 187.10768:37826 204.12241:11468843 204.15034:523407 205.12732:814603	3.135364615
PE(16:0/18:2)	12.465	716.52155	[M+H]+	716.52246	53.03946:17961 55.05525:556596 57.03443:58657 57.07091:388792 59.05005:61120 60.0816:19715 62.06113:20596 65.03957:18701 67.05531:967536 68.05842:26982 69.07079:1580715 71.0501:85320 71.08631:245319 79.05476:276172 81.07038:1478305 82.07362:48921 83.08647:1312002 85.06555:87962 85.10204:122762 86.09712:75342 89.0605:17593 93.07079:513320 95.08625:1573547 96.08943:69959 97.06533:89554 97.1016:1009777 98.10468:46544 99.08048:67556 101.09652:20093 107.08651:409072 109.10192:748970 110.10516:20339 111.08035:111785 111.11701:504290 113.09586:75550 121.10125:1175356 122.10454:61647 123.11679:373350 125.09655:60966 125.13239:64132 127.1124:18860 133.08618:22682 135.11725:1081207 136.12071:60558 137.13255:284456 139.11227:60179 149.13292:418129 151.14798:100642 153.12732:140864 155.01134:20689 163.14897:158525 165.16356:80392	-1.270022994

					167.144:61007 177.16518:82869 184.07448:959192 185.07565:23581 205.1971:18205 219.21043:213663 237.22244:490833 238.22348:45983 247.24226:131402 265.25201:508155 266.25476:28422 280.26266:118166 308.29388:471330 309.29706:25899 311.25632:207617 312.25876:26870 339.28802:187268 434.26672:18746 452.28339:20582 533.34851:16969 575.50574:10276330 576.50244:2698922 716.53192:27039	
1-(9Z-Eicosenoyl)-sn-glycero-3-phosphocholine	11.53	550.38605	[M+H-H2O]+	550.38672	55.05514:15861 57.07081:22602 58.06624:26791 60.08163:203169 67.05523:10523 69.07071:31534 69.49016:21051 71.07369:33237 71.0862:27629 81.07085:31787 83.08633:40404 86.09713:598974 87.09987:8470 95.08607:33310 97.10146:18718 98.98515:7098 104.1074:3000124 105.11082:68939 109.10179:8605 121.10111:7848 123.11662:9514 124.99979:207302 135.11714:8014 163.01454:24103 181.02643:7711 184.07442:3770045 185.07814:80377 258.11099:67600 367.31882:54085 473.30493:21280 532.38013:275361 533.38373:38385 550.38177:1010896 551.38702:164243	-1.217325883
[6,10a-Dihydroxy-4-(hydroxymethyl)-4,7,11b-trimethyl-9-oxo-1,2,3,4a,5,6,6a,7,11,11a-decahydronaphtho[2,1-][1]benzouran-5-yl]	8.378	426.24637	[M+2H]2+	426.24863	61.02904:4743 67.05524:4164 69.03425:237446 70.03783:9691 71.04985:4362 73.02946:58477 73.06564:26039 78.03922:8564 80.05501:17629 81.03429:7903 81.07098:4272 83.0498:55411 84.05367:2912 87.04515:251013 88.04811:13192 89.06052:822930 90.06362:28018 91.04772:2801 95.05:19172 99.04494:133557 100.05325:22591 101.05991:3679 102.06851:9813 107.07039:24481 109.06506:8474 111.04467:94440 112.04808:2711 113.06055:22373 115.07551:3448 117.09167:16283 122.06487:62040 122.56783:3420 124.08091:14305 125.05941:21606 129.05499:41042 130.08595:30277 131.07079:33456 133.08612:381780 134.08888:23836 137.0594:12630 144.07799:47282 146.09499:3651 151.09685:11644 153.08383:3589	-5.302069827

acetate					155.07007:66105 156.07486:3525 157.08574:4524 166.0916:26615 173.08127:22213 175.09605:24009 177.11305:81819 178.1145:7576 186.60381:3556 188.10362:14267 195.12408:12900 197.11076:13550 199.09714:20174 203.12741:18852 210.11789:3174 217.10782:16277 219.12363:23866 221.13748:10178 239.15103:14612 241.1369:15724 241.6389:3713 243.12421:8464 261.13431:21131 263.15067:4252 283.17737:11525 287.15063:4109 305.16031:14101 306.16382:2593 327.20282:11873 349.18417:3729 371.2291:4730 395.22079:2891 415.25143:3164 426.21707:87685 426.71799:46955 427.22134:8274	
Dimethyl myristamine	11.037	242.28458	[M+H]+	242.28418	53.00323:5039 57.07088:247949 58.07423:5247 59.57339:2297 69.0707:3903 69.74857:8439 71.0863:207053 72.08978:2751 74.03043:2652 83.0857:3296 84.08147:2980 85.10201:128587 86.09715:2396 86.10548:3830 93.03663:4053 94.61916:2293 99.117:5831 109.10075:2802 112.07623:2647 147.11693:2807 149.13106:2476 152.73416:2428 187.69896:2392 197.12993:2423 197.16669:3637 230.78966:2253 242.28502:777098 243.13504:5539 243.17024:20475 243.21057:15269 243.28615:112216	1.650953851
4-ethyl-7,9-dimethyl-3-phenyl-5,7,9-trihydro-1H,4H-1,2,4-triazino[4,3-h]purine -6,8-dione	10.211	337.14218	[M-H]-	337.14182	69.57209:1292 71.0266:1862 78.95765:181937 96.96825:456334 97.68127:1354 102.94675:27851 108.97908:1759 133.51808:1348 145.06219:1655 145.92105:1262 146.93756:49120 147.93958:1387 167.93016:1372 174.33255:1541 190.92805:14785 236.10966:1398 265.90466:1400 337.14145:88165 337.20529:51038 338.14752:3236 338.20651:1979	1.067799895
[3-Methyl-1-	6.27	339.1554	[M+CH3O	339.15601	68.82329:31908 72.04508:6492 85.02905:70788 97.02886:35071	-1.79858231

[3-methyl-1-[3-methyl-1-oxo-1-(2,3,4,5,6-pentahydroxyhexoxy)pentan-2-yl]oxy-1-oxopentan-2-yl]oxy-1-oxopentan-2-yl] 2-hydroxy-3-methylpentanoate			H+H]+		103.03969:19522 126.05481:10217 127.03905:18857 132.08136:18947 134.05983:18554 146.05981:28744 148.07619:94551 156.06552:17732 160.07616:4299200 161.08002:356236 162.07507:7230 172.07489:161690 173.08125:17796 174.07678:204537 175.07874:7029 177.10202:109684 184.07703:37925 186.09232:6957 189.10168:34451 190.08719:9113 192.08632:22951 200.07088:17601 201.10284:299822 202.08577:214304 203.08916:26030 214.08736:26228 219.11162:58151 226.08757:29099 244.09785:7983 255.11275:18772 256.09686:32792 262.10922:7504 274.10898:184244 275.11032:25418 285.1225:38360 286.1059:7488 292.11966:291751 293.12189:45521 303.13293:94084 304.11581:191490 305.11957:33597 310.13004:49452 321.14697:413129 322.12289:233619 323.13171:47460 339.15723:443271 340.16122:72336	
1-acetyl-4-[(4-chlorophenyl) methoxy]-3-methoxybenzene	3.464	289.06708	[M-H]-	289.06372	71.01355:1299 88.46311:1200 89.11617:1337 99.92404:1416 103.91845:2829 110.02346:5821 111.01855:5922 128.45882:1338 130.94257:2030 130.98268:12403 146.95982:13831 152.03496:1696 157.88072:1328 158.97765:12815 174.95407:32575 177.91251:1557 199.94078:2400 200.05612:7346 237.23543:1283 243.06224:24365 261.89166:6320 278.59213:1469	11.62373473
Oleoylcarnitine	11.272	426.35696	[M+CH3OH+H]+	426.35779	55.05514:30398 57.03448:57756 57.0708:84326 58.06582:17182 60.0816:783571 67.05531:35219 69.07064:78018 69.63322:20473 71.08628:67756 79.05461:9655 81.07026:62229 83.08642:73685 85.029:5744720 85.10184:44644 86.03271:134245 93.07066:27531 95.08609:84377 97.06517:19035 97.10152:62676 103.03967:9260 107.08635:23084 109.10195:48367 111.08134:20577 111.117:32977 121.10128:61762 123.11661:20740 125.09655:20580 135.11725:70387	-1.94672179

					137.13246:10231 139.1124:35504 144.10243:192162 149.13274:38335 151.14792:10783 153.12692:16040 157.04977:32269 163.14885:23765 177.16464:18714 181.15817:19442 195.17577:9672 203.12758:22281 227.12605:8449 241.14444:20032 247.24214:45765 255.15813:39198 265.25211:86331 266.25476:10649 269.17551:44993 283.19135:43312 297.20398:44738 311.22:39277 367.28519:175715 368.28897:21972 426.35977:3381876 427.36365:627173	
LPE(18:1)	12.502	480.30966	[M+H]+	480.30847	78.95723:256939 96.84069:60874 96.95826:14718 133.73927:6704 140.01007:245392 152.99446:70283 196.03596:888942 197.04185:15415 208.14035:7027 214.04771:186696 225.8578:7561 281.24838:8564411 282.25296:846870 287.91824:6942 325.8212:7432 406.013:7005 417.24341:9757 478.29007:1985289 479.29324:310147	0.397245284
GABA	1.393	104.07109	[M+H]+	104.07058	58.0659:1031636 59.06944:21530 59.07375:30230 60.08167:2899060 61.08516:22704 71.02034:28798 87.04518:18905 104.10738:9044387 105.00371:22052 105.0711:30796 105.11097:270638	4.900520397
Gly-Trp-Gly	6.905	319.13654	[M+Na]+	319.14008	50.27039:4538 68.3678:16973 68.37209:29435 72.28405:4373 92.06217:4472 98.9889:4676 143.24019:4789 147.99065:4497 226.06178:4958 229.63347:5488 261.13:943684 262.13275:66407 312.96652:4826 319.13708:7358084 320.13971:877761	-11.0923078
Myristic Monoethanolamide	10.027	272.25854	[M+H]+	272.25839	57.07066:38195 58.06621:15702 69.07069:3336 70.066:20210 71.08662:19785 72.08163:11473 81.07028:2723 83.08711:2828 85.10192:13554 86.0606:154419 88.07614:20960 100.07645:238582 101.08014:9814 104.07088:30812 118.08716:42498 129.0188:9406 153.01917:2552 159.11624:3441 171.03065:2513 177.36163:2853 195.13992:15572 201.12985:3709 212.23677:27064 213.14868:3180 219.13947:4576 226.25208:3287 228.23395:3572 237.1496:2948	0.550947209

					238.45033:3750 243.63193:3089 254.24937:2130746 255.25212:328361 272.25922:1076618 273.18597:8837 273.26324:164858	
SKF-75670 hydrobromide	6.659	270.15414	[M+NH4]+	270.151	59.05015:32610 71.08623:3816 87.04445:114900 88.04813:3651 89.06051:309000 90.06374:9267 95.08614:2790 103.0397:437928 103.07528:25063 104.04295:20281 107.07062:14133 119.07055:16477 121.04935:2407 121.08598:138299 122.08923:3680 130.08588:4274 131.0708:129055 132.0742:3926 133.08618:245130 134.08931:11501 147.06442:46500 151.09653:21964 163.0976:15638 165.07507:3049 177.11327:14045 181.99129:2666 191.0929:8755 195.12154:4665 207.123:209254 208.12668:17442 209.10193:2987 219.44443:2303 235.11922:79807 236.12085:4932 239.15036:9990 253.12656:220548 254.12973:18682 260.00906:3403	11.62312929
Furanone A	1.305	85.02907	[2M+H]+	85.02895	55.01875:27786 56.05019:30061 56.96545:34201 57.0345:227115 57.05368:60085 57.07086:40642 58.06598:38405 67.05524:97721 68.05049:65571 68.89247:26626 69.03436:85878 69.07076:70913 71.07375:47577 84.94279:75539 84.96037:33084 85.02901:900042 85.04826:246956 85.06539:57577 85.07664:67400 85.08469:67237 86.02466:34790 86.03211:35110 86.06078:1153687 86.09713:894106	1.411284039
2-(methylethylidene)butanedioic acid	6.933	157.04942	[M-H]-	157.05061	58.02821:5417 59.01239:12946 65.0131:3042 65.9966:1558 68.19491:14792 71.01241:2105 72.99163:2341 74.02268:1720 76.22522:1459 81.04431:2402 85.02798:2533 86.02267:7294 87.00721:1920 88.98624:5305 89.02286:184361 90.02629:2396 91.02863:1496 95.04832:111002 96.05193:5238 96.84157:5172 97.0643:1644 99.92498:2109 102.05499:2003 102.98733:2880 109.03944:119387 110.04315:2732 113.05872:351314 114.05495:4645 114.06304:18715 114.09086:34211 115.00236:6459 116.07079:17792 127.00205:1614 128.96709:2408 129.00296:10798	-7.577175281

					129.06577:2183 130.03955:14840 130.08559:5786 139.03757:4825 140.06949:19967 156.89378:11471 156.99825:2971 157.04858:240723 157.06793:11068 157.08475:10491 157.86189:3210 158.03511:2867 158.05539:12149 158.08023:171987	
Isonerylgeraniol-18-oic acid	12.485	285.22153	[M-2H2O+H]+	285.22101	55.05514:14154 57.07093:33997 67.05533:33700 69.07069:15861 71.04985:5697 71.08621:15643 73.06553:5473 75.02688:4010 79.05462:4215 81.0704:18637 83.04977:4529 83.0864:5179 85.06561:21860 85.10209:23364 89.06051:3813 91.05513:9389 93.03746:8846 93.07066:4778 95.08611:11239 97.06519:3786 105.07021:19086 107.08636:3856 109.1018:3779 117.07051:32072 119.0854:5368 121.10113:3910 129.07031:248028 130.07291:17094 131.08514:146567 132.08856:9145 141.07002:62166 142.07346:4526 143.08603:37089 145.1019:131250 146.10504:9285 155.0849:56157 157.10085:139997 158.10458:14400 159.08026:60279 159.1162:20584 161.09715:4073 161.13205:4760 169.10187:73903 170.10529:5173 171.11781:95175 172.11984:10025 173.09656:52173 173.13297:50761 175.14906:10674 176.14278:29541 177.14677:5781 183.1167:177553 184.11986:20140 185.13289:44805 187.11273:39552 187.14896:48316 188.11421:4470 188.15092:5335 197.13269:95466 198.13647:13397 199.14828:34580 201.1273:138143 201.16519:101683 202.13231:16384 202.16728:13244 203.14413:3457 211.14716:56660 212.15169:4942 213.16351:29233 214.1676:4150 215.14333:45607 215.1787:36053 216.1823:3601 225.1646:33441 229.15889:16881 229.19514:5443 239.1433:13310 239.17838:8290 240.14958:3625 241.19423:114649 242.1996:22252 243.10263:10830 243.17455:3143 252.11732:8614 253.11887:110413 254.11096:196211 257.22812:11305 267.13931:36005 267.20929:281937	1.823147601

					268.21451:56711 285.15222:129598 285.22211:1196161 285.2998:26088 286.15179:17157 286.22525:213019 286.27579:7415 286.30795:4883	
gamma-Glutamyltyrosine	6.38	311.1235	[M+H]+	311.12378	69.76182:10618 73.02945:61978 84.04491:157657 85.0491:5284 89.06056:63939 102.05581:5070 119.04958:50526 120.08132:6107 123.04404:94446 130.05069:94238 133.08612:29014 136.07555:425704 137.07945:27923 147.04414:68506 165.05495:526843 166.05872:37763 167.09076:4704 177.11327:5299 180.08763:590908 182.08211:479246 183.08574:38670 202.08554:68761 248.09216:65545 249.09605:4713 265.12531:5718 283.17288:4538 293.12161:19857 294.09503:65964 295.10217:6366 311.12598:90027 312.12805:13309	-0.899963352
Icos-19-Ene-1,2,4-Triol	10.459	346.33121	[M+NH4]+	346.33151	57.07106:64241 58.06582:6584 68.03234:63370 70.06582:132651 85.10192:29826 88.07651:442815 89.07964:19456 102.09214:236305 103.09526:8077 132.10152:30658 146.11676:18912 150.11366:38541 240.26962:23293 258.28003:10973 284.29712:2052023 285.2999:399659 328.32281:149158 329.3237:30093 346.3306:6055714 347.33521:1212289	-0.866222077
(4E)-7-Acetoxy-6-hydroxy-2-methyl-10-oxo-3,6,7,8,9,10-hexahydro-2H-oxecin-3-yl (2E)-2-butenoate	8.704	344.16989	[M+H]+	344.17032	56.05019:9809 67.05523:12452 69.07082:15273 69.18517:6521 79.0553:2738 81.07082:11204 83.04978:5956 84.04498:204565 85.04835:3122 85.06546:3580 89.06062:28424 91.05511:22464 93.07067:9620 95.0492:3579 95.08613:3300 102.05585:251782 103.05855:7137 105.07024:17427 107.08632:20523 109.06507:3147 109.10176:31012 113.06022:10804 117.07033:5890 119.08541:8084 123.11664:6145 130.05077:233025 131.05373:10100 133.08719:22673 133.10083:201608 134.10426:16450 135.11725:12737 137.0956:15837 143.08638:13304 148.06075:245621 149.06369:7128 151.11107:124244 152.11484:9873 161.09535:94802 162.10052:6288 169.12274:7668 177.11325:2226 178.12354:3689 179.1062:294130 180.11066:25778	-1.249381411

					196.13399:27206 197.11699:35752 215.12863:16323 252.15912:6952 262.14548:9573 280.15543:105945 281.15915:13447 298.16449:25502 299.13846:2920 308.1496:11218 326.16141:75326 327.16354:14009 327.20285:7634 344.17075:104870 345.17108:12577	
N,N-Dimethyldecylamine	9.968	186.22153	[M+H]+	186.22157	53.00287:3154 55.05516:3596 55.93536:3110 57.0709:322820 58.07381:3778 69.0707:2865 70.04785:4966 70.06621:3617 71.0863:227423 72.08166:4271 72.08978:3606 83.08642:9792 84.04517:20736 84.08147:2733 85.02902:3514 85.10202:118274 86.10549:4890 88.02222:30793 88.957:2466 95.06072:26267 98.06049:9792 102.05582:2726 112.07623:14297 113.07082:11839 114.0556:4598 115.08724:2882 116.95271:11155 117.95958:4080 118.9672:10490 119.9753:19661 123.0557:26920 124.04041:2799 126.05476:19915 128.07063:3119 128.95076:2666 130.05072:11811 134.9624:5762 138.05554:2610 140.07124:47676 140.10721:26524 140.14322:17523 141.0645:17524 141.10193:9235 142.99178:4097 144.06485:5445 158.08012:3195 159.11249:5065 163.94035:3476 168.06599:4543 168.10329:17349 169.06021:13092 186.05626:3573 186.07552:26394 186.11185:59931 186.14764:34471 186.18631:15751 186.22243:404417 187.08588:4933 187.11014:8796 187.12714:18692 187.22433:44955	-0.214797888
2-Carboxy-4-dodecanolide	10.453	241.14342	[M-H]-	241.14398	57.03291:9795 68.35703:5973 68.36132:6762 71.04817:2415 80.02505:3382 83.04874:1598 96.83704:2212 96.84337:2662 106.03918:1965 111.08068:2493 150.91393:9583 161.1324:1959 165.09151:5405 168.92523:2088 171.06731:2196 177.12733:3380 179.14317:394850 180.14555:41116 181.15901:2914 184.00185:5641 184.50235:2037 194.90565:3022 195.13814:5880 197.11534:2266 197.15465:27276 198.15887:2011 205.12332:2236 212.91452:3203 214.07132:1726	-2.322264068

					223.13339:264819 224.13588:36802 225.14803:7923 241.14363:1467179 241.57404:3504 242.06622:2392 242.1487:170926 242.177:29843 243.15938:2246	
6-Ethoxy-2-mercaptobenzothiazole	10.354	212.02019	[M+H-H2O]+	212.01988	69.48466:25926 113.09587:6733 135.0144:11250 136.02145:81709 160.02238:9832 165.97795:306778 166.98425:21559 167.99373:3390781 168.99614:197906 194.00966:3376746 195.01317:275973 207.00429:19473 212.02:5455200 213.02209:404326 213.12212:7996	1.462127042
3-Amino-2,2-dimethylpropanoic acid	9.061	118.08672	[M+NH4]+	118.08626	55.05517:407880 58.06589:1782372 59.0738:2626367 61.52355:43693 70.84187:66060 72.0817:4044108 73.0851:271414 88.91087:46339 91.05434:43765 118.08727:12194820 119.09142:417391	3.980141297
Berberine	8.905	336.12335	[M]+	336.12399	69.86249:11363 185.04939:2960 208.06761:2780 249.00653:3017 272.02911:3608 272.81378:2727 275.09299:10629 276.09976:2868 278.08142:10525 290.03806:4568 291.09027:6010 292.09604:303268 293.09781:42463 304.0961:64602 305.09961:10990 306.0777:105093 307.08011:23903 320.09085:363098 321.09811:819008 322.1044:128846 336.12125:3253674 337.12845:569460	-1.904059273
Arachidonic acid	12.694	305.24683	[M+H]+	305.24747	55.05524:64030 57.07088:112372 67.05531:117925 69.07075:119980 69.68121:16636 71.04986:18007 71.08629:78850 79.05463:132383 81.07035:171330 83.04979:19227 83.08649:81644 85.06546:15362 85.10194:26293 87.0444:8362 89.06052:9616 91.05512:137509 92.05798:6690 93.07074:259423 94.07333:5309 95.08618:118502 97.10159:31386 99.04497:6793 101.05991:16693 105.07013:255783 106.0731:7631 107.0864:143408 109.06507:23013 109.1018:57407 111.08025:5187 117.07038:61443 119.08553:307702 120.08881:21313 121.06449:19134 121.10117:162026 122.10457:5550 123.11664:25597 127.07574:9396 129.07033:41586 131.08513:123410 132.08858:9673	-2.096659474

					133.10085:270431 134.10402:24500 135.07993:25373 135.11722:72398 137.13249:16211 143.08601:51796 145.10216:42745 147.0798:65749 147.11702:202197 148.12051:20442 149.09651:69209 149.13277:18440 151.14795:7984 153.09102:15152 157.10077:64998 159.11627:16322 161.09535:170696 161.132:59864 162.10054:8452 163.14888:24028 165.09114:23835 165.16359:4971 167.10715:24028 171.11742:65741 172.11986:7096 173.13095:6855 175.11177:196129 175.14688:25677 176.11598:7099 177.1649:34262 179.10611:127456 180.11058:9393 181.12352:8694 185.13301:25723 189.12665:63383 189.16347:9093 190.13196:5111 191.18057:30699 193.1232:204176 194.12497:19363 199.14815:9031 203.14417:9964 203.1797:35229 205.19437:23072 207.13722:115542 208.14168:8421 221.15309:76042 235.17052:21314 243.21054:25911 245.2265:79572 246.22765:9302 249.18556:6326 259.2037:22932 259.24329:14038 269.22614:38546 287.23807:113609 288.24289:18122 305.15561:194522 305.24634:566699 306.15875:25645 306.24979:97520	
1,2-Cyclohexanedicarboxylic acid, 4-methyl-	8.474	185.08057	[M-H]-	185.08194	57.03292:1414 59.01237:2707 65.01311:1593 69.91403:5304 74.02287:30034 75.02641:8042 81.06924:3113 83.02363:8144 87.92345:2085 91.02864:1717 95.04829:5467 97.96792:10763 116.92694:3102 121.05012:3001 121.06402:1671 123.08006:208633 124.08304:14177 125.09486:3123 128.03317:1484 138.88843:2167 139.09358:1449 141.09087:148276 141.10985:7999 141.95786:2385 142.08627:5242 145.0506:1497 157.09718:2083 158.03552:10694 159.06706:2453 167.07071:7967 185.04552:7665 185.08133:275820 185.10043:75158 186.08362:16270 186.11412:11343	-7.402126863
Milrinone	0.888	210.06445	[M-H]-	210.06732	71.01808:6456 87.00721:8613 92.92619:868730 94.9238:881779	-

					105.23087:5176 127.86848:72347 129.86635:27513 131.08069:854597 132.08476:43229 152.88301:22988 152.99384:64454 159.85774:27610 161.85651:6941 164.8343:25281 165.8425:24536 173.10342:7180 174.0863:420463 175.09035:22109 211.00046:56483 211.03246:9584	13.66228693
beta-Alanine	1.264	90.05564	[M+H]+	90.05498	50.76519:12349 55.21202:12569 57.39978:13172 69.66302:59543 72.0451:51241 72.08166:22224 78.87629:14368 90.0557:197516	7.328856216
Orthanilic acid	1.02	156.01443	[M+NH4]+	156.011	53.94002:26511 55.01875:8992 55.05515:12163 56.05019:3644 56.96547:4483 57.07082:2808 58.06583:8741 67.05524:4735 68.05045:8142 68.98314:18408 69.03423:3969 69.07076:21365 70.06573:43953 71.04997:33168 72.0451:9345 72.08165:4894 81.07029:4266 82.01431:23768 82.06594:41640 83.04979:8811 83.07348:4705 83.08641:12660 84.04502:22127 84.08157:42439 85.02901:2680 85.06546:3839 86.06089:46356 86.09727:23068 86.97359:4029 86.99361:56560 87.0444:3092 88.95301:3390 93.07067:7745 95.08613:4899 96.04481:5117 97.02908:15181 97.06519:11767 97.10149:3506 98.06048:11122 100.02454:103932 100.03956:19511 100.07568:10011 101.02524:10870 103.02482:4102 105.00383:136081 105.95509:3105 110.00881:29475 110.06043:66227 110.0713:16241 110.0968:20364 111.04513:15097 111.05607:19842 111.08137:11143 111.09247:4511 112.03909:4418 112.07648:16359 113.03546:4675 114.05582:25559 114.06713:9010 114.0914:7763 115.05094:3215 115.08733:108566 118.03515:46346 119.03613:4406 121.10114:2589 123.0144:31952 123.04024:13518 123.96574:3149 126.95508:22420 128.01987:292201 128.05637:14801 128.07085:65619 128.0816:9353 128.10782:52878 129.02022:25627 129.03966:4770 129.10236:33481 132.01363:14414 132.95816:3146 132.99889:8282 133.03526:4947 133.95097:11676	21.98562922

					134.01274:3298 134.03041:3229 138.05577:28568 138.09108:15033 139.0392:3713 139.05008:12291 139.07497:11402 139.08585:9187 140.07114:2835 140.10732:3419 141.05106:3017 144.96587:22718 146.02979:85698 146.06696:21979 147.01373:3664 147.03064:12132 147.05106:16506 151.00882:12196 151.03519:45783 151.96014:12280 152.03658:4622 155.97514:14496 156.03043:2996 156.05075:43284 156.06555:136979 156.10258:237060 156.13766:67234 156.17447:19986 156.96445:3115 156.97565:10205 157.03535:4480 157.04842:7594 157.06148:8228 157.09695:21692 157.12125:3074 157.13431:11646 157.16982:3539	
Bakers yeast extract	6.67	275.10959	[M+Na]+	275.10721	68.60378:15579 68.60755:19996 74.00649:45407 85.02901:10451 88.03966:73373 89.04777:8047 112.02222:8233 120.01145:60911 126.01213:53466 152.99081:75530 156.01193:27191 161.52464:28135 182.0262:27910 183.03474:120842 184.52769:27264 210.01973:30341 217.10487:317112 218.10939:17702 227.00604:23973 275.11041:5415563 276.11288:517919 276.17819:21551	8.651172756
Plantagonine	7.272	178.08658	[M+H]+	178.08681	69.13716:14722 69.14098:11282 88.02226:18939 115.05441:36085 117.05711:9139 132.08128:138364 133.06436:20773 133.08475:8996 134.05984:6828 142.06543:18952 143.07449:13660 146.05983:20860 147.06416:60086 148.07607:8076 150.09254:6050 155.9753:38918 159.06868:17369 160.07616:4269610 161.07982:371269 178.0874:1052389 178.12128:20319 178.15857:31842 179.06291:43046 179.09268:72016 179.11746:18288	-1.291504969
Glutamic acid, 1-[5'-hydroxy-2,6'-dimethyl-	8.243	382.22031	[M+2H]2+	382.22238	55.05515:5295 59.05006:11738 61.02904:6103 69.03426:309814 69.85862:10977 70.03783:5875 71.04985:5968 73.02938:67475 73.06559:31701 74.03223:3448 78.03988:4167 80.05531:14113	-5.415695439

5-(1-methylethenyl) spiro[cyclopentane-1,3'-[7]oxabicyclo[4.1.0]heptan]-2'-yl] ester					81.03429:12824 81.07028:4901 83.0498:66109 85.02901:10091 87.04441:310986 88.04811:9178 89.06052:979687 90.06379:43897 95.0501:17384 99.04495:150286 100.0532:45374 101.05991:4129 102.06754:15140 103.03969:4604 107.07057:33986 109.06506:5188 111.04468:132195 112.04808:5226 113.06054:31128 117.09195:9947 122.06488:85096 122.56655:8255 125.05942:26327 129.05493:52424 130.08725:30654 131.07071:46552 133.08614:403415 134.0892:30079 137.0594:13863 144.0779:60982 144.5795:5992 151.09662:24445 153.08383:11108 155.06996:93441 155.09927:10138 156.07486:5867 157.08762:4968 164.59134:3941 166.09151:40365 173.08119:30788 174.08333:3424 175.09662:39381 177.1131:92671 178.11676:5197 185.10912:3765 186.60381:7846 188.10445:15351 195.1241:13821 197.108:24460 199.09698:23878 217.10774:15509 219.12405:32662 221.1375:11350 239.1468:9008 241.13737:13096 243.12399:19561 261.1348:19912 263.15067:4657 283.17566:17360 287.15063:11549 305.15912:22188 327.20282:16667 349.18417:4733 382.1893:25472 382.6875:15459 383.33432:19323	
1-{[(3-Cyano-6,7-Dihydro-5H-Cyclopenta[B]Pyridin-2-Yl)Sulfanyl]Acetyl}Proline	7.705	330.09491	[M-H]-	330.09183	68.60859:7891 96.84338:2384 101.10535:1406 103.91844:3256 129.05469:179504 130.05745:3260 134.05969:55058 141.91135:1587 150.64879:1297 165.04326:1904 178.0499:299436 179.05293:14992 192.74565:1555 200.03154:20555 218.02881:2582 242.52771:1626 249.98149:1673 262.03217:32039 263.03702:1664 330.09692:40837 331.10001:2257	9.330736844
Sphingosine 1-phosphate	11.415	380.25586	[M+H]+	380.25598	55.05515:10098 56.05019:6564 57.0709:27306 67.05531:44692 69.0707:21911 69.63103:8685 70.06578:13594 71.08629:13512	-0.315576891

					79.05476:42742 80.05872:2560 81.07037:50855 82.06596:247365 83.06916:8592 83.08657:16375 85.10193:9368 86.09714:6310 91.05514:3840 93.07073:98109 94.07419:2994 95.07381:2469 95.08624:46050 96.0815:45944 97.10162:13336 98.98515:2754 107.08641:41587 109.10178:24138 110.09641:9933 111.1169:2835 121.1012:30051 123.11663:7285 135.11703:32123 140.0114:7164 149.13284:15796 151.14793:2416 165.16359:2403 247.24191:34426 248.24437:2964 262.25476:36969 263.25595:2754 264.26764:1974354 265.27271:325080 282.28015:7229 362.24332:25745 363.25342:2380 380.24966:6695 380.34811:9456 381.35614:5705	
Retinol	11.913	269.22607	[M+H-H2O]+	269.22601	55.0551:20049 57.07087:29028 67.05531:31688 69.07078:85345 70.07399:2654 71.08625:14159 79.05471:28423 81.07038:154390 82.07365:4362 83.08643:23817 85.10194:8598 91.05482:14775 92.05798:2249 93.07074:202943 94.07333:10021 95.05009:3243 95.08623:167094 96.08937:15472 97.0652:2449 97.10149:10265 105.07013:42794 107.08644:117554 108.08865:3339 109.10187:67177 111.11691:4165 117.06913:2959 119.08547:96143 120.08881:7324 121.1012:88298 122.10458:4172 123.11674:24649 131.08644:18851 133.10088:60546 134.10402:4344 135.11731:31138 136.06203:28386 136.12074:3046 137.06674:21431 137.13274:14602 139.00343:7228 143.08586:22734 145.1019:45177 146.10506:2339 147.11703:38554 148.12051:2675 149.13242:13907 157.01295:4271 157.10075:59943 158.10649:4266 159.1165:41845 160.12038:3279 161.13219:22277 163.14888:8870 170.10953:2498 171.11746:32428 173.13313:34082 174.13564:3263 175.14906:49571 176.15146:2114 177.16451:26541 178.16638:2302 185.13307:28775 186.13811:2262 187.149:38615	0.222861082

					188.1534:8701 189.16357:45416 190.00026:7246 190.16678:2891 199.14798:62077 200.15414:12748 201.16267:16620 211.15005:2458 213.16364:87331 214.17088:27622 215.17896:2851 226.17145:3062 227.17834:18014 241.19423:3024 246.23499:3219 251.20107:10955 254.20319:3094 269.22562:398150 270.22916:77960 270.31342:19517	
Pheniramine maleate salt	6.193	357.17743	[M+H]+	357.18088	68.0503:19672 69.36891:15081 70.06619:471732 71.06924:16104 84.0449:4763 84.08145:3601 86.06079:228114 87.06442:4278 116.07123:4886 127.08667:136411 128.08984:9154 129.10223:3152 141.1019:4084 143.08104:169464 144.0845:8717 155.08105:179509 156.08595:5498 171.07736:205755 172.07916:9242 173.09206:9830 183.0766:5220 187.10777:1276454 188.11218:84195 198.12326:4405 216.00757:2911 226.11981:12645 227.10374:16248 244.1304:261775 245.13208:26025 268.13074:36748 269.13339:3657 286.13797:17456 311.17313:11967 339.1633:14500 357.17819:475216 358.17966:72511	-9.658971667
Eicosanedioic acid	12.806	341.26874	[M-H]-	341.26974	69.45775:3049 69.46104:3248 75.00668:9338 172.05836:1716 195.85626:1628 223.48474:1682 223.51642:1587 243.22414:1496 279.26816:375218 280.27121:30158 283.26315:2154 297.27905:12415 298.28159:2098 323.25943:279283 324.25946:25713 341.27081:1000538 342.2717:103822	-2.930233428
7,7-dimethyl-1-{[(4-pyridylamino)sulfonyl]methyl}bicyclo[2.2.1]heptan-2-one	0.947	307.11487	[M-H]-	307.11221	58.02831:15075 59.01211:33778 66.03323:2546 69.3404:7278 70.02782:6249 71.01237:16833 74.02267:2736 78.95698:6904 82.02834:95055 84.04401:111205 87.00733:202697 88.03857:33277 89.02311:28935 92.92614:24242 94.92374:144915 96.92042:54688 96.95861:32268 96.96822:28043 98.05981:19533 100.03875:7363 101.02259:50630 101.07063:5780 102.05399:7651 104.03407:3425 112.03845:17298 113.07027:42122 115.08544:3121 118.05019:3596	8.661329356

					119.03326:14107 125.03382:3558 125.071:33069 126.05444:10638 127.04957:10045 128.03329:31646 130.049:28807 130.06024:25644 136.91635:3540 143.04524:6729 143.08102:107378 144.06482:7898 144.0845:3298 145.06061:207689 145.09698:8792 145.99803:28439 146.04489:97818 146.06323:3912 149.07065:3066 151.04941:4300 154.92377:25851 167.08064:12850 169.06105:12951 170.00908:9637 170.04489:95635 176.89949:3849 180.08615:239507 181.08965:8097 187.07216:825640 188.02058:58493 188.05647:17502 188.07359:33533 190.01616:3444 200.05656:16236 217.08209:34197 218.06497:15756 229.08228:2580 307.11676:29442 308.09772:13705	
3-Methoxytyrosine	6.258	212.09206	[M+H]+	212.09178	68.60216:8369 68.60647:13475 70.0657:23909 82.06519:3592 84.04491:9991 84.08146:11435 88.07633:8986 91.05431:3717 93.03407:9227 95.05008:3193 96.0564:2698 98.06048:3091 100.02158:5103 100.03961:3535 106.06585:11829 111.04473:13330 116.07124:3112 119.04966:17578 121.06488:17334 125.05956:59955 125.07004:6309 126.06422:2539 134.06009:26919 136.07556:2957 149.06023:420559 150.06287:32919 151.07571:10107 153.05508:522086 154.05826:32993 154.08571:2764 163.03825:2806 166.08736:423319 167.09137:34346 167.11739:4183 171.11287:15957 177.0547:32469 185.12823:2852 194.08136:12310 195.0645:324206 196.06912:31385 212.07545:238795 212.12827:11093 212.16344:3254 212.96584:3177 213.08005:16950 213.12448:29419 213.16052:5507	1.320183177
Butyrylcarnitine	6.478	232.15446	[M+H]+	232.15428	57.03444:9746 60.08165:592448 69.48412:32191 71.04984:65500 85.02905:5818215 86.03275:182577 144.10254:125419 163.97617:13674 164.97855:7964 173.08138:1291753 174.08359:89263 208.95573:8566 232.15579:2782246 233.15895:333210	0.775346464

2-(3,4-dihydroxyoxolan-2-yl)-2-hydroxyethyl (9E)-octadec-9-enoate	13.231	429.31921	[M+H]+	429.32108	57.07082:8896 65.74067:1426 69.07069:1622 71.03111:1508 71.08622:6896 81.07029:1644 83.0864:2666 85.10193:2389 86.09714:2819 87.0444:3257 114.06135:1550 128.38458:1584 149.0965:6382 163.11195:12359 164.08383:2075 165.09129:57149 166.09557:2124 177.0909:1788 184.07404:14802 191.10789:2025 205.12189:6453 257.07953:1587 395.25806:1629 429.32269:2192666 430.32028:346178	-4.355714376
2,6-Di-tert-butyl-4-nitrophenol	12.704	250.1442	[M-H]-	250.14482	52.98746:1755 65.01308:1930 70.03558:2593 78.95695:1879 106.03915:2240 109.0276:2772 111.11729:1708 131.03351:1719 150.02982:1810 164.53157:1844 179.10603:14426 196.06174:2440 201.81737:1586 207.10168:21273 223.09857:2991 224.05612:2979 234.11617:2127 235.12166:2133 250.14268:4542070 251.0923:14175 251.14757:586404	-2.478564217
5-Methylcytosine	6.176	126.06634	[M+H]+	126.06618	53.03948:29488 55.01876:34506 56.0502:14577 56.96548:9207 69.04567:15901 69.58312:37178 70.06565:17863 71.04987:37086 80.04992:68367 81.03451:69621 81.04539:13548 82.0659:10244 83.06129:31149 84.04492:12415 84.08147:50793 84.96075:242993 95.02465:67376 96.00912:9770 98.06049:39626 99.04498:38391 99.05527:9716 102.97031:31647 108.04517:257608 109.02875:110746 109.04037:285566 119.11625:7574 126.05485:256110 126.06691:1525049 126.09109:39427 127.03923:1305063 127.07008:67139 127.12332:8033	1.269174651
FT-PFSA; C4H5F5O3S	1.41	226.97736	[2M-H]-	226.98068	68.70409:7090 74.02268:2117 78.95699:1955 89.02288:7067 96.84338:1636 96.95827:2559 97.92987:6209 99.92418:153790 112.98355:6522 115.91898:13652 116.03381:12065 116.92693:1436 127.08626:6287 127.95489:1502 130.04901:1414 146.9023:2224 152.91708:7739 155.08119:9189 155.95111:10659 157.96542:2615 165.10106:9596 166.99495:1409 169.01065:1989 171.98145:37252 173.96191:73924	-14.62679555

					180.91151:42086 183.96635:8809 198.92303:2251 208.96878:7818 226.80083:2854 226.96613:3716 227.10291:22318 227.97183:46097 228.16103:3025	
LPC(14:0)	11.53	490.29132	[M+Na]+	490.29041	57.07077:625635 58.06666:329770 60.08155:2648910 69.07068:121195 71.0737:374715 71.08614:589290 81.07027:108734 85.10191:260943 86.0971:8405111 87.10064:213208 95.08611:314830 104.10735:43565520 105.1077:207647 105.11076:1427593 109.10179:77248 123.11662:80482 124.99965:2732218 163.01651:244882 181.02643:93965 184.07436:53402120 185.07791:1538177 211.20531:126113 225.82339:109042 258.1105:790697 285.24039:1977889 286.2482:215510 391.22717:360675 450.30109:3697281 451.298:522699 468.30594:13263117 469.31622:1791143	2.412939488
Glycocholate	9.272	464.30029	[M-H]-	464.30121	52.50174:1714 69.14695:6084 69.15076:1832 74.02341:176656 74.04624:1438 81.26203:1430 85.57569:1528 95.46712:1548 125.55259:1340 135.68184:1421 182.74892:1562 344.99026:1977 394.85767:1660 398.07718:1574 402.29874:6120 418.2963:2375 420.30801:12141 421.31473:2521 464.29865:954279 465.30609:133132	-1.981472329
2-Aminoisobutyric acid	0.869	102.05437	[M-H]-	102.05602	51.08323:2376 51.14527:2237 57.03291:4089 57.17451:2032 59.01236:3305 70.83395:2873 74.02267:2967 74.95987:4772 83.19536:2426 101.03979:2132 102.01781:5028 102.054:133147 102.9556:3495 103.03788:8961 103.05772:2561	-16.16759109
Chenodeoxyglycocholic acid	10.172	450.3212	[M+H]+	450.32138	55.05505:179605 67.05518:303597 69.07059:147787 69.25065:49835 76.03978:3908911 77.0432:53596 79.05518:230632 81.07091:1024192 83.04971:44480 83.08633:286189 85.06544:501780 89.06045:41302 91.05479:204520 93.07059:629972 95.08605:996416 97.06511:52903 97.1014:72471 99.08042:26028 105.06992:511829 107.08625:1084033	2.944726115

					108.08961:66568 109.06498:59474 109.10172:822073 111.08016:65615 111.11792:44594 112.07578:101708 114.05548:46091 117.07023:69343 119.0853:712525 121.10104:913648 122.10446:69450 123.08016:86125 123.11655:282505 125.09659:193165 129.07014:23729 131.08498:272746 133.10068:894096 134.1039:60781 135.07982:62945 135.11708:1073474 136.12057:81017 137.09596:278488 138.09233:68240 139.11218:47835 143.08569:132300 145.10178:615431 147.11682:1264592 148.12068:102316 149.09657:155185 149.13271:1115273 150.13582:95456 151.1127:276812 152.10759:55390 155.08632:53865 156.06546:182315 157.10065:199526 158.08182:2059984 159.08408:81756 159.11623:848993 160.12024:64269 161.09703:64344 161.13203:1378990 162.13606:101906 163.11128:646036 163.14882:483823 164.1156:43442 165.12723:63735 166.12183:66986 169.10164:115922 171.11743:205445 172.09631:268087 172.11972:24639 173.13297:452376 174.13548:48735 175.11183:307529 175.14893:1485152 176.15149:132371 177.12659:835921 177.16402:153066 178.13014:73503 179.14232:41547 183.11681:120045 184.09827:148510 185.13303:511126 186.11192:93697 186.13553:62139 187.14883:640221 188.15079:65199 189.12674:267996 189.16339:962059 190.13181:39419 190.16666:93085 191.14287:277591 197.13228:150918 198.11282:146394 199.14812:444114 200.12711:62917 200.15128:40150 201.16492:2476545 202.16721:289904 203.14415:312905 203.17966:428305 204.18321:41700 205.15768:113929 209.13329:43555 210.11243:84945 211.14703:586470 212.12808:369387 212.15154:67626 213.1635:557181 214.16743:60047 215.14285:99814 215.17885:2172826 216.18239:264470 217.15959:197489 217.19461:158764 219.17316:43549 224.127:179099 225.16493:333785 226.14221:223203 226.16801:54687 227.17793:384297 228.18475:49876 229.15939:516863	

					229.19455:416792 230.19867:63768 231.17549:237760 238.14331:561892 239.17822:340623 240.15999:116535 241.19434:292244 243.17436:292480 243.21033:482737 244.21346:53271 245.19052:122284 250.14366:160203 251.1778:79741 252.15926:457771 253.16064:47548 253.19504:72089 254.19913:48076 255.20924:82790 257.18909:367123 258.19324:54961 264.15784:212888 265.194:192792 266.17645:220369 269.19183:43730 271.20532:49027 278.17349:200728 279.21014:77233 280.19049:94826 281.22586:26938 283.20474:86304 292.19156:172639 293.22607:42851 295.24185:128167 297.22394:73887 297.25797:269124 298.26181:21550 304.19183:266430 305.19547:42827 306.20441:160482 311.23538:67566 311.27182:90016 318.20377:177423 320.22122:102978 321.25635:2190874 322.2637:433680 332.22198:219517 333.22913:25857 334.23523:40978 339.26971:3395506 340.27356:669483 358.2366:57041 368.29544:52491 396.28928:140971 414.2981:6869666 415.29959:1406157	
PE(18:2/18:0)	12.3	742.53882	[M-H]-	742.5387	69.88243:3163 78.95742:22352 96.84156:2821 121.99924:2536 140.01012:28163 168.03957:4598 196.03642:19525 224.06892:3680 253.21762:3353 255.23199:54039 256.23672:3427 279.23273:785591 280.23566:76450 281.24847:233335 282.2533:25856 283.26331:233635 284.26654:15497 458.26654:2245 462.30322:2621 478.28976:3907 480.30923:22689 742.53979:164694 743.53894:35418	0.161607738
4'-Chloro-N-methylformanilide	1.401	170.03297	[M+H]+	170.03668	55.05516:8737 65.062:6005 69.30322:38947 70.06565:9073 71.04987:10223 72.04507:46645 83.06129:9781 83.08642:13899 84.04565:8865 85.06548:14092 86.06057:68260 89.07172:63900 91.78134:6674 96.06892:33364 97.0761:7244 100.9194:6132 105.45519:5934 109.07711:60015 117.83938:6676 124.07579:31758 124.08759:179299 125.07138:6123 126.10318:14496 128.07062:9868 142.08633:10529	-21.81882168

					142.12329:7565 152.03134:307374 152.05025:62364 153.03537:37546 169.9854:254457 170.04218:138063 170.0885:54308 170.09271:146138 170.11763:99859 170.15373:9552 171.14944:119593	
Nb-methylusambarensine	8.432	448.25922	[M+Na]2+	448.26218	67.76279:35644 69.03424:159058 70.03784:6051 73.02943:42319 73.06564:22766 80.05533:14279 81.0343:9952 83.04983:47945 85.02901:4711 87.0444:201278 89.06052:705348 90.06357:22260 95.05009:14798 99.04496:102364 100.0531:27211 102.06852:16303 107.07061:16645 111.04465:78635 113.06047:19957 117.09196:13032 122.06487:44775 122.56785:4301 125.06076:12705 129.05493:40257 130.0872:23080 131.07059:31542 133.08609:343064 134.08931:19549 137.05942:15100 144.07829:19621 151.09686:5316 153.08385:5269 155.06989:45994 155.09929:4490 157.08575:5548 166.09152:23178 173.08128:16733 175.09677:18159 177.11296:89521 178.11678:4629 188.10446:14559 195.12411:15021 197.11154:10377 199.09752:17146 199.12416:4104 210.1179:4034 217.10776:4759 219.12411:17567 221.14062:11689 239.15034:6291 241.13739:5866 243.12424:13726 261.134:11002 263.14664:5085 283.1774:12445 287.15067:4772 305.16034:9832 307.17233:5683 327.20285:10240 371.22916:5153 415.25949:3360 448.23001:40781 448.72638:19843	- 6.603278465
Trans-Hexadec-2-Enoyl Carnitine	10.873	398.32693	[M+H]+	398.32703	55.05515:10518 57.03439:14351 57.07084:20353 60.04541:6031 60.08159:175616 67.05511:15482 69.07059:20134 70.03616:4211 71.08633:17712 73.02941:7715 79.05529:2866 81.07088:27813 83.08641:29495 85.02898:1361561 85.10193:7581 86.03265:35224 86.09707:11475 88.07632:6085 89.06052:7601 93.07066:10510 95.08604:26832 97.06519:5819 97.10148:16484 99.08143:2356 107.08636:8527 109.10165:15834 111.08135:3416 111.1169:6907	- 0.251049998

					121.10107:20921 123.11793:7293 125.09657:3247 126.05482:2022 133.0862:2393 135.1171:22021 137.13248:6915 139.11229:3139 144.10239:40800 147.1169:2724 149.13254:11843 153.12874:3159 157.05026:7860 161.13206:2498 163.14886:7504 167.14197:2338 175.14687:2204 177.16467:3145 179.99141:2391 184.07443:3683 195.17322:2475 198.00165:3477 219.21048:19658 225.99733:10381 227.12608:2165 237.22183:25330 238.22351:2221 241.14447:8745 244.00746:2411 249.01402:2475 255.15913:7625 269.17529:10864 283.19235:11428 297.20474:3056 311.18875:3647 321.20721:2272 336.32578:8325 339.25262:60739 340.25595:8820 390.26025:2318 398.32373:798019 399.32907:166331	
1-Naphthalenecarboxylic acid, 1,2,3,4,4a,5,8,8a-octahydro-1,4a,6-trimethyl-5-[(2,3,5-trihydroxy-4-methylene-7-oxabicyclo[4.1.0]hept-1-yl)methyl]-	9.839	437.21533	[M-H]-	437.21811	69.65415:6197 78.95763:8313 111.0052:10137 125.09615:9840 151.11102:3577 152.99414:33770 167.10754:1647 169.0856:322803 170.08868:15586 177.09175:11489 191.06874:478868 192.07207:18502 195.10179:159848 196.10603:8813 209.06516:15833 217.08232:175694 218.08624:10600 233.06651:1913 235.08066:2350 237.06216:42007 238.06232:3048 289.18396:16799 307.18784:3139 315.1998:2197 325.20517:10083 329.1713:2970 333.207:2085 369.22577:16711 393.18881:9297 437.20267:30633	-6.358382547
Robustic acid	11.931	379.12079	[M-H]-	379.11871	59.01237:2110 78.95764:1941 84.93733:6158 87.92344:2552 96.8405:14349 96.95827:3300 99.92408:283881 100.92431:12093 100.93201:28076	5.486408202

					103.91866:17634 104.92642:6828 106.94178:2634 114.9474:6785 115.91897:137746 116.92688:256549 117.92742:12101 130.94255:9169 133.10201:1698 140.96368:2034 166.97858:3075 180.99493:7120 199.00554:3158 225.96732:2308 227.03748:2242 233.02608:8403 235.0435:12449 261.02155:1816 267.03088:7025 311.16821:13522 311.2204:5749 317.1568:5832 331.09998:2713 333.11508:14796 335.13065:118466 336.1322:11929 343.10187:3288 349.10992:20059 361.10995:42474 362.11496:9878 365.10678:2680 367.11993:11936 375.12766:9666 379.15659:266204 379.24762:9242 380.15988:46320	
9-methyl-8-{[3-(trifluoromethyl)phenyl]methylthio}hydropurin-6-one	6.675	339.05682	[M-H]-	339.05301	51.98779:1327 58.44847:1605 70.3625:1777 72.00746:2168 102.94769:1639 103.91844:3396 106.60484:1608 108.91328:2763 112.9836:38174 116.04961:10723 117.20083:1335 122.89236:8869 123.8999:7791 130.06448:1823 135.92351:2291 141.91158:15304 142.06378:1743 146.93777:6677 152.49649:1680 158.92445:6597 159.09155:10417 161.90346:44934 163.91872:23229 203.08212:224473 204.08562:16919 221.97777:7359 225.0645:151019 226.06749:11267 234.04494:1421 264.40991:1478 269.05368:1674 271.06979:120106 272.07162:7463 292.97879:125140 293.04852:22380 293.97971:13462 294.05692:1651 310.99081:30117 327.13046:1539 338.98627:2072	11.23718088
Tetrahydroharmol	10.321	203.12106	[M+H-H2O]+	203.11844	59.99109:56783 60.04541:4511 69.2529:19066 72.08165:7191 86.00632:139491 87.00976:5449 88.02232:513498 89.02546:9019 99.04498:4126 112.07622:5174 116.05351:1762557 117.05646:57217 126.05483:4690 130.0325:2221251 131.03586:87358 143.08586:5073 203.08371:5572 203.12762:53226 204.13913:38995	12.89887811
10-(4-Sulfophenyl)de	13.36	327.12729	[M-H]-	327.12711	61.50411:1465 69.62386:2584 69.62716:2001 84.93734:2221 88.0385:1443 92.67924:1538 96.84067:2173 99.9242:78007 100.92528:2739	0.550244827

canoic acid					100.93201:10433 106.03919:1822 115.91904:53338 116.92708:178029 117.92743:10658 132.7256:1409 194.97279:2099 218.84328:1388 238.04845:1488 241.17926:1756 253.25204:2523 281.2796:1769 283.13663:8408 284.13788:1703 297.11728:3422 299.25702:11678 315.12677:16508 327.12482:243308 327.18097:103737 327.28925:21645 328.12695:30874 328.18268:9485	
(5Z,8R,9S,11R,13E,15S,19R)-9,11,15,19-Tetrahydroxy-5,13-prostadienoic acid	9.825	393.22491	[M+Na]+	393.22473	52.14426:1859 64.27826:1728 69.17754:10083 71.08509:2836 81.06892:1786 82.34583:2279 95.08526:2593 111.30155:1915 138.12633:2249 149.02229:3825 150.02632:2049 155.00774:11655 193.08261:3572 194.13269:1721 199.07356:1655 211.08221:14229 212.08142:2281 229.08994:11203 257.11084:2033 285.18265:13361 303.19437:56870 304.19699:3341 331.18613:74086 332.19342:9750 339.1929:39405 340.1965:7479 349.19751:17677 350.20135:2408 357.20419:242878 358.20511:39699 375.21567:484747 376.2168:92630 393.22363:320607 394.22928:53069	0.457753509
Phosphoric acid	0.895	96.96793	[M-H]-	96.96962	69.26871:9129 69.27254:12618 78.95764:4215552 79.95584:97506 96.92027:518408 96.95829:3493378 96.96793:868392 97.00255:83714 98.02276:9258	-17.42813883
3-[2-(6,7-dihydroxy-1,2,4a-trimethylspiro[3,4,6,7,8,8a-hexahydro-2H-naphthalene-5,2'-oxirane]-1-	8.362	384.2359	[M+2H]2+	384.23801	59.05007:3543 62.26569:1654 71.02261:2323 103.66652:1804 210.23642:2025 215.17899:1648 263.11429:1859 335.16473:2377 367.21219:1248360 368.21533:208091	-4.901807403

yl)ethyl]-2-hydroxy-2H-furan-5-one						
Lauroylcarnitine	10.205	344.2789	[M+H]+	344.27948	57.03444:8834 57.07078:21028 58.06583:4225 60.08163:251185 67.05524:2558 69.31907:12533 71.08621:14235 81.07028:4006 83.0864:9711 85.02902:2093223 85.10181:14959 86.03272:68907 89.06049:29386 95.08608:19781 109.1019:24525 123.11663:3861 133.08617:20302 144.10239:54381 165.1656:2708 177.11101:2781 183.17528:67917 184.1787:3534 201.18398:8026 285.20389:173744 286.21213:23782 327.20282:12660 344.27972:1134335 345.28159:220445	-1.684677809
Hydroxyproline	1.276	132.06569	[M+H]+	132.06548	58.06595:101755 68.05047:2585376 69.05384:81284 69.54404:54200 70.06619:21635 74.02438:48920 84.04491:26715 86.06084:8347895 86.09714:64484 87.06438:325723 114.05573:212007 132.06561:2731513 133.06891:113812	1.590120295
Ncgc00385798-01_C31H43N7O6_	12.26	608.31891	[M-H]-	608.32019	51.53184:1462 59.0498:1450 66.49675:1824 68.99397:2699 72.62983:1439 79.95517:1544 89.0229:38985 96.83796:8680 112.98357:439560 113.02115:2960 113.98798:5834 114.018:2144 114.15322:1411 201.35597:1662 226.00922:2072 255.23186:13268 279.2327:2904 480.31012:5253 504.30576:2496	-2.10415505
Citramalic acid	3.236	147.02846	[M-H]-	147.02992	57.03311:29785 59.01192:9753 69.84475:9361 75.13498:2106 85.02808:123366 86.03098:2973 87.00731:210096 87.92342:5071 88.01028:2795 96.84064:2790 101.02245:3364 102.94668:14739 103.03799:25101 116.7257:2163 118.96539:4343 122.37967:2103 125.90153:2039 129.01703:30396 147.02896:125618 148.03171:2719	-9.929951672
Butalbital	6.851	223.10797	[M-H2O-H]-	223.10883	66.03323:1331 69.16004:1314 69.16331:7175 94.02842:233142 95.03181:10511 100.00272:8245 110.02345:1391 123.04369:2101	-3.854621083

					123.90009:11851 125.89893:13593 128.07042:25032 139.91559:1615 176.97987:2554 176.99774:2330 188.91304:1394 201.45628:1612 211.94843:1618 223.10818:158357 223.79953:13024 224.05623:1706 224.11031:14343 224.13576:2018	
TG(18:1/18:1/22:4)	9.154	952.82434	[M+Na]+	952.8327	69.07066:4602 70.0295:22702 70.06613:274001 71.06915:13647 72.0816:329250 73.08506:13095 74.06113:55474 82.06586:5242 83.06118:25628 84.04485:397331 84.08141:585728 85.04832:11989 85.08454:31763 86.09709:659514 87.05588:58770 87.10046:39692 88.04025:14029 91.05512:32769 93.07063:4716 98.06044:4811 99.09262:5790 101.07147:239010 101.10807:14267 102.05578:140550 102.07533:15719 103.05453:19580 105.06692:101352 110.06023:5861 110.09747:15505 111.09354:8207 112.07616:5517 113.10725:3613 115.05089:11994 115.08718:6642 119.04963:16253 119.08157:25242 120.08123:654118 121.08458:53336 126.05611:14875 127.05124:5494 127.08656:6442 128.10768:13700 129.06596:40525 129.10219:681809 130.05061:36342 130.1057:41304 131.11774:14256 136.07549:275653 137.0791:24237 137.10808:4873 139.08736:13112 140.07089:23628 141.06529:14599 141.10176:49805 143.11827:4511 146.12845:11191 147.07625:7105 151.08623:3664 152.10765:3691 152.14355:22210 153.06581:17229 153.10175:12161 154.09842:28082 155.08084:62071 155.11725:36625 157.06142:12268 157.09689:3857 159.1124:5596 164.03387:6652 165.06694:4305 165.10298:54443 167.08044:17273 167.11732:12763 168.0659:5069 168.11345:5558 169.09755:55730 170.09262:4378 171.07724:24669 171.11337:16805 172.10909:16655 173.09212:21212 173.12877:170289 174.13121:12966 175.08748:4851 180.07613:4831 181.06097:51156 181.09572:12109 182.09354:34199	-8.773838261

					182.12874:15734 183.07671:50916 183.11424:35537 184.07437:14717 184.10754:11684 185.09286:31703 185.12816:12519 185.16635:21875 186.08986:6418 186.12347:48572 187.10736:22771 188.10437:15563 189.08693:5982 191.10281:15056 194.08897:8186 194.13002:5222 195.07767:35688 195.11345:26176 196.07881:3790 197.12721:12709 198.08894:39306 199.0712:52328 199.10809:14574 200.10329:23784 200.14061:40988 201.08652:13713 201.12428:122282 202.08257:30427 202.12088:5249 205.09709:22483 205.13297:13833 207.11449:38001 208.11028:6057 209.09323:13921 211.10632:11594 212.10481:48334 213.08665:36553 213.16043:14141 215.13995:7125 216.09789:164721 217.08037:12045 217.10159:16454 217.13452:60885 218.1368:5085 219.11172:4243 221.09064:5134 222.12404:19887 222.16081:17790 223.07121:5841 225.12335:11224 226.08427:4100 226.11975:7108 226.15501:91509 227.15848:5075 228.13606:23103 229.11882:29716 230.11241:51393 231.09576:5278 231.14908:4098 233.16531:17147 234.12367:24389 235.10933:26521 238.12234:5791 239.14673:11379 240.09657:9165 240.1356:57885 240.17068:4599 241.08025:58572 241.1373:6112 242.08116:5828 242.14774:22115 243.17807:8450 244.13034:3760 245.12849:44012 246.12498:13713 247.10771:20439 249.12206:5705 251.10281:19315 252.13623:12715 256.09323:30207 256.1282:4005 257.16153:13183 257.19727:24529 258.14615:6386 258.20123:4723 259.08859:35723 262.11719:12476 264.13281:22276 266.15173:20359 267.14697:16003 270.14581:41324 271.14615:3793 274.10464:13632 276.13452:45387 276.16934:27115 277.11633:5894 282.10919:3823 283.14563:79254 284.12421:6087 284.15149:7211 285.15811:4713 286.13794:5710 288.1546:54034 289.15927:6073	

					292.12924:6152 293.16431:10987 294.14551:25726 295.10684:5791 297.15543:30381 300.19119:15103 301.15063:25033 302.15417:3893 304.13208:19237 305.16525:3565 310.10336:13344 311.1366:38737 311.17303:35321 312.11752:45057 313.11874:12117 315.16702:22032 316.13043:6585 321.13068:5030 323.1376:12280 323.17065:15205 325.18567:4163 328.11398:12695 329.1423:14191 329.18195:19248 331.12775:4298 334.14267:11845 336.23792:6554 337.18723:4864 339.1691:3790 341.18036:18470 342.14468:4421 349.19025:11016 351.16656:3623 354.24997:25479 355.16354:4617 355.25244:4002 359.17166:20667 362.13306:4300 369.18204:13032 371.27646:3972 375.20187:5896 377.181:6507 379.16226:32880 381.1795:5924 382.2121:3954 385.23358:35853 385.73721:16857 386.24066:4296 386.68018:12395 387.18549:14305 392.19766:14220 398.20322:18752 410.24109:4144 412.17868:5619 416.216:6845 417.21643:5349 423.155:5119 426.20114:14366 439.20056:13088 440.1799:15912 441.16168:22443 444.21014:3967 455.22592:3843 457.28711:3410 464.29471:16112 465.28308:4221 467.70065:6749 468.19989:4697 473.23648:4931 476.21866:11375 476.71185:10905 477.20581:3915 478.2258:3858 482.31058:34863 483.31461:5150 485.21326:30174 485.71179:14130 494.22833:15600 498.23315:3925 499.28958:4602 500.28568:4373 509.2012:5821 510.19434:3561 520.28754:6556 522.24023:5195 527.21045:3919 528.19922:5521 540.2486:4252 541.76422:13282 542.75824:4245 544.29657:11138 545.2251:11238 555.23767:4531 597.33295:11677 624.20996:9846 641.24847:36077 642.25226:14383 643.24133:3723 648.39349:3480 650.29236:6735 650.40247:10428 659.25464:29824 659.86475:6634 660.26685:5038	

					706.36346:4077 710.42261:4862 717.4057:4471 719.41461:3939 730.39093:5943 737.31036:6748 754.34729:6125 755.33063:36884 756.33582:12082 769.44501:37491 770.45813:14723 772.3479:15001 789.92566:5028 798.43732:4044 806.41364:3690 847.44403:5860 884.4837:3643 903.49573:3850 936.49133:5138 937.68842:4901 943.48846:3785	
PS(17:0/20:3)	11.955	798.52808	[M-H]-	798.52899	57.03291:3004 59.01205:34651 69.26105:6569 78.95769:23786 99.07984:20155 113.09538:30515 133.10054:11814 135.11699:82534 136.11906:2934 152.99425:23302 163.14731:31010 168.04137:27047 179.10669:212044 180.11131:11164 189.16386:24843 217.1945:85979 224.06802:21728 235.20555:16741 242.08035:51317 261.18527:156441 262.1893:13142 279.19724:825167 279.23264:1629520 280.19992:69929 280.2355:136091 504.30585:586627 505.31641:79983 798.52319:6508	-1.139595445
16-Hydroxy-10-oxohexadecanoic acid	12.039	285.20685	[M-H]-	285.20657	51.49921:1482 59.01194:1515 68.57194:9479 81.45203:1565 82.03945:1428 96.84155:3000 100.16413:1468 115.91898:2066 116.92692:2241 156.11943:1760 164.80418:1810 166.56798:1567 183.11026:1699 194.97276:3155 196.97104:1870 202.83005:1595 211.15459:1510 223.20615:554069 224.20905:57082 241.21881:18129 261.84338:1719 267.1965:550240 268.19772:53105 285.20828:1380456 285.90402:2529 286.21158:146629	0.981744565
Glycodeoxycholic acid	10.18	448.30585	[M-H]-	448.30685	69.09837:9597 74.02322:562329 75.02634:6629 152.99409:3875 386.30634:23122 387.31006:2048 404.31873:10279 430.29697:3043 448.30829:1833082 449.30942:278169	-2.230615035
1-acyl-PAF	12.555	538.35016	[M+H]+	538.35034	56.05019:3814 57.07082:4635 57.16785:2629 58.06667:11992 59.05049:2583 59.0746:2727 60.08158:83429 67.05524:5515 69.07069:4103 71.07368:18441 71.08622:5332 72.08164:3960 81.07028:3981 83.0864:9516	-0.334354762

					86.09711:274026 87.10065:3691 89.06052:2463 90.9876:2737 95.08612:5802 97.10239:4172 98.98516:4790 101.07147:13152 104.10738:1328499 105.11087:36701 109.10181:5021 115.07668:2577 124.9998:86966 125.65845:2575 128.25638:2740 151.66742:2634 161.77023:3024 163.01653:3860 167.05592:2898 181.02646:9755 184.0744:1572848 185.07806:40778 258.11072:21498 264.26758:4278 301.0564:4906 337.27545:4401 355.2843:5102 359.39075:2921 511.91479:3196 520.34369:105800 521.34735:15257 538.34845:445108 539.35657:73284	
1-(Aminocarbonyl)-L-proline	1.323	159.07655	[M+H]+	159.07639	68.05044:35516 68.97008:131397 68.97388:35412 70.06617:12319108 71.04985:94916 71.06291:32464 71.06913:391052 74.02499:25407 81.04536:27808 84.0449:38375 86.06071:408629 88.07632:26416 95.06062:78738 96.04478:211272 96.84697:29156 97.07706:158734 99.04496:40505 111.05565:219607 113.07191:10356811 114.05552:2593313 114.07505:404208 114.09138:38122 115.05912:92376 115.08716:1129532 116.07114:542607 123.05563:266016 131.08218:30773 141.06534:471312 142.04961:218027 142.06863:28651 159.07623:4172756 160.08131:180689 160.09688:230084	1.005806078
Methionine sulfone	1.358	182.04868	[M+H]+	182.04819	59.01236:4721 63.96103:266098 70.11849:8572 71.01238:4532 78.98421:2444781 79.98363:9836 79.9877:18989 89.02285:5296 100.03873:3152 101.02245:5038 122.97376:2749 124.42473:2365 141.86641:11625 143.86308:22308 159.87877:5041 161.87402:13613 179.84108:21627 179.85709:8446 180.0332:232469 180.81242:35130 180.85802:16990 180.88177:2614 181.03645:3334 181.07112:14980	-5.443427733
Prostaglandin F2b	10.055	353.23276	[M-H]-	353.23279	59.01217:9773 69.03257:2220 69.27583:7921 71.01241:5413 83.04903:10398 96.95827:2891 99.07964:2810 111.07951:15400	-0.084929828

					113.09525:8074 125.05903:1969 137.09584:2456 165.12749:26079 171.1011:27096 173.13148:7004 181.12177:11225 191.14406:8475 193.12175:51705 193.15964:17696 194.12604:1931 209.1176:27908 211.16917:3034 217.12476:8393 219.17526:3380 221.11766:7985 229.19414:3006 235.13174:13652 237.18689:1790 247.20815:34147 248.21033:2882 255.21278:8690 263.20178:11447 265.21918:6386 273.19022:4084 273.22446:15179 281.21249:7597 291.19815:56894 292.20053:3097 299.20218:12457 309.20944:125737 310.21283:12460 317.20996:21876 335.22382:31718 336.2272:2390 353.23645:499410 354.23959:48527	
PC(16:0/20:4)	12.496	840.57324	[M-H]-	840.57379	59.01218:11946 64.32162:1906 69.03256:7379 71.0283:1953 78.95764:3823 83.02377:47759 96.06532:2274 107.085:12605 133.10056:2256 135.11586:20656 139.11119:14639 152.99422:3115 153.12712:7255 157.09903:1893 163.11186:27414 164.11607:2189 168.04164:2066 179.10689:171201 180.11134:11131 181.12201:2142 203.17859:13701 207.10207:22512 208.1087:17558 224.06897:18788 240.09941:4091 242.08052:3202 257.22736:68579 258.22836:3791 269.25049:2018 275.23566:8875 281.2482:199333 282.25354:16748 283.20889:2135 291.23181:2422 301.21521:113450 302.21915:6753 303.23364:15777 305.24649:3071 319.22925:743831 320.23236:68923 331.26004:2765 506.32083:135535 507.32803:12937 605.14478:2083 634.31134:2127 780.5752:13651	-0.654314953
LPC(18:0)	13.451	524.37073	[M+H]+	524.37109	55.05513:6536 57.07082:52438 58.06623:12724 60.0815:45918 67.05522:6840 69.07068:10733 69.093:10012 69.09682:5937 71.07372:33737 71.08627:50827 81.07086:27018 83.08637:20256 85.10201:39251 86.0971:506584 87.10063:12572 89.06049:4905	0.896856013

					95.08617:35197 97.10236:16669 104.10735:2641494 105.11066:69322 109.10203:22896 123.1166:13163 137.13246:5300 146.98152:612371 164.99257:14175 176.99254:24904 184.0744:6836 188.00902:17837 341.30563:316003 342.30692:40180 443.25403:43343 487.27884:1830221 488.28036:258392 546.34979:737632 547.35547:134561 547.47797:13728	
Derwentioside B	10.319	467.15836	[M+H]+	467.15479	57.07096:39277 67.05472:9669 69.07069:7220 71.08634:47310 81.07047:27114 83.0864:8586 85.10198:27999 95.0863:35692 97.10148:3299 109.10181:8221 116.05344:11908 130.03226:49578 137.13248:3399 140.94341:38137 141.10202:42230 155.14391:18163 195.01814:10035 201.10551:10702 205.04111:7708 221.03461:10787 223.04938:241859 224.05406:9787 239.23802:3596 246.06813:25974 263.02802:23701 265.04327:410720 266.04517:25093 281.03879:67199 283.05533:946863 284.05634:74138 297.06873:8993 304.05609:8406 306.06729:235991 307.07465:17920 467.15439:991546 467.41254:78512 468.16104:147711 468.4115:9164	7.642006625
Inosine	1.47	269.08704	[M-H]-	269.08807	59.01195:6125 69.68561:2408 71.01241:2051 89.02289:379007 90.02629:7973 96.95827:7318 103.91844:2569 122.89366:2795 125.8721:1822 133.01323:1963 150.90515:3238 154.89404:13670 156.91432:6413 157.00517:3205 157.01077:16108 168.88986:1641 178.91972:26193 179.92123:2579 181.05733:2018 200.92038:1706 201.0365:62049 202.04062:2082 207.03871:9931 225.04875:3188 231.96698:6691 268.83197:3169 268.95743:1856 269.07462:2308 269.21283:7199 269.83392:6215 269.95834:20422	-3.827743088
3-Ureidopropionic acid	1.02	177.02469	[M+H-H2O]+	177.02499	55.01874:4236 55.93548:52212 56.96546:3906 68.98311:11654 69.03422:3164 70.95848:2994 71.02258:2343 72.93774:7112 73.06573:15910 73.9453:2715 74.95358:3110 84.95988:2617	-1.694675989

					86.99337:34557 88.02229:13516 88.96906:21625 88.9912:7541 89.06057:12576 90.94817:24070 91.05512:2300 103.02477:21585 105.0037:74970 105.02205:4202 105.93541:3787 106.00473:9585 106.97923:7274 107.00154:53372 107.08635:3771 107.95142:12380 108.95818:7160 110.0099:6892 111.98397:3170 113.00904:48697 113.96435:7761 114.01051:2907 114.97137:6584 117.04028:2978 117.0703:2871 118.94255:3128 121.01648:3235 121.03509:13027 121.10111:11171 122.06105:2727 123.01447:26808 125.01198:17761 125.96115:25361 126.04004:2371 128.01973:21864 128.95081:121299 130.01692:6949 131.01961:701441 131.05513:9436 131.97488:3847 132.02069:66910 132.08136:2698 135.9462:58359 136.07553:2788 136.95322:15314 145.03415:12778 146.9614:139129 147.0509:2800 147.96161:3209 148.0282:3335 149.01271:44943 149.0293:340221 149.06529:35507 150.03139:35596 150.06636:4147 150.09077:3021 151.03519:9173 153.95506:71771 154.03494:111787 155.03722:15170 158.9621:32491 159.01347:154815 159.04971:59909 160.01512:15380 160.05119:3854 160.07616:10443 163.04613:8634 167.02315:41225 167.03952:11530 167.05795:9037 168.05148:3536 169.97688:36358 172.02777:7305 172.04491:36341 172.08128:6545 176.9727:30962 177.02397:513871 177.06137:109882 177.10069:21051 177.13913:16793 177.16464:7888 177.95625:16579 178.02615:61127 178.04807:18384 178.06264:17749 178.08633:56230 178.12125:5372 178.15923:39174	
Phthalic anhydride	12.648	149.02328	[M+H]+	149.02328	65.0396:211421 69.201:52236 91.05432:9750 93.0341:117325 109.07696:15183 111.04485:91492 121.02913:980563 121.03923:166924 121.0885:33863 122.03188:59011 135.10524:10639 149.02399:3330280 149.08096:40366 150.02629:227748 150.10301:34639 150.1275:301247	0

3h-Indole-3-propanoic acid,a-amino-	6.672	203.08142	[M-H]-	203.0826	68.51926:36429 72.00743:783159 73.01107:7206 74.02323:1020642 75.02633:10094 116.04919:2882661 117.05279:158695 129.0573:49082 130.06479:60252 132.08122:29114 142.06523:785977 143.06841:46554 157.07652:36612 159.09164:909332 160.0956:77997 161.04692:28276 185.07158:7451 186.0547:174280 187.05745:16936 203.08224:3941278 204.08533:344903	-5.810443632
Trimetozine	11.529	282.1311	[M+2H]2+	282.13358	55.05515:12877 57.07088:149870 58.07423:3606 60.08162:4411 65.87739:2490 67.05529:31190 69.07074:31396 71.02942:3660 71.08629:111513 72.08978:3802 81.07037:49182 83.08643:22488 85.10204:59050 86.09722:39361 86.10548:2920 89.06057:210022 95.08628:76081 96.08946:3876 97.10149:16910 104.10745:59090 107.07062:2807 109.10183:42212 111.11691:9873 123.11665:20071 124.99977:3076 125.09659:3370 130.08588:5088 133.0862:80953 137.13251:4653 146.97318:5927 149.01884:6162 150.519:4167 151.09511:2686 156.51775:22070 165.0248:6355 165.52434:27887 166.54314:3059 176.53346:5959 177.03323:17710 177.11104:16295 178.91985:3273 181.02649:3107 184.07422:33921 190.87262:3224 193.53638:19726 196.93059:3192 202.54134:11298 203.42458:2568 205.04672:4741 208.88416:16861 211.20627:23378 213.88849:12395 214.93994:33187 218.86902:5714 222.94498:3007 224.877:3284 231.90112:51595 236.88004:32009 239.15036:2806 240.10078:26281 240.95639:11311 241.88527:26133 249.9109:5017 252.95494:43868 254.91582:12332 255.00848:3633 256.00388:257889 257.00516:20134 258.00473:4027 258.89926:3283 259.8941:45622 261.61942:25835 262.11731:9878 270.62323:20211 270.96429:16533 271.12509:3391 274.01471:30661 280.12903:4406 282.13031:22811 282.22174:9171	-8.790162447

					282.28018:17209 282.63138:5684 282.9108:20140 283.17291:17509 283.26331:10248	
5-Cx-MEHA	8.61	287.14923	[M-H]-	287.15002	59.01237:1988 69.07284:8505 73.02772:6415 83.04802:1813 95.04828:2982 97.06452:11382 99.07963:10046 109.06435:5999 111.07957:8284 113.05876:17093 115.07491:3769 125.05902:3154 127.07542:25605 129.0909:30183 137.0593:3189 143.06946:16189 143.1053:1905 155.07019:5575 171.10126:5848 173.082:13135 179.1432:2269 189.12744:5615 197.15471:3380 207.13879:29810 218.98149:8060 223.13341:2272 225.14819:73184 226.15106:5676 241.14438:14910 243.15939:247066 244.1624:21901 251.12886:10875 269.13739:208105 270.14151:20177 287.1506:1088346 288.15457:101314	-2.751175152
5-Hydroxytryptophan	6.672	219.07657	[M-H]-	219.07751	69.59521:2093 69.59851:3149 72.00768:51799 74.02287:84162 83.04893:30750 86.02286:44275 88.0385:2494 89.02287:7025 92.04865:6000 101.05907:2530 116.04931:2572 118.0283:2280 118.06479:2248 130.06447:6395 131.03639:9682 132.04393:280369 133.04732:16714 139.05:13367 144.0437:152753 145.04886:37826 146.05977:11305 148.07617:2626 155.0592:2771 157.07497:123095 158.05975:99343 158.07962:9833 159.06325:7875 172.97603:6465 173.07101:8871 174.01874:15211 175.08603:171418 176.09061:15562 177.0424:3133 190.98625:8546 192.02937:5307 201.06657:18961 202.01305:16911 202.04898:12525 203.05217:1742 219.07683:500118 220.02402:8479 220.07964:48013	-4.290718842
N-(3,5-dimethoxyphenyl)-2-[2-oxo-4-(piperidylcarbo	9.26	494.19379	[M-H]-	494.1933	59.01237:10934 70.36138:3179 122.05959:19730 134.05965:2943 136.07568:21432 146.05991:40713 148.07466:11679 150.34933:1877 161.07228:2406 172.07549:2689 180.06551:1955 186.09082:4041 187.08676:2314 194.08244:1859 198.09276:3953 199.09877:2327	0.991514859

nyl)hydroquinolyl]acetamide					205.18742:1746 212.10652:2736 214.12178:89503 215.12683:9345 227.08292:1739 228.08911:2503 229.09871:73115 230.10184:6079 239.11797:2403 241.09761:203596 242.10201:20776 246.11337:2283 255.11259:14223 256.12:117492 257.12988:273081 258.13519:31639 259.12015:2245 259.14365:17819 269.25049:3526 276.21268:1728 283.10953:11472 300.11237:72535 301.12085:709024 302.12454:61283 315.32199:1722 346.11722:29323 347.12112:3743 422.16907:2012 450.20715:13704 476.18362:1783 494.19159:12116 494.32733:3079 495.27728:2744	
[3-Methyl-1-[3-methyl-1-oxo-1-(2,3,4,5,6-pentahydroxyhexoxy)pentan-2-yl]oxy-1-oxopentan-2-yl] 2-hydroxy-3-methylpentanoate	7.774	525.28918	[M+Na]+	525.29059	69.97949:8505 100.20873:3676 118.47398:3871 156.41937:4019 178.11223:3666 183.74091:3870 280.87305:3941 323.39127:4881 330.86511:4641 348.81314:3775 357.97333:4791 382.75137:4104 387.74982:4221 525.28711:8186639 526.29309:1060823	-2.684228552
Tri(butoxyethyl)phosphate	12.57	399.25034	[M+H]+	399.25058	55.05518:256499 57.07084:824706 58.07381:13497 59.05003:75854 70.49087:9382 73.02943:27290 83.08639:297361 84.08952:7429 89.06053:42668 98.98512:139595 101.09658:550036 102.09984:29662 124.9997:67686 142.12329:37668 143.01106:166029 184.07448:35771 195.23276:7201 199.0735:1197917 200.07631:40558 225.08824:29542	-0.60112624

					243.09909:35155 264.26764:12460 271.15475:7481 299.16281:520129 300.16669:42860 396.58807:8405 399.25305:107742 400.24606:7680	
Vincadifformine(1+)	8.214	340.20905	[M+2H]2+	340.20999	58.04235:40743 68.11066:7771 68.11492:26283 73.02944:149384 73.06554:16208 74.03285:3981 80.05531:153096 80.55698:6998 87.04448:56158 89.06052:829857 90.06385:31553 91.07575:4388 102.06846:119476 102.57012:14836 107.07061:14997 111.07375:22031 115.07552:3408 122.58072:3653 124.0809:66698 130.08743:28711 131.07082:14896 133.08612:437133 133.58855:6571 134.08894:22677 144.59436:5810 146.09326:25743 153.59891:13149 155.09886:28461 159.10295:11361 164.10579:16638 168.10732:11053 175.61168:11756 177.11293:114351 199.12683:10615 221.13751:15648 283.17743:12987 341.30606:5104	-2.762999405
Dibutyl phthalate	12.648	279.15933	[M+H]+	279.15909	50.01648:19857 57.0708:3867912 58.07411:125500 65.0396:33708 69.06362:76557 69.06798:30499 79.6749:17734 87.8065:19073 121.02913:16835 125.30784:20226 149.02391:13510532 150.0262:936157 157.05215:17338 164.09581:18067 167.03342:282670 190.04994:106233 205.08569:103745 215.52965:18726 223.09659:31496	0.859724826
13-Hydroxyoctadeca-9,12-dienoic acid	12.37	295.22787	[M-H]-	295.22787	62.86155:1567 71.02831:1257 85.72607:1342 88.61403:1568 106.0392:1623 113.09526:3407 140.15134:1604 171.1013:26650 179.14323:1604 181.12204:1404 195.13823:85269 196.13995:6334 216.54333:1306 233.18475:1364 251.23825:2588 259.20328:1347 277.21686:127089 278.22116:15845 295.22708:497063 296.23041:70072	0
Pentalenolactone D	9.846	309.13455	[M-H]-	309.13431	59.0121:11681 71.01241:1431 74.02269:8487 86.05901:1670 95.04829:1488 96.95828:7719 102.9467:2308 113.05877:7673 115.03874:43016 123.04369:6312 123.07993:7486 127.07539:2202 130.98236:3322 133.06416:1520 135.08127:2081 137.05948:22139 146.93777:7098	0.776361576

					149.09485:5454 151.07416:33240 153.05348:1442 158.97768:3041 166.06148:2870 174.87741:1942 175.07498:2916 178.06114:1972 178.97734:5519 179.07048:1575 187.11105:1713 193.08636:46558 197.87611:1409 203.14279:2309 205.12315:32612 214.10106:2273 218.86784:2015 223.13345:6575 229.12508:1877 236.87886:2717 238.46353:1513 239.0724:1528 247.13091:2422 249.11432:26959 250.1165:1709 259.77017:1669 265.18234:4447 266.17627:3467 267.12216:150709 268.12717:13125 291.12344:1481 292.15793:1818 309.13245:45100 309.17371:48363 310.16577:24365	
(19E)-4-Ethyl-2,16-Didehydrocur-19-En-4-Ium-17-Al	6.191	355.16132	[M-H]-	355.15799	69.99283:2702 88.03863:40083 110.02345:5288 114.05381:8453 127.04958:1760 128.03317:6925 129.06578:7984 130.04901:1345 131.80659:1401 139.0858:1553 139.53963:1590 141.10152:11398 153.06606:7147 157.09718:2388 169.06061:17925 185.09082:182549 186.09566:7512 187.07222:5495 198.12451:2460 266.11514:17466 269.8045:1986 269.84235:1401 293.16461:1412 312.02914:2022 325.15292:1799 334.13202:1515 354.93005:1721 355.16476:288324 356.16391:23877	9.376108926
Tetraethylene glycol	6.434	195.12312	[M+H]+	195.12268	69.76347:16290 70.06564:11213 73.06553:20729 87.04458:62121 89.06059:6429632 90.06377:194703 107.07061:381877 108.07478:5820 113.96436:6745 130.08713:190964 133.08606:1354089 134.08894:59755 136.07555:17461 148.97742:5857 150.09254:17157 151.09654:229209 152.09882:6055 163.03824:7825 168.0887:9636 172.97765:21397 177.11298:93571 178.08745:6077 178.11676:6319 181.05879:5902 195.07756:27732 195.1244:357275 196.08408:83496 196.12836:11025	2.254991578
Pterosin G	11.948	235.13327	[M+H]+	235.13287	53.00322:4790 55.05515:9214 57.07082:1696190 58.0742:52525 67.59751:79877 81.07098:5713 93.07059:35222 95.08613:9607	1.701165813

					96.84246:10103 97.06519:5800 111.08025:5868 121.06576:7921 123.04411:28148 123.08037:6204 133.10222:5385 135.08142:5402 147.08138:10342 149.0965:7395 151.07567:100644 152.07925:7612 161.06024:171244 161.13208:8807 162.06793:47782 163.07578:6010 165.09114:7130 173.09639:19528 174.10295:5916 175.07605:50633 175.11108:44121 177.0909:29007 179.0697:1089438 180.07358:99957 189.12881:73161 190.13196:7298 193.0849:36290 193.12331:27996 199.11084:6579 202.09917:9904 207.13713:34866 217.12288:219695 217.19273:6448 218.12512:29332 235.13281:1811074 235.17052:60622 236.13799:221006 236.23442:6132	
PC(18:1/16:0)	12.316	760.58459	[M+H]+	760.58508	57.07079:192563 58.0637:265480 58.06664:1408983 59.07414:227775 60.0816:6290794 67.05521:229540 69.07087:847149 69.13004:696138 71.07364:1259355 81.07094:677605 83.08636:646592 86.09711:27076288 87.10044:954394 95.08588:881873 97.10143:570881 98.98434:1072369 104.10737:2963602 109.10175:240576 121.10234:168683 124.99966:12052684 149.13269:192996 184.07423:333764192 185.07806:14160663 186.07814:1696034 311.25632:177551 478.32495:205874 496.33997:223800 575.50476:2676408 576.50201:620204 760.57544:9705583 761.58795:4337836	-0.644240878
N-lactoyl-Glycine	0.869	146.0442	[M-H]-	146.04533	59.01238:5647 71.01241:9339 84.04388:6869 85.02799:24322 89.02288:13521 96.83886:6176 102.05494:1436269 103.0518:7474 103.0575:53942 120.9832:6138 128.03447:744801 129.03806:39907 146.04475:548820 147.04764:31170	-7.737323747
Sebacic acid dimethyl ester	10.406	231.1595	[M+H]+	231.15907	57.07083:2004 65.16066:1170 67.05526:1444 69.07071:5096 71.01864:1393 71.02318:1518 76.16451:1168 81.0703:2744 83.08643:6413 91.05516:1221 93.07069:2064 94.64187:1071 97.06522:2202 107.08639:2692	1.860190907

					109.10075:2026 111.08028:3033 121.10117:1870 149.13275:15286 159.11821:1790 167.14201:7595 174.12695:5160 184.93015:1616 185.13306:1343 207.94536:1394 208.95578:1916 231.08258:2164 231.1059:2318 231.13922:2177 231.15923:8801 231.25258:6282 232.11685:10914 232.16916:1928 232.20607:2036	
Pyridine	13.586	80.05029	[M+H]+	80.04948	53.00327:50133 53.03955:45148 53.99817:9135 54.03474:18604 65.03911:2556 66.04709:3308 69.35577:7255 79.05528:102926 80.05058:425892 81.03432:19770 81.04536:39490 81.05345:14867 81.0703:167733	10.11874156
2,2,6,6-Tetramethylpiperidin-1-ol	11.141	158.15427	[M+H]+	158.15395	57.07083:396459 58.0738:8694 69.49236:22725 70.06618:33265 71.04984:10273 74.06063:12454 76.93369:42259 81.04526:64159 82.05318:15124 84.04482:130226 84.08144:41158 86.09713:8781 95.06058:136347 97.00806:13369 98.06046:41081 102.09197:1658538 103.09499:64914 106.99287:31754 107.07059:33764 111.05579:451126 112.07616:579558 112.11201:112098 113.07991:27873 114.09254:8607 116.10845:45363 117.95952:163515 123.05573:501709 125.00246:81278 130.05067:12888 130.08699:43714 131.08218:11102 131.11777:9403 135.10075:12731 135.97037:57359 140.07112:38162 141.01926:14064 141.06532:602865 158.08195:655967 158.09705:180954 158.11812:230944 158.15363:4616143 158.98688:26179 159.07628:43092 159.15837:289491	2.023344975
N-[4-[(9S,13R)-16-hydroxy-7,9,13-trimethyl-5-oxapentacyclo[	12.387	478.32782	[M+Na]+	478.32913	53.1506:12062 55.05513:31892 56.05049:47416 57.03439:11122 57.0708:234076 60.08155:5276 65.03953:28414 67.05511:37962 69.07061:48050 70.06612:10300 71.07366:260012 71.08617:193719 81.07088:83400 83.04974:20531 83.08631:87090 85.10188:139006 86.09705:3285721 87.09517:4987 87.1005:94842 95.08605:152358 97.10147:48289 98.98506:31622 99.11783:8801 104.10726:5250	-2.738700024

10.8.0.02,9.04,8.013,18]icosa-6,18-dien-6-yl]-2-methylbutyl]acetamide					109.1017:81418 111.1179:9359 123.11779:41682 124.99966:707529 125.13229:10391 126.00371:4580 136.99988:37154 137.13225:15163 143.01108:9066 151.0157:13604 151.1478:4934 163.01631:1038763 164.0199:26607 166.0264:10573 181.02623:266093 182.03067:4712 184.07419:311581 185.0779:8709 239.23796:57621 240.09996:10149 283.26303:89109 284.26489:11654 295.26227:16813 337.27512:8483 419.25235:689638 420.25555:104249 478.25537:30238 478.3248:5474187 479.32849:867468	
Dehydroabietamide	6.806	300.2019	[M+NH4]+	300.19943	68.32226:194795 68.32655:187549 87.04518:232483 89.06052:17333406 90.06376:604384 107.07089:404095 130.0858:272847 133.0862:9175005 134.08911:523111 151.09688:156301 177.11316:2519737 178.11679:235290 195.12425:442326 221.13753:183461 239.14986:485359 283.17709:6819980 284.17847:990073	8.227863724
[3-heptadecanoyloxy-2-[-octadeca-9,12-dienoyl]oxypropyl] 2-(trimethylazaniumyl)ethyl phosphate	10.654	830.59076	[M-H]-	830.59003	67.65118:38945 67.6554:25900 78.95728:69169 152.99409:9472 168.04158:95921 224.06871:65313 255.2319:6635 279.23236:2484808 280.23517:202120 281.24802:628921 282.25204:57525 283.26288:883379 284.26938:55180 307.26425:8685 486.29541:8965 490.32767:15997 506.33035:9620 508.34671:75047 770.57831:880025 771.57227:180168 830.5968:5775	0.878893285
[1-(7-Hydroxy-2,4b,8,8-Tetramethyl-4,4a,5,6,7,8a,9,	8.528	509.3121	[M+NH4]2+	509.31	71.04986:4206 73.02937:35070 73.06554:18471 80.05532:11713 83.04979:40333 85.02901:4232 87.0444:202379 89.06052:763718 90.06368:29794 95.05018:20359 99.04496:90230 102.06754:5137 107.07061:18472 109.06507:3946 111.04466:81446 112.04809:4208	4.12322554

10-Octahydro-3H-Phenanthren-2-Yl)-2-[3,4,5-Trihydroxy-6-(Hydroxymethyl)Oxan-2-Yl]Oxyethyl] Acetate					113.06076:22194 117.09076:17097 122.06487:24309 122.56656:4556 125.06075:15552 129.0549:30456 130.08684:21476 131.07085:45013 133.08614:447920 134.0892:26903 137.05942:15955 144.07788:19800 151.09686:6473 153.08383:3754 155.07008:58790 155.09927:4605 157.08575:5613 166.09152:17392 173.08127:20507 175.0963:21343 177.11313:147498 178.11678:5828 188.10445:12138 195.1241:12281 197.11154:4462 199.097:20450 210.1179:4949 217.10776:15359 219.12541:18115 221.13751:17072 239.15033:17206 241.13707:29161 243.12422:17585 261.134:17881 263.15005:32773 263.64938:5442 271.6636:3642 283.1774:16921 285.16153:34521 285.66583:11834 287.15067:12850 305.16034:17643 307.1763:24684 307.67844:5913 327.20148:19333 329.18774:6310 337.70471:3871 349.1842:16370 351.19794:12518 371.22913:14700 393.20898:6686 415.25146:14414 437.23447:16227 459.27847:12482 481.26556:6569 492.28564:15972 492.78326:11335 493.90564:5206 503.30286:11059	
3-Hydroxy-2-methylpyridine-4,5-dicarboxylate	1.963	196.02188	[M-H]-	196.0246	71.01808:1653 72.2346:1436 78.95765:2838 88.04869:1404 96.95827:2214 103.91842:22890 105.86542:1317 112.03956:1414 116.92694:1305 121.66508:1434 128.03323:425674 129.03665:15732 143.0826:1351 150.9632:1557 151.89636:9343 152.89363:179336 152.95296:1797 158.84467:2228 160.83986:191809 161.84088:2239 168.01523:13740 168.88777:5225 168.96065:2764 183.87646:2845 195.80957:173113 196.00714:3349 196.88251:11083 196.96059:1366	-13.87580946
PC(14:0/16:1)	12.296	704.52313	[M+H]+	704.52246	55.05513:5972 57.0708:17640 58.0633:6234 58.06665:34290 59.05005:13456 59.07458:12132 60.08161:158011 67.05522:17677 69.0705:28825 71.07376:29553 71.0862:14510 81.07011:29478 83.08638:21565 85.10191:7797 86.09711:713618 87.10063:20662	0.95099878

					89.06049:7202 95.08623:28819 96.84244:7120 97.10146:17061 98.98514:22335 104.10744:77198 109.10178:19326 111.11798:5362 121.1011:8080 124.99971:310113 135.11713:6228 149.13272:5428 184.07426:8402226 185.0782:258743 225.59825:6079 519.44244:35817 520.44519:6886 535.48096:13086 549.48663:52270 550.49115:7451 563.50659:62329 564.5097:6946 704.51575:260446 705.52747:81468	
3-Hydroxytetradecanedioic acid	9.421	273.17059	[M-H]-	273.1702	59.01195:1887 61.43646:1283 68.6144:10608 79.95585:5791 83.02364:1771 85.06445:1675 85.33894:1237 94.89731:1380 96.84248:2136 96.95835:18839 107.72964:1191 111.07959:2519 114.60221:1465 115.03864:2406 115.9486:1400 128.03319:2178 129.03659:1757 130.98218:20037 131.98187:1943 139.11226:1960 140.1655:1441 144.10913:2066 145.04893:5348 146.77574:1385 149.04651:2760 158.97748:36755 159.97858:1900 180.95338:1381 186.0788:1286 193.15994:21896 194.04401:2584 203.43968:1406 216.96707:1320 229.0027:14023 229.16125:4813 237.14844:40348 238.153:2481 255.15849:54132 256.16284:2900 273.12955:14775 273.17307:183625 273.62759:5894 274.00574:2216 274.13055:2144 274.17294:21519	1.42768135
2,3,4-Trihydroxy-5-[3-(4-hydroxyphenyl)prop-2-enoyloxy]hexanedioic acid	0.958	355.06604	[M-H2O-H]-	355.06702	56.18184:1307 59.01238:5177 71.01298:1399 72.99164:7044 83.87752:1454 85.02799:2278 92.92607:96714 94.9237:42796 96.92027:8311 96.96825:2685 103.0022:2347 113.02238:12508 122.42082:1861 124.00593:5157 126.90167:2210 131.03357:2917 144.86852:2892 150.88582:3412 160.8399:2226 162.83943:1843 164.83633:1644 168.02721:1653 175.02446:1601 178.89561:2057 179.05458:1578 181.96419:6537 182.04103:2560 188.01982:3051 193.03502:23947 193.50676:1405 194.89537:1921 205.41623:1332 232.54105:1455 234.86552:2292 236.86362:11675 239.96188:5905 240.00067:7730	-2.760042315

					249.50984:1318 260.88165:5720 278.07684:1637 279.08231:1947 287.05841:1461 294.82343:3200 296.08539:7713 296.81638:5678 296.9328:2252 297.08817:2738 297.96429:1507 318.84024:5944 319.05093:5614 355.0856:10288 356.04254:1649 356.09351:1643	
1,2-Dihydroisoquinoline	9.82	130.06467	[M-H]-	130.06622	65.01275:20230 69.55833:6497 73.91708:2956 85.02724:3699 85.06443:4337 86.05976:16270 87.0433:26905 88.03853:27697 89.01252:17743 102.98732:9325 112.03842:10005 129.05739:185599 130.03934:62756 130.06448:1872458 130.92976:3463 131.03479:58885 131.06787:127381	-11.91700658
3,3,3-Trifluoro-1-propanol	1.418	113.02264	[M-H]-	113.02197	51.63444:80997 52.66847:83789 61.13432:76416 68.99397:44851856 69.51048:208895 69.99727:165175 91.18452:86571 92.84023:91981 106.42446:80413 112.98444:2829723	5.92805098
2-Methyl-1,4-benzoquinone	3.803	123.04422	[M+H]+	123.04408	53.03947:8144 67.05524:17278 68.05042:30679 78.03465:8794 79.0545:27205 80.04993:407778 81.05368:6691 81.07029:18604 95.05011:247716 95.05984:28052 96.04488:245207 96.08141:16733 105.04555:22410 106.02961:37440 112.04034:67312 123.0506:125884 123.05577:2543788 123.09133:46945 124.03914:660725 124.05863:116585 124.07578:49325 124.08627:8482	1.137803623
Sodium taurocholate monohydrate	7.548	516.30273	[M+NH4]+	516.29901	55.05515:6669 59.05006:7834 67.05524:2722 69.03425:84987 69.74634:5719 69.74966:2884 70.06564:2274 73.0294:23072 73.06557:13230 81.03429:12473 83.0498:47546 85.02901:9392 87.04442:216544 88.04811:2268 89.06051:507109 90.06372:12249 95.05:17913 99.04494:187169 100.04815:4401 103.03969:3489 107.07055:28554 109.06506:2875 111.04471:138547 113.06023:14454 117.0547:3845 117.09195:3162 125.06065:51567 127.03905:2987 127.07573:2634 129.05493:28453 130.08701:17296 131.07066:39743	1.413909355

					133.08614:331096 134.08928:8359 137.05954:24693 145.08701:2162 151.09674:32925 155.06985:124290 156.07486:3333 157.08574:2506 173.08112:37990 175.09622:26582 177.11307:72595 178.11674:2446 195.12331:24595 199.09702:36808 213.11328:3532 217.10674:14452 219.1241:7819 221.13748:4276 239.14983:13629 243.12337:26300 257.13815:7950 261.13409:42927 262.13739:2308 283.17737:4094 287.15097:17573 303.48868:2158 305.1597:63206 331.17358:7554 349.18494:17218 367.19882:3882 393.20892:2871 411.21933:21116 413.80261:2154 455.25247:18585 499.27777:251062 500.27451:29613 516.30377:8603	
Acetylglycine	1.597	116.03378	[M-H]-	116.03532	59.01234:5028 69.99001:2922 71.0487:8806 72.04402:17378 73.02782:68168 74.02335:87755 88.03854:14043 98.02271:8176 99.00695:9281 99.92402:38596 115.9189:25126 116.03378:50163 116.07059:30608 116.92686:311137 117.01791:16161 117.05416:11030	-13.27182103
Dodecanedioic acid	10.416	229.14365	[M-H]-	229.14452	59.0119:8260 70.17641:2637 83.02356:6310 99.92395:9656 116.92684:5599 132.97966:6524 139.11058:2031 160.97527:6646 165.12761:1697 167.14241:158307 168.14699:12514 181.89653:1892 182.9528:10331 185.15283:5599 185.92465:1812 211.134:256464 212.13559:27071 228.96048:1793 229.02293:5430 229.14458:844161 230.14806:75415	-3.796730552
9K,12,13-DiHODE	9.837	325.20123	[M-H]-	325.20203	59.01236:5221 69.0331:2698 69.14148:5777 109.06434:5591 123.0799:2697 125.059:3648 125.0962:76200 141.09071:19277 147.11696:1893 151.07585:11766 163.11169:7453 169.08565:740630 170.08879:41114 183.01103:11945 183.10555:1729 191.10643:9089 197.02872:1524 209.11778:1794 245.19308:2449 279.19281:5768 289.18283:25829 290.18341:2509 290.58704:1582 307.19293:2727 325.18613:433940 326.18427:48929	-2.460009244

Alliogenin	8.484	487.29886	[M+2H]2+	487.30301	69.03425:201465 70.03783:6282 73.02941:46123 73.06561:31579 80.05532:5257 81.0343:5893 83.04994:47052 87.04442:239935 88.04812:6446 89.06052:951108 90.06366:27774 95.05008:14220 99.04494:125750 100.0529:13645 102.06852:7832 107.0706:21868 109.06507:5331 111.04469:112578 113.06018:36898 117.09196:19379 122.06465:42323 125.06075:16978 129.05502:40611 130.08607:22503 131.07057:51490 133.08615:559709 134.08914:26953 137.05942:16772 139.07497:6628 144.07796:27915 151.09685:6474 155.06992:92832 155.09927:5191 157.08575:5817 166.091:24034 173.08115:32916 175.09634:46258 177.11307:152193 178.11676:12317 188.10445:12936 195.12152:17071 197.10892:12462 199.09715:23624 199.62611:4967 210.1179:7141 217.10776:20491 219.12389:37573 221.13762:38431 232.1322:5410 239.15036:22000 241.13667:41155 241.63892:6256 243.12422:18304 261.13367:22493 263.15033:47786 263.65341:13852 283.17493:22563 285.1611:49835 285.66583:16356 287.15067:12590 293.6745:4423 305.16034:16968 307.17673:40229 307.67844:4417 327.20285:20001 329.18774:15591 331.17361:7179 349.1842:19336 351.20416:4912 371.22913:18707 393.20895:4675 415.25146:18214 437.23447:7574 459.27847:16612 470.26611:26549 470.77606:14571 481.26556:5700 487.25882:7114	-8.516261781
Sclareol	10.787	273.254	[M+H]+	273.2565	55.05516:2022 57.07084:3307 62.83197:1539 67.05526:2482 69.07071:2351 69.62608:4809 81.071:7675 83.08643:9045 88.07635:3699 95.08611:12609 97.10151:5827 106.08659:2385 109.10184:6612 119.08668:2078 129.01884:8968 133.10226:2008 135.11719:1672 137.09598:1977 148.50084:1455 155.97502:3458 157.01486:1923 175.14932:13487 193.15898:10234 194.83237:1661 209.20811:1887 211.17047:3410	-9.148913201

					212.23677:1834 228.19469:26851 229.17877:2579 238.45042:3628 255.21295:13032 256.2645:2977 273.09619:2823 273.14328:2316 273.22464:2408 273.26318:2501 274.18231:9122 274.27283:109501	
Isoplumbagin	1.663	187.04051	[M-H2O-H]-	187.03952	59.01235:5289 70.38823:4058 71.01244:31626 75.00727:5553 87.92342:47861 89.02284:14021 96.9684:16463 97.06426:3347 99.924:10516 100.92428:3756 103.9184:11829 105.93462:4278 115.01865:535406 116.02319:13302 116.07043:17729 119.0331:4585 123.94436:26433 125.09614:90628 126.09871:5237 141.86639:10865 142.94304:5652 142.96231:68813 143.05008:4619 143.96077:17122 145.09689:31250 159.87683:3962 172.01764:4174 186.85367:25348 187.04047:2582398 187.09641:63081 188.0439:126560 188.10045:3104	5.292999041
PE(O-20:0/22:6)	11.141	804.58691	[M-H]-	804.59125	78.95727:158354 96.84115:41389 96.9673:23544 152.99449:83067 168.04172:130082 223.00049:39964 224.06883:85063 241.01233:162349 253.21754:600796 255.23203:1839109 256.23642:187918 257.23938:10922 259.02121:8116 279.23282:152139 280.23529:154624 281.24814:3436539 282.25275:314334 283.26309:25448 297.03949:23512 389.2084:48329 462.3031:28179 480.30966:114788 481.30988:19346 488.31583:16807 506.33063:25646 551.25885:50490 744.55798:1236536 745.56177:264591 805.48126:415948	-5.394043249
Hapalindole D	1.338	337.17215	[2M+H]+	337.17328	60.05648:127992 62.98292:4458 69.98174:6104 70.06617:340073 71.06918:11786 72.08169:53139 80.94859:6711 84.08147:17913 96.92297:7293 97.077:19137 98.91982:6462 104.10738:6621 112.08749:138629 113.09134:12080 114.09141:252558 114.10297:217333 115.09544:12479 115.10598:16275 120.00399:4271 131.1291:35860 138.1017:15194 156.11375:46663 157.11565:3897 164.02985:27713 164.93034:13595 173.13962:589605 174.14291:37740 175.11833:18679	-3.351392495

					177.06186:7256 198.11272:17028 203.01512:6377 216.12221:26768 217.12904:255833 218.13333:21062 218.98895:60800 219.08107:6508 220.06535:52897 220.41573:3912 220.9879:11612 221.01906:11072 234.13419:13733 257.16165:74635 258.16202:6746 260.11249:4919 280.09348:17245 293.1835:18938 301.1506:4365 319.16418:4210 337.16989:191418 338.1759:31954	
Anthranilate	13.457	102.03436	[M+NH4] 2+	102.034	56.96553:5485268 58.5056:307162 67.51063:1412005 69.74581:491281 70.0134:190232 76.37994:121673 76.51614:974998 79.01871:2105568 81.555:115009 82.97162:116042 85.02904:297594 88.02391:2401804 90.52679:547799 97.02895:158667 99.53197:526679 102.09202:156749	3.528235686
Cholestane-3,7,12,25-tetrol-3-glucuronide	11.144	611.37903	[M-H]-	611.37952	57.03291:7590 59.01194:8331 71.01183:10702 72.99162:3102 73.02772:2389 75.00683:80168 78.95765:7944 85.02808:54554 87.00743:24801 88.00247:10879 88.98624:2992 89.02296:101500 95.01226:3235 96.83795:9599 96.84428:4756 99.00671:9073 112.01481:8990 113.02237:77961 115.00236:3357 116.01034:15645 117.01692:2394 129.01711:7526 157.01317:9935 175.02444:3897 226.0898:2185 283.26199:18888 508.33673:12292 593.37469:3486 611.3764:1089252 612.38098:214064	-0.801466166
Diisobutyl adipate	12.147	257.17554	[M-H]-	257.17529	59.01195:2122 60.20479:1554 71.01184:1563 72.99104:2977 74.02268:2119 85.02799:2829 99.92403:1539 106.0392:1920 108.46835:1323 116.92694:2896 141.12709:13863 155.14349:6589 160.04961:1443 171.13737:2618 183.1385:2336 193.19055:1429 195.17436:7503 211.16919:7834 213.18588:2744 217.85791:1484 231.05157:1644 239.16394:9715 255.16255:1625 257.17679:515987 258.17737:64978	0.972099613
indole-3-glycol aldehyde	9.809	176.07076	[M+H]+	176.0706	71.04986:2776 84.08146:2397 89.06052:6303 91.05514:2263 97.00808:2597 104.07111:6657 106.99289:6911 107.08636:1641 112.07621:6529	0.908726386

					115.03923:1719 120.08142:13087 121.10114:1884 125.00232:27495 128.95073:1782 130.06479:1276465 131.06944:81995 131.08362:2505 134.05984:1676 146.96133:2840 148.01797:11640 148.07607:8514 148.09657:14059 149.02228:2126 149.0706:2865 153.93732:13632 153.95518:2250 153.98055:2036 154.03493:1821 158.00284:3359 158.05936:2870 158.08199:5950 158.1178:1854 160.07619:3027 166.02859:9280 171.09239:32664 171.94652:1671 172.09416:2921 176.01405:9034 176.07164:454626 176.10712:17758 176.12706:13340 176.14258:8052 177.0529:4860 177.07301:36193 177.10146:12863 177.12666:4863 177.16467:3304	
12 Hydroxy arachidonic acid	12.491	319.22742	[M-H]-	319.22787	57.0329:249388 59.01208:1976653 67.05358:94339 69.03333:493701 69.10659:206122 71.01239:221542 83.04872:107934 107.08466:861758 109.06434:82241 117.06974:91199 119.08486:88261 127.11069:76434 133.10052:110645 135.11707:1743513 139.11073:1070174 145.10027:75502 153.09116:111994 153.12733:598879 161.09552:247306 163.11168:1755177 163.14923:258452 164.11604:72966 177.09152:128466 177.16307:72400 179.10684:11430944 180.11133:617423 181.12198:230993 189.16443:96100 203.17859:1133097 207.10132:1192866 208.10889:1624004 229.1942:431267 240.09937:355620 257.22757:4702039 258.22849:397570 271.20956:83257 275.23611:519086 283.20435:67464 301.21521:5672926 302.21808:483854 319.22928:17158614 320.23218:1654684	-1.409651357
3-(4-Methoxy-2-methylphenyl)-2,5-dimethyl-N-pentan-3-	10.89	351.2171	[M-H]-	351.21902	51.01128:1338 56.00769:1206 63.36561:1284 71.01237:359252 72.01556:5696 75.4765:1304 78.95763:2325 96.83884:1259 98.84619:1285 116.92691:1644 124.84044:1480 143.74879:1470 173.93802:1661 177.9845:1388 179.10681:2650 195.10193:2039 207.10187:1971 261.18558:2210 269.939:1541 271.13757:1725 279.19867:11811	-5.466674328

ylpyrazolo[1,5-a]pyrimidin-7-amine					289.18271:2831 333.20703:2518 351.2175:110992 352.21851:12517	
Ectoine	1.15	165.06374	[M+Na]+	165.06342	102.05582:7330 105.00372:6232 120.08134:14249 121.06452:4399 164.92018:108637 165.06306:432711 165.10124:8273 165.13948:5381 166.04691:91578 166.0672:19356 166.08339:127436	1.938648793
N-Hydroxy-1-aminonaphthalene	6.253	160.07593	[M+H-H2O]+	160.07623	51.0241:5049 55.05511:7619 55.93535:57957 56.93562:42194 56.94297:40076 58.06621:5330 67.04267:40263 69.07059:29347 69.66846:17398 70.06559:13924 72.04504:14500 72.93768:97114 74.95354:6076 77.04:5461 82.04891:7978 84.0814:17366 86.06072:16852 86.09708:22421 90.94811:74745 91.05508:7706 96.06438:8164 97.96929:25428 98.06036:49633 99.0449:7554 100.07656:13672 101.05984:7541 102.05573:21881 105.06982:63762 107.95121:5901 108.95813:18354 111.05575:14065 112.05251:9888 112.05925:56736 112.07613:13887 113.96427:573490 114.09129:99645 114.12828:34953 114.96546:16420 115.05437:339062 115.09534:6618 115.9716:6989 116.05811:23404 116.07115:7394 117.05711:145352 117.06978:40593 118.06599:46013 118.94248:8489 119.95777:8222 124.0589:76644 124.07568:14702 125.00232:8469 125.96082:17021 128.95036:26538 129.95277:39014 130.06467:24690 130.96689:7259 131.04933:34411 131.07356:15808 131.97481:247641 132.0813:1020831 133.06445:169518 133.08456:70246 134.06857:13326 135.94458:19156 136.94525:7790 141.95927:46520 142.06534:156053 142.08577:98035 143.07341:67514 146.96123:15389 147.96249:38222 148.97733:13863 153.95506:21898 154.95821:8277 154.98921:60424 159.06853:79124 159.96861:58583 160.07611:14916189 161.07985:1212964 161.10674:17194 161.1339:4827	-1.874107105

Platelet-activating factor	13.224	524.37103	[M+H]+	524.37109	57.07081:149578 58.06666:245299 59.07416:70240 60.0816:1398612 71.0737:80285 71.0862:83902 86.09715:6081708 87.10064:164312 98.98515:148466 104.10737:1262822 124.99978:2653065 184.07446:69774480 185.07591:1797156 258.11081:184884 341.30597:225516 506.3588:619973 507.36456:137591 524.375:2030892 525.37885:416553	-0.114422784
PFSA-ether	0.694	430.92792	[M-H2O-H]-	430.92642	67.76324:31104 70.99763:4094 96.83883:10016 112.98481:19558 114.98679:19235 130.98239:166640 131.98326:13132 158.97755:1432541 159.97829:123239 226.96593:590089 227.96808:63176 294.95343:70362 295.95483:4405	3.480872674
Fumaric acid	2.586	115.0022	[M-H]-	115.00372	52.84761:1207 56.71759:1215 59.01233:2302 63.38041:1168 69.13054:7091 70.02778:1328 71.01246:12021 71.04869:5144 72.04393:6221 74.02263:6255 88.03844:7268 97.93001:8784 98.0227:7391 99.92395:14046 114.93216:72555 115.00227:1416 115.01397:1211 115.03853:11087 115.91891:101468 116.03371:21355 116.07057:6315	-13.21696376
Cholesteryl sulfate	11.002	465.30338	[M-H]-	465.30399	52.33149:1399 54.98281:1662 68.89786:6685 78.95765:2175 96.83976:2192 96.95832:404135 100.68626:1567 102.94669:1487 116.92693:3293 146.93776:5404 148.58162:1523 152.99423:1619 154.06233:3360 160.31494:1511 166.26665:1676 166.28291:1555 173.92499:2526 179.10229:1627 190.9287:2732 221.20813:1657 239.48311:1517 407.28079:3514 465.30627:1064081 466.30765:151584	-1.31097092
Methanone, phenyl(2,4,6-trihydroxyphenyl)-	13.551	229.05325	[M-H]-	229.05061	69.29661:9226 69.30044:9194 79.95588:589118 80.9635:50501 83.02362:11223 97.92986:5286 99.92403:5384 132.97975:18086 138.96301:59827 149.09656:1639856 150.09953:122571 154.95853:24558 160.97537:23249 166.9581:29752 181.89676:64591 182.95305:300408 183.95215:8729 184.87372:5654 185.94403:5847 202.88487:6409	11.52583702

					210.94826:95936 228.96088:52414 229.05273:2931796 230.0555:248823	
LPE(20:0)	10.742	508.34006	[M-H]-	508.3403	78.95761:734177 96.96724:10673 110.98406:62565 122.9841:54592 152.99408:147445 168.04146:369602 182.05713:310184 183.06079:6354 224.06868:945411 225.07146:31332 242.08017:206785 243.08009:6433 281.24805:14994 283.26294:9849836 284.26932:963337 329.78369:6793 387.22992:20328 405.23788:6191 419.25607:134498 420.2583:12702 437.26395:41206 449.26413:31833 476.31134:16460 508.33676:3665758 509.34903:580262	-0.472124677
Leukotriene C4	6.372	624.29858	[M-H]-	624.29602	59.50774:1532 60.76332:1467 69.75845:4865 74.02322:12270 97.03886:1679 100.00265:2091 110.02329:13122 125.07089:2760 129.06569:6957 143.08072:13800 153.06604:14543 164.10602:2074 169.09811:10410 187.07187:32800 187.10851:1939 248.10637:2352 266.11349:44353 282.14496:3412 284.1286:8472 436.22327:28190 437.21985:2339 454.19739:10054 546.23553:1507 611.37598:1773 624.2948:199903 625.30225:33676	4.100618806
4-Hydroxy-N-methylcarbostyril	9.823	174.05479	[M-H]-	174.05602	61.98682:2916 65.0131:4697 65.99711:3126 71.0124:3382 83.02362:4041 90.97018:2068 99.92387:27875 100.93201:2293 102.96948:2983 112.08343:2670 115.91869:16954 116.923:3690 116.92661:33393 117.92742:2843 118.9641:156233 130.0645:134589 130.87436:8259 130.94255:11762 131.0677:12684 132.05522:3676 133.0394:7687 134.03462:9393 146.9597:1349482 147.05438:2556 148.05054:11234 160.97539:7616 174.01857:7250 174.02945:10030 174.05341:12566 174.95421:168604 175.03262:3385 175.03922:13894 175.06178:10657	-7.066690368
Hept-4-enoylcarnitine	7.894	272.18597	[M+H-H2O]+	272.18619	57.41792:1359 60.08162:50219 83.08637:51191 85.02903:533995 86.03278:17892 111.08147:28352 120.08131:1363 129.01881:6085 129.09111:7446 144.10262:10262 155.97498:1454 160.09732:2515	-0.808270251

					213.1134:148737 214.11397:15868 225.8653:2632 229.4323:1432 272.18677:453162 273.12607:2449 273.19067:55701	
Aloxistatin	12.49	387.21436	[M-H]-	387.21371	59.01193:6183 69.0331:2796 107.08561:3378 117.06854:1888 135.1174:10231 139.11221:2394 163.11183:10223 179.10667:69103 180.03798:6714 180.11133:1866 203.18118:5287 207.10187:7986 208.10901:10304 229.08226:5461 230.09178:59611 231.09483:3546 257.22763:28565 262.08057:87203 263.08551:6638 301.21533:37779 302.21912:2636 319.22903:205706 320.23212:24717 341.21106:37190 342.21149:2232 387.05023:2574 387.21567:40197 387.31033:3079 388.25186:5662	1.67865957
Dirithromycin	9.205	857.53979	[M+2H]2+	857.53455	60.04535:200601 69.07066:8288 70.02938:39454 70.06612:362036 71.06971:8244 72.08158:542740 73.08505:19766 74.06059:90393 83.06112:37908 84.04483:484929 84.08138:693659 85.04831:20964 85.0847:38772 86.09707:1111151 87.05588:102843 87.09522:6921 87.1003:50158 88.04024:19904 91.05515:44265 93.07063:7767 96.84242:9493 101.0714:351809 101.10782:23746 102.0557:144590 102.07532:15520 103.05394:28773 105.06692:174595 110.06132:6641 110.09637:6097 111.09242:13189 112.07616:5643 115.05089:5405 115.08717:14818 119.04963:18305 119.08146:28443 120.08118:1065540 121.08472:98207 126.05602:23528 127.04987:5480 127.08791:6290 127.12325:4849 128.10767:15288 129.06625:66024 129.10216:748090 130.05043:38887 130.06612:18134 130.10567:49239 131.11916:15187 136.07547:381728 137.07928:35283 139.08734:18240 140.07088:26043 141.06688:20647 141.10188:54448 143.11827:5565 145.06044:4699 146.12843:7596 147.07623:17864 151.08621:6528 152.14339:24334 153.06581:17438 153.10173:15226 154.05118:6383 154.09837:37277	6.110540969

					155.08093:65994 155.11761:44248 157.06142:8211 157.09689:5574 159.11429:19103 164.03366:23441 165.10303:71419 167.08249:21683 167.11731:18580 168.0659:4763 168.11343:7220 169.09746:80542 171.07736:29139 171.11337:18942 172.10742:23154 173.09244:24079 173.12859:224388 174.1312:22233 175.07208:5674 175.08746:12598 181.06079:85651 181.09802:12562 182.09331:57240 182.12874:15536 183.07639:51479 183.11368:39978 184.07199:7921 184.10753:20851 185.09242:25317 185.12816:11795 185.16521:30603 186.12305:57980 187.1076:18439 188.10191:12568 191.10281:18920 194.09154:17594 195.07712:42369 195.11356:35658 196.06058:7781 197.1272:13502 197.16396:4720 198.0885:52740 199.07089:52755 199.10808:13416 200.0735:6786 200.10303:23267 200.14064:47471 201.12431:161522 202.0822:30606 202.12086:6313 205.09674:20413 205.13295:12398 207.11415:59882 208.11026:7033 209.09322:19575 211.10922:7214 212.1046:58692 212.13989:6180 213.08665:38601 213.16042:7190 215.13995:15583 216.09773:205693 217.08035:15503 217.10332:23543 217.13472:64825 219.11479:4339 222.12312:21396 222.1608:14452 225.12334:6326 225.82977:4739 226.08426:5432 226.11974:5760 226.15497:84281 227.15846:5951 228.13591:29783 229.11906:43284 230.11215:63702 231.09575:5576 231.14906:4984 233.16541:43345 234.12369:49300 235.10822:25787 240.13489:65611 241.08347:54355 241.13728:5415 242.08473:13007 242.14868:35734 243.1313:4640 243.18166:6698 245.12105:24664 245.12833:64661 246.12497:17636 247.10829:28912 248.14044:5177 249.12204:6257 251.1028:19569 252.09825:18841 252.13622:17055 256.09271:26366 257.16153:20147 257.19556:24704 258.14615:15471 259.0928:25154 261.1619:15229	

					262.11719:19764 264.10864:4378 264.13211:26705 266.1517:21717 267.15109:6835 267.18423:4879 270.14496:40941 274.10034:6471 275.1059:4482 276.13452:60538 276.16934:24330 277.1163:8082 282.10919:4996 283.14523:94336 284.12421:6953 284.15146:14808 285.15353:14177 286.14252:7423 287.1044:4698 288.15417:68193 292.12924:7894 294.14053:35694 295.10202:5528 297.15671:28049 300.19119:7645 301.14932:25779 304.13095:30211 306.1434:8311 309.19382:4772 310.10336:6220 311.1366:47947 311.17303:33933 312.11743:35838 313.11871:7379 315.16748:26369 316.12506:5761 322.14276:20638 323.1376:5125 323.17065:7882 327.12973:7289 329.14795:16546 329.18192:18487 330.18112:19201 331.12775:5489 332.12433:4506 334.14267:5777 337.19308:5030 339.1691:6439 341.1445:6047 349.19025:4727 351.16656:5084 354.25116:19404 355.25241:4715 358.17245:6771 359.16455:21743 369.17529:5226 375.20184:20843 377.181:6518 379.16196:33264 380.16513:5151 381.17947:4492 385.23511:43641 385.73718:7670 386.68018:21985 387.18549:15517 392.19763:19351 395.19083:4545 398.20322:22083 399.19229:5268 412.17865:4900 416.216:5877 417.2164:6060 426.20111:8063 428.25314:4534 440.17111:16085 441.16251:16269 442.15726:4457 444.21011:5558 455.21671:5556 458.18802:5950 464.21878:6859 464.29468:15569 465.29257:13293 476.2088:16921 476.71185:18673 477.20581:15451 482.31262:36537 485.21118:36628 485.72192:18033 494.2283:12927 496.77026:5895 498.23312:5968 509.20117:14801 520.29877:6039 522.24017:4481 527.21045:7206 528.19916:5049 541.76422:7261 544.30859:5633 555.25006:5315 577.29053:4513 579.24243:5560 586.32751:5624 597.33295:7227	

					616.34241:12261 616.83612:7351 633.28583:5902 641.24371:30811 642.2522:18547 650.4024:13361 659.26758:35433 659.84833:58047 660.26685:13611 660.3634:34452 660.86261:22156 716.38647:43971 716.89716:30486 717.38745:6718 754.32758:7239 755.33069:20423 765.41241:5466 769.46655:40567 770.45813:16667 772.3479:12976 773.34631:4802 789.9256:17801 790.43121:15477 798.11646:5478 798.43732:4384 830.41443:5398 846.95288:15872 847.44397:20541 847.95898:5207	
Pyridoxamine	1.196	169.09485	[M+H]+	169.09718	53.42441:1803 54.48659:1841 56.7216:1628 69.71544:2896 79.82081:1794 80.99974:1764 86.06081:2155 96.06893:3079 100.75423:1842 105.00372:7893 109.0759:2912 123.01437:1875 126.10319:2397 128.01979:3660 142.03496:3120 146.02974:4204 147.0256:2142 151.08456:1903 152.06842:21112 160.04549:4118 169.04767:14132 169.09563:180852 169.33157:1841 170.02963:7271 170.078:13013 170.09483:13258 170.11798:4081	-13.77905888
8-HEPE	12.272	301.21567	[M+H-H2O]+	301.215	55.05515:13836 57.07082:3022 67.05527:40515 69.07078:16997 71.03793:2854 73.02883:2975 79.0546:31306 81.07038:51090 83.04979:3677 83.08641:13549 85.06547:8331 89.06052:2645 91.05511:57185 93.07066:53677 94.07334:3719 95.08624:45581 97.0652:3857 97.10149:3152 101.05992:3237 105.07011:44228 107.08642:34764 109.10177:22544 113.06055:2826 117.07038:65152 118.07387:4063 119.08554:69084 121.10128:57580 122.10458:3362 123.08038:4960 123.11665:7997 129.07027:12051 131.08516:78270 132.09003:7509 133.10086:78928 134.10402:9140 135.07994:9656 135.11725:39302 141.07013:4359 143.08588:12649 145.10197:67408 147.08139:9446 147.11688:46932 149.09639:20253 149.13277:10779	2.224324818

					153.09103:2784 155.08647:8859 157.10086:15171 159.11638:96728 160.12038:5353 161.09523:10644 161.1321:30381 163.11134:7955 167.10716:10262 169.1019:12662 171.11795:26349 173.09641:5356 173.1333:90998 174.13782:12362 175.11172:9976 175.1469:8829 179.10611:13229 181.10042:11631 181.12354:4129 183.11702:17124 185.13254:18623 187.11256:12755 187.14926:26803 189.12648:4768 191.10793:7530 193.12332:12893 195.11636:10674 197.13255:12129 199.11351:9337 199.14789:23393 201.1272:12511 201.16493:18580 203.14143:3187 205.12192:4659 207.13998:10368 209.13347:11093 211.14716:2948 213.1279:15922 213.16348:4895 215.14305:4274 217.12294:4893 219.13643:14028 221.15311:13581 223.14792:15572 227.1456:4493 229.159:2676 231.13919:10677 235.17053:8303 237.1635:10336 239.17841:5204 241.15871:5295 241.19423:11900 245.15385:9933 255.17464:12548 259.16812:13945 265.19461:31429 266.19714:2925 273.18604:4324 283.20465:38605 284.21066:9534 301.21509:478724 302.21875:87247 302.26892:11512 302.3038:14194	
Platifillin	8.07	338.19308	[M+2H]2+	338.19617	55.05515:6041 59.05006:9620 69.03425:212829 70.03784:4018 70.09457:9892 71.04986:3120 73.02944:41084 73.06554:11349 78.03989:5485 80.05533:9822 81.03431:11896 81.07029:3092 83.04981:41328 83.08641:3211 85.02901:4421 87.04442:222060 88.04812:4102 89.06052:614195 90.06366:25705 95.04921:16167 99.04494:99946 100.05318:31545 101.06088:3216 102.06754:4660 103.03871:3472 107.07078:22650 109.06508:3162 111.04474:81882 113.06054:14965 117.09076:4097 122.0649:56382 122.56656:4571 125.05943:14699 129.05495:32243 130.08571:26479 131.07077:37116 133.08617:233438 134.08929:10385 137.05942:9327 144.07791:35224	-9.136709029

					144.57951:3017 151.09686:15261 153.08385:8504 155.06989:69351 156.07303:4087 163.09554:3113 166.09142:29101 166.59206:5730 173.08109:25270 175.09634:31536 177.11319:41977 188.10446:3824 195.12152:6077 197.10892:12316 199.09752:15195 217.10776:13346 219.12404:24967 219.62334:2971 221.13751:3337 239.15034:12594 241.13741:3377 243.12064:4730 261.13458:23512 283.1774:9126 287.15067:3169 305.16034:14813 327.20285:4623 339.28778:3054	
PC(17:0/20:4)	11.424	854.59113	[M-H]-	854.58942	69.00755:4566 70.36697:3005 78.95736:66408 83.0229:13924 83.04802:4223 112.98354:3215 152.99422:12723 163.07616:3017 168.04205:97288 177.08932:3290 205.19606:22342 224.06918:59897 255.2318:23781 259.24277:94185 260.24731:4474 279.23218:25440 281.24823:89834 282.25302:12179 283.26324:724745 284.26514:54527 285.22208:8684 303.23325:1231674 304.2374:128486 305.24664:160490 306.25113:16013 307.26364:15575 331.26596:41472 419.2554:7207 490.32809:13575 506.33078:2872 508.33606:84118 509.34827:8952 510.29813:5100 528.30914:4467 794.5788:685599 795.56641:168417 854.60376:3273	2.000960882
Olivetolic acid	9.842	223.09665	[M-H]-	223.09763	56.02479:1368 59.01212:20909 69.88796:1275 69.89129:3272 96.95826:2035 99.92402:2032 107.08561:1744 109.03951:1241 110.74388:1212 123.89986:8960 125.89896:10223 135.11586:14834 142.77788:1323 151.07582:5957 161.09554:2863 176.9776:1626 177.08931:1665 179.10684:65520 180.10902:7470 193.86206:1270 196.06438:1451 205.08713:11398 222.97858:2772 223.0607:1705 223.09546:7937 223.13339:1712 223.79874:6910 223.84003:2763	-4.3926957
N-Lactoylleucine	8.31	202.10776	[M-H]-	202.10793	69.37273:5300 71.01201:17437 73.02772:2993 84.08075:19034 88.0385:6038 99.92403:3426 114.0909:45717 115.48579:2852	-0.841134734

					128.07028:5468 129.00182:5289 130.08577:254420 131.08905:10734 132.05524:2629 140.06953:2904 140.10724:32758 141.10985:2753 146.95972:3300 156.1028:9681 156.99838:26506 158.08221:8399 158.11804:293818 159.12048:20225 175.06178:4720 176.0457:4854 184.09659:5896 202.10867:1394512 203.11002:108476	
Bicifadine	12.198	174.1277	[M+CH3OH+H]+	174.12773	57.03444:3240 57.07092:13261 65.03959:1579 69.03477:1592 71.02998:2526 72.08164:2594 73.02941:1859 81.07029:3029 82.06589:2311 84.04491:2805 84.08145:7603 86.06078:6226 86.09714:7043 87.0445:10240 91.05514:2682 95.08612:2116 100.07568:3532 106.06584:9844 112.07631:22279 114.05557:3536 114.09139:2000 118.0651:47186 119.06815:1930 119.08541:3196 128.07074:16110 128.10757:14798 130.06616:2640 130.08586:5550 131.07365:5025 132.08139:176255 133.08475:9406 133.10077:2183 142.07828:1810 144.08095:15707 145.08865:28555 146.05983:3320 146.0816:14617 146.09666:10006 147.05431:1770 147.1169:2268 156.06554:6418 157.09695:3117 158.09682:11437 159.1049:43535 174.05502:9051 174.0768:16920 174.12692:1241499 175.05019:2096 175.12054:206889	-0.17228732
6,10,14-Trimethyl-5,9,13-pentadecatrien-2-one	11.9	263.23709	[M+H]+	263.23749	55.05515:9319 57.07091:22195 67.05482:50329 67.87881:12963 67.88306:8187 69.07078:18975 71.04986:2909 71.0864:13267 73.02943:5299 79.05489:10182 81.07043:103046 82.0659:2924 82.07365:2316 83.08642:24641 85.06547:7022 85.10194:5276 91.05535:10368 93.07086:23148 95.08626:103774 96.08946:5217 96.84789:3506 97.0652:8263 97.10168:12545 99.08051:9253 105.0702:15675 107.08669:24318 109.10194:72926 110.10519:5673 111.08026:6276 111.11691:3041 113.0959:7564 119.0854:23833 121.10135:34960 122.10458:2286 123.11681:35260 124.12036:3055	-1.519540397

					127.11244:2151 131.08505:1787 133.10106:31274 134.10403:1862 135.11737:31747 137.09596:6803 137.13248:16254 141.12735:6307 147.11717:29118 148.11882:1818 149.09651:2174 149.13304:27424 151.11272:4365 151.14819:11043 161.13228:26774 162.13579:2276 163.14917:23237 165.16362:18689 175.14909:22495 176.15146:1545 177.16246:5646 179.17888:9524 180.18169:2077 189.16341:12951 191.18062:2243 203.17989:6496 205.19438:2115 207.17392:1626 217.19579:3349 219.2103:6279 245.22701:124818 246.23178:17443 263.08188:16961 263.23581:233722 264.08435:1928 264.19836:2192 264.23911:39868 264.26761:39645	
Hexadeca-4,7,10,13-tetraenoate	12.491	247.17012	[M-H]-	247.17035	71.03399:1595 74.78416:1218 78.95699:1388 192.59601:1244 203.1071:2410 220.06122:1775 246.90631:2131 247.17145:310383 248.07321:1461 248.17328:42874	-0.930532323
1-(2,3-dibenzimidazol-2-ylpropyl)-2-methoxybenzene	13.081	381.17377	[M-H]-	381.17203	68.14579:18223 84.93729:23727 96.83885:3744 96.84428:3519 99.92403:430679 100.92431:22483 100.93201:71584 115.91891:236639 116.92683:767447 117.92742:34295 118.99126:2662 127.95625:5054 184.01842:3218 233.1037:9910 251.23822:11218 295.22708:4142 319.17523:2772 337.14127:3980 337.18173:34335 337.31158:4571 351.16116:29966 352.16846:8210 363.18387:74016 364.18246:12509 369.17636:49395 370.17438:9410 377.14127:14124 380.97668:14057 381.17407:1296050 382.17807:222555	4.564867994
Eicosatetraenoic acid	13.443	303.23254	[M-H]-	303.23294	59.01213:30354 65.01309:2766 79.95583:2435 96.84337:6815 106.03946:24152 134.05817:6714 143.03551:1520 177.09155:2140 194.97302:25583 196.97105:1541 205.19603:14217 209.9838:1736 231.21152:1760 259.24277:43696 260.24332:5515 276.09949:2952 285.22208:5916 303.02802:6794 303.23325:428017 304.03613:3361	-1.319117903

					304.2373:44573	
2-amino-6-{[2-(furan-2-yl)-2-oxoethyl]amino}hexanoic acid	6.45	253.11909	[M-H]-	253.11932	66.03323:3134 70.08177:5107 72.99163:2577 84.06215:1446 88.03851:9317 94.02834:171039 95.03159:6678 100.00176:1803 112.03956:1824 124.03862:349565 125.04214:16364 128.07027:32725 129.07413:1402 133.22006:1388 142.04933:2222 146.07999:1884 154.73012:1366 164.84836:1558 186.04028:1492 186.0788:1695 217.84875:1860 218.10179:23100 223.10794:62334 224.11029:7015 253.11818:596034 254.12181:68398	-0.908662365
(6E,8E,10R,12Z)-10-Hydroxy-3-oxooctadecatrienoic acid	11.707	307.19119	[M-H2O-H]-	307.19095	50.00297:1316 57.03292:1837 59.01195:1664 69.31139:1316 79.91451:1497 83.04875:2052 87.00722:1937 96.41994:1485 96.95828:1613 97.0634:1408 130.61208:1423 172.50877:1256 195.13817:1779 195.8277:1510 217.15816:1472 238.83418:1548 243.1738:5445 245.18938:64816 246.19398:5833 247.16776:1815 263.16248:2706 263.20322:35044 264.20618:2202 271.17154:7309 287.16452:1665 289.18283:112001 290.18341:13381 303.16821:1435 307.19296:378853 308.19583:44552	0.781273016
2-(3-methyl-2,6-dioxo-1,3,7-trihydropurin-7-yl)acetic acid	0.871	223.04553	[M-H]-	223.04732	59.01214:137198 69.94564:3932 71.01205:143016 72.99124:65150 73.02789:37137 75.0073:15648 78.95765:5362 81.03255:4408 83.01215:6237 85.02813:39842 87.00739:20338 89.02298:105665 90.02629:3444 94.92381:58209 95.01226:5070 96.84157:4607 96.92056:34320 96.96838:20691 97.02837:30420 99.00672:17074 99.04317:2855 101.02265:112232 102.95561:4911 103.00235:408309 104.00591:10362 105.01721:5480 113.02248:45696 115.00237:11306 115.03746:3015 119.03314:9805 125.0234:23887 129.01727:22847 131.03355:17181 133.01335:152386 136.91637:3469 143.03386:42736 145.0125:10674 147.02904:3700 154.89819:8287 154.92757:38500 161.04515:5665 163.0229:81305 164.83444:20761 165.03917:47328	-8.025202903

					176.83481:2871 177.04018:3637 180.85414:9889 186.8661:8927 198.84856:2938 205.03419:4318 223.04492:194232 223.83054:14580 224.04985:13355	
9,10,13-TriHOME	10.078	329.23312	[M-H]-	329.23279	57.0329:2940 70.08175:2224 75.43173:1449 79.73994:1513 87.03336:1344 90.93149:3392 99.0799:12336 113.25509:1329 127.11204:5655 139.11079:20285 157.1214:5090 171.10126:63424 172.10542:1738 183.13844:8296 193.12175:2397 197.06026:10124 199.13336:1583 209.11491:1983 211.13419:82575 212.13577:8361 229.14459:54767 241.05136:9162 282.36542:1744 285.03937:7295 293.21213:2112 311.22034:3312 329.23361:388091 329.67029:2413 330.23871:33249	1.002330296
(2S)-N-[(2S)-1-[(2-amino-2-oxoethyl)amino]-4-methyl-1-oxopentan-2-yl]-1-[1-(4-methylphenyl)sulfonylpiperidine-4-carbonyl]pyrrolidine-2-carboxamide	11.905	429.2818	[M+Na]+	429.28598	57.07082:9701 59.04963:2837 60.71284:3198 67.71434:45006 69.07069:2804 71.08621:4062 81.07098:4771 85.10193:4278 86.09714:4677 95.08612:3406 99.12354:2628 149.09648:4705 163.11133:10802 165.09135:67051 175.11171:3060 177.09088:4580 184.07443:13689 191.10789:3763 205.12189:4045 284.90262:2761 387.23618:207060 388.24179:24019 429.28067:3155687 430.28595:548770 430.3793:12299	-9.737098798
Corynanthine	8.071	355.22012	[M+2H]2+	355.2182	55.05515:2131 59.05006:3464 69.03428:102524 70.03783:3464 71.04986:2612 73.02944:52627 73.06567:12332 80.05532:6420 81.0343:7454 83.04983:17705 87.04516:122748 88.04812:3147 89.06052:477223 90.06367:15628 91.07574:2603 95.05008:3843	5.405128453

					99.04498:64009 100.05337:12299 101.05991:2401 107.07061:16239 111.04469:54419 113.06054:8457 117.09196:2936 122.06584:25266 125.06068:13620 129.05476:22876 130.08696:12607 131.07089:19870 132.07851:2204 133.08614:241250 134.08913:12289 137.06094:7176 144.07787:23994 144.57951:4080 151.09708:15356 153.08383:6272 153.58626:2348 155.06993:48924 156.07487:2118 163.0975:2773 166.09103:14468 173.08131:16437 175.09595:30355 177.11302:57026 186.60382:3179 188.10445:10316 195.12424:12942 197.10863:28382 199.0975:9797 210.1179:2857 217.10579:13674 219.12363:52246 219.62642:9195 221.1375:2855 239.15013:15705 241.13663:38135 241.63892:9911 243.12242:12626 261.13281:18076 263.15009:26802 263.65341:3060 283.1767:20735 285.16278:10541 285.66583:2168 287.15067:7236 305.15945:19769 307.17743:3421 308.90027:9703 309.90161:3128 316.18387:7861 317.18335:2249 327.20285:10836 331.17361:2754 331.91791:2976 338.19186:41872 338.69424:18717 339.19888:2619 349.1904:8475	
4-Acetoxy-2-hexyltetrahydrofuran	11.427	213.14865	[M-H]-	213.14906	59.01216:185879 60.01562:1292 65.01261:1677 71.02433:1366 79.95517:2225 83.02364:2434 87.00645:1810 94.89994:1257 99.92404:6704 106.03957:14652 111.52507:1306 114.93456:6836 115.91901:2501 116.92695:5242 123.89991:1487 133.06429:14417 144.05501:1518 151.11108:2350 160.04962:2026 168.09953:1404 169.12117:7668 169.15872:2156 170.11848:1555 171.06734:4776 173.04512:1321 174.40678:1226 187.06009:2627 197.9897:2266 212.83502:2098 212.96466:3008 213.02068:2872 213.09441:2060 213.11211:8353 213.14772:61107 214.01494:1799 214.05057:5380 214.07137:2882 214.13376:9714	-1.923536515

Serotonin	6.256	177.10236	[M+H]+	177.10228	55.93535:58359 68.72691:121200 88.02227:21129 115.05445:60752 128.95065:127049 132.08151:222099 135.94469:57582 146.96135:77047 148.07608:25789 153.95518:33722 160.07619:16873158 161.07982:1359692 169.97704:17183 177.10208:24945 178.05141:25422 178.08746:59479 178.12354:29601 178.15962:30333	0.451716375
Cellobiose	0.943	377.08484	[M+Cl]-	377.0856	50.75824:2693 59.01214:42036 63.119:3519 69.56877:6943 71.01202:49563 73.02788:64963 81.03323:2765 83.01213:12478 85.02797:3399 87.00719:5777 89.02299:81867 92.92628:47447 94.92359:5218 95.01225:3326 96.84155:3670 96.96822:3439 97.02828:36972 101.02267:99008 113.0225:48079 115.0386:13755 119.03347:20561 119.09719:2490 122.70519:2673 125.0232:4666 126.60522:2799 131.03352:5121 143.03409:40095 149.04474:3166 150.88577:6496 161.0433:202861 162.04803:4533 165.78575:3042 179.05458:148633 180.0563:2721 184.73073:2933 203.05489:2969 221.06462:5293 237.26309:2764 260.88556:3954 263.07739:2882 278.15155:3192 300.83856:9881 313.86679:3319 341.10938:5668 377.08542:346166 378.08536:21136	-2.015457498
PFCA-diether_Hsubstituted	2.393	411.95892	[M-H]-	411.96219	50.74283:1284 52.56928:1204 69.26982:2105 69.2731:3257 87.92345:2779 103.91849:15035 104.92643:6587 121.99797:6410 122.25398:1227 123.94439:1948 124.01378:1391 127.93019:9429 129.92111:3153 131.93733:12131 139.92972:1671 149.93756:2001 156.93297:1403 156.95721:1621 157.94099:11781 158.93776:2796 161.96007:5552 165.00504:11689 165.93369:1902 166.0128:12541 167.01889:11115 172.95232:2196 173.93588:3054 189.87219:1680 189.95659:1766 190.82866:1294 199.92468:1418 199.94077:9531 200.9474:7130 225.94157:3340 236.93474:12184 236.97182:40808 237.94351:15882	-7.937621654

					238.94974:7400 243.6781:1427 251.99535:1444 253.93767:5942 254.98096:9106 255.95288:33625 256.96185:5472 273.96198:20497 277.97488:21951 279.98996:17510 295.98392:9036 297.99982:11239 307.96045:1637 308.95712:72260 313.99258:15528 321.96243:3190 326.96777:2664 339.97107:8370 344.95383:1534	
1H,1H,2H,2H-Perfluorohexanol	1.636	263.00821	[M-H]-	263.01239	69.07664:8104 89.02298:327551 90.02546:7635 130.96565:13276 142.96413:3093 144.94458:6934 144.98112:46065 148.97574:7592 152.89377:47590 160.95406:3364 162.99133:39644 170.95897:38219 172.93965:26197 172.976:188896 173.97935:6606 188.9698:24312 190.98634:264543 191.98897:3232 200.97115:19000 203.01372:2875 208.88245:23266 212.97049:2918 214.98607:99647 215.98825:3071 216.96701:7472 217.00337:54835 218.98174:54552 222.86177:2721 232.99582:249121 234.00073:16597 234.91667:6176 234.97473:8787 235.01241:299252 236.01665:14071 262.89069:44191 263.00861:641216 264.01059:34788	-15.89278741
4-Methylproline	12.288	130.08627	[M+H]+	130.08681	51.79002:1898 55.05515:2271 56.05014:16720 57.07082:7724 58.02976:1826 67.05524:3760 69.18135:10218 70.0662:3518 71.04986:10221 74.09692:2093 84.04492:628922 84.08146:280546 85.04314:2649 85.04828:31791 85.06547:9556 85.08461:13440 91.05515:9668 96.5152:2420 102.05581:3823 112.07617:34975 116.06176:4092 130.05072:89609 130.06587:16236 130.08589:178090 130.12247:6866 130.15907:8428 131.05374:3800 131.08932:8158 131.1178:2187	-4.151074194
Pyrroline hydroxycarboxylate	1.956	128.03375	[M-H]-	128.03477	55.94059:7580 67.28857:6375 70.36363:10657 82.0282:35461 84.04315:6855 85.028:14367 96.83978:8515 99.23698:6802 99.8625:6907 106.13558:5921 109.245:6564 123.75093:6559 128.0332:1634836	-7.966585952

					128.87686:12141 129.03658:72574	
Threonine	1.279	120.06585	[M+H]+	120.06548	56.05021:1592414 57.03448:93738 57.0536:50589 71.02657:8967 74.06068:2916198 75.04476:16912 75.06387:83307 84.04498:140292 91.05515:12407 93.07076:47266 102.05586:646790 103.05856:26173 110.07123:9622 120.04514:13917 120.05637:33507 120.06635:135586 120.08007:13450 120.08881:10449	3.081651779
[2-[5,7-dihydroxy-2-(4-hydroxy-3-methoxyphenyl)-4-oxochromen-6-yl]-4-[4,5-dihydroxy-6-(hydroxymethyl)-3-[3,4,5-trihydroxy-6-(hydroxymethyl)oxan-2-yl]oxyoxan-2-yl]oxy-5-hydroxy-6-(hydroxymethyl)oxan-3-yl] (E)-3-(3,4-dihydroxyphen	9.243	949.26349	[M+H]+	949.2608	69.743:9010 70.02947:11025 70.06583:160125 72.0818:209371 74.06077:35174 82.06586:3728 83.06124:4238 84.04501:290358 84.0816:396096 86.09727:365689 87.05611:33762 87.10062:4607 88.03946:8235 91.05504:20652 93.07063:2648 99.09169:3188 101.07159:133583 101.10807:11381 102.05587:163282 103.05453:14307 105.06602:65838 107.04955:2771 110.09747:3883 111.09242:4794 112.07616:4652 115.08718:11114 119.04963:11139 119.08167:14261 120.08129:451078 121.08466:11558 126.05477:9007 127.04987:4704 128.10768:3731 129.06586:25661 129.10222:435413 130.05064:40249 130.06471:4261 130.10551:4933 131.11774:11754 136.07549:203869 139.05002:3150 139.08578:4990 140.07074:17655 141.06529:5280 141.10184:36388 143.11827:3412 146.12845:4852 147.07625:3767 151.08623:2902 152.14325:13248 153.06581:10637 153.10353:9591 155.0826:60520 155.11729:29751 157.06142:12799 159.1124:4285 164.03387:8284 165.10281:36101 167.08234:14846 167.11732:10473 168.11345:4660 169.09737:40926 170.09262:4487 171.07729:20674 171.11337:11180 173.092:8385 173.12851:103996 175.08748:4319 180.07841:4597 181.06088:32939 181.09802:9700 182.09378:4505 182.12874:4809 183.07628:59964 183.11354:24222 184.07437:5437 184.10991:14039 185.05652:3685 185.09166:20138 185.13055:5000	2.83378393

yl)prop-2-enoate					185.16565:20669 186.12282:37607 187.10762:8475 188.10193:4815 189.08693:3317 191.10281:9753 194.0941:5364 194.13002:3580 195.07735:34829 195.11331:20198 197.12982:11488 197.16396:4649 198.08858:28398 199.07039:39101 199.10809:10273 200.14023:33879 201.12383:76062 202.08203:41113 202.11815:2905 205.09674:14417 205.13576:13436 207.11388:28434 208.11028:3432 209.09323:11589 211.10632:6664 212.10431:38218 212.13991:5010 213.08597:32772 213.16043:4913 215.13995:5384 216.09726:96912 217.08197:14889 217.13408:35214 219.11479:2767 222.12561:15041 222.16081:11425 225.12335:10083 226.11974:4622 226.15445:69608 228.13467:24024 229.11813:23291 230.1151:48256 231.09909:9725 231.14908:3895 233.16531:15010 234.12375:20173 235.10712:16811 238.11885:2923 240.1001:4298 240.13455:42742 241.08324:80679 242.08116:3761 242.1526:17441 243.13492:3676 243.18166:5113 245.12033:7167 245.12761:24003 246.12497:4278 247.10849:21635 248.14044:2760 249.12578:8651 251.10281:10628 252.13623:14708 256.0914:21708 257.16153:11902 257.19675:13761 258.14615:9572 259.08994:42228 261.1619:8135 262.12122:10612 264.13715:13404 266.15173:13587 268.13113:3340 270.14371:28502 272.12271:2713 274.10464:11186 276.13452:26793 276.17371:15917 277.1207:2976 280.13333:4926 282.10919:4067 283.14505:43887 284.12274:18353 286.14255:11865 288.15799:25335 292.0961:8169 292.12924:5030 293.15955:5214 294.14578:19349 295.10684:4608 297.15405:19524 300.19119:8870 301.14835:17872 304.13144:8879 306.1434:2877 310.10379:15488 311.14178:13045 311.17303:26415 312.12167:44731 312.1593:12176 313.11346:8416 313.15552:3395 315.16498:17025 316.1304:7927	

					322.14276:4871 323.1376:3464 323.17065:4833 325.19122:3816 325.70349:3988 327.13538:4571 328.11398:8691 329.18219:15244 331.12775:4932 334.14267:10552 336.24377:4347 339.1691:5371 340.20227:3523 341.15048:4706 341.18634:11095 342.14468:2688 343.20352:3217 346.17746:4188 349.19025:3841 353.18491:3069 354.24908:20387 359.16528:14922 360.15433:2854 362.13962:5255 363.20734:3140 369.18204:5009 371.27646:3121 375.20877:12635 376.18948:3693 377.181:7613 379.15881:22752 381.1795:3054 385.22952:18303 387.19272:11564 392.19766:4896 398.20322:11790 410.24109:5105 411.22794:2963 413.2522:2994 414.24194:3379 423.155:4068 426.20114:4203 428.25317:3115 430.23523:2927 441.15802:22990 446.20581:3402 464.3042:12535 473.23648:4885 482.31351:19658 497.27472:3236 513.31256:2747 526.23596:2831 528.19922:10300 544.30859:4323 555.25012:2896 579.24243:3117 586.32751:4445 597.33295:4929 611.32306:2848 616.34241:4298 621.32098:2851 624.22473:4772 626.31561:3223 633.37653:2841 642.23682:4047 650.27661:4852 650.40247:4037 659.86475:12871 666.88116:2794 716.40442:14312 737.32935:8486 742.40637:3004 755.32184:36505 769.45746:32818 797.77441:3099 836.41571:3905 846.97626:4529 884.50861:3009 889.97272:3044 890.45129:2787 924.48553:2632 928.81958:2901 936.51849:3195	
LPC(17:0)	11.107	568.3623	[M-H]-	568.36194	70.40786:81933 78.95718:1032885 152.99463:604894 168.04163:788994 224.06897:4193448 225.07114:236193 242.08121:898462 283.26315:43436308 284.26959:3771928 419.25537:211121 508.33594:24479998 509.34818:4663020 568.35333:200792	0.633399203
Chenodeoxych	7.745	471.24503	[M-H]-	471.24164	59.0121:16128 61.0279:7615 68.75758:8084 72.99159:12864 75.00667:3282	7.19376157

olic acid sulfate					96.83884:2318 101.02264:32244 105.0538:15633 145.36754:1635 208.79979:1494 471.24533:268535 472.24634:27899	
Stearic acid amide	13.31	284.29514	[M+H]+	284.29477	55.05515:9463 57.07092:45470 60.04541:13289 67.05472:3923 69.0707:20501 70.06564:4165 70.5296:3291 73.02946:27830 73.06554:2677 74.06083:23732 81.07029:8431 83.08628:16799 84.08145:9355 85.10193:13447 86.06078:9377 88.07639:103582 89.06061:46106 90.06373:3456 95.08613:4886 97.10148:14293 100.07663:4331 102.09206:54014 114.09139:3537 116.10681:23594 130.12245:3763 133.08649:21316 144.13866:3655 176.14258:13244 195.41653:2817 200.1998:3277 239.15071:20738 240.15312:3010 252.11369:102606 253.11884:12749 266.28366:2463 284.29697:1531282 285.29971:263466	1.30146608
PS(16:0/18:2)	12.297	758.49866	[M-H2O-H]-	758.49774	78.95716:312709 96.96841:45652 134.98291:9186 152.9942:546129 171.00385:15855 253.21727:1199120 254.22148:82439 281.2478:466440 282.25229:29334 389.21075:368104 390.21036:24018 407.21786:146001 408.22531:8848 417.24194:84666 418.24725:9458 435.24881:17274 671.45923:515500 672.46765:76238 758.49011:140515 759.49854:27680	0.487806331
Mmv676063	7.732	490.28644	[M+NH4]+	490.289	59.05007:23874 69.67625:8155 73.06554:11665 77.06059:3115 87.04443:163280 88.04812:2784 89.06052:479440 90.06374:11670 91.07658:4708 103.0397:649738 103.07536:15458 104.04304:16880 107.07069:16321 117.09196:7915 119.07063:7514 121.08614:16262 130.08588:9111 131.0708:57962 133.08617:344960 134.08929:13698 147.06615:63988 147.10168:2458 163.09785:21916 175.09602:42374 177.11304:108850 191.09239:37964 195.12411:3986 207.12299:21494 219.12413:12019 221.13725:20772 235.11908:50296 239.14685:3610 251.14822:4125 265.16217:3205 269.15857:8031 279.14447:9778 283.17743:9358 333.89966:2896 367.19888:2605 371.22916:3542	-5.221410229

					385.20624:3041 415.2515:2875 427.25488:9242 429.23846:7505 455.25378:7551 473.25598:85887 474.26343:16472 490.29242:8222 491.29269:3297	
Indole-3-acetyl-valine	8.638	273.12357	[2M-H]-	273.12442	59.01237:6778 67.01716:2394 69.83038:3616 95.04827:16876 99.92403:2785 107.04884:2225 109.06436:3301 110.02345:2417 110.05957:6144 120.04331:13127 121.05137:6638 122.05927:23130 123.03074:69587 124.03866:8244 130.98242:14445 134.05963:100031 135.06339:6628 136.03925:195060 136.07536:383306 137.04745:69195 137.07896:25081 138.05392:192314 139.05806:11719 139.07489:2502 146.05989:3048 148.07617:2524 150.05415:17194 152.07036:36874 158.97757:33034 159.0556:47948 159.1013:3195 159.97856:2831 160.06303:7390 162.05588:3533 162.09111:6989 187.0625:3721 187.11104:3184 188.07115:2264 189.07812:6147 197.08372:13109 200.07219:2466 201.07988:37476 202.08418:7544 202.12233:9237 212.10649:90390 213.10913:8760 214.08618:6782 215.09372:16406 225.06474:8993 226.07341:37714 227.07642:3261 227.11536:4467 228.10217:6729 230.11816:85635 231.12152:9086 239.08289:3702 240.08868:77707 241.09401:10089 254.10658:2279 255.11218:440404 256.11673:58788 258.09964:10569 273.1257:74311 273.16669:21705 274.12189:6697	-3.112134755
LPC(20:5)	11.616	542.323	[2M+H]+	542.32465	58.06666:22599 59.07416:4349 60.0816:186410 67.05523:22197 69.07068:4579 71.0737:35407 79.05528:19875 81.07097:6347 86.0971:624915 87.10064:14473 89.0605:5083 91.05524:22720 93.07066:21751 95.08611:7901 97.06518:4932 98.98515:12450 104.10736:2697837 105.07004:15725 105.11081:76341 107.08635:5119 109.10179:5823 117.0703:6950 119.08662:6314 121.10111:4938	-3.042458055

					124.99972:233632 129.05493:7523 129.07022:6042 131.08644:12816 133.1022:4531 145.10025:4305 146.98158:15962 163.01651:4333 181.02612:23903 184.07433:3339052 185.07832:82308 199.03621:5686 258.10995:40761 359.26151:7176 483.24417:18524 524.31744:197961 525.31995:43259 542.3269:770509 543.32385:140650	
Uridine	4.049	243.06206	[M-H]-	243.06223	59.01237:1625 61.98682:1386 66.03332:23186 68.0126:2502 70.12019:1669 71.01184:1988 82.02821:47487 84.04388:2019 91.68288:1359 94.02829:1629 96.0439:2354 96.32214:1386 99.92403:5261 108.04436:9155 110.02339:373920 111.01854:21222 111.02631:16916 116.92694:1651 117.01813:6139 120.04339:1673 120.43604:1325 122.02334:20065 124.03867:9022 126.05446:1458 132.02934:2172 138.01852:3068 138.05544:1849 140.03334:52439 146.95961:56692 147.21013:1367 152.03316:73645 153.02808:36782 154.95856:1584 174.95404:106500 175.91507:1627 175.95935:2399 182.04568:5713 187.97824:1759 199.97563:2774 200.05592:166734 201.05821:9390 229.95625:1670 243.06216:136512 243.15941:2822 244.06496:12614	-<br>0.699409365
Histidylproline	6.642	253.12767	[M+H]+	253.12949	59.05008:171110 68.60433:30413 77.06058:20190 81.07098:6046 87.0444:498891 88.04811:15271 89.06051:1093166 90.0636:44726 95.08612:6418 103.0397:1595322 103.07532:96651 104.0429:61816 107.07059:59553 119.07061:49077 121.08601:394289 122.08921:20566 130.0874:23703 131.07076:344909 132.07426:23056 133.0862:662625 134.08939:37398 144.06647:5132 147.0661:97147 151.09727:52236 163.09779:40891 165.07504:5103 177.11301:22322 186.03426:47137 191.09286:6864 195.1241:12359 207.12294:276952 208.12735:22959 209.1019:5057 230.02341:7831 235.1192:36106 253.12694:54790	-<br>7.189995919
8-({2-[4-(tert-	7.367	385.20718	[M+H]+	385.211	59.05006:49577 73.02942:2409 73.06554:7095 77.06059:11453	-

butyl)phenoxy]ethyl}amino)-1,3,7-trimethyl-1,3,7-trihydropurine -2,6-dione					87.04442:267306 88.04812:9625 89.06052:634924 90.06374:19911 103.0397:835280 103.07555:31101 104.0429:26743 107.07076:32199 117.09196:3193 119.07063:8567 121.04933:3210 121.08609:24581 130.08588:17523 131.07072:112464 133.08618:337520 134.08983:14552 144.06647:3153 147.06448:72293 147.10168:7287 148.06923:2223 163.09778:25161 165.11328:2272 175.09627:43043 176.10046:2034 177.11308:84194 178.11678:3061 191.09225:18978 193.10803:2674 195.1241:2895 207.12288:16888 219.12411:8265 221.13751:4263 235.11862:14424 251.14821:2636 253.16469:2615 283.1774:2569 339.19888:2798 385.20621:9470	9.916643087
Asparagine propyl ester	1.407	175.10757	[M+H]+	175.10768	60.05647:730474 62.98244:13647 68.05045:8019 69.5567:33442 70.06616:1351358 71.06918:31495 72.08168:63374 84.04491:30671 84.08137:95325 86.99348:71402 87.04441:14115 98.06049:10751 111.05584:30758 112.07607:938166 112.08666:72898 113.07195:69763 113.07993:38357 114.05559:11875 114.10297:24230 115.08694:67431 116.07108:803955 117.07394:23188 129.06609:13994 129.10214:225193 130.0508:44495 130.08728:58448 130.09854:255555 133.92892:7137 134.01845:299722 141.06694:6515 151.94061:8392 157.06137:49942 157.11005:38491 158.0305:50021 158.09276:222405 159.07608:128765 175.07217:82434 175.12038:988588 176.04066:126409 176.06944:8520 176.10712:14404 176.1244:44111	-0.628184897
Hexanoylglutamine	6.227	243.1348	[M-H]-	243.13503	58.02818:6656 59.01191:1615 66.03321:10875 68.0489:13653 68.4422:12222 82.02814:2831 84.04383:10469 86.05972:2963 88.98698:1584 96.83789:1634 105.31992:1447 110.02229:2100 112.07549:2810 118.96535:1800 128.03447:2078 129.10185:1635 130.04909:258288 130.08549:7072 131.05197:7533 141.10181:1802	-0.945976398

					143.11832:235459 144.12186:10635 146.95961:34984 153.03003:6280 171.1118:260653 172.11678:16889 174.95412:57115 179.11809:2993 181.13348:278632 182.13676:24566 199.14395:1648 225.12547:2194 225.14789:1560 243.13405:647632 244.13712:50986	
Creatine	1.399	132.07704	[M+H]+	132.07678	68.05045:91936 69.66024:154393 86.06078:255108 86.07214:285087 87.05592:1759934 90.05559:41610756 91.05908:910907 114.06704:1000437 115.05076:612420 132.077:21774064 133.08144:478513	1.968551929
Tetradecanedioic acid	11.253	257.17545	[M-H]-	257.17581	59.01235:2308 85.02796:7829 96.83701:7729 96.84425:7307 99.924:10507 116.92705:34616 117.92738:2639 145.93938:2771 195.17441:440976 196.17657:36618 213.18605:17277 225.44624:2309 239.1637:744246 240.16643:86276 240.20523:2291 257.17685:1503808 258.17737:174032	-1.399820613
[3,4,5-trihydroxy-6-[3,4,5-trihydroxy-6-(hydroxymethyl)oxan-2-yl]oxyoxan-2-yl]methyl (E)-2-methylbut-2-enoate	9.839	469.15366	[2M-H]-	469.15631	69.27637:4481 71.25766:1446 96.84067:1890 99.92403:3218 103.91843:5902 104.92642:1603 115.919:10038 116.92725:11269 155.10684:2180 159.04233:1598 174.95419:6979 195.00894:2262 211.04117:3337 223.00687:22288 223.04178:7605 225.0201:95784 226.02533:6522 241.01593:5621 249.02086:7033 265.01855:7293 267.03094:290434 268.03555:19411 269.01175:57682 270.01477:6991 335.13062:2409 349.11087:2348 351.12402:1527 361.1106:2671 379.12186:115998 380.12433:14922 381.13898:2405 397.12973:7440 405.13812:20148 423.14813:18084 425.16522:51881 426.17093:2315 433.13037:7014 451.13983:2876 469.15009:32154 469.30124:1806	-5.648437298
3,6,9,12,15,18,21,24,27,30-Decaoxadotriacontane-1,32-diol	7.775	503.30737	[M+H]+	503.30618	59.05006:5039 69.88244:8853 73.02882:3090 73.06554:39067 87.04443:185751 88.04811:3195 89.06052:2096301 90.06377:46297 91.07574:11242 107.07069:52115 117.09188:18840 130.08591:83798 131.0708:52495 133.08623:1185843 134.08916:42290 135.10225:7653 151.09685:3665 160.97311:2194 175.09648:22424 177.11327:275918	2.364365961

					178.11674:4780 179.12881:2698 195.12151:2509 221.13745:34487 239.15056:14136 265.16214:3367 283.17737:13005 299.05994:2420 327.20282:4666 371.2291:2448 415.2514:2295 459.27844:9771 503.30225:19704	
Icosa-2,4,6,8,10-pentaenoic acid	12.49	301.21701	[M-H2O-H]-	301.21732	57.0329:3152 59.01218:95770 67.05358:3225 69.03255:8783 83.048:2845 93.06886:3035 106.03916:3148 107.08455:6166 113.09522:1932 117.06853:9140 119.08486:3533 121.10062:1914 127.11069:7429 130.03912:1614 131.08475:1709 135.11696:3043 141.09073:1616 147.11696:1678 149.13106:6321 161.13237:1848 163.14751:10092 175.14745:2241 177.09152:1558 177.16307:8390 189.16441:5247 203.17863:102681 204.18173:9916 215.18065:5576 223.17131:8089 229.1946:14607 255.21272:3000 257.22766:345568 258.23218:42907 271.20532:8383 275.10593:2090 283.20432:7995 301.21515:532264 302.11447:1676 302.21887:69281	-1.029157287
4-((9S)-3,5,14-trihydroxy-10-((E)-((1-hydroxybutan-2-yl)imino)methyl)-13-methylhexadecahydro-1H-cyclopenta[a]phenanthren-17-yl)furan-2(5H)-	11.524	498.28238	[M+H]+	498.28262	55.05515:15606 57.03453:33880 57.07086:171375 60.04543:98931 67.05528:32721 69.07075:42399 69.93123:7110 70.0295:17995 71.08623:169686 81.07034:60225 83.08637:62656 85.10196:112079 88.04028:770738 89.04299:7479 89.06052:8701 95.08604:117204 97.10141:38423 98.98513:130666 104.10737:14410 106.05036:418395 107.05379:5871 109.10178:67232 111.11691:14091 123.11658:39864 125.13242:6331 126.02262:15809 137.13249:19074 155.0114:371855 173.02078:116638 186.01532:7572 221.22798:6625 239.23788:76966 242.04198:16422 257.24771:13434 260.05328:26564 280.26315:24753 313.27148:1542317 314.27814:179772 319.24527:4531 326.26758:159186 327.27023:15636 337.25195:6098 393.23883:229364 394.24725:27774 462.26376:25084 480.27579:14910	-0.481654367

one						
5-Hydroxymaltol	4.626	143.03406	[M+H]+	143.03444	55.01875:19385 58.06583:45724 69.03434:60577 69.76735:9108 69.77011:18143 70.06564:41557 71.04986:36607 84.04491:40460 84.08154:1266400 84.96065:13415 86.06078:11433 97.02893:17483 98.0605:125337 98.09734:163984 99.04498:9400 115.08723:11275 116.97171:76145 117.98022:14268 125.02348:85424 126.09108:41112 143.03392:2026267 144.03696:113164 144.06647:50068 144.10251:878085 144.13866:10650	-2.656702819
2-Naphthol-6-sulfonic acid	10.642	223.00594	[M-H]-	223.00702	69.81378:6058 79.95583:10799 116.92694:5451 123.89989:17386 125.89922:14472 143.00633:6857 143.04851:470942 144.05171:32998 179.10684:7505 187.00186:5728 194.9857:2119 222.98178:59882 223.00703:309898 223.0955:2657 223.7981:14624 223.98625:3907 224.0085:28816	-4.842896874
Octadecylamine	11.483	270.31546	[M+H]+	270.31549	53.00286:4209 55.05515:3145 57.07092:289848 58.07423:9925 67.05473:3052 69.07069:9678 69.48743:8311 71.08632:268823 72.08978:12727 81.07098:3132 83.08641:4855 85.10202:159865 86.10548:11946 95.08613:8791 97.10149:3431 99.11794:4754 103.97753:2108 109.10074:4693 113.13241:2575 126.46117:2123 191.99548:2733 255.10506:3948 270.13281:4239 270.31406:1002086 271.20557:3232 271.31989:161158	-0.110981432
[Gln1-Val9]-moroidin-[QLLVWRAH]	11.993	559.31396	[M+H-H2O]+	559.31689	55.05514:11532 56.05058:164976 57.03444:8296 57.07081:3408 60.0816:3868 67.05523:53070 67.63124:36280 67.63547:8916 69.07079:22541 72.04509:569631 73.04835:9627 74.0606:75659 79.05531:29152 81.07097:43900 83.08639:5307 84.04481:32261 85.06544:8056 86.09702:31519 89.0605:5319 91.05508:41628 93.07069:38189 95.08601:40395 97.06518:12418 97.10146:3470	-5.238533026

					101.07147:2455072 102.07221:12981 102.07515:44551 104.10732:35631 105.07019:27654 107.0862:30307 109.10184:15951 117.0703:14666 119.08109:206823 121.10105:20834 123.08035:4284 123.11662:3666 124.99974:3100 129.05481:23655 129.07022:3512 131.08644:19080 133.10065:27635 135.11713:8487 143.08583:3364 145.10025:11010 147.11729:15718 149.09647:3789 157.10066:11552 159.11624:11060 162.95546:15419 171.11766:11657 173.13309:3848 175.14685:8578 181.03798:4436 184.07426:48717 185.13298:3027 189.16344:5027 199.04951:8721 201.16502:5130 203.17976:28152 256.26437:12720 257.26724:11459 269.22525:16047 273.0838:22041 361.27191:257459 362.27765:35679 387.3013:45411 388.30798:4089 500.22211:13094 541.30975:19605 542.30292:4492 559.30615:33286 559.44452:18529 560.31012:4930	
Patulin	6.274	153.0155	[M-H]-	153.01932	54.39974:1380 67.02861:1418 68.0126:2883 68.99668:4359 69.03311:2298 71.01184:1416 81.03255:2006 82.02823:12838 83.01215:1587 83.04874:7430 83.99129:1259 85.02799:3139 87.04353:40626 88.04634:1528 91.02864:1924 97.02804:1430 102.98733:1604 108.98663:2143 109.02786:15575 109.03953:2734 109.06437:6274 109.99937:1950 110.02345:4490 110.05958:2845 111.01854:5320 111.04398:12119 112.03843:2984 112.98355:1604 114.69069:1338 125.0219:3086 125.03516:1805 125.05904:1456 126.01822:5712 126.05445:7014 127.036:2301 135.01874:3215 153.01578:170758 153.05557:18365 154.01338:5371 154.04964:9847	-24.96416792
3-Aminopicolinaldehyde	3.83	123.05556	[M+H-H2O]+	123.05529	53.03948:17550 68.05045:19773 69.12845:50588 78.03466:48962 80.04994:1020894 81.05369:24897 95.05012:170787 95.06065:22735 96.04493:466014 106.02857:59669 112.04023:73498 123.05579:6492622	2.19413566

					123.09112:98489 124.03916:915542 124.05883:390642 124.07579:64622	
Ssr-241586	10.462	601.26642	[2M+H]+	601.27069	55.01874:7711 69.97449:9048 89.0605:8565 136.07553:15799 174.09201:7350 191.11784:63373 210.08897:10625 212.10771:19684 213.10146:7247 224.10805:219127 225.10062:142621 226.10689:13067 227.11653:154715 228.12279:17082 238.12239:19963 239.11775:39205 240.08955:19569 241.09875:68082 241.1337:78802 255.11281:1782196 256.11627:157552 257.1265:82124 266.11838:28558 267.11389:150631 268.11868:14534 271.14233:32577 273.12601:16052 281.12711:35251 283.10959:256481 284.13095:114635 285.12167:695311 286.12912:78099 292.11984:7689 299.13852:419197 300.14694:381772 301.11581:1633516 301.15546:876267 302.11935:167860 302.15921:98105 305.12915:48628 313.15558:13305 319.14246:22284 320.11737:25538 321.12131:22998 323.13766:15693 333.12027:30012 347.13925:54758 351.13492:79620 365.151:64632 375.13431:61125 376.12036:6736 393.14304:146500 394.14987:18724 411.15714:260317 412.15598:33022 420.19046:12559 466.1983:15099 601.27087:580250 602.26642:149151	-6.70247206
Maackiain	8.624	302.10263	[M+NH4]+	302.10233	67.05627:5635 67.60646:39395 93.03575:4291 95.05095:5580 111.04582:4132 120.04636:12705 121.03036:9685 123.04539:5642 134.06129:14956 136.07564:36382 139.04073:15001 146.06149:6310 148.04016:5403 157.10254:3368 160.07616:6333 162.05418:82509 163.05995:5682 169.08934:4245 172.04062:3900 172.11211:21846 174.05499:6095 180.067:15125 182.09616:4380 184.07678:6015 184.11232:15730 185.09715:9504 185.11626:4624 186.09146:43434 188.07234:29234 192.30121:3472 196.07625:5767 197.08525:10348 198.08885:5457 199.09747:4417 200.07135:32010 200.10825:85270 201.07576:4867 201.11092:4558 210.09142:39694 211.07729:6097	0.993041

					211.09764:18730 212.10773:14208 213.09557:11127 214.0517:3828 214.08734:93791 214.10518:30051 215.09213:11140 224.06995:13431 227.07751:26063 227.11633:10314 228.06602:46124 228.10349:204195 229.10684:27687 238.08755:28138 239.06964:4256 239.09067:13756 241.07884:4516 241.09659:75637 242.10034:248088 243.11259:46965 255.11349:46458 256.09705:2420881 257.09955:340894 258.11081:9474 258.1344:9570 266.08179:5338 267.08493:5811 274.10519:36693 275.11032:3424 283.10504:4427 284.08795:156740 285.12183:58473 302.09943:585603 303.10397:91120	
8,12-Octadecadienoic acid	11.907	281.2478	[M+H]+	281.2475	55.05524:33444 56.05856:1546 57.03445:3216 57.07086:37894 67.05534:24209 69.03424:8905 69.0708:86098 70.07399:2717 71.04986:6596 71.08633:22053 73.02883:5883 79.05463:7004 81.07038:46035 83.0498:6383 83.08649:76535 84.08951:3331 85.06547:5526 85.10194:8968 87.04441:7320 91.05515:8246 93.07101:12435 95.08624:60281 96.08946:2754 97.06525:10749 97.10162:71064 98.10471:5148 99.04498:2202 99.08144:6457 105.07032:10368 107.0865:30100 109.06509:2158 109.10182:42447 110.10519:2078 111.08138:6754 111.1171:32832 112.12125:2392 113.06055:1564 113.0959:2799 115.07553:2262 119.08541:21191 121.10116:23433 123.08038:6829 123.11681:23976 125.09659:6707 125.13242:9011 127.07575:2028 127.11244:2004 129.09113:2094 133.06438:3328 133.10081:24783 135.07986:15145 135.11729:17313 137.09602:10385 137.13239:10250 139.1123:7164 139.14812:2357 141.09079:2566 147.11703:25204 148.12053:2086 149.13257:16067 150.07861:1719 151.11292:11371 151.14796:7140 153.12697:6155 155.10661:1573 161.13249:21492 163.1492:9328 165.12741:11104	1.066676148

					165.1636:5588 167.14404:2664 169.12276:1620 175.14908:15623 176.15146:1253 177.16246:1487 179.1425:6658 179.17888:3140 181.15822:5336 183.31415:1434 185.15453:1286 189.1637:12380 193.15897:7467 203.17989:3247 205.19438:2141 207.17392:7045 219.2103:5369 221.22504:12007 225.56941:1941 235.88016:1681 245.22688:55224 246.231:9339 263.16284:3192 263.2074:1911 263.23578:37552 264.23911:3277 281.15009:1885 281.21716:1596 281.24847:8978 282.22244:26108 282.28012:22250	
Picolinic acid	1.146	106.02685	[M+H]+	106.028	50.6792:1794 53.6056:1858 54.83691:2020 58.0734:1730 68.34638:14656 68.35067:3971 74.65968:1814 79.05531:2460 87.99409:4844 88.99123:3998 101.30706:1820 101.36898:1780 106.00454:18720 107.00132:13249	-10.84619157
methyl 2-{[4-(acetylamino)phenyl]carbonylamino}acetate	6.673	249.08769	[M-H]-	249.08801	53.46591:1086 69.7784:3415 78.215:1114 91.83211:1228 102.32246:1546 110.27125:1521 112.98356:2731 160.04002:6044 163.02682:1608 175.02666:5454 176.03465:4978 188.0345:4825 202.60564:1516 203.0824:4883 205.06207:6726 232.02397:1514 249.0097:1862 249.0507:2947 249.15138:1473 250.14653:8311	-1.284686485
PE(17:0/20:4)	13.013	754.53326	[M+H-H2O]+	754.53809	55.05518:105079 56.05018:43814 57.07082:382022 59.05005:14246 67.05524:197983 69.07067:270932 71.07372:197109 71.08625:269759 79.05528:32312 81.07101:308546 82.07363:30087 83.08646:291290 85.10203:189623 86.09713:2111552 87.10037:80294 93.07084:86988 95.08611:410756 96.08944:16065 97.10151:192122 99.08142:12846 107.08659:78692 109.10181:176814 111.11681:75142 120.966:20104 121.10113:122442 123.11658:96975 124.99974:17983 135.11725:133118 137.13304:74720 146.98157:5128354 147.98564:76329 148.98604:32807 149.13274:45888 163.01651:17131 164.99207:91303 179.98224:12732 184.07416:151937 188.00877:131961 207.99933:73897 219.21025:32011	-5.910901065

					237.22127:68552 239.23801:57896 439.22675:132470 440.22382:12955 441.24176:51080 476.31738:14697 478.33505:19790 498.29657:20230 549.43854:27951 549.48743:784864 550.48962:201483 569.45215:40086 570.45874:52534 571.46747:1748166 572.47949:422609 693.43927:14150 694.46222:16015 695.46948:1660498 696.46204:442337 752.52338:15637 754.54437:2100533 755.5481:681765	
alpha-Carboxy-delta-nonalactone	9.238	199.09653	[M-H]-	199.09703	59.01195:2127 65.01261:1645 71.01582:1989 83.02292:1405 83.04803:5566 99.00702:11689 99.07965:2827 103.91845:1664 106.0392:1495 113.03369:2034 127.04959:7153 128.0334:11029 129.98856:1342 137.09604:46999 139.29762:1455 155.10693:93522 156.10097:6173 156.11206:2774 157.05051:1987 172.04982:2610 173.04512:1654 181.08505:18885 186.08122:1549 199.04286:2419 199.09612:136560 199.13344:6140 200.05344:1760 200.09908:8259 200.1286:6852	-2.511338316
2-[4-hydroxy-3-(sulfooxy)phenyl]acetic acid	6.359	246.99152	[M-H]-	246.99124	59.01237:2065 69.59631:3330 79.95554:20561 80.96342:524920 81.03324:2451 81.96269:2734 93.03312:3133 95.01225:1945 99.04316:1884 99.92419:12641 103.91843:2735 108.01973:77770 109.02833:12958 116.92693:7655 117.05415:3450 118.94204:2538 121.02738:237791 122.03102:14932 123.04362:95230 124.04652:6502 137.02278:6654 146.95967:31524 147.04424:2200 162.93207:2748 164.07013:25759 167.03415:11473 180.06549:8069 187.1086:1865 188.97214:1947 201.00682:3289 203.00008:223902 204.00511:17243 246.99097:388064 247.99564:30618	1.133643444
(6R,8Z)-6-Hydroxy-3-oxotetradecenoic acid	10.954	255.15916	[M-H2O-H]-	255.15964	57.0329:8869 59.01236:2361 69.1224:8446 71.04873:1349 80.56824:1468 85.02797:2742 85.06441:8271 99.07961:3275 102.52003:1327 145.22469:1598 184.50233:1599 184.66644:1425 193.1599:119581 194.1618:14067 211.16893:16168 212.17096:1435 219.84456:2031	-1.881175252

					237.1487:161977 238.15292:20157 255.15868:436535 255.23117:20496 255.58824:1801 256.16278:51758	
Homocysteine thiolactone	1.019	100.02499	[M+H]+	100.022	54.03473:13412 55.01882:146246 55.05522:451660 56.05025:112700 57.07083:27637 58.02977:32369 58.06588:74796 59.05008:135607 59.93129:8401 65.90377:7881 69.03424:14996 70.34572:15049 72.0451:247964 72.08165:63085 73.02938:80436 73.06564:54007 82.01446:32828 82.02923:58939 82.06582:81259 83.01321:239405 83.04981:92937 83.08643:18252 100.02555:116017 100.03963:216190 100.07664:1118820 100.11275:285986 101.05981:70278 101.08017:37272 101.0956:9038	29.89342345
Benzophenone	11.948	183.08035	[M+H]+	183.08047	51.02413:12120 53.03947:10485 55.05514:15222 57.07082:5967 60.0816:13053 68.82912:24393 70.06619:14648 72.93775:16117 77.03888:29590 81.07028:15507 84.0449:5192 85.06545:4378 86.09714:54123 90.9481:6825 95.05002:81682 95.08596:24416 105.03429:4129710 106.03683:218021 109.1018:6168 111.04472:10502 113.96432:101058 118.96812:20622 123.04411:4791 124.9996:36729 131.97491:55629 137.13248:11257 138.0909:14308 141.95949:19508 141.98355:13786 147.1169:5288 154.99123:17493 155.08095:5064 156.1026:4255 159.96844:39457 165.05493:4642 165.12735:5238 166.0509:4541 166.08743:12057 183.01118:26992 183.08125:1001867 183.1377:13991 184.0061:21397 184.06956:111158 184.13365:16032	-0.655449486
Tridecanedioic acid	10.844	243.15993	[M-H]-	243.16013	51.90896:1328 53.52602:1282 59.01195:1444 71.01922:1448 75.4361:1449 99.92403:5672 106.0392:1482 110.21416:1389 118.80192:1381 118.85474:1276 123.8999:6573 124.15798:1223 146.95973:28046 148.05226:1492 163.07617:2046 174.95439:46250 181.15906:101511 182.16222:8094 185.11716:1580 199.17075:3175 225.14813:190311	-0.822503262

					226.15134:24894 243.15938:437535 244.16216:47251	
7-hydroxy-3-[(2R)-1-(2-hydroxy-4-methoxyphenyl)-3,3-bis(4-hydroxyphenyl)-1-oxopropan-2-yl]-4H-chromen-4-one	6.671	523.14111	[2M-H]-	523.13983	71.01241:2763 82.58911:1648 85.89192:1538 174.96956:2582 178.00481:1608 187.97826:3480 202.98909:5843 220.9743:10409 275.06274:2642 300.05826:2266 302.07465:32537 303.07309:2001 319.05066:117812 320.05322:8748 333.05753:23456 337.05914:12129 431.1283:349982 432.13165:53337 449.13861:36653 450.14182:5912 477.13672:14248 495.15189:3504	2.446764568
cis-Aconitate	3.24	173.00764	[M-H]-	173.00912	59.01192:6339 61.9868:6828 69.86469:5179 85.02796:428042 86.03082:12397 89.02285:7740 111.00733:45450 111.07956:43860 112.01141:1679 115.91896:6886 118.96381:13147 127.11204:1508 128.87665:220262 129.01826:101239 129.09077:2989 130.02084:2134 130.08562:9910 131.08189:5857 146.99518:1551 154.94569:5407 155.07016:1960 156.06577:9125 172.82936:6249 172.866:4588 172.93932:7804 173.00766:13781 173.08125:43982 173.11848:5835 174.07536:9649 174.11:1792	-8.554462331
1-Carboxyethylphenylalanine	8.604	236.09209	[M-H]-	236.09229	71.01229:13814 73.02769:6176 80.96339:3282 87.92333:29008 88.03848:842156 89.04198:21736 91.05329:36534 102.9469:18261 103.91843:82309 104.92638:6376 105.93463:2367 106.94186:15973 118.06476:3486 123.9443:20388 135.04399:7328 146.93771:38935 147.04416:111239 148.04707:8387 164.07005:60124 165.07336:3262 190.9287:12492 192.1028:17180 218.08063:1979 236.09236:700056 237.09711:67781	-0.84712635
6-Methoxy-	1.269	154.04732	[M+H-	154.05042	55.93497:5157 67.05526:6342 69.31909:22919 70.06566:9556	-

pyridine-3-carboxylic acid			H2O]+		81.07031:7681 82.06591:6955 84.04493:8417 84.08147:8571 96.92207:9448 98.0605:10790 101.8781:4937 108.04498:8430 108.08121:37568 109.0651:5706 109.10184:7846 110.0603:7036 112.03912:8009 112.07624:7885 113.06056:7210 126.05486:23331 126.07767:19703 126.0911:22482 127.08664:10654 135.94621:8336 136.07521:39332 137.06097:7620 138.01709:20005 138.05402:5006 153.95703:6118 154.04765:418072 154.07304:7945 154.08574:100924 154.12202:16276 155.04196:54381 155.07001:12526 155.08018:73734 155.11763:7438 155.15428:18787	20.12328172
12,15-Epoxy-13,14-dimethyleicosa-10,12,14-trienoic acid	12.729	347.25848	[M-H2O-H]-	347.25861	51.74722:1458 55.61776:1536 59.01213:13936 69.30152:3118 81.21965:1508 83.04873:2499 106.03917:6566 135.11697:2699 137.32141:1479 153.12711:6356 163.14726:9262 179.10452:1544 181.12198:2107 189.12743:3566 191.10643:1443 191.14403:1653 192.66187:1673 205.12329:2531 207.13857:233432 208.14323:19875 209.15508:3485 231.21149:5761 235.13174:23940 236.1405:18696 244.45192:1623 251.20419:1766 268.13095:2428 285.25879:36539 286.26196:2124 329.25052:170176 330.24921:19062 347.25607:276746 348.26492:31903	-0.374360768
St027719	8.399	317.11365	[M-H]-	317.11432	59.01237:7377 70.35578:3258 93.06887:3206 99.92403:3002 110.02344:6213 112.98354:2300 120.04337:7420 121.05137:3444 122.05933:94658 123.03049:27417 124.03865:8750 124.07535:2587 134.05966:53536 136.03931:263023 136.07544:465788 137.04796:30369 137.07916:23543 138.05392:170929 139.05779:6791 146.05988:2174 148.07617:2839 149.04639:100491 150.05449:25120 151.06174:2320 152.07062:15833 159.05563:3053 161.04707:2594 162.05411:12282 162.09109:10152 168.06601:22792 174.03163:6344 180.06549:7719	-2.112802727

					187.06248:8026 189.04359:2894 189.07751:21073 190.05089:26197 197.08385:6497 200.0695:3734 201.07968:49221 202.08417:9646 202.12233:2179 212.10651:60654 213.11208:5658 214.08618:2909 215.09384:28545 225.06473:2881 226.07365:8481 227.11861:2927 228.10217:5720 229.10858:3728 230.06871:9593 230.1181:111674 231.12151:6943 240.08858:86762 241.09401:2677 243.07668:8834 244.0858:27056 245.0916:32388 246.09505:2537 255.11224:299874 256.11871:21043 257.0943:14602 258.10278:14532 273.12576:227324 274.12769:23167 289.92108:2958 299.10315:17195 302.08963:6715 317.11398:243311 318.11758:24073	
Dehydrophytosphingosine - 2H	13.18	314.26959	[M+H]+	314.26898	50.4008:1746 54.36169:1706 57.07083:3060 67.05526:2954 69.07071:3182 71.03454:2031 71.08624:3026 77.67957:1818 81.071:4261 82.18591:1813 83.60384:2135 95.0501:2194 95.08615:2940 97.10241:1960 109.10184:4612 115.46052:1868 121.10242:2035 129.08975:1939 133.1008:2341 135.11569:2370 175.14912:2028 184.07449:9174 209.69395:1997 278.13873:1949 278.24875:3216 296.2305:3287 296.25949:21891 314.26764:56931 314.34164:7312 315.19446:3411 315.23694:4078 315.27408:12557	1.941012441
Acetaminophen	1.147	152.06833	[M+H]+	152.07059	56.52884:4119 65.92052:3863 69.65089:10031 96.08144:5182 102.02259:4254 106.00478:15808 107.00131:6983 110.0603:3955 119.03616:4092 129.02023:18584 130.01698:21199 134.00101:3875 152.00995:6956 152.03305:24229 152.0686:254002 152.10776:5644 153.00665:9294 153.05334:7313 153.06592:21982 153.36089:4437	-14.86151925
(2E)-1-(1,3-Dimethyl-1H-Pyrazol-4-Yl)-	9.601	317.14938	[M+H]+	317.14957	120.08131:3005 152.07036:3882 196.11273:2886 198.09154:6594 198.11533:7067 199.10017:2483 210.0921:55751 211.09767:9169 211.12386:8325 212.10776:16385 212.1312:12687 213.10149:10020	-0.599086418

3-(3,4,5-Trimethoxyphenyl)Prop-2-En-1-One					214.10817:2451 223.09973:2675 224.10825:17926 225.10251:16242 226.10706:17652 227.09364:10453 227.1196:50829 228.12283:6907 236.10706:3492 238.12244:7563 239.11783:25723 240.10019:22173 240.12489:27915 241.13383:5922 252.10237:13964 253.13029:3157 254.11998:27798 255.11664:9373 256.12051:8987 257.12854:19694 259.14435:2892 269.13019:14925 270.11148:42695 271.14182:146818 272.11429:44561 272.14835:17432 273.11752:3758 282.11411:22741 283.10962:3685 284.11514:46763 285.12259:18164 286.12695:13349 287.13687:93325 288.14102:12570 299.13843:1293996 300.14203:199358 302.12476:12510 317.11905:5500 317.15118:13943	
PC(16:0/18:1)	12.47	760.58472	[M+H]+	760.58508	55.05512:185836 57.07079:92129 58.06371:121225 58.06664:521554 60.08164:2931016 67.05521:208080 69.07066:287455 71.07362:533194 81.07025:285430 83.08636:292333 86.09715:11695178 87.10043:510741 95.086:387669 97.10144:100500 98.98464:438968 104.10741:1450208 109.10176:81877 121.10108:236384 124.99973:5709500 135.1171:94779 184.07431:142649568 185.07584:6907550 186.07785:1150059 339.2876:96801 496.34:240273 575.50354:1093706 576.50208:295616 577.5274:89894 760.57654:4236432 761.59027:1258071	-0.473319829
Benzofuran	3.807	119.04947	[M+H]+	119.04969	55.93536:4452 56.94319:129235 65.0397:32690 70.19209:9527 72.08107:6114 72.93791:182897 73.0845:16962 73.9381:6422 74.06065:16416 74.95362:55555 76.40462:4949 79.05463:7581 90.94827:256939 91.05531:562307 92.05799:29619 93.07068:8816 97.00809:5425 103.05459:10915 104.06206:8426 108.95852:45509 113.96467:33936 117.07034:28739 118.94261:56399 119.03614:5335 119.04996:197578 119.05955:21609 119.08544:261745 120.02394:6913 120.0439:18399 120.05605:38810 120.08133:10412 120.08881:22691	-1.847967853

Licochalcone C	10.211	337.14664	[M-H]-	337.14453	67.80773:4855 67.81197:13464 78.95761:188025 96.83706:2520 96.84338:5264 96.96821:449802 100.95222:1748 102.94666:42377 120.38843:1504 144.94131:2097 145.93944:1809 146.93759:80055 190.92853:25389 191.93102:1666 253.27499:1523 337.14108:81703 337.20541:13151 338.14752:5147	6.258443523
LPE(16:0)	12.333	454.29364	[M+H]+	454.29279	55.05515:13687 57.03453:35186 57.07099:155027 62.06081:462510 67.0554:23024 67.55484:10241 67.55853:62319 69.07069:22732 71.08636:148852 81.07042:63705 83.08655:54645 85.10211:87380 89.06052:7274 95.08631:90415 97.10175:30239 98.98517:20709 104.10737:14572 109.10182:56174 111.11691:5915 123.11664:22769 124.0155:12993 137.13249:6374 142.0269:19887 155.00974:121891 173.02083:21140 184.07445:7397 198.05215:34649 216.06488:61167 239.23853:44598 257.24771:16558 282.28003:220535 283.28137:16927 313.27133:2155998 314.27811:265342 393.23871:96493 436.28168:59958 454.2966:41858	1.87104004
10-HDoHE	12.419	343.22791	[M-H]1-	343.22781	57.0329:2403 59.01244:22931 59.55045:1406 67.05358:2800 69.0331:7403 69.33054:7431 71.91712:1390 83.04873:2358 85.06441:2214 93.06971:2198 95.04826:1334 95.63631:1318 103.77288:1265 106.03917:1562 107.08459:21437 108.13267:1418 112.15209:1405 119.08486:7839 121.06399:6767 121.10063:5600 133.10052:9230 135.11697:6373 137.09581:7984 145.10028:2971 147.11696:5530 151.11104:6500 153.09117:8507 161.13246:42541 173.13362:2679 175.14966:1877 189.12743:9079 189.16443:1541 205.12326:40636 206.12726:3200 207.13861:2126 227.18027:7210 233.11705:13491 234.12564:22071 281.22589:52001 299.2366:8989 315.2529:1599 325.21982:18720 327.19211:1383 343.22751:54950 343.28189:11351 344.23178:6466	0.291351683

8-bromo-6-chloro-3-(4-hydroxyphenyl)-4-methylchromen-2-one	0.694	362.9408	[M-H2O-H]-	362.94299	68.37685:55992 96.84064:16525 102.9873:31815 112.98351:35733 114.98689:108835 130.98241:895149 131.98352:64636 158.97762:5172292 159.97832:432326 226.96594:1224458 227.96812:104494 294.95297:29579	-6.034005506
Hydroxyethyl glycine	0.843	118.04943	[M-H]-	118.05042	53.36039:2834 54.39975:3498 54.90055:3683 59.89014:2867 64.11858:3099 69.05869:15758 72.00763:101101 74.02337:169158 96.84248:4474 118.02853:23643 118.04048:6334 118.049:47070 118.89901:3093	-8.386247165
Jaeschkeanadiol	1.306	221.18619	[M+H]+	221.189	54.88199:26346 58.06625:53297 60.08162:111877 70.44823:60778 75.60713:27761 104.10737:21329546 105.11083:803215 120.00274:38481 164.02983:374840 178.06493:161931 203.05351:36555 221.06953:181083 221.18742:30127	-12.70406756
Undecanedioic acid	9.962	215.12804	[M-H]-	215.12881	57.03291:1777 59.01194:3262 69.91402:3173 83.02362:1560 113.05989:1611 114.93462:13079 116.93292:9674 123.89989:1867 125.87218:19088 135.04424:21133 146.0331:1692 153.1272:195203 154.13083:16064 157.86188:13517 168.12637:1510 169.15871:1869 171.13736:6825 171.86588:1881 174.02946:1565 189.07812:1452 197.11792:241937 198.12123:21968 214.83086:6462 215.01033:129460 215.12688:732484 216.0128:11057 216.13295:62647	-3.579250961
5-(4-Acetyloxy-3-hydroxy-2,5,5,8a-tetramethyl-3,4,4a,6,7,8-	8.294	379.24875	[M+H]2+	379.24902	58.04234:14758 69.64371:13153 73.02938:85404 73.06554:19858 80.05531:117827 80.55698:12527 87.04444:64815 89.06052:824683 90.0637:31073 100.56839:5074 102.06848:104436 107.07061:18638 111.07359:13585 115.07552:4346 117.09196:5463 124.0808:54952 124.58329:5016 130.0858:23341 131.07092:27363 133.08612:515547 134.08902:34345 146.09303:25941 151.09686:5317 153.59889:4309	-0.711933283

hexahydronaphthalen-1-yl)-3-methylpentanoic acid					155.09903:74156 155.60062:4764 164.1035:20255 175.09634:11925 175.61168:6723 177.11302:190768 177.61508:11363 178.11678:13189 195.12411:6632 197.62698:10372 199.12662:81771 199.6288:16839 219.12411:4426 221.13708:84274 221.6405:6756 222.14207:5647 239.15034:14592 243.153:19488 265.16626:17880 283.1774:12990 309.19397:4326 327.20285:12866 340.20837:4510 362.22305:43058 362.72873:5499 379.18301:12597 379.68073:5142	
Cytidine	6.192	302.09921	[M-H]-	302.099	53.28209:1304 70.11461:4917 75.00727:12175 92.6344:1554 124.05043:363958 125.05376:13647 130.50735:1377 131.03354:2059 144.95784:5536 158.97765:2242 187.08675:1794 188.94749:2947 191.84274:1452 234.75287:1417 256.09283:8200 295.04913:1381	0.695136363
Trifluoroacetic acid	1.412	112.98401	[M-H]-	112.98559	51.09224:51641 60.34593:45698 60.46615:43650 61.66316:46242 68.99397:33634780 69.77618:173726 69.99727:195021 78.85527:42105 88.54602:43388 88.91543:49351 95.74553:47302 96.84066:56066 112.98355:2371861	-13.98408417
Trigonelline	1.405	138.0526	[M+H]+	138.05498	58.10867:9374 68.58924:23922 68.59355:63512 69.04567:49103 80.94859:18226 92.05046:12131 94.06546:100791 96.04482:20441 96.08947:10267 110.06042:116079 121.02914:9907 124.69549:9952 138.05551:1579146 139.05009:65703	-17.23950849
Acetone cyanohydrin	1.289	86.06063	[M+Na]+	86.06004	53.00286:21459 57.03445:23328 58.06589:199847 68.05045:462884 69.03426:207161 69.05383:8954 69.0707:172569 69.58807:10498 69.59193:12580 75.04476:7908 80.93408:5588 86.00629:26418 86.02457:49846 86.03278:26047 86.06079:2121944 86.09715:1468294 86.93975:8040 87.04433:129169 87.06429:53305 87.10066:33401	6.855678896
Phenylsulfate	7.552	172.98991	[M-H]-	172.99142	79.9558:561280 93.03313:3420961 94.03615:128001 109.02765:23633 111.07977:88139 128.87682:94284 172.98903:5629738 173.08182:44046	-8.728756605

					173.99283:273510	
Boc-D-Tpi-OH	9.611	315.13461	[M-H]-	315.13501	69.92068:2648 87.00722:1647 96.84068:1651 102.47086:1439 104.6152:1574 112.2626:1355 122.05934:1712 134.05966:3270 147.38307:1383 196.51306:1330 213.10326:5006 219.84464:3224 223.0892:2069 224.09441:5796 228.1022:2227 237.10379:1843 238.11118:9213 241.1331:5645 253.13361:334451 254.13722:36040 271.108:5879 285.12637:32044 286.12888:1628 315.09402:4999 315.13116:1920 315.17868:2973	-1.269297245
3-Methylcytidine	6.175	258.10846	[2M+H]+	258.10846	57.03445:4937 69.04566:5287 69.31252:16055 75.32796:3657 84.04491:4964 85.02902:5236 109.04025:4467 112.05148:540263 113.04687:5912 113.05485:15834 115.03925:6474 126.0669:2580180 127.07061:103785 128.02802:5218 129.01883:4984 133.04982:5472 155.97684:4437 169.0602:8326 207.87709:4023 225.88785:15379 234.95178:7782 235.86984:5305 258.11087:17802 259.11273:8188	0
2-Hydroxy-2-ethylsuccinic acid	6.429	161.0444	[M-H]-	161.045	57.03289:7238 59.01233:90591 65.01308:2997 65.99709:2427 68.96676:7664 71.01241:29415 71.04869:89591 72.99159:3663 73.02769:105275 74.03111:2066 75.00727:9938 81.03321:6680 83.01206:19886 85.02787:191855 87.00717:10112 87.92341:1850 89.02279:18363 91.02859:2764 96.83972:2179 97.02799:7196 99.04398:125795 100.04631:3042 101.02239:325681 102.02669:11639 103.91839:2153 104.98447:32287 113.02337:8659 115.03858:9528 117.05405:43814 118.02825:7027 125.02317:1933 129.01834:209665 130.02083:6374 131.03343:38988 132.97935:92435 134.02428:2286 134.03458:12602 135.01868:1855 143.03369:49387 143.86325:2846 144.0369:2342 157.86183:3011 160.84161:169624 160.89166:22810 160.97533:5275 161.04497:294078 161.08102:9944 161.84081:5623	-3.725666739

					161.89377:11090 162.01848:68758 162.04695:10241	
L-alpha-Amino-1H-pyrrole-1-hexanoic acid	6.82	195.11272	[M-H]-	195.11336	55.73517:1488 57.54532:1437 59.95698:1360 66.03323:346054 67.03639:8825 69.39298:2637 69.39627:4510 72.00743:2307 80.04865:14089 92.92765:9285 96.83882:1636 107.34144:1388 111.04401:3056 114.05492:1603 114.11964:1402 124.03862:2623 125.03379:1870 128.03313:2520 128.07027:93370 128.99066:1419 141.02875:1506 147.09155:1779 148.96709:2563 151.04936:2367 152.07045:1743 160.83972:44199 178.08585:3184 195.11234:1853522 195.80939:44127 196.05914:2121 196.11681:139374	-3.280144425
Acaranoic Acid	12.027	297.2066	[M-H]-	297.20712	69.3311:6063 79.19709:1335 96.83977:1866 106.0392:1727 117.11544:1373 121.86517:1455 169.08571:1825 183.01164:6840 209.18958:7159 235.20682:66011 236.20947:6704 253.21722:11109 279.19696:68980 280.19998:6198 297.15137:271100 297.20474:191268 298.15469:33720 298.20834:23279 298.24744:5591	-1.749621611
premyrsinol-3-propanoate-5-benzoate-7,13,17-triacetate	8.008	693.28247	[M+2H]2+	693.28815	98.05953:18220 116.06884:7629 118.08479:7353 125.07001:43169 126.05502:20764 129.10083:8505 157.09505:12872 167.08214:28256 168.06387:3683 171.07716:15401 175.10509:9166 185.09203:180019 186.09471:9330 195.11113:12598 196.05983:28504 204.11142:3886 213.08578:51219 214.0903:4473 250.11703:3627 264.13312:10774 282.1452:3042 292.13165:26564 293.13104:3259 310.13968:9916 356.19421:9523 357.14096:18193 379.05692:8854 392.12408:12926 394.14243:3351 397.17282:3709 438.13126:30807 439.1308:8283 454.20108:14830 456.13821:19712 478.12903:24991 485.16412:3275 489.18216:7580 491.69772:13640 497.68521:3998 507.19119:4346 511.16855:10222 513.15826:9092 515.69342:15853 519.69159:3400 527.18762:4312 528.70624:11629 529.71075:8924 530.20831:3471	-8.192841606

					537.70807:22740 538.20605:12779 538.71417:13404 541.2146:7751 549.21942:4514 555.21637:19659 555.72205:11919 556.22162:13682 557.216:3211 563.22717:4066 564.21967:19510 564.72607:8078 565.22363:20792 565.72015:4360 568.22974:6955 569.22021:8870 571.22186:4405 577.2251:28971 577.7265:21775 578.22949:19092 578.74048:10412 579.22931:7670 586.23309:53860 586.73358:31385 587.24475:89317 587.74365:50945 588.24438:11789 595.74396:8434 597.23615:9538 604.75269:35068 605.24902:17807 605.75641:4207 608.23846:3508 611.75342:2980 613.75226:55234 614.25793:32514 614.74927:12061 617.75262:9918 618.24805:3680 621.20361:3586 626.24139:8601 626.76123:115866 627.26758:69110 627.76154:17764 634.73944:8337 635.24023:3573 635.77167:683683 636.27386:410657 636.77539:103207 639.20679:3876 642.25238:4496 643.75073:18459 644.24872:15069 644.74493:3401 656.23065:9409 670.27521:3183 674.24451:17378 675.25665:8309 684.28235:37933 684.77509:51428 685.26935:18214 693.28326:397549 693.78461:273768 694.28784:89366	
N-Methyldodecylamine	10.098	200.23772	[M+H]+	200.23727	53.00286:3483 55.05524:68399 55.93536:5111 57.07092:180171 64.34331:3444 70.06621:4135 71.0863:136230 72.08978:5008 74.09753:6652 75.10029:3554 81.0703:3407 83.0498:5474 84.04491:14906 84.08147:4978 85.10195:48732 86.06079:5099 88.96893:5212 101.05988:39025 111.04477:55053 115.97999:4870 119.97158:3500 127.08662:3629 128.95078:50405 129.05498:58101 130.0507:4692 131.97493:13910 135.9462:5362 146.96161:31197 147.06448:17493 149.9845:4130 153.95702:5112 154.08572:16947 154.12383:12896 154.15831:4803 155.11761:4904 156.06557:3913 158.9622:17856 159.96869:5964 169.97705:6251 172.09846:5148 172.19487:3528	2.247333875

					176.97252:5607 177.97934:3610 182.08223:3853 183.10248:7207 200.09239:18118 200.12704:40909 200.23741:1923711 201.11098:7610 201.1272:17826 201.16237:4978 201.24072:194021	
2-methoxyethyl 5-hydroxy-2-methyl-1-{3-[(4-nitrophenyl)amino]propyl}benzo[g]in dole-3-carboxylate	10.938	478.19769	[M+H]+	478.19727	57.0708:9409 69.18242:5856 69.18624:4917 71.0737:4832 71.08677:4025 81.07027:4712 83.08639:3406 86.09717:73969 94.06567:24342 95.08611:4446 104.10734:3338 120.08141:25187 122.05976:3386 124.07574:9874 124.99997:18313 134.0598:9979 138.05548:11989 146.0598:13376 148.07614:23796 152.07059:17137 163.01431:17102 164.07001:31544 166.08553:35491 184.0744:11725 192.0661:12903 194.0815:16145 197.10623:9469 209.10759:5173 225.10167:14801 227.11656:28134 237.09718:15805 238.11156:21221 239.11871:12941 243.11337:9348 251.11795:12374 252.1021:4206 253.09677:18153 253.13478:19997 255.11272:8205 262.10516:8496 267.11389:14850 269.12936:88142 270.13275:4478 271.10803:138628 272.10999:12492 279.11255:15635 283.10956:4772 284.11517:5355 285.12155:42137 286.12881:12962 287.13681:28720 290.14114:3562 297.12219:709894 298.12497:79457 299.13818:24326 300.14685:14059 303.13791:9069 309.1217:3440 313.11877:8711 315.14114:3601 316.14111:10972 317.15134:19882 318.15973:33677 325.11899:10022 327.13541:14318 328.14221:60798 329.15369:4633 330.15897:26380 331.16782:9504 332.17606:12372 337.27686:21164 341.15054:13630 342.16205:31171 343.16739:5111 344.14017:12449 344.17648:9204 346.15311:4717 357.14609:4213 358.15466:23318 359.16537:44450 360.17383:6840 362.15274:11455 372.17493:36727 373.17679:11340 375.16058:5169 376.16852:20842 377.17413:14587 378.1824:18416 386.14716:14099 387.15646:79950 388.16522:15917 390.18015:127594 391.1842:22046	0.75601233

					396.16879:3595 400.16251:29212 401.17429:35462 405.1741:8925 406.21259:3543 414.17929:17305 415.18716:4923 416.19193:10983 418.17923:21706 419.25348:15254 432.18896:37881 433.19586:3923 435.17795:3478 442.17496:4719 445.1618:9580 450.20154:639188 451.20935:103800 460.18533:841971 461.18896:141939 478.1962:1034311 478.32333:128009 479.19937:194286 479.3277:17801	
N-(4-acetylphenyl)-2-{4-[(7-methyl-2-oxochromen-4-yl)methyl]piperazinyl}acetamide	11.816	478.19745	[M-H]-	478.1983	59.01236:11486 78.95705:203007 96.84336:34493 108.04421:10413 120.07954:19553 122.05931:30688 134.05963:16799 136.07523:44258 148.07462:134081 152.07076:65750 152.99445:85658 160.06302:9215 168.04172:145180 180.0656:393336 181.06883:17888 192.06468:16374 213.10345:60864 224.06888:547215 225.07112:16764 227.11858:27287 238.11111:19368 239.11861:51264 241.13289:173584 242.08061:116895 242.13765:9933 243.14856:10718 253.1337:371728 253.21756:5880298 254.13844:35460 254.22171:342811 281.24878:48198 285.12613:697908 286.12897:64001 374.1853:8963 406.17599:17689 434.20975:53718 450.20462:41572 460.18924:107995 461.19269:10069 478.20041:605755 478.2897:1206215 479.2037:94977 479.29327:166057	-1.777505273
LPC(16:1)	11.815	494.32404	[M+H-H2O]+	494.32468	55.05514:177064 56.05018:114433 57.03443:98127 58.06624:496759 59.07416:106090 60.08159:4615964 67.05523:114844 69.07068:353461 71.07355:750409 81.07096:305890 83.08639:481351 86.09711:13574113 87.10064:358965 93.07066:125453 95.08611:343582 97.10237:292194 98.98515:85584 104.10735:65609384 105.10754:208406 105.11061:1741829 107.08634:100486 109.10178:139920 121.10111:276518 124.99968:4819901 135.11713:167408 149.13274:91236 163.01651:343441 181.02643:180073 184.0743:83972024 185.07816:2184726 199.03619:127910 225.89421:109233 240.10014:88347 258.11081:1168248 311.25586:1962870	-1.294695624

					312.26401:181659 417.24075:553075 476.31677:6214278 477.31519:623207 494.32224:23289626 495.32458:2667666	
N-Methyl-a-aminoisobutyric acid	3.098	118.08672	[M+CH3OH+H]+	118.08681	50.31302:1975 52.32974:1951 53.03949:2455 55.05522:25002 58.06591:62155 59.07378:83947 69.3914:13444 72.08171:271276 73.0851:11373 76.93359:20380 79.05531:1932 82.04828:1884 84.24917:1949 87.04519:2883 91.05513:41794 93.07069:1944 94.94385:11337 95.0501:9766 96.04482:2930 100.11274:3541 103.05559:2593 105.04455:3008 117.07035:7699 117.95958:4741 118.06519:28611 118.08725:377614 119.0534:2139 119.06075:20834 119.08489:79182	-0.508100778
(6-bromo-2-oxochromen-3-yl)-N-[(2-iodophenyl)carbonylamino]carboxamide	0.763	510.88028	[2M-H]-	510.88	70.25738:2513 85.58395:1679 92.92609:282523 94.9237:141219 96.92022:19663 102.95473:53541 104.95293:8728 112.98359:15345 123.55268:1852 150.88565:81818 152.8829:53661 154.8799:2157 160.91341:2557 170.94217:3083 204.64769:1897 208.84564:60441 210.84065:68406 212.83794:2875 218.87396:2405 220.86839:3532 222.86812:7705 238.93193:15793 266.80322:101612 268.80252:159276 270.79495:7588 272.79236:3177 276.83197:10408 278.83041:9800 290.85507:2942 306.91742:6587 314.80771:3382 316.80353:15980 324.76413:13012 326.7605:42089 374.76324:18978 376.75922:1929 382.79575:1824 384.72089:5029 384.79245:3789 430.72095:1917 431.28107:2054 432.72028:2903 442.75204:7024	0.548073912
(1-Methyl-1h-Imidazol-2-Yl)-Acetic Acid	1.355	141.06593	[M+NH4]+	141.06586	56.94306:10242 70.06565:10163 71.01637:7841 72.93777:13660 81.04546:246245 82.04828:7138 83.3687:6874 85.5077:6110 95.06074:779890 96.06446:28313 96.08143:11913 111.05587:58738 113.96426:52476 114.0556:6397 114.09142:6739 123.05582:750429 124.05907:50064 131.97493:14213 141.01772:27084 141.0654:424289	0.496222119

					142.04939:24217 142.07027:23455 142.08633:40964 142.12329:31760	
2-Thiocytidine	5.157	260.07025	[M+H]+	260.06998	54.21753:1478 57.97393:198076 58.95694:1506 60.00414:1292 69.35518:5239 70.16251:1347 81.0443:5874 91.02779:1424 101.94944:1571 102.94703:18674 109.03959:33111 145.93939:5701 146.93791:30093 146.95801:1943 183.91199:9607 190.92616:7145 203.79512:1314 206.6306:1548 211.94836:5744 219.84427:22662 221.84288:48786 222.84283:1470 227.63889:1457 229.9595:3103 230.95828:1565 257.81952:3046 258.05563:26800	-1.782562831
Camalexin	0.961	199.03667	[M-H]-	199.03352	51.26761:15140 54.27703:14718 69.33876:19766 69.34205:64151 75.4274:15856 125.87209:28756 126.63362:14976 134.10971:14727 141.56403:13938 161.8409:167982 162.8374:6550182 163.83727:448483 164.83429:5936061 180.21915:16063 186.23778:17750 198.80598:443314 199.80403:10998255	15.82647988
N-Acetylornithine	0.97	173.09186	[M-H]-	173.09312	58.02826:74093 59.01193:4567 74.02327:10123 84.04385:13218 85.07558:5088 87.05493:11951 96.96822:9271 102.05399:10388 105.01718:9719 111.00742:15322 111.05518:28047 111.07953:46574 112.07552:19023 113.07022:24152 114.05498:27418 116.03387:4155 125.07095:8887 127.04955:9706 127.08596:26634 128.03326:38993 128.07025:5069 128.87688:25234 129.06555:23720 129.09218:4872 129.10176:57207 131.08054:999478 132.0842:28398 137.07146:15176 146.0448:12862 153.06601:5338 155.04454:12822 155.08116:10313 156.07687:8372 172.84232:226308 173.05586:18365 173.09258:916747 173.84274:20155 174.0957:47649	-7.279318785
Diflufenzopyr	6.261	333.07974	[M-H2O-H]-	333.08051	57.03289:7186 59.01236:9938 68.28096:12483 71.0126:15262 72.99161:2119 75.00687:108945 85.02808:79156 87.00719:23850 89.02285:3422 95.01223:6176 96.84063:5523 99.00762:8719	-2.311753396

					112.01498:20692 113.02238:74820 117.01809:2289 129.01729:13893 130.99654:2197 141.05417:2417 154.99509:3307 155.0555:3044 157.01306:15150 175.02438:5847 189.07561:5892 205.07018:14497 229.06906:2487 247.08292:8614 265.09213:984657 266.09781:52723 287.06247:77888 288.06653:8677 333.08133:38823	
2-[2-(19-Acetamido-16,18-dihydroxy-5,9-dimethyl-6-oxoicosan-7-yl)oxy-2-oxoethyl]butanedioic acid	6.417	313.66037	[M+2H]2+	313.664	68.05045:15626 69.17917:22869 70.06568:212208 71.06918:6269 76.04046:4228 86.06084:292505 86.09718:283299 87.10066:15396 120.57079:7993 127.08671:42573 143.08092:30182 143.11842:494838 144.12123:30036 155.08107:50125 171.0775:190058 171.11336:78038 172.07919:13693 177.09486:35849 177.59488:4998 180.08766:7043 181.09119:8393 189.08716:205789 190.08972:7325 191.0929:13407 208.71533:4452 211.10933:5253 228.60727:7631 268.12723:45731 269.13345:4793 286.13824:220612 287.1431:28532	-11.57289329
LPE(20:5)	11.605	498.26157	[M-H]-	498.26263	50.33731:1307 59.01216:12832 78.95712:28832 84.12871:1547 85.68768:1431 96.83976:9494 96.95827:2646 122.00052:1485 131.06487:1392 135.11551:1655 135.53058:1526 140.01016:22026 152.99423:7374 160.59833:1391 167.10759:1386 196.0361:70861 197.04189:2531 200.17961:1685 203.17848:5856 214.04787:23359 226.05434:1841 257.22803:51680 258.22839:2279 301.21561:254243 302.21994:19731 304.77664:1425 309.27682:2237 333.94672:1461 438.2973:1950 498.26953:141207 499.31595:46032	-2.127392135
PC(16:0/16:0)	12.327	734.56866	[M+H]+	734.5694	55.05513:28319 57.03441:4907 57.07082:91371 58.0637:7184 58.06664:33698 59.05014:30054 59.07457:11838 60.08158:145273 67.05526:62290 69.07063:79607 71.07356:25180 71.08619:82240 79.05525:13933 81.07092:91459 83.0864:85645 85.10187:52309	-1.007392902

					86.09708:667876 87.10017:27740 88.04018:20906 89.06047:6326 93.07062:19697 95.0861:126044 96.84241:10143 97.10135:49240 98.98441:23605 104.10722:58116 107.08631:13438 109.10164:60416 111.11684:15089 117.09189:4846 121.10086:45471 122.1045:4772 123.11655:34951 124.99966:311008 126.00376:4818 135.1169:34279 136.12067:4492 137.13234:21659 149.13268:17496 155.00946:5986 184.0742:8151596 185.078:423242 186.07773:92554 219.21323:6557 225.51787:4935 237.22223:39120 239.23737:22577 311.25629:17749 312.25867:6909 313.27551:27861 324.25229:39388 325.25793:5730 326.26724:4908 478.33484:6821 496.33994:14081 548.4754:12972 549.48737:613222 550.49023:178490 734.56512:285326 735.56848:80528	
1-Ethyl-4-butyltetralin-6-sulfonic acid	6.895	295.13898	[M-H]-	295.13733	57.03289:3928 59.0121:115248 61.02803:68408 69.45113:6856 72.99178:134762 75.00746:42312 75.04374:14603 101.02263:291050 102.02659:7371 103.03786:2426 105.05408:224578 106.05779:3965 119.03309:3135 130.98241:11231 136.90871:8245 158.97757:25501 159.9785:2817 164.92459:2827 180.8979:2432 183.92369:3123 193.10645:6065 221.02975:12413 249.02434:45969 250.02635:9252 295.14038:1734295 296.14313:143334	5.590617764
Hexadecanedioic acid mono-L-carnitine ester	9.762	430.31705	[M+H-H2O]+	430.31686	57.03445:3491 57.07082:7861 58.06583:2761 60.08162:106090 67.05524:9500 68.25441:16796 69.07069:9685 71.08679:6283 81.07089:18994 83.08623:12925 85.02899:469010 85.10194:3636 86.03278:8518 95.08599:21657 97.06519:6006 97.10149:8857 107.08637:5751 109.10196:18269 111.08137:8575 111.11691:2525 115.07669:1931 121.10114:14834 123.11619:11440 125.09657:9804 129.09111:2697 133.10077:3334 135.11694:19193 137.09595:1905 139.1123:9150 143.10696:2532 144.10229:28807 149.13251:16308	0.441535105

					151.11095:2809 153.12695:10098 163.14888:3066 167.14403:2781 181.1582:2518 205.19441:32611 206.19882:2809 215.17896:3323 223.20697:30993 233.18906:3645 251.20073:15367 269.21301:5698 287.21964:138498 288.224:14326 325.2355:22533 326.23947:2082 353.23438:20764 355.10022:1803 371.24271:9535 430.31958:640262 431.3204:104178	
Glutamylleucine	6.77	259.12973	[M-H]-	259.12991	69.10226:7430 82.02819:3120 84.04388:1577 96.84158:2341 102.94673:41947 103.91844:2373 112.03844:2579 128.0332:286835 129.03636:11780 130.0856:181115 131.08907:6705 146.93759:58674 146.95636:1844 179.11823:7267 179.54643:1484 184.95006:1811 190.92818:11146 197.12822:40663 198.13245:3273 212.94696:1560 221.84296:2726 223.10774:31877 223.84007:2536 224.11031:1995 230.96167:2968 241.11836:53543 242.11986:2952 259.01733:1623 259.1279:28934 260.13193:1955	-0.694632279
Sulfapyridine	3.021	248.05347	[M-H]-	248.04993	55.20311:1349 68.26761:1392 69.84533:2987 72.0071:11734 93.0326:12522 94.67849:1405 102.9467:5681 103.91844:1604 104.92643:1775 106.04127:2052 107.04885:6107 112.98355:5623 119.04801:10599 127.84926:1353 136.07544:1342 151.03886:2652 154.97319:2144 163.0387:57594 164.04234:3055 174.08827:1322 180.06554:567241 180.91183:7523 181.06906:49107 186.02118:32161 187.0237:1914 202.04874:108306 203.05247:9254 204.03268:2646 220.05809:6014 230.0125:2527 248.0213:6778 248.05466:36565 249.05441:2799	14.27132029
13-Dodecan-2-yl-6-(1-hydroxyethyl)-3-	7.909	564.35938	[M+NH4]+	564.36194	59.05004:10177 69.39137:14459 73.02939:10656 73.06551:74308 87.04442:387962 88.04809:5636 89.06053:3956476 90.06364:84868 91.07568:24371 107.07063:101788 113.0605:5035 117.09188:45698 130.08588:117724 131.07077:156491 133.08623:2525413 134.08936:82341	-4.536096109

(hydroxymethyl)-12-methyl-9-propan-2-yl-1-oxa-4,7,10-triazacyclotridecane-2,5,8,11-tetrone					135.10222:15008 151.0968:14859 159.10289:6445 161.1165:6223 175.09644:88570 177.11316:805880 178.11612:30573 179.1265:5850 195.12144:4948 219.12456:24682 221.13734:129387 223.1534:13608 239.14966:33578 265.16614:38112 283.17703:67428 309.18927:33170 327.20245:44180 353.21417:26022 371.23077:21031 415.2529:20908 459.27896:24051 503.30133:44700 547.33215:397386 548.34161:41667 564.35968:50227 565.28693:135479	
2-[2-amino-3-(1H-indol-3-yl)propanamido]acetic acid	6.652	260.10355	[M-H]-	260.10403	58.02821:2810 59.01237:2984 69.16113:7323 70.02782:7468 71.02319:6228 72.00746:2049 73.03897:7097 74.02268:7370 85.03915:2661 86.02267:6462 87.05495:7720 90.93152:8374 92.049:10419 95.02312:44997 99.05439:3188 111.01875:108011 112.02156:2271 113.03387:52285 114.018:5965 116.04949:60815 117.05295:2262 125.03384:2390 128.03351:25599 129.0368:17475 130.06448:3307 130.08557:15757 131.04515:154232 131.08969:12310 132.04803:3020 141.09077:2796 142.06506:9822 143.04526:7634 146.92256:5682 147.93785:1758 156.04547:1912 157.07477:2167 171.09067:2957 181.04807:3421 183.0565:12354 184.06343:2028 186.05472:2198 188.06871:3009 198.12979:3058 199.08568:134089 200.09155:20574 201.06631:1994 203.08266:10126 214.09808:2028 215.08195:5930 216.11145:18759 221.84294:6663 223.83688:3376 224.08165:2643 224.11029:3046 240.07825:2906 242.09137:83699 243.0959:10049 260.10406:359406 261.10574:45369	-1.845415467
Citric acid	1.754	191.01862	[M-H]-	191.01971	57.03297:311819 69.37545:67840 85.02798:2015586 86.03098:60153 87.0072:3213958 88.01028:59038 102.94667:79012 111.00742:8776466 112.01031:315466 129.01711:643513 130.99655:101955 147.02899:82477 154.99693:60306 173.00818:256121 191.01881:1786369 192.02176:78306	-5.706217437
