# Supplementary material for: Yigong San Extract Modulates Metabolism, Antioxidant Status, and Immune Function to Improve Health in Diarrheic Calves
Source: Metabolites. 2025 Sep 18;15(9):618. doi: 10.3390/metabo15090618 (PMC12471574; doi:10.3390/metabo15090618)
Supplement: Supplementary file 1 [file metabolites-15-00618-s001.zip › Supplementary Information3.pdf]

北京美康堂医药科技有限公司

检验报告书

原料☐ 辅料☐ 半成品☐ 成品■ 包材☐　　编号：SOR-ZL/Z029-03

检验单号：C2306075

品　名	炙甘草	规　格	1kg/袋
编号/批号	230627001	包　装	纸袋
产　地	内蒙	检品数量	150g
请验日期	2023.06.29	报告日期	2023.07.05
检验项目	全检	检品来源	待包装品库
检验依据	《中国药典》2020年版一部及四部		
检验项目	标准规定	检验结果	
【性状】	应符合炙甘草标准要求	符合炙甘草标准要求	
【鉴别】	供试品色谱中，在与对照药材色谱相应的位置上，显相同颜色的荧光斑点；在与对照品色谱相应的位置上，显相同的橙黄色荧光斑点	符合标准要求	
【检查】			
药屑杂质	不得过3%	0.4%	
水分	不得过10.0%	8.5%	
总灰分	不得过5.0%	2.9%	
二氧化硫残留量	不得过150mg/kg	16mg/kg	
【含量测定】	本品按干燥品计算，含甘草苷($C_{21}H_{22}O_9$)不得少于0.50%，	0.72%	
	甘草酸($C_{42}H_{62}O_{16}$)不得少于1.0%	2.8%	
结论：依据《中国药典》2020年版一部及四部标准检验，结果符合标准规定			
负责人：	检验人：	复核人：	

北京美康堂医药科技有限公司 检验专用章

北京美康堂医药科技有限公司

检验报告书

原料☐ 辅料☐ 半成品☐ 成品■ 包材☐　　编号：SOR-ZL/Z029-03

检验单号：C2212062

品　名	茯苓	规　格	1kg/袋
编号/批号	221221002	包　装	纸袋
产　地	安徽	检品数量	150g
请验日期	2022.12.21	报告日期	2023.01.11
检验项目	全检	检品来源	待包装品库
检验依据	《中国药典》2020年版一部及四部		
检验项目	标准规定		检验结果
【性状】	应符合茯苓标准要求		符合茯苓标准要求
【鉴别】	1. 应具有茯苓的显微特征		具有茯苓的显微特征
	2. 取本品粉末少量，加碘化钾碘试液1滴，显深红色		显深红色
	3. 供试品色谱中，在与对照药材色谱相应的位置上，显相同颜色的主斑点		显相同颜色的主斑点
【检查】			
药屑杂质	不得过3%		0.3%
水分	不得过18.0%		16.9%
总灰分	不得过2.0%		0.8%
二氧化硫残留量	不得过150mg/kg		16mg/kg
【浸出物】	醇溶性热浸法测定，不得少于2.5%		4.6%
结论：依据《中国药典》2020年版一部及四部标准检验，结果符合标准规定			
负责人：王□□	检验人：杨□□		复核人：□□

（印章：北京美康堂医药科技有限公司 检验专用章）

（印章：北京美康堂医药科技有限公司 检验专用章）

北京美康堂医药科技有限公司

检验报告书

原料☐ 辅料☐ 半成品☐ 成品■ 包材☐ 编号：SOR-ZL/Z029-03

检验单号：C2209018

品　名	麸炒白术	规　格	1kg/袋
编号/批号	220911004	包　装	纸袋
产　地	安徽	检品数量	150g
请验日期	2022.09.22	报告日期	2022.09.26
检验项目	全检	检品来源	待包装品库
检验依据	《中国药典》2020年版一部及四部		
检验项目	标准规定	检验结果	
【性状】	应符合麸炒白术标准要求	符合麸炒白术标准要求	
【鉴别】	供试品色谱中，在与对照药材色谱相应的位置上，显相同颜色的斑点，并应显有一桃红色主斑点（苍术酮）	符合麸炒白术标准要求	
【检查】			
药屑杂质	不得过3%	0.5%	
水分	不得过15.0%	6.2%	
总灰分	不得过5.0%	3.7%	
色度	照溶液颜色检查法试验，与黄色10号比色液比较，不得更深	符合标准要求	
二氧化硫残留量	不得过400mg/kg	13mg/kg	
【浸出物】	醇溶性热浸法测定，不得少于35.0%	42.7%	
结论：依据《中国药典》2020年版一部及四部标准检验，结果符合标准规定			
负责人：	检验人：	复核人：	

北京美康堂医药科技有限公司 检验专用章

北京美康堂医药科技有限公司 检验专用章

北京美康堂医药科技有限公司

检验报告书

原料☐ 辅料☐ 半成品☐ 成品■ 包材☐　　编号：SOR-ZL/Z029-03

检验单号：C2209043

品　名	党参片	规　格	1kg/袋
编号/批号	220925002	包　装	纸袋
产　地	甘肃	检品数量	150g
请验日期	2022.10.03	报告日期	2022.10.10
检验项目	全检	检品来源	待包装品库
检验依据	《中国药典》2020年版一部及四部		

检验项目	标准规定	检验结果
【性状】	应符合党参片标准要求	符合党参片标准要求
【鉴别】	1. 应具有党参的显微特征<br>2. 供试品色谱中，在与对照品色谱相应的位置上，显相同颜色的斑点或荧光斑点	具有党参的显微特征<br>符合标准要求
【检查】		
水分	不得过16.0%	15.6%
总灰分	不得过5.0%	4.0%
药屑杂质	不得过3%	0.8%
二氧化硫残留量	不得过400mg/kg	16mg/kg
【浸出物】	醇溶性热浸法测定，不得少于55.0%	77.1%

结论：依据《中国药典》2020年版一部及四部标准检验，结果符合标准规定

负责人：[signature]　　检验人：[signature]　　复核：[signature]

北京美康堂医药科技有限公司 检验专用章

北京美康堂医药科技有限公司 检验专用章

北京美康堂医药科技有限公司

检验报告书

原料☐ 辅料☐ 半成品☐ 成品■ 包材☐　　编号：SOR-ZL/Z029-03

检验单号：C2306054

品　名	陈皮	规　格	1kg/袋
编号/批号	230615001	包　装	纸袋
产　地	广东	检品数量	150g
请验日期	2023.06.20	报告日期	2023.06.26
检验项目	全检	检品来源	待包装品库
检验依据	《中国药典》2020年版一部及四部		
检验项目	标准规定		检验结果
【性状】	应符合陈皮标准要求		符合陈皮标准要求
【鉴别】	1.应具有陈皮的显微特征		具有陈皮的显微特征
	2.供试品色谱中，在与对照品色谱相应的位置上，显相同颜色的荧光斑点		显相同颜色的荧光斑点
【检查】			
药屑杂质	不得过3%		0.4%
水分	不得过13.0%		10.5%
黄曲霉毒素	本品每1000g含黄曲霉毒素B_1不得过5μg，黄曲霉毒素G_2、黄曲霉毒素G_1、黄曲霉毒素B_2和黄曲霉毒素B_1的总量不得过10ug		未检出<br>0.152ug/kg
二氧化硫残留量	不得过150mg/kg		16mg/kg
【含量测定】	本品按干燥品计算，含橙皮苷($C_{28}H_{34}O_{15}$)不得少于2.5%		6.4%

结论：依据《中国药典》2020年版一部及四部标准检验，结果符合标准规定

负责人：[signature]　　检验人：[signature]　　复核人：[signature]

北京美康堂医药科技有限公司 检验专用章

北京美康堂医药科技有限公司 检验专用章
